# Zintl Clusters as a Platform for Lewis Acid Catalysis

**DOI:** 10.1021/acs.inorgchem.4c00433

**Published:** 2024-05-30

**Authors:** Benjamin L. L. Réant, George F. S. Whitehead, Meera Mehta

**Affiliations:** † Department of Chemistry, University of Manchester, Oxford Road, Manchester M13 9PL, United Kingdom; ‡ Department of Chemistry, University of Oxford, 12 Mansfield Road, Oxford OX1 3TA, United Kingdom

## Abstract

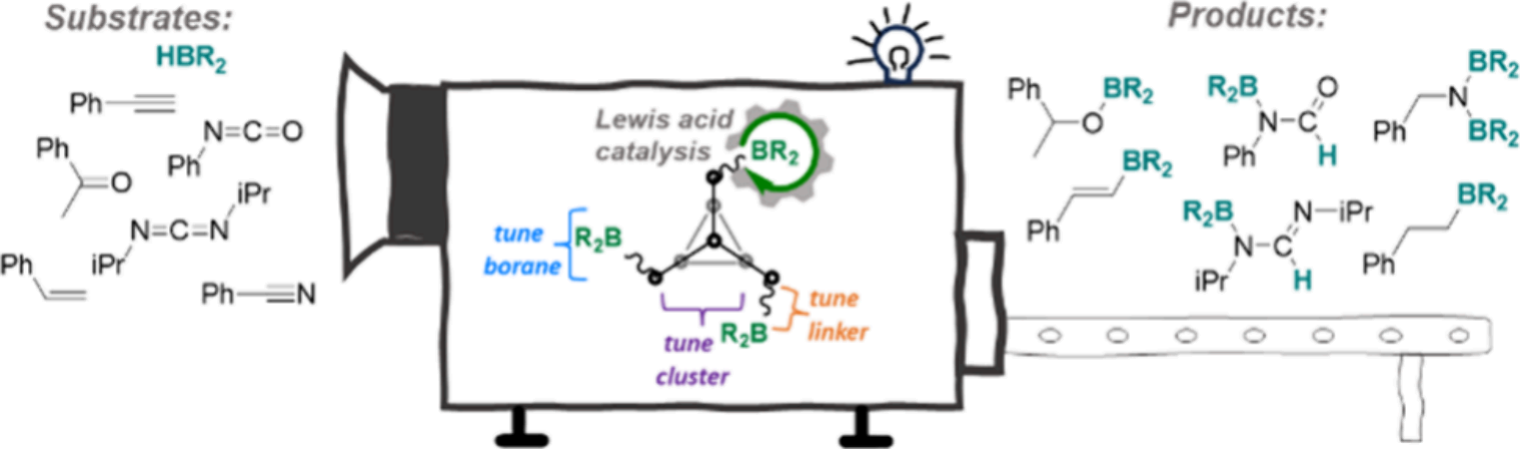

Clusters of the main group elements phosphorus and arsenic, commonly categorized as Zintl clusters, have been known for over a century. And, only now is the application of these systems as catalysts for organic synthesis being investigated. In this work, boranes are tethered via an aliphatic linker to Zintl-based clusters and their Lewis acidity is examined experimentally, by the Gutmann–Beckett test and competency in the hydroborative reduction of six organic substrates, as well as computationally, by fluoride ion affinity and hydride ion affinity methods. The effects of tuning the aliphatic linker length, substituents at the boron, and changing the cluster from a seven-atom phosphorus system to a seven-atom arsenic system on reactivity are studied.

## Abstract 2

We systematically tuned the structural features of boron Lewis acids mounted on Zintl-based clusters and examined the impact on their Lewis acidity both experimentally and computationally.

## Introduction

Organoboranes, compounds featuring B–C bonds, are ubiquitous in organic synthesis,^1−3^ functional materials,^4^ and neutron capture therapy.^5^ Within organic synthesis, boranes are commonly employed to facilitate the reduction of carbonyls to protected alcohols,^6^ as precursors in cross-coupling chemistry,^7^ and as catalysts in their own right.^2,8−10^ Boron-based catalysts are of growing interest to the synthetic community because they are often inexpensive and more sustainable alternatives to their transition metal counterparts.^10^

Anionic clusters of main group elements, called Zintl clusters, have been known for over a century, but investigations into their subsequent chemistry has remained largely ignored. Recently, their chemistry is experiencing a resurgence with these clusters being employed as ligands on d- and f-block metals allowing access to unique physical properties, as components for small molecule capture and “frustrated Lewis pair” chemistry, and as precursors to controlled nanostructures and light emitting diodes.^11−13^ One growing area of interest is the application of these clusters in catalytic science. These clusters are of interest because they can be understood as potential molecular prototypes for heterogeneous materials.^14−16^ For instance, red phosphorus structurally resembles clusters of phosphorus coupled and cross-linked together.^17^ Red phosphorus is an inexpensive, abundant, and shelf-stable material, but it is also highly insoluble in common laboratory solvents, making it difficult to study. Meanwhile, heptapnictogen clusters ([Pn_7_]; Pn = pnictogen), especially once functionalized, have increased solubility, enabling solution-state studies into their reactivity. There has been recent interest in developing red phosphorus as a platform for photocatalysis,^18^ and to this end, transition metals have been anchored to it to increase performance.^19^ Additionally, Velian and co-workers have surface modified black phosphorus with Group 13 Lewis acids both to better understand Lewis acid–base interactions at the surface and as a method to tune the electronic properties of black phosphorus,^20^ whereas to the best of our knowledge, well-defined transition metal-free catalysts have yet to be anchored onto a heterogeneous phosphorus platform, and anchoring them to [P_7_] clusters may provide insight on what to expect from their reactivity.

**Figure 1:**
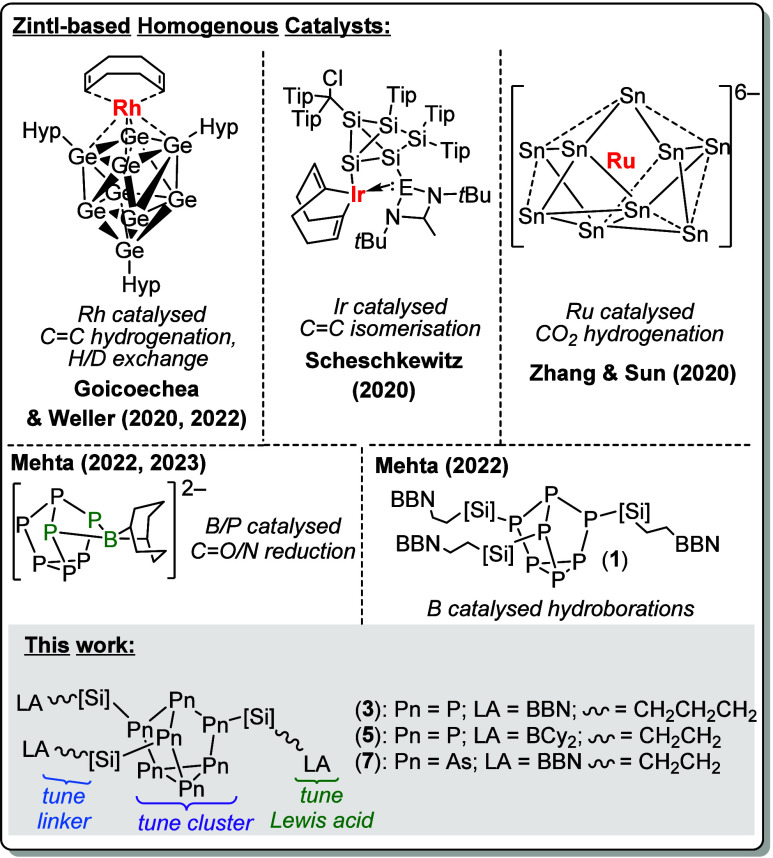
Examples of previously reported Zintl-based homogeneous catalysts and this work. Hyp = Si(SiMe_3_)_3_, Tip = 2,4,6-triisopropylphenyl, 9-BBN = borobicyclo(3.3.1)nonane, [Si] = SiMe_2_.

Within homogeneous catalytic science, thus far Zintl clusters have largely been coordinated to a transition metal and then applied in transition metal catalysis, where the role of the cluster is as a ligand. In 2020, Goicochea and Weller coordinated a deltahedral [Ge_9_] cluster to rhodium which facilitated the hydrogenation of cyclic alkenes, and later in 2022, the same system was found to scramble H/D (Figure 1).^14,21^ Fässler and co-workers reported a range of [NiGe_9_] clusters with varying phosphine ligands on Ni and employed these systems in alkene isomerization catalysis.^22^ Scheschkewitz and co-workers also mediated alkene isomerization catalysis but this time with iridium coordinated to a silicon cluster.^23^ Also in 2020, Zhang and Sun encapsulated ruthenium within a [Sn_9_] cluster, dispersed it on a CeO_2_ surface, and found that it mediated the reverse water-shift reaction.^24^ In 2022, we reported the first example of transition-metal free Zintl catalysis where the cluster participated in the catalytic cycle by aiding the activation of substrates.^15^ In this 2022 work, the synthesis of a boron-functionalized [P_7_] cluster was reported and found to facilitate the reduction of CO_2_ to a methoxyborane with high activity and complete selectivity. Later, we reported the same cluster as an active catalyst in the hydroborative reduction of pyridines, imines, and nitriles.^25^ We also demonstrated that a range of Zintl ions and phases could initiate the hydrophosphination of HPPh_2_ across alkynes, alkenes, and imines.^26^

Also in 2022, we prepared a metal-free heptaphosphane cluster with borane moieties linked via an aliphatic chain, ({C_8_H_14_}BCH_2_CH_2_SiMe_2_)_3_P_7_ (**1**).^27^ In this system, we found that the cluster was an innocent platform while the tethered boranes mediated the hydroboration of heteroallenes (carbodiimides and isocyanates), ketones, alkenes, alkynes, and nitriles. Herein, we probe how tuning the structural features of **1** influences the Lewis acidity at the reactive borane groups. Three variables are tuned: (1) the C2 tethers are changed to C3 tethers, (2) boron units are converted from 9-borabicyclo[3.3.1]nonanes to dicyclohexylboranes, and (3) the cluster platform is altered from [P_7_] to [As_7_]. The Lewis acidity of each of the borane tether heptapnictogens was then quantified both experimentally, using the Gutmann–Beckett method, and computationally, using fluoride ion affinity and hydride ion affinity data. Finally, these derivatives were tested in the hydroboration of a carbodiimide, isocyanate, ketone, alkene, alkyne, and nitrile, and the catalytic activity was directly compared to the parent catalyst **1**. Although the catalytic performance of these systems was found to be modest, catalytic competency could be used as an additional experimental measure of how Lewis acidity is being affected by these structural manipulations.

## Results and Discussion

### Synthesis of Lewis Acid-Tethered Cluster Catalysts

We were motivated to probe how tuning the structural components of ({C_8_H_14_}BCH_2_CH_2_SiMe_2_)_3_P_7_ (**1**) affected its Lewis acidity and ultimately catalytic competency. There are four regions where the tris-tethered [P_7_] compound **1** can be modified (Figure 1, this work): (i) the distance between the pnictogen cluster and the Lewis acid, (ii) the Lewis acidic moiety, (iii) the heptaphosphide cluster, and (iv) the silyl linker to the cluster.

**Scheme 1:**
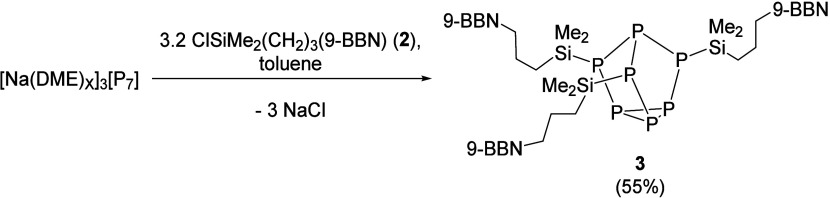
Synthesis of ({C_8_H_14_}BCH_2_CH_2_CH_2_SiMe_2_)_3_P_7_ (**3**)

**Figure 2:**
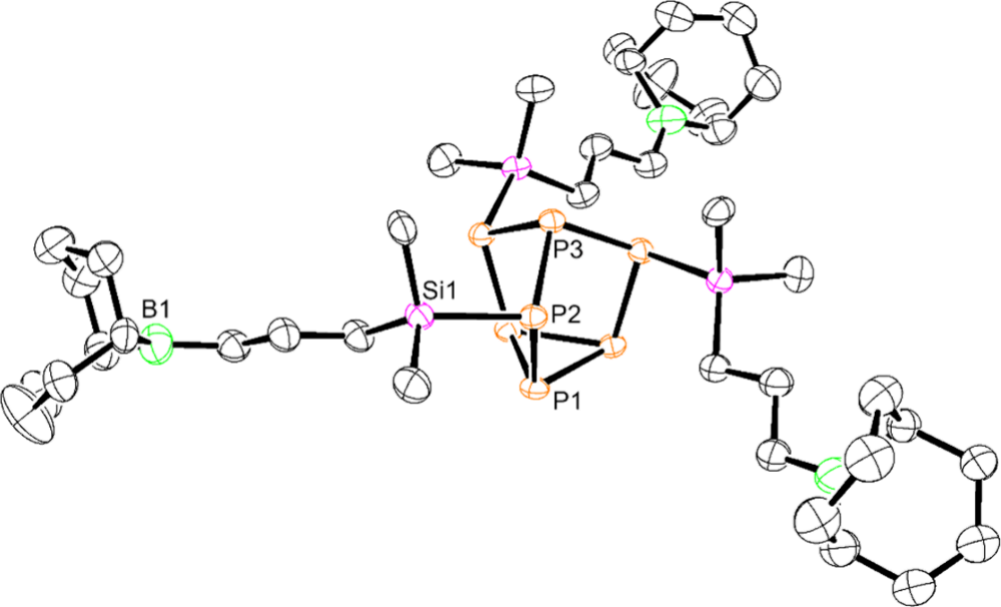
Molecular structure of ({C_8_H_14_}BCH_2_CH_2_CH_2_SiMe_2_)_3_P_7_ (**3**). Anisotropic ellipsoids pictured at 50% probability. Hydrogen atoms omitted for clarity. Phosphorus, orange; silicon, pink; boron, green; carbon, black.

First, we targeted the synthesis of a boron-tethered Zintl catalyst where the alkyl linker between the boron and the [P_7_] cluster was extended from C2 to C3. To achieve this, allyl(chloro)dimethylsilane was hydroborated with 0.5 equiv of 9-borobicyclo(3.3.1)nonane dimer [(9-BBNH)_2_] to give the chlorosilane with a tethered 9-BBN, ClSiMe_2_CH_2_CH_2_CH_2_B{C_8_H_14_} (**2**). Next, ClSiMe_2_CH_2_CH_2_CH_2_B{C_8_H_14_} (**2**) was reacted with literature known [Na(DME)_*x*_]_3_[P_7_]^28^ in toluene to eliminate NaCl and give ({C_8_H_14_}BCH_2_CH_2_CH_2_SiMe_2_)_3_P_7_ (**3**), see Scheme 1. Consistent with the nuclear magnetic resonance (NMR) data collected for **1**, the ^11^B NMR spectrum revealed a signal at 88.5 ppm (**1**: δ_B_ = 86.5 ppm) and the ^29^Si NMR spectrum revealed a doublet at 8.7 ppm with ^1^*J*_PSi_ = 48 Hz (**1**: δ_Si_ = 11.8 ppm (d), ^1^*J*_PSi_ = 49 Hz). This data is consistent with the silane being successfully installed on the pnictogen cluster and three-coordinate boranes where there is no inter- or intramolecular adduct formation with the cluster. Crystals suitable for X-ray diffraction (XRD) were obtained from a concentrated pentane solution at −35 °C and confirmed the solid-state structure of **3** (Figure 2). Compound **3** crystallized in the *P*6_3_ space group with average P–Si bond lengths of 2.828(18) Å, consistent with previously reported {P_7_Si_3_} compounds.^12,28^

**Scheme 2:**
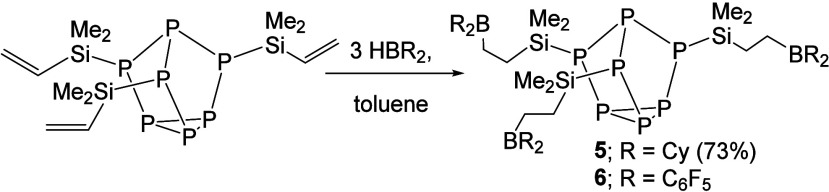
Synthesis of ({Cy_2_B}CH_2_CH_2_SiMe_2_)_3_P_7_ (**5**) and ({(C_6_F_5_)_2_B}CH_2_CH_2_SiMe_2_)_3_P_7_ (**6**) An analytically pure sample of compound **6** could not be obtained.

Next, we focused on the synthesis of a boron-functionalized [P_7_] cluster where the Lewis acidic component is tuned from 9-BBN to dicyclohexylborane (BCy_2_). To this end, ClSiMe_2_CH_2_CH_2_BCy_2_ (**4**) was prepared from the reaction of ClSiMe_2_CHCH_2_ and HBCy_2_. Efforts to install **4** directly onto the [P_7_] cluster via salt metathesis with [Na(DME)_*x*_]_3_[P_7_] resulted in an intractable reaction mixture with the desired ({Cy_2_B}CH_2_CH_2_SiMe_2_)_3_P_7_ (**5**) cluster present and unreacted **4**. As an alternative approach, the previously reported vinyl-functionalized [P_7_] cluster (CH_2_CHSiMe_2_)_3_P_7_ was prepared^27^ and then hydroborated with 3 equiv of HBCy_2_ in toluene, affording analytically pure ({Cy_2_B}CH_2_CH_2_SiMe_2_)_3_P_7_ (**5**) after workup in a 73% yield as a yellow oil (Scheme 2).

Despite multiple efforts, single crystals suitable for XRD studies of **5** could not be obtained. Mass spectrometry confirmed the presence of **5**, and NMR spectroscopy revealed the expected resonances. ^1^H NMR spectroscopy confirmed the disappearance of the vinyl signals (ca. region of 5.5–6.5 ppm), and ^31^P{^1^H} NMR spectroscopy revealed three multiplet resonances consistent with a tris-functionalized symmetric [P_7_] cluster. The ^29^Si{^1^H} NMR spectrum showed a downfield shift to a doublet at 12.2 ppm with ^1^*J*_SiP_ of 46 Hz, slightly larger than the vinyl precursor ((CH_2_CHSiMe_2_)_3_P_7_; δ_Si_ = −2.4 (d), ^1^*J*_SiP_ = 43 Hz).^27^ Finally, the ^11^B NMR spectrum revealed a broad resonance at 81.5 ppm, consistent with three-coordinate boron and significantly downfield from the HBCy_2_ (δ_B_ (CDCl_3_) = 29.9) starting material.^29^ (CH_2_CHSiMe_2_)_3_P_7_ was also reacted with HB(C_6_F_5_)_2_ in an effort to install a borane with perfluoroarene electron-withdrawing groups (Scheme 2). Although the expected ({(C_6_F_5_)_2_B}CH_2_CH_2_SiMe_2_)_3_P_7_ (**6**) could be observed by ^1^H, ^19^F, ^29^Si{^1^H}, and ^31^P NMR spectroscopies (see Supporting Information section 2.5), analytically pure material could not be isolated, precluding further investigations with ({(C_6_F_5_)_2_B}CH_2_CH_2_SiMe_2_)_3_P_7_ (**6**).

**Scheme 3:**
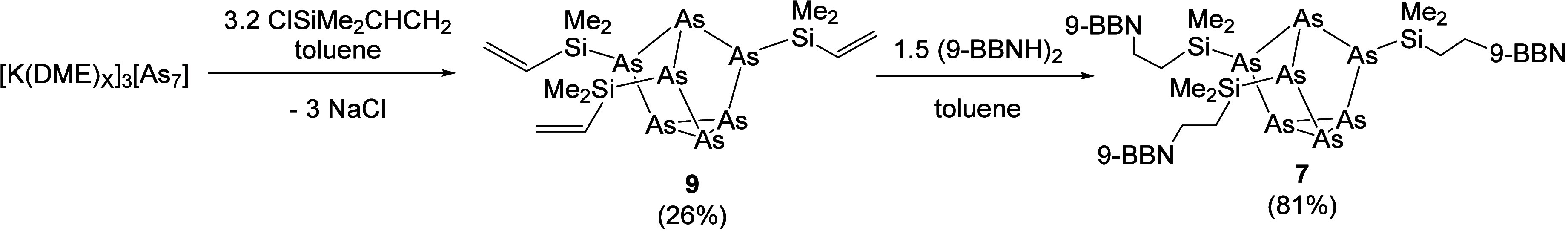
Synthesis of ({C_8_H_14_}BCH_2_CH_2_SiMe_2_)_3_As_7_ (**7**)

Previously, parallel reactivity between the [P_7_] and the [As_7_] clusters has been reported;^13,30^ thus, we set out to make the [As_7_] analogue of **1**. To this end, we first attempted to synthesize ({C_8_H_14_}BCH_2_CH_2_SiMe_2_)_3_As_7_ (**7**) from the salt metathesis reaction of [K(DME)_*x*_]_3_[As_7_] and the previously reported ClSiMe_2_CH_2_CH_2_B{C_8_H_14_} (**8**) chlorosilane;^27^ however, no conversion was detected by ^29^Si{^1^H} NMR spectroscopy even after 1 week in toluene. Next, [K(DME)_*x*_]_3_[As_7_] was allowed to react with an excess of ClSiMe_2_CHCH_2_ in toluene and found to yield (CH_2_CHSiMe_2_)_3_As_7_ (**9**) as a red powder in 26% yield after extraction with toluene (Scheme 3). And, subsequent hydroboration with 9-BBNH dimer in toluene afforded **7** (({C_8_H_14_}BCH_2_CH_2_SiMe_2_)_3_As_7_) as a pale red powder in an 81% yield.

**Figure 3:**
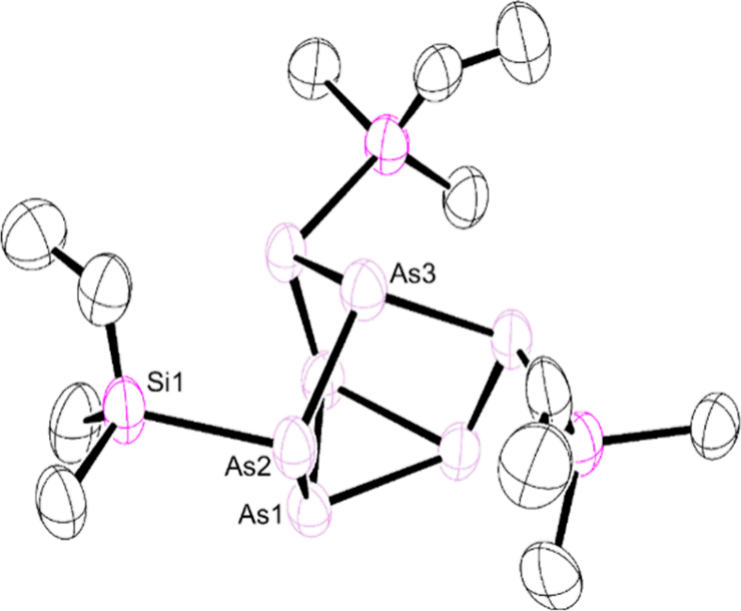
Molecular structure of (CH_2_CHSiMe_2_)_3_As_7_ (**9**). Anisotropic displacement ellipsoids pictured at 50% probability. Hydrogen atoms and pentane omitted for clarity. Arsenic, plum; silicon, pink; carbon, black.

Crystals of the vinyl precursor **9** suitable for XRD studies could readily be obtained from a concentrated toluene solution at −35 °C (Figure 3). The single-crystal XRD data confirmed installation of the silyl groups at the [As_7_] cluster and preservation of the vinyl moieties with average C–C bond lengths of 1.332(22) Å (consistent with C=C double bonds^31^). Successful installation of the silyl group and preservation of the vinyl moiety could be further validated by NMR spectroscopic studies. The ^1^H NMR spectrum revealed the three expected vinyl signals between 5.5 and 6.5 ppm, while the ^29^Si{^1^H} NMR spectrum showed a singlet at 2.2 ppm, slightly upfield from that observed for the [P_7_] analogue (ca. 4 ppm).^27^ Unfortunately, despite multiple efforts, crystals suitable for single-crystal XRD studies could not be obtained for **7**. As with catalysts **1**, **3**, and **5**, NMR spectroscopy confirmed successful hydroboration of the vinyl moieties and generation of cluster **7**. The vinyl signals were no longer detected in the ^1^H NMR spectrum, the ^29^Si{^1^H} NMR spectrum displayed a downfield shift of the singlet resonance to 17.3 ppm, and the ^11^B NMR spectrum revealed a broad signal at 86.9 ppm akin to the boron resonance observed for the parent system **1** (**1**: δ_B_ = 86.5 ppm).

**Scheme 4:**
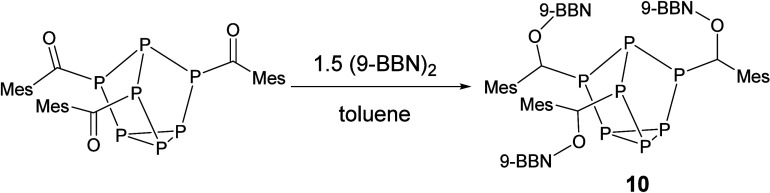
Synthesis of (Mes{(C_8_H_14_)BO}CH)_3_P_7_ (**10**) An analytically pure sample of compound **10** could not be obtained.

**Figure 4:**
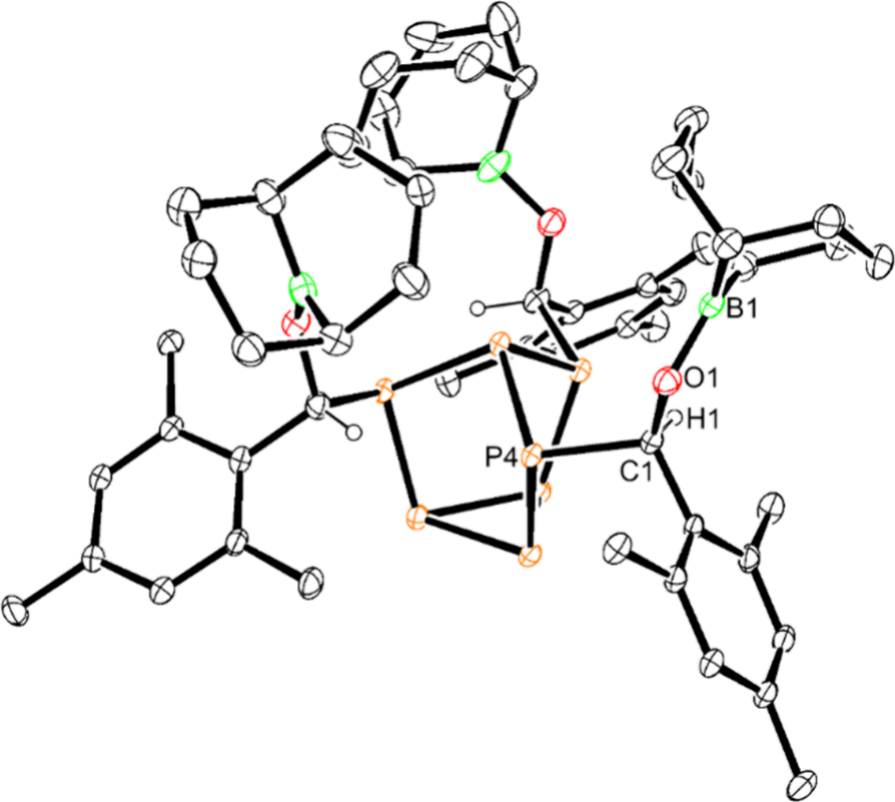
Molecular structure of (Mes{(C_8_H_14_)BO}CH)_3_P_7_ (**10**). Anisotropic displacement ellipsoids pictured at 30% probability. Hydrogen atoms and pentane omitted for clarity. Phosphorus, orange; boron, green; carbon, black.

Finally, we explored tuning the phosphorus element bond that connects the tethered boron to the cluster. To achieve this, the carbon-functionalized [P_7_] cluster previously reported by the Grützmacher group (MesC(O))_3_P_7_ was prepared.^32^ This cluster features three carbonyl moieties, which were investigated in hydroboration with 9-BBNH dimer and found to afford (Mes{(C_8_H_14_)BO}CH)_3_P_7_ (**10**) as a yellow solid (Scheme 4). Crystals suitable for XRD analysis could be obtained from a concentrated Et_2_O solution of the reaction mixture at −35 °C, confirming the solid-state structure of **10** (Figure 4). The molecular structure of **10** revealed the {OBBN} units to be oriented upward near the apical phosphorus, which results in shorter B–P_apical_ distances (range 3.668(5)–4.036(4) Å; average 3.889(8) Å) compared to **1** (B–P_apical_ average 7.533(26) Å) and **3** (B–P_apical_ average 8.231(8) Å). Although **10** could also be identified by mass spectrometry, despite multiple recrystallization efforts, clean characterization by NMR spectroscopy and elemental analysis could not be obtained, which precluded further investigations into its reactivity.

### Lewis Acidity Tests

The Lewis acidity of clusters **3**, **5**, and **7** was probed experimentally through the adapted Gutmann–Beckett method^33^ and computationally by the fluoride and hydride ion affinities (FIA and HIA). The findings are summarized in Table 1. Further, the untethered chlorosilanes **2** and **4** were assessed in a similar manner, and the results were compared to those previously reported for **1** and chlorosilane **8**.

**Table 1:** Gutmann–Beckett Acceptor Number, Fluoride Ion Affinity, and Hydride Ion Affinity of Compounds **1**–**8** and **10**

catalyst	acceptor number (AN)	FIA (kJ mol^–1^)^a^	HIA (kJ mol^–1^)^a^
({C_8_H_14_}BCH_2_CH_2_SiMe_2_)_3_P_7_ (**1**)	28.8^b^	350^c^	352
ClSiMe_2_CH_2_CH_2_CH_2_B{C_8_H_14_} (**2**)	16.3	340	341
({C_8_H_14_}BCH_2_CH_2_CH_2_SiMe_2_)_3_P_7_ (**3**)	20.5	321	323
ClSiMe_2_CH_2_CH_2_BCy_2_ (**4**)	12.1	344	347
({Cy_2_B}CH_2_CH_2_SiMe_2_)_3_P_7_ (**5**)	18.6	356	358
({(C_6_F_5_)_2_B}CH_2_CH_2_SiMe_2_)_3_P_7_ (**6**)	d	438	465
({C_8_H_14_}BCH_2_CH_2_SiMe_2_)_3_As_7_ (**7**)	18.0	348	346
ClSiMe_2_CH_2_CH_2_B{C_8_H_14_} (**8**)	19.4^b^	340^c^	341
(Mes{(C_8_H_14_)BO}CH)_3_P_7_ (**10**)	d	344	334

a Calculated at the BP86/SV(p) level of theory.

b Acceptor number (AN) redetermined.

c FIA previously reported.^27^

d Not determined due to difficulties obtaining analytically pure material.

As reported by Melen and co-workers, for the Gutmann–Beckett test, a capillary containing triphenylphosphine in C_6_D_6_ was introduced into the NMR tube to calibrate the ^31^P{^1^H} NMR spectra, which was found to produce more consistent results for a series of fluorinated triaryl borates.^34^ We internally validated this method with the established B(C_6_F_5_)_3_ acceptor number (AN) value of 77.2, consistent with previous reports.^35^ Assessing the AN of all catalysts **1**–**5**, **7**, and **8,** an increase in the AN value is observed when comparing the chloro tether to when the tether is attached to the [Pn_7_] cluster (i.e., AN = 12.1 (**4**) vs 18.6 (**5**)). Comparing clusters **1** and **3** revealed that increasing the chain length from ethyl to propyl results in a decrease of the AN by 8.3, while exchange of the Lewis acidic components from {9-BBN} to {BCy_2_} decreases the AN by 10.2. Interestingly, a reduction in the AN value for the heavier [As_7_] catalyst **7** (AN = 18.0) vs the [P_7_] congener **1** (AN = 28.8) was observed. Upon addition of triethylphosphine oxide (Et_3_PO), the red solution of **7** becomes dark brown; this occurs regardless of the sample concentration or exclusion of light. Analysis of the reaction mixture by NMR spectroscopy (see Supporting Information section 3.1) revealed an upfield shift in the ^29^Si{^1^H} NMR spectrum from 17.3 (**7**) to 7.8 ppm (**7** + (Et_3_PO)_3_). This resonance is not indicative of retrohydroboration, which would yield the vinyl precursor **9** (δ_Si_ = 2.2). Additionally, vinyl signals were not found in the ^1^H NMR spectrum of **7** + (Et_3_PO)_3_. Addition of Et_3_PO to catalysts **1**, **3**, and **5** results in a color change from yellow to red and no evidence of cluster decomposition by NMR spectroscopy. From these observations, it can be concluded that the Gutmann–Beckett test may not be an appropriate method for systems that contain the [As_7_] clusters, as reaction of Et_3_PO to the As_7_-vinyl precursor **9** showed a similar downfield shift to the ^29^Si resonance to −5.4 ppm (δ_Si_**9** = 2.2) as well as new signals in the ^1^H NMR spectrum, suggesting secondary cluster reactivity in the presence of the phosphine oxide.

Computational investigations into the fluoride ion affinities (FIAs) and hydride ion affinities (HIAs) of **1**–**5**, **7**, and **8** gave values in good agreement with one another (ca. 2 kJ mol^–1^ difference). Table 1 summarizes the first FIA and HIA values, where one fluoride/hydride is added. We previously determined that the addition of two and three fluorides to **1** returns reduced FIA values, presumably due to Coulombic repulsion influences.^27^ This is also observed for the second and third FIA values of **3**, **5**–**7**, and **10** and for the calculated second and third HIAs (see Supporting Information sections 3.2 and 3.3). For chloro tethers **4** and **8**, when attached to a cluster an increase between 5 and 12 kJ mol^–1^ in the ion affinities was observed, whereas for the propyl tether **2**, a reduction of ca. 20 kJ mol^–1^ in both the FIA and the HIA was found upon coordination to the [P_7_] cage. Meanwhile, the FIA and HIA values of catalysts **1** and **7** were found to be very similar to one another, consistent with the cluster being an innocent platform.^27^ In contrast to the Gutmann–Beckett test, when comparing **1** to **5**, where the Lewis acidic components are changed from 9-BBN to BCy_2_, there is an increase in Lewis acidity with an increase in the ion affinities. For completeness, although compounds **6** and **10** could not be prepared in high enough purity that enabled them to be studied as catalysts or in the Gutmann–Beckett test, their the FIA and HIA values were, respectively, computed to be 438 and 465 kJ mol^–1^ for **6** and 344 and 334 kJ mol^–1^ for **10**. Overall, it is worth noting that the computed differences in the FIA and HIA values is rather small between all compounds, and likely within the error of the computational method.

### Hydroboration Catalysis

Although not perfectly correlated, increased Lewis acidity at boron can often lead to greater catalytic competency in hydroboration and hydrosilylation chemistry.^9,34^ As an additional experimental probe of how the Lewis acidity at the boron moieties changes between clusters **1**, **3**, **5**, and **7**, their application in hydroboration catalysis was tested and compared. Diisopropylcarbodiimide (**11a**), phenyl isocyanate (**12a**), acetophenone (**13a**), styrene (**14a**), phenylacetylene (**15a**), and benzonitrile (**16a**) were selected as representative substrates of 6 different unsaturated organic functional groups that have previously been extensively studied in Lewis acid and borane-mediated hydroboration catalysis. These 6 substrates were also selected as they have previously been directly studied with the parent cluster **1**.^27^

To compare the catalytic competency of clusters **3**, **5**, and **7** with the parent cluster **1**, we tested them in the hydroboration of a carbodiimide, isocyanate, ketone, alkene, alkyne, and nitrile. First, the stability of catalysts **3**, **5**, and **7** in C_6_D_6_ was assessed. All catalysts were shown to be stable with no evidence of decomposition by NMR spectroscopy at room temperature (RT) and 50 and 110 °C after 120 h (5 days). Similarly, as determined by high-temperature NMR spectroscopy, clusters **3**, **5**, and **7** were not found to retro-hydroborate even at 100 °C. In order to compare clusters **3**, **5**, and **7** directly with the parent cluster **1**, we used the catalytic conditions previously reported with **1**, specifically (i) a catalyst loading of 5 mol %, (ii) in C_6_D_6_ solvent, (iii) reaction scale and concentration of 0.1 mmol, (iv) pinacolborane (HB(pin)) as the borane reductant, and (v) temperature of either 50 or 110 °C depending on the organic substrate. The sample substrates were selected to be diisopropylcarbodiimide (**11a**), phenyl isocyanate (**12a**), acetophenone (**13a**), styrene (**14a**), phenylacetylene (**15a**), and benzonitrile (**16a**). The newly synthesized chloro tethers **2** and **4** were also tested under identical reaction conditions with a catalyst loading of 15 mol % to maintain identical effective Lewis acid loadings between the chloro tethers and the functionalized clusters. The results from these experiments along with previously reported results for **1** and **8** are summarized in Table 2, and key findings are discussed below.

**Table 2:**
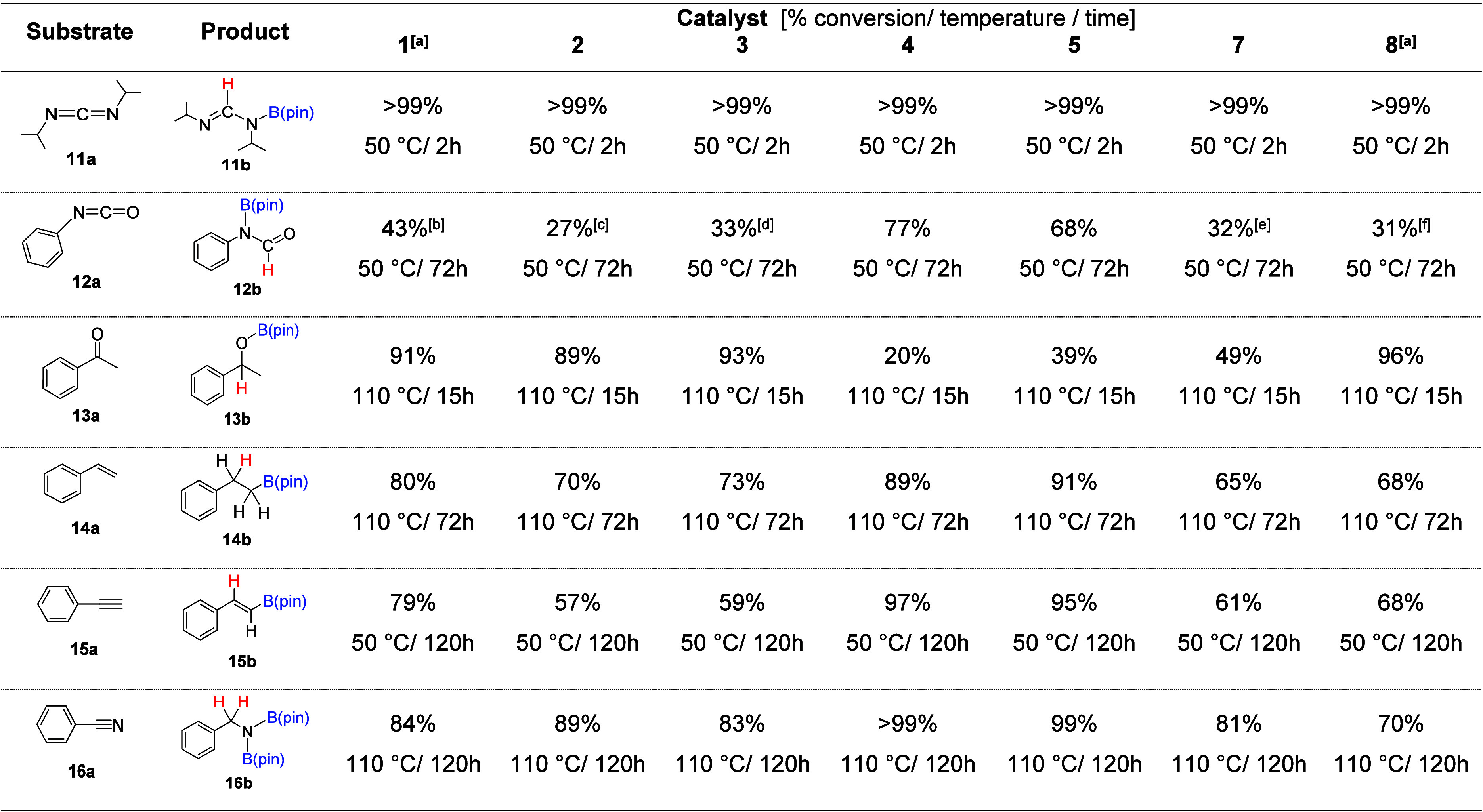
Hydroboration of Diisopropylcarbodiimide (**11a**), Phenyl Isocyanate (**12a**), Acetophenone (**13a**), Styrene (**14a**), Phenylacetylene (**15a**), and Benzonitrile (**16a**) Using Catalysts ({C_8_H_14_}BCH_2_CH_2_SiMe_2_)_3_P_7_ (**1**), ClSiMe_2_CH_2_CH_2_CH_2_B{C_8_H_14_} (**2**), ({C_8_H_14_}BCH_2_CH_2_CH_2_SiMe_2_)_3_P_7_ (**3**), ClSiMe_2_CH_2_CH_2_BCy_2_ (**4**), ({Cy_2_B}CH_2_CH_2_SiMe_2_)_3_P_7_ (**5**), ({C_8_H_14_}BCH_2_CH_2_SiMe_2_)_3_As_7_ (**7**), and ClSiMe_2_CH_2_CH_2_B{C_8_H_14_} (**8**)^g^

a Previously reported.^27^

b Additionally, 8% **12c** and 3% **12d** observed.

c Additionally, 9% **12c** and 2% **12d** observed.

d Additionally, 6% **12c** observed.

e Additionally, 6% **12c** and 1% **12d** observed.

f Additionally, 8% **12c** and 3% **12d** observed

g Catalyst loading = 5 mol % (**1**, **3**, **5**, and **7**), 15 mol % (**2**, **4**, and **8**). Solvent = C_6_D_6_.

To begin, under similar reaction conditions (2 h, 50 °C), when mediating the hydroboration of isopropylcarbodiimide (**11a**) to **11b** there was virtually no difference in conversions observed between catalysts **1**–**5**, **7**, and **8**. However, the hydroboration of phenyl isocyanate (**12a**) with 1 equiv of HB(pin) revealed that the catalysts with {BCy_2_} units, **4** (naked chloro tether at 15 mol % loading) and **5** (cluster mounted system at 5 mol % loading), had greater catalytic competency with conversions > 68% obtained, while the other catalysts gave conversions < 43% under the same reaction conditions. Interestingly, the performance for catalysts with {9-BBN} vs {BCy_2_} Lewis acids appears to reverse in the case of acetophenone (**13a**) hydroboration, where the [P_7_] with {9-BBN} units mounted and corresponding naked tether catalysts **1**–**3** and **8** perform well with conversions > 89% to **13b** obtained, while systems with {BCy_2_} units **4** and **5** underperformed with 20% and 39% conversions observed, respectively. This reaction is also the only instance where the [P_7_]-tethered catalyst **5** outperforms the corresponding chloro tether **4** to an appreciable degree (+19% conversion obtained for catalyst **5**). Generally, a similar performance is observed between the naked chloro tethers and the corresponding cluster-mounted systems, with a ±5% difference in conversions observed. In the case of styrene (**14a**), phenylacetylene (**15b**), and benzonitrile (**16a**) hydroboration, the {BCy_2_}-derived catalysts **4** and **5** perform noticeably better than the {BBN}-derived catalysts (>89% conversion observed in all three substrates, and complete conversion obtained for **16a** hydroboration). Further, addition of the {CH_2_} unit in catalyst **3** compared to **1** resulted in noticeably worse catalytic performance, whereas catalyst **3** gave 10% lower conversion in the hydroboration of styrene (**14a**) and 20% lower conversion in the hydroboration of phenylacetylene (**15a**). In the case of benzonitrile (**16b**) reduction, catalysts **1** and **3** were found to be comparable (84% and 83% respective conversions obtained).

Finally, the [As_7_]-tethered catalyst **7** was tested under ambient light and in the dark, as we had previously found that [As_7_] clusters can demonstrate light sensitivity. The exclusion of light slightly enhanced the catalytic performance, and slightly higher catalytic conversions were observed (ca. 5%) and hence are discussed below. Only in the case of **13a** hydroboration did the ambient light reaction significantly decrease the observed product conversion to 18% from the dark reaction, which gave a conversion of 49%. Comparing the [As_7_] catalyst **7** to its [P_7_] analogue **1**, the most notable performance discrepancy was observed during the hydroboration of **13a**, where catalyst **7** was found to give a conversion of 49% compared to the 91% obtained when catalyst **1** was employed. Product **13b** is thought to coordinate to **7**, evidenced by ^1^H NMR spectroscopy where a small upfield shift of 15% of the **13b** CH quartet from 5.4 to 5.2 ppm was observed. Similarly, an additional resonance could be observed in the ^11^B NMR spectrum. This coordination of the product to catalyst poisons it toward substrate reactivity and is consistent with the lower conversion observed. During the reduction of the other substrates, except diisopropylcarbodiimide (**11a**), catalyst **7** gave lower conversions relative to catalyst **1** by ca. 15%.

Further, in all cases, no monohydroboration of benzonitrile (**16a**) was observed when 1 equiv of HB(pin) was employed, consistent with literature precedence.^36^ Additionally, no bis-hydroboration of diisopropylcarbodiimide (**11a**) or phenylacetylene (**15a**) was observed when an excess of HB(pin) was used and 5 mol % of catalysts **3**, **5**, and **7**, analogous to the reactivity observed with catalyst **1**.

Hydroboration catalysis has previously been extensively studied with s- and p-block Lewis acidic catalysts; relative to many other systems the catalytic performance of compounds **1**–**5**, **7**, and **8** is as expected and modest.^1,37−39^ In addition to the comparisons made above between the tethered and the untethered boranes, comparisons can be made to closely related boranes in similar transformations. Specifically, 9-BBNH has been reported to mediate the hydroboration of carbodiimides in complete conversion at 2.5% catalyst loading at RT,^40^ the hydroboration of alkynes in modest conversion at 5 mol % catalyst loading at RT,^41^ and the bis-hydroboration of nitriles at 10 mol % catalyst loading at 60 °C,^41^ while Cy_2_BH has been reported to facilitate the hydroboration of haloalkynes and alkylalkynes in modest to high conversion at 5 mol % catalyst loading at RT.^41,42^ However, it is worth noting that while 9-BBNH, Cy_2_BH, and even HB(C_6_F_5_)_2_ have been reported in hydroboration catalysis, these secondary boranes are able to access mechanisms where the borane catalyst hydroborates across the unsaturated organic substrate itself and then undergoes a transborylation reaction with the borane reducing agent,^43^ whereas, mechanistically, catalysts **1**–**5**, **7**, and **8** are expected to operate in line with previously reported tricoordinate borane Lewis acids,^37,44^ with activation of the H–B bond of HB(pin), which can then be transferred to the substrate, and subsequent hydride transfer from the catalyst. As no evidence for decomposition or retro-hydroboration of catalysts **3**, **5**, and **7** was observed, secondary borane catalyst mechanisms that invoke initial catalyst hydroboration are not expected to be possible pathways. Coordination of HB(pin) to the catalyst could not be observed by NMR spectroscopy when reacted in a 3:1 ratio with **3**, **5**, and **7**. Additionally, substrates **11a**–**16a** were not found to exhibit any detectable coordination under similar reaction conditions. However, substrate activation that is undetectable by NMR spectroscopy can still promote catalytic activity and would be consistent with the mild catalyst performance observed here. It has previously been reported that when HB(pin) is used as a hydroborating agent, small quantities of BH_3_ can be generated, which is capable of catalyzing hydroboration.^45^ Previously, no evidence for this hidden catalysis was uncovered with **1**.^27^ In an effort to test for this hidden catalysis with **3**, **5**, and **7**, the most forcing conditions were employed, specifically 5 mol % of catalyst, 2 equiv of HB(pin), and **16a** were allowed to react for 120 h at 110 °C. Following reaction completion, an excess of tetramethylethylenediamine (TMEDA) was added to the reaction mixture to capture borohydrides and enable detection by NMR spectroscopy. No evidence of TMEDA-captured borohydrides was detected by ^11^B NMR spectroscopy, consistent with these reactions not being promoted by hidden borohydride catalysis.

## Conclusions

In conclusion, we tuned the structural features of Lewis acids tethered to pnictogen Zintl-derived clusters and quantified how their Lewis acidity and by proxy catalytic competence in hydroboration chemistry were affected. Three structural features were altered: (1) the length of the hydrocarbon linker connecting the Lewis acid and cluster motifs, (2) the borane Lewis acid, and (3) the cluster platform. The Lewis acidity of these systems was quantified experimentally by the Gutmann–Beckett method and by testing hydroboration across six different functional groups and computationally by analysis of the fluoride and hydride ion affinities. We find that the impact of tuning these structural features is more evident when comparing their catalytic competency than by analysis of their Gutmann–Beckett acceptor number and ion affinities alone. We find that tuning the length of the hydrocarbon linker and cluster platform has only a minor impact than when the borane itself is tuned, consistent with reactivity at the borane remaining unperturbed by grafting on the cluster framework. This work evidences that the well-established solution-state chemistry of boranes can be reliably translated using the “silyl aliphatic tether” strategy to polypnictogen structures. Although there have been an increased number of reports where Zintl-based clusters feature as components in catalyst design, the application of these clusters as catalytic tools is still in its infancy. By demonstrating that clusters can act as an innocent platform for a diverse range of anchored Lewis acids without perturbing their reactivity, these tether strategies could be applied to unlock the vault of well-established Lewis acid chemistry to larger and more complex polypnictogen structures, including heterogeneous systems.

## Supporting Information Available

All experimental details, including general procedures, synthetic methods, catalytic procedures, and characterization data (PDF)
