# Supplementary material for: Zintl Clusters as a Platform for Lewis Acid Catalysis
Source: Inorg Chem. 2024 May 30;63(43):20117–25. doi: 10.1021/acs.inorgchem.4c00433 (PMC11523240; doi:10.1021/acs.inorgchem.4c00433)
Supplement: Supplementary file 1 — ic4c00433_si_001.pdf [file ic4c00433_si_001.pdf]

Supporting Information for

Zintl Clusters as a Platform for Lewis Acid Catalysis

Benjamin L. L. Réant,[a] George F. S. Whitehead,[a] Meera Mehta*[b]

[a] Department of Chemistry, University of Manchester, Oxford Road, Manchester, M13 9PL, United Kingdom.

[b] Department of Chemistry, University of Oxford, 12 Mansfield Rd, Oxford OX1 3TA

* meera.mehta@chem.ox.ac.uk

Contents

1. Methods & Materials....S3

1.1. Experimental Considerations....S3

1.2. Analytical Considerations....S3

1.3. Single Crystal X-ray Diffraction Considerations....S4

1.4. Computational Considerations....S5

2. Synthesis....S5

2.1. Synthesis of $ClSiMe_2CH_2CH_2CH_2B\{C_8H_{14}\}$ (2)....S5

2.2. Synthesis of $(\{C_8H_{14}\}BCH_2CH_2CH_2SiMe_2)_3P_7$ (3)....S7

2.3. Synthesis of $ClSiMe_2CH_2CH_2BCy_2$ (4)....S9

2.4. Synthesis of $(\{Cy_2B\}CH_2CH_2SiMe_2)_3P_7$ (5)....S11

2.5. Synthesis of $(\{(C_6F_5)_2B\}CH_2CH_2SiMe_2)_3P_7$ (6)....S13

2.6. Synthesis of $(\{C_8H_{14}\}BCH_2CH_2SiMe_2)_3As_7$ (7)....S15

2.7. Synthesis of $(CH_2CHSiMe_2)_3(As_7)$ (9)....S17

2.8. Synthesis of $(Mes\{(C_8H_{14})BO\}CH)_3P_7$ (10)....S19

3. Lewis Acidity Examination & Solution Stability....S20

3.1. Gutmann-Beckett Testing....S20

3.2. Fluoride Ion Affinity (FIA)....S27

3.3. Hydride Ion Affinity (HIA)....S28

3.4. Energies & Enthalpies of selected Lewis Acids....S30

3.5. HOMO and LUMO Representations....S34

3.6. Solution Stability of Catalysts 3, 5, and 7 .... S38

3.7. Retrohydroboration Examination of Catalysts 3, 5, and 7 .... S42

4. General Procedure for Hydroboration S45

5. Chloro-tether Hydroboration Catalysis Comparison ....S**Error! Bookmark not defined.**

6. Comparative Hydroboration Characterisation Data S46

6.1. Carbodiimide – 11b – N,N'-diisopropyl-N-(4,4,5,5-tetramethyl-1,3,2-dioxaborolan-2- yl)formimidamide.. S46

6.2. Isocyanate – 12b – N-phenyl-N-(4,4,5,5-tetramethyl-1,3,2-dioxaborolan-2-yl)formamide S50

6.3. Ketone – 13b – 4,4,5,5-tetramethyl-2-(1-phenylethoxy)-1,3,2-dioxaborolane S54

6.4. Alkene – 14b – 4,4,5,5-tetramethyl-2-phenethyl-1,3,2-dioxaborolane S58

6.5. Alkyne – 15b – (*E*)-4,4,5,5-tetramethyl-2-styryl-1,3,2-dioxaborolane S62

6.6. Nitrile – 16b – N-benzyl-4,4,5,5-tetramethyl-N-(4,4,5,5-tetramethyl-1,3,2-dioxaborolan-2-yl)-1,3,2-dioxaborolan-2-amine S66

7. Studies into Catalyst–Substrate Interactions S70

8. Testing for Hidden Catalysis S70

9. Crystallographic Data S72

10. Geometry Optimized Structures S73

11. References S230

1. Methods & Materials

1.1. Experimental Considerations

All manipulations were performed under an inert atmosphere using standard Schlenk-line, and glovebox techniques. Glassware was flame dried prior to use.

Dry THF, Et_2O, toluene, and pentane were obtained using Innovative Technologies anhydrous engineering solvent purification systems and subsequently degassed. DME was dried over Na and purified by distillation. C_6D_6, and toluene-d_8, were dried over activated 3 Å molecular sieves. All solvents were stored over activated 3 Å molecular sieves.

Elemental phosphorus (Merck), Naphthalene (Fluorochem), chloro(dimethyl)vinylsilane ($ClSiMe_2CHCH_2$, Merck), chloro(phenylmethyl)vinylsilane ($ClSiMePhCHCH_2$, Merck), chloro(diphenyl)vinylsilane ($ClSiPh_2CHCH_2$, Merck), chloro(dimethyl)allylsilane ($ClSiMe_2CH_2CHCH_2$, Merck), 9-Borabicyclo[3.3.1]nonane dimer (9-BBN dimer, Merck) and all organic substrates (**11a** to **15a**) used in this study were purchased from commercial sources and used without any further purification. $[Na(DME)_x]_3P_7$,[1] $(CH_2CHSi)_3P_7$,[2] $HBCy_2$,[3] and $(MesCO)_3(P_7)$,[4] were synthesized according to previously reported synthetic procedures.

1.2. Analytical Considerations

NMR Spectroscopy. ^1H, ^{11}B, $^{11}B\{^1H\}$, $^{13}C\{^1H\}$, ^{19}F, ^{29}Si DEPT90, ^{31}P NMR spectra were recorded on a Bruker AVIII 400 spectrometer (operating frequencies: 399.78 MHz, 128.36 MHz, 100.53 MHz, 376.17 MHz, 79.48 MHz and 161.83 MHz for ^1H,^{11}B ^{13}C, ^{19}F, ^{29}Si and ^{31}P, respectively). ^1H and $^{13}C\{^1H\}$ NMR chemical shifts were internally referenced to the residual solvent resonances (C_6D_6 (benzene-d_6): ^1H δ = 7.16 ppm, $^{13}C\{^1H\}$ δ = 128.02 ppm, C_7D_8 (toluene-d_8): ^1H δ = 7.09, 7.00, 6.98, 2.09 ppm, $^{13}C\{^1H\}$ δ = 137.86, 129.24, 128.33, 125.49, 20.4 ppm). ^{11}B, ^{19}F, ^{29}Si, ^{31}P chemical shifts were externally referenced to $BF_3{\cdot}Et_2O$, $CFCl_3$, Me_4Si, H_3PO_4, respectively. NMR samples were prepared under an inert atmosphere in 5 mm J Young NMR tubes. Data was analyzed using MestReNova V14.0.0 software.

Elemental Analysis. Elemental analysis was carried out by Mr. Martin Jennings and Mrs. Anne Davies at the Microanalytical Service, School of Chemistry, the University of Manchester using a Flash 2000 elemental analyzer. The elemental analysis carbon value found for the synthesis of compound **7** was larger than expected, and for compound **9** was lower than expected, despite multiple attempts at obtaining better data. Best data is reported, and NMR spectroscopy and mass spectrometry data are consistent with product formulation. It is noteworthy, that in a study carried out by Melen and co-workers evaluating elemental analysis found that random error can variably lead to differences outside of 0.4%.[5] Elemental analysis was not conducted on compounds **2**, **4** and **5** as these compounds were isolated as oils which caused issues in accurate weighing when crimping the sample capsule close.

Mass spectrometry. Mass spectrometry samples were measured by the Mass Spectrometry Service, School of Chemistry, the University of Manchester using an electrospray ionization or atmospheric pressure chemical ionization equipped Thermo Orbitrap Executive Plus Extended Mass Range mass spectrometer. Samples were prepared under a nitrogen atmosphere and injected into the ionization source of the mass spectrometer. Due to the sensitivity of compound **6**, mass spectrometry was not possible despite multiple efforts. Despite multiple attempts, we were unable to characterize compound **13b** by mass spectrometry.

1.3. Single Crystal X-ray Diffraction Considerations

Data collection: X-ray diffraction data for compounds **3**, **9**, and **10** were collected using a dual wavelength Rigaku FR-X rotating anode diffractometer using CuKα (λ = 1.54146 Å) radiation, equipped with an AFC-11 4-circle kappa goniometer, VariMAX^{TM} microfocus optics, a Hypix-6000HE detector and an Oxford Cryosystems 800 plus nitrogen flow gas system, at a temperature of 100K. Data were collected and reduced using CrysAlisPro v42. Absorption correction was performed using empirical methods (SCALE3 ABSPACK) based upon symmetry-equivalent reflections combined with measurements at different azimuthal angles.

Crystal structure determination: The crystal structure was solved and refined against all F2 values using the SHELX and Olex2 suite of programmes.[6] All atoms

were refined anisotropically. Hydrogen atoms were placed in calculated positions and refined using idealized geometries and assigned fixed isotropic displacement parameters.

Crystallographic data have been deposited with the CCDC (CCDC 2328547 (**3**), 2328549 (**9**) and 2328550 (**10**)).

1.4. Computational Considerations

Density Functional Theory (DFT) calculations were performed with the Gaussian09 program package3 (version g09, rev.d01).[7] Geometry optimizations and frequency calculations were conducted at the BP86/SV(p) level of theory. No symmetry constraints were applied during optimization. All minima were confirmed by the absence of imaginary frequencies. Initial geometries were prepared using X-ray diffraction coordinates where possible and Facio V22.1.1.64 software. Gibbs free reaction energies and enthalpies were calculated for standard conditions (p = 1 atm, T = 298 K) and are unscaled. HOMO and LUMO images were prepared using Avogadro V1.2.0 software.

2. Synthesis

2.1. Synthesis of $ClSiMe_2CH_2CH_2CH_2B\{C_8H_{14}\}$ (2)

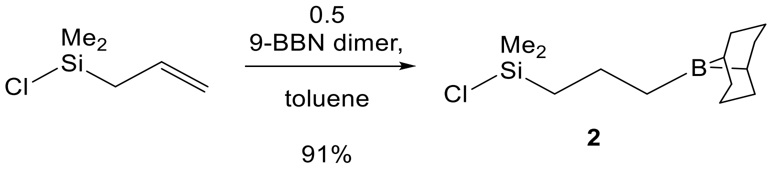

Scheme S1. Synthetic route to $ClSiMe_2CH_2CH_2CH_2B\{C_8H_{14}\}$ (**2**).

To a Schlenk flask charged with a stir bar, 9-BBN dimer (1.614 g, 6.6 mmol) was partially dissolved in toluene (10 mL). A toluene (5 mL) solution of $ClSiMe_2CH_2CHCH_2$ (1.782 g, 13.2 mmol) was then added dropwise at which all remaining borane dissolved. The reaction was stirred at room temperature for three days and then all volatiles removed under reduced pressure giving $ClSiMe_2CH_2CH_2CH_2B\{C_8H_{14}\}$ (**2**) as a colorless oil.

Isolated Yield: 3.089 g, 12.03 mmol, 91 %.

^{1}H NMR (400 MHz, 298 K, C_6D_6): δ = 1.78–1.89 (m, 6H, BBN-CH_2), 1.72–1.74 (s, br, 2H, CH_2), 1.61–1.70 (m, 6H, BBN-CH_2), 1.43 (t, $^1J_{HH}$ = 8.2 Hz, 2H, CH_2), 1.15–1.23 (m, 2H, BBN-CH), 0.78 (t, $^1J_{HH}$ = 8.2 Hz, 2H, CH_2), 0.27 (s, 6H, SiCH_3) ppm.

^{11}B NMR (128 MHz, 298 K, C_6D_6): δ = 88.10 (s) ppm.

^{13}C{^{1}H} NMR (101 MHz, 298 K, C_6D_6): δ = 33.51 (s, BBN-*C*H_2), 32.25 (s, *C*H_2), 31.40 (s, BBN-*C*H), 23.66 (s, BBN-*C*H_2), 22.85 (s, *C*H_2), 18.55 (s, *C*H_2), 1.79 (s, Si*C*H_3) ppm.

^{29}Si{^{1}H} NMR (79 MHz, 298 K, C_6D_6): δ = 30.58 (s) ppm.

Mass spectrometry (APCI): $C_{13}H_{26}BSiCl$+Na ([M+Na]$^+$); Calcd. = 279.1483, Found = 279.1423.

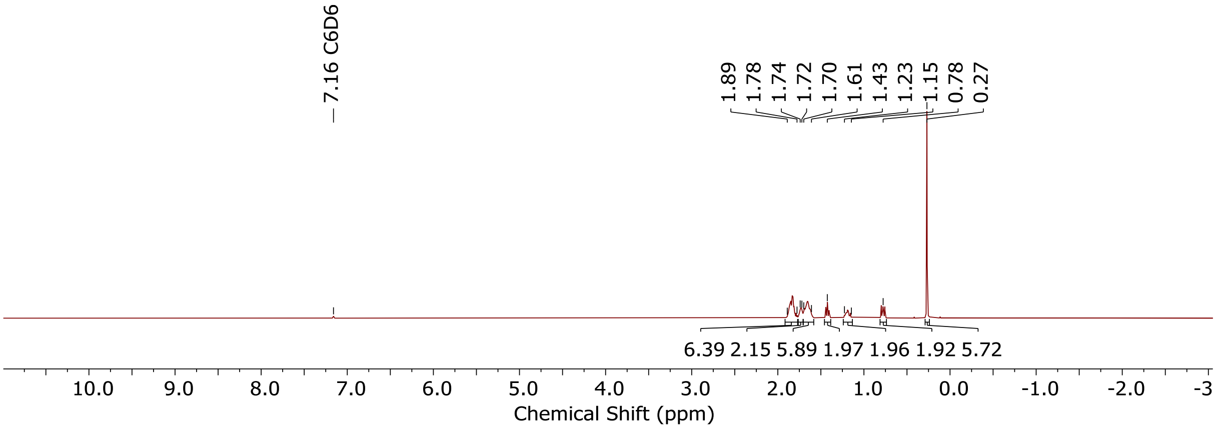

Figure S1. ^{1}H NMR spectrum (C_6D_6) of **2**.

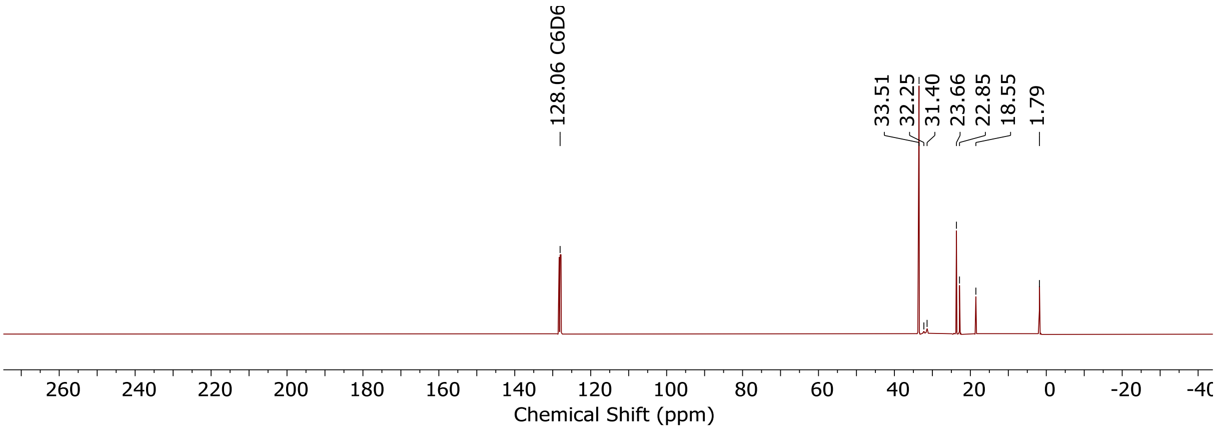

Figure S2. ^{13}C{^{1}H} NMR spectrum (C_6D_6) of **2**.

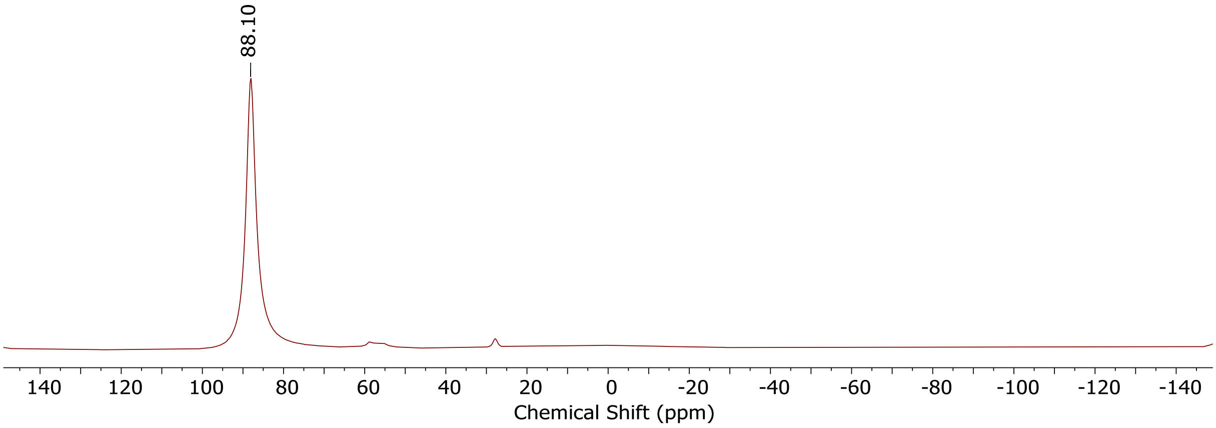

Figure S3. ^{11}B NMR spectrum (C_6D_6) of **2**.

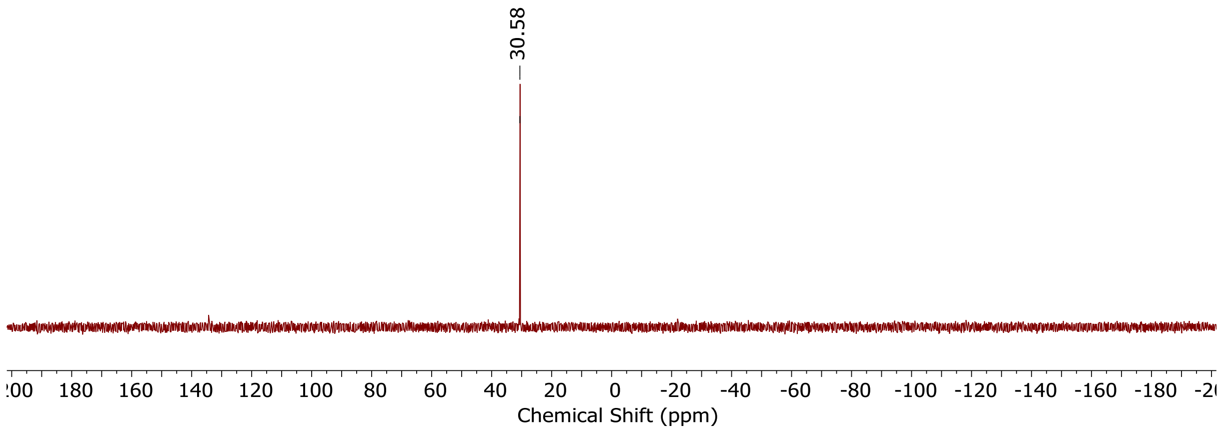

Figure S4. $^{29}Si\{^1H\}$ NMR spectrum (C_6D_6) of **2**.

2.2. Synthesis of ($\{C_8H_{14}\}BCH_2CH_2CH_2SiMe_2)_3P_7$ (3)

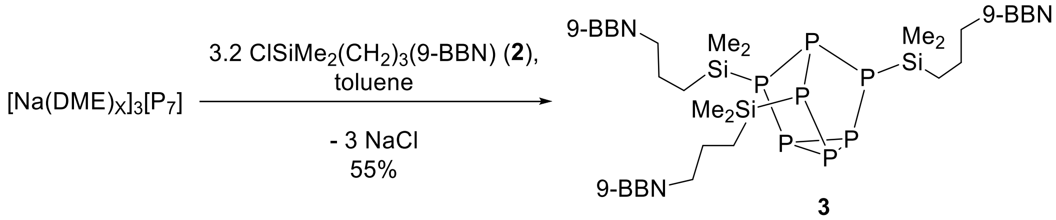

Scheme S2. Synthesis of ($\{C_8H_{14}\}BCH_2CH_2CH_2SiMe_2)_3P_7$ (**3**).

A Schlenk flask was charged with a stir bar, $[Na(DME)]_3[P_7]$ (0.500 g, ~1 mmol) and toluene (15 mL). $ClSiMe_2CH_2CH_2CH_2B\{C_8H_{14}\}$ (**2**, 0.834 g, 3.25 mmol) in toluene (5 mL) was added dropwise at room temperature and the suspension stirred for five days. The suspension was settled and filtered yielding a clear yellow solution. Volatiles were removed under reduced pressure and the residue was washed with pentane (8 mL) yielding ($\{C_8H_{14}\}BCH_2CH_2CH_2SiMe_2)_3P_7$ (**3**) as a beige solid. Crystals suitable for single crystal XRD analysis were obtained from a concentrated pentane solution at –35 °C after several days.

Isolated Yield: 0.493 g, 0.56 mmol, 55 %.

^{1}H NMR (400 MHz, 298 K, C_6D_6): δ = 1.94–1.68 (m, 42H, BBN-CH_2 & CH_2), 1.49 (t, $^1J_{HH}$ = 7.3 Hz, 6H, CH_2), 1.20–1.29 (m, 6H, BBN-C*H*), 0.89 (s, br, 6H, CH_2), 0.33 (s, 18H, SiCH_3) ppm.

^{11}B NMR (128 MHz, 298 K, C_6D_6): δ = 88.45 (s) ppm.

$^{13}C\{^1H\}$ NMR (101 MHz, 298 K, C_6D_6): δ = 33.57 (s, BBN-CH_2), 32.62 (s, CH_2), 31.42 (s, BBN-*C*H), 23.69 (s, BBN-CH_2), 20.00 (s, CH_2), 14.33 (s, CH_2), 1.97 (s, SiCH_3) ppm.

$^{29}Si\{^1H\}$ NMR (79 MHz, 298 K, C_6D_6): δ = 8.73 (d, $^1J_{SiP}$ = 47.5 Hz) ppm.

^{31}P NMR (162 MHz, 298 K, C_6D_6): δ = –155.35 – –159.86 (m, 3P, *basal*), –97.37 – –104.21 (qq, $^{1}J_{PP}$ = 322.6 Hz, $^{2}J_{PP}$ = 46.2 Hz, 1P, *apical*), 0.38 – –6.06 (m, 3P, *linking*) ppm.

Elemental analysis: Expected/Found: C = 53.20/53.22; H = 8.93/9.05; N = 0/0.

Mass spectrometry (APCI): $C_{39}H_{78}B_3P_7Si_3$+H ([M+H]$^+$); Calcd. = 881.3932, Found = 881.3973.

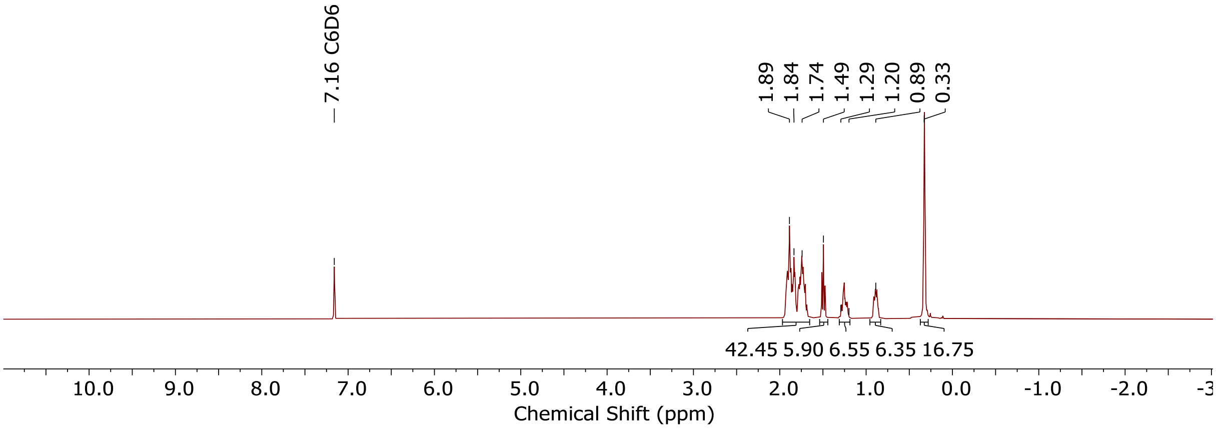

Figure S5. ^{1}H NMR spectrum (C_6D_6) of **3**.

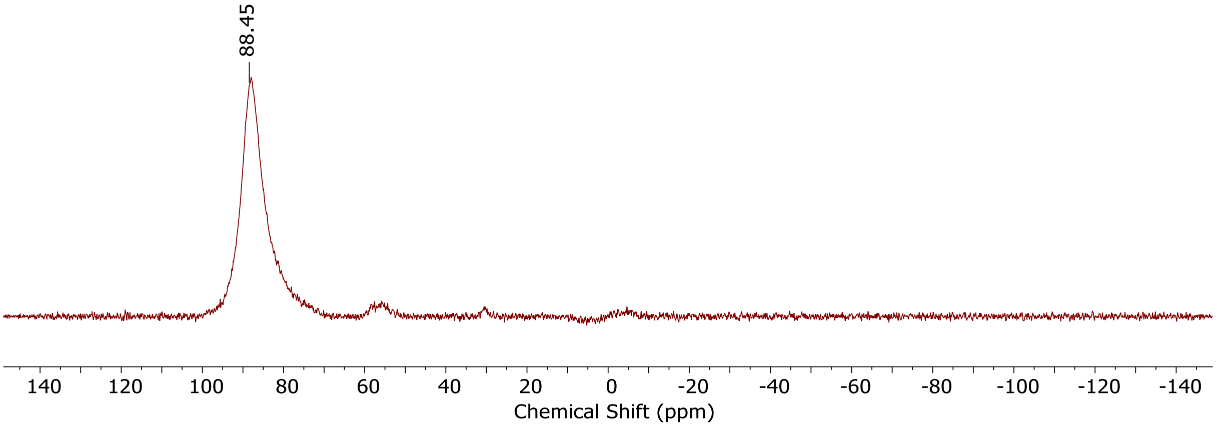

Figure S6. ^{11}B NMR spectrum (C_6D_6) of **3**.

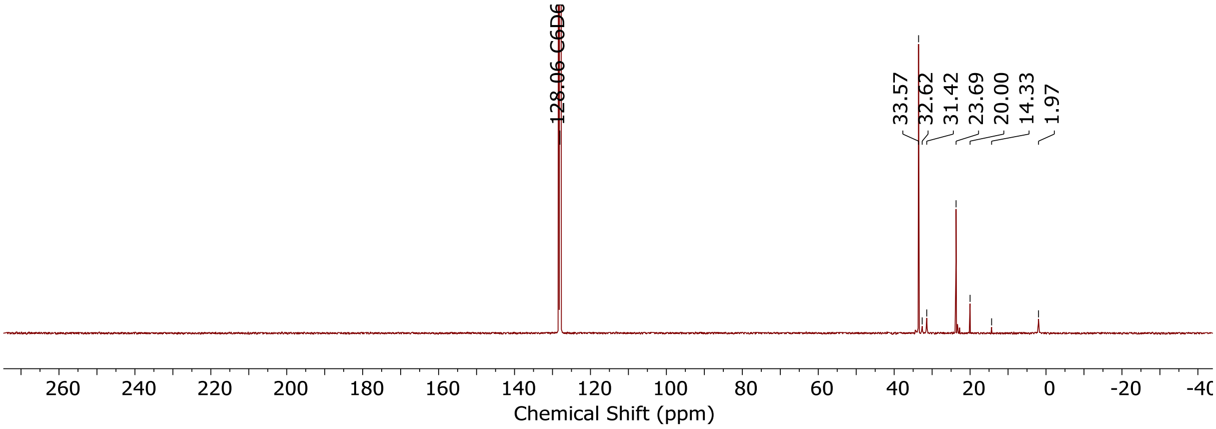

Figure S7. ^{13}C{^{1}H} NMR spectrum (C_6D_6) of **3**.

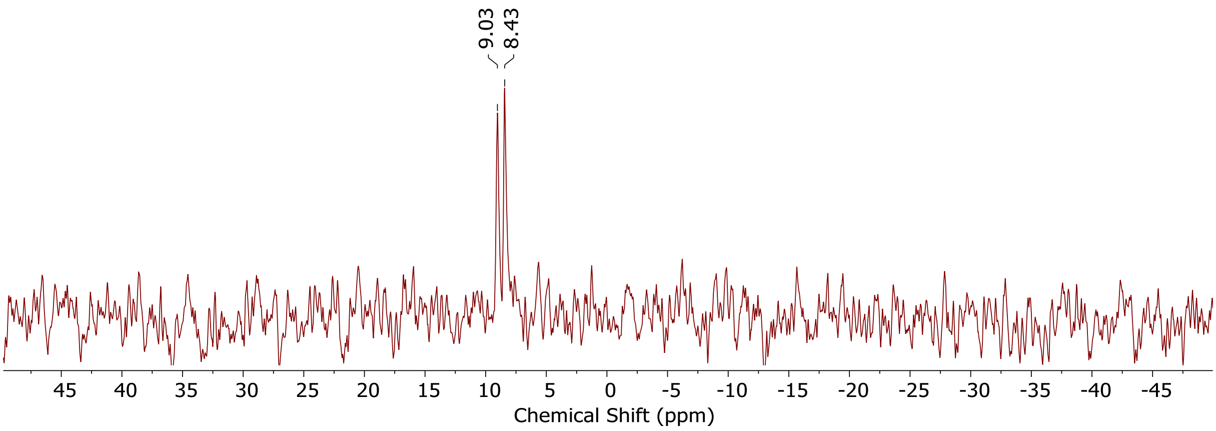

Figure S8. $^{29}Si\{^1H\}$ NMR spectrum (C_6D_6) of **3**.

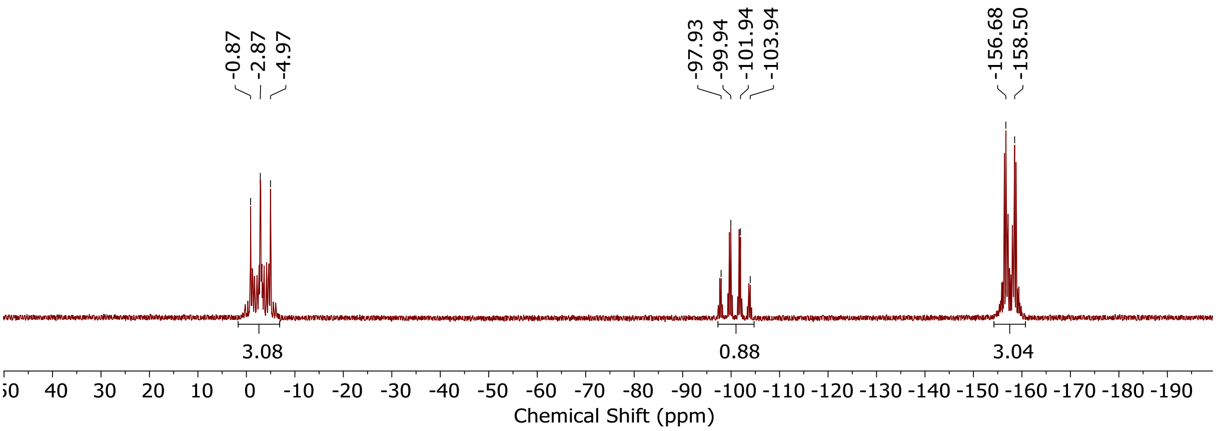

Figure S9. ^{31}P NMR spectrum (C_6D_6) of **3**.

2.3. Synthesis of $ClSiMe_2CH_2CH_2BCy_2$ (4)

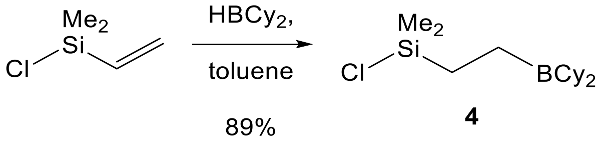

4

Scheme S3. Synthetic route to $ClSiMe_2CH_2CH_2BCy_2$ (**4**).

To a Schlenk flask charged with a stir bar, $HBCy_2$ (2.529 g, 14.5 mmol) was partially dissolved in toluene (15 mL). A toluene (5 mL) solution of $ClSiMe_2CHCH_2$ (1.748 g, 14.5 mmol) was then added dropwise at which point all remaining borane dissolved. The reaction was stirred at room temperature for three days and then all volatiles were removed under reduced pressure giving $ClSiMe_2CH_2CH_2BCy_2$ (**4**) as a colorless oil.

Isolated Yield: 3.864 g, 12.93 mmol, 89 %.

^{1}H NMR (400 MHz, 298 K, C_6D_6): δ = 1.80–1.73 (m, 6H, Cy-CH_2), 1.56–1.59 (m, 4H, Cy-CH_2), 1.41 (tt, $^1J_{HH}$ = 12.2 Hz, $^4J_{HH}$ = 2.7 Hz, 2H, Cy-C*H*), 1.33–1.11 (m, 12H, Cy-CH_2, CH_2), 0.63 (t, $^1J_{HH}$ = 12.0 Hz, 2H, CH_2), 0.29 (s, 6H, SiCH_3) ppm.

^{11}B NMR (128 MHz, 298 K, C_6D_6): δ = 82.13 (s) ppm.

$^{13}C\{^1H\}$ NMR (101 MHz, 298 K, C_6D_6): δ = 35.95 (s, Cy-*C*H), 28.02 (s, Cy-*C*H_2), 27.76 (s, Cy-*C*H_2), 27.43 (s, *C*H_2), 15.53 (s, Cy-*C*H_2), 11.00 (s, *C*H_2), 1.13 (s, Si*C*H_3) ppm.

$^{29}Si\{^1H\}$ NMR (79 MHz, 298 K, C_6D_6): δ = 32.65 (s) ppm.

Mass spectrometry (APCI): $C_{16}H_{32}BSiCl$+Cl ($[M+Cl]^-$); Calcd. = 333.1749, Found = 333.1745.

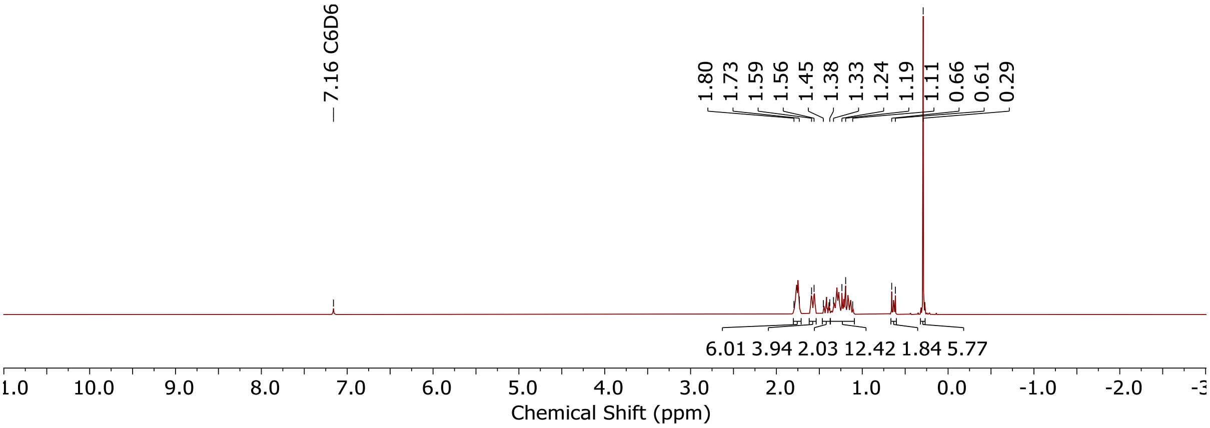

Figure S10. ^{1}H NMR spectrum (C_6D_6) of **4**.

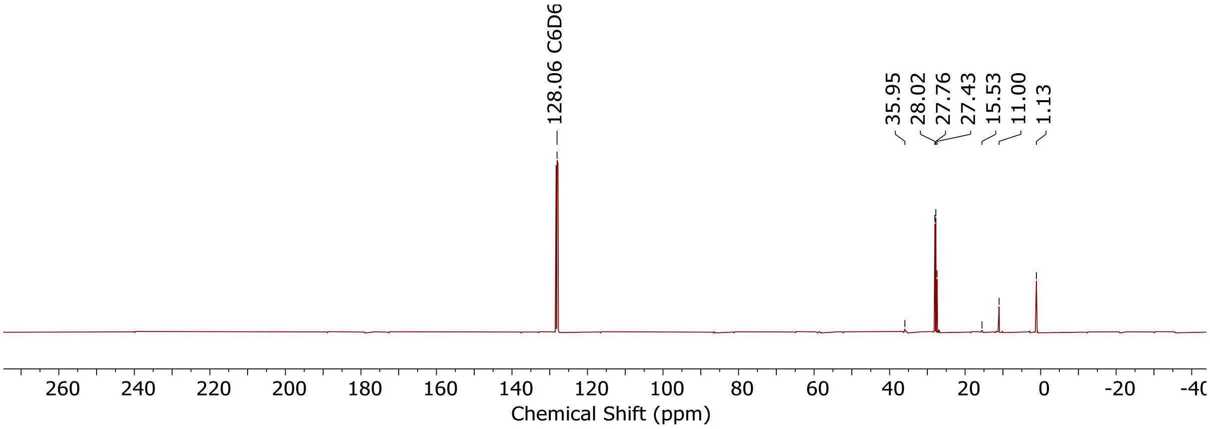

Figure S11. $^{13}C\{^1H\}$ NMR spectrum (C_6D_6) of **4**.

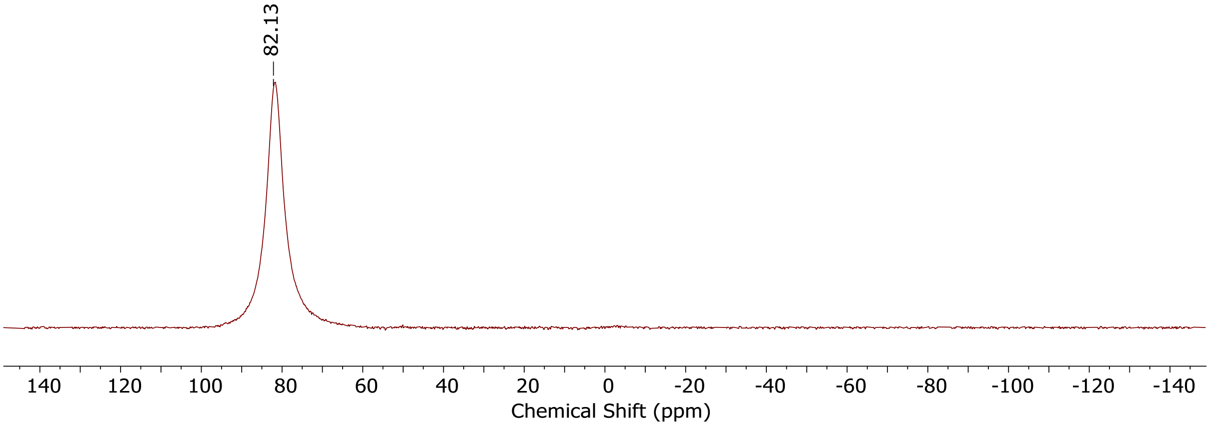

Figure S12. ^{11}B NMR spectrum (C_6D_6) of **4**.

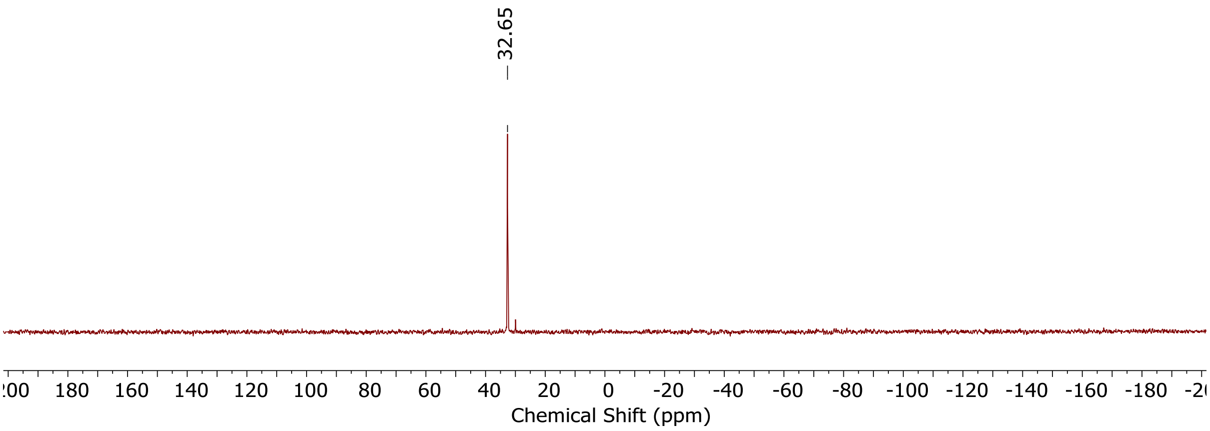

Figure S13. ^{29}Si{^{1}H} NMR spectrum (C_6D_6) of **4**.

2.4. Synthesis of ({Cy_2B}$CH_2CH_2SiMe_2$)$_3P_7$ (5)

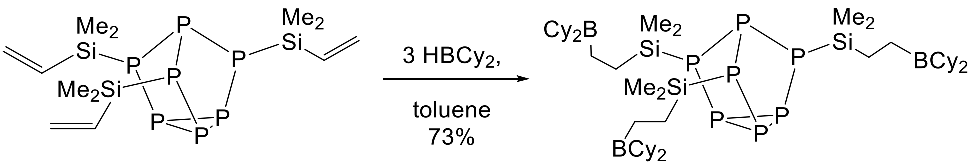

5

Scheme S4. Synthesis of ({Cy_2B}$CH_2CH_2SiMe_2$)$_3P_7$ (**5**).

To a Schlenk flask charged with a stir bar, ($CH_2CHSiMe_2$)$_3P_7$ (0.500 g, 1.06 mmol), $HBCy_2$ (0.566 g, 3.18 mmol) and toluene (10 mL) were added at room temperature. The suspension was stirred for three days, volatiles were removed under reduced pressure and the resulting yellow oil was dried for a further five hours yielding ({Cy_2B}$CH_2CH_2SiMe_2$)$_3P_7$ (**5**).

Isolated Yield: 0.780 g, 0.77 mmol, 73 %.

^{1}H NMR (400 MHz, 298 K, C_6D_6): δ = 1.83–1.77 (m, 18H, Cy-C*H*$_2$), 1.68–1.64 (m, 12H, Cy-C*H*$_2$), 1.48–1.18 (m, 42H, Cy-C*H*, Cy-C*H*$_2$, C*H*$_2$), 0.71–0.67 (m, 6H, C*H*$_2$), 0.36 (s, 18H, SiC*H*$_3$) ppm.

^{11}B NMR (128 MHz, 298 K, C_6D_6): δ = 81.46 (s) ppm.

^{13}C{^{1}H} NMR (101 MHz, 298 K, C_6D_6): δ = 36.08 (s, Cy-*C*H), 28.14 & 28.03 (s, Cy-*C*H$_2$), 27.72 & 27.53 (s, Cy-*C*H$_2$), 26.91 (s, *C*H$_2$), 17.12 (s, Cy-*C*H$_2$), 11.10 (s, *C*H$_2$), 1.59 (s, Si*C*H$_3$) ppm.

^{29}Si{^{1}H} NMR (79 MHz, 298 K, C_6D_6): δ = 12.20 (d, $^1J_{SiP}$ = 46.3 Hz) ppm.

^{31}P NMR (162 MHz, 298 K, C_6D_6): δ = –155.22 – –157.60 (m, 3P, *basal*), –96.70 – –103.55 (qq, $^1J_{PP}$ = 325.7 Hz, $^2J_{PP}$ = 46.8 Hz, 1P, *apical*), –1.19 – –5.29 (m, 3P, *linking*) ppm.

Mass spectrometry (APCI): $C_{48}H_{96}B_3P_7Si_3$+H ([M+H]$^+$); Calcd. = 1007.5335, Found = 1007.5380.

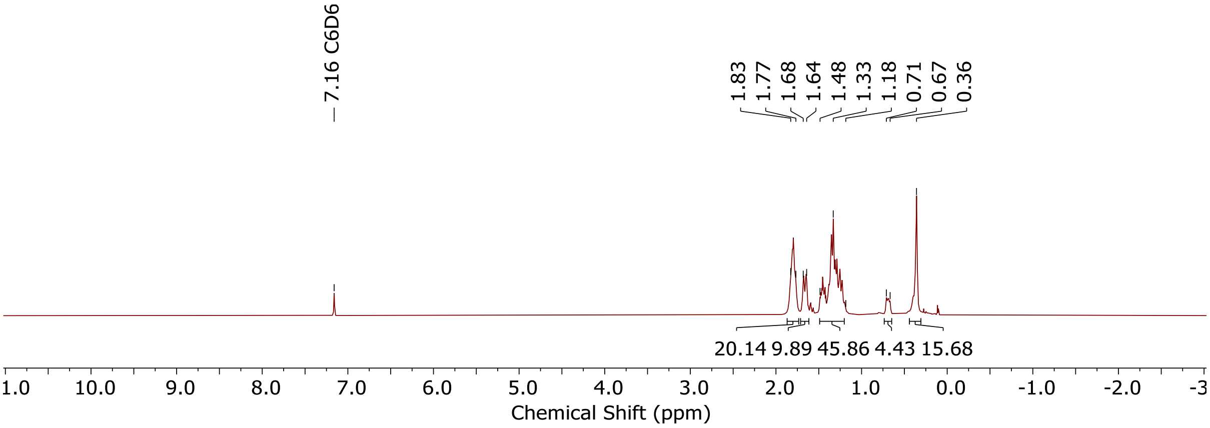

Figure S14. ^{1}H NMR spectrum (C_6D_6) of **5**.

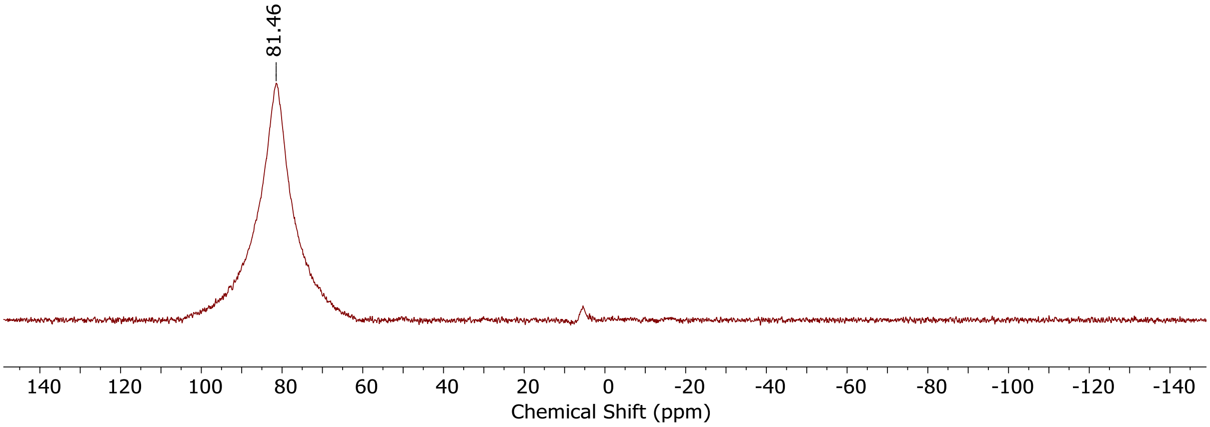

Figure S15. ^{11}B NMR spectrum (C_6D_6) of **5**.

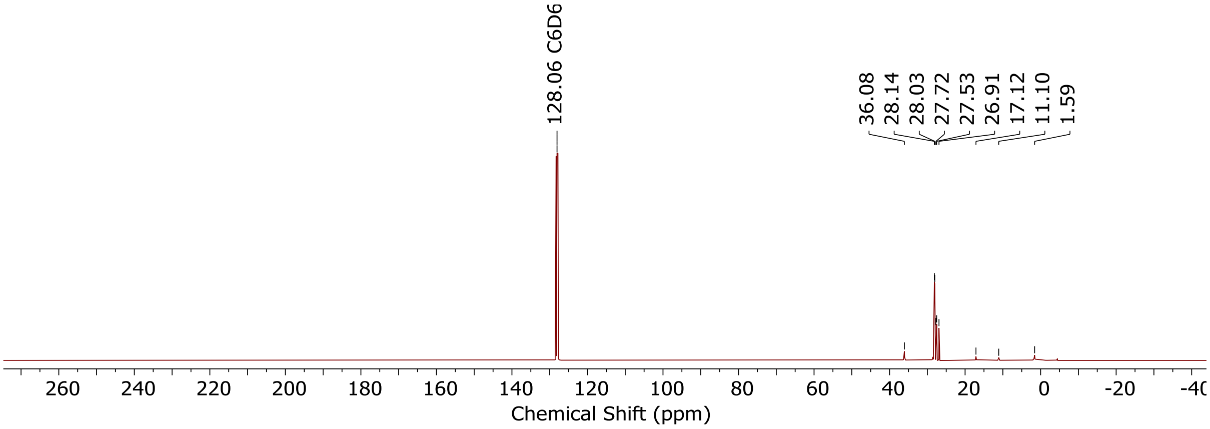

Figure S16. ^{13}C{^{1}H} NMR spectrum (C_6D_6) of **5**.

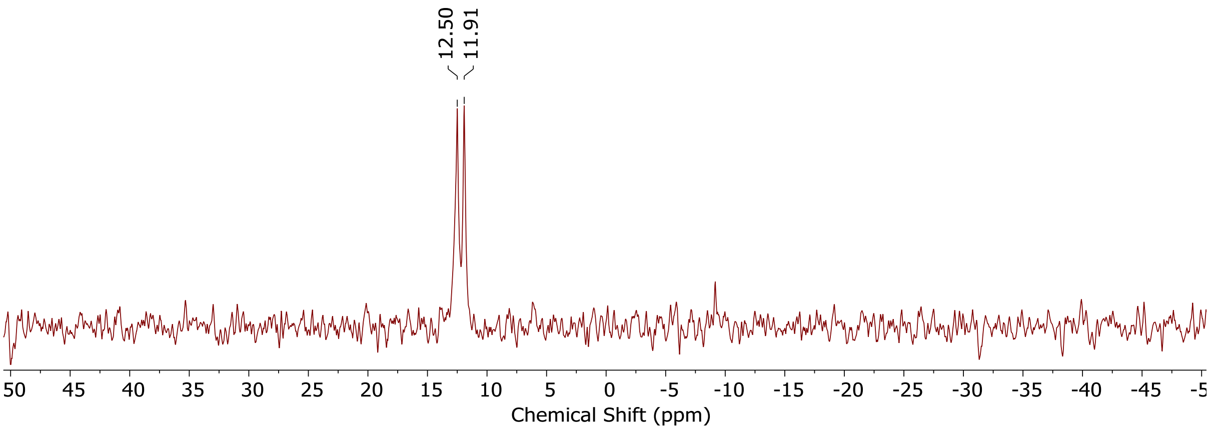

Figure S17. $^{29}Si\{^{1}H\}$ NMR spectrum (C_6D_6) of **5**.

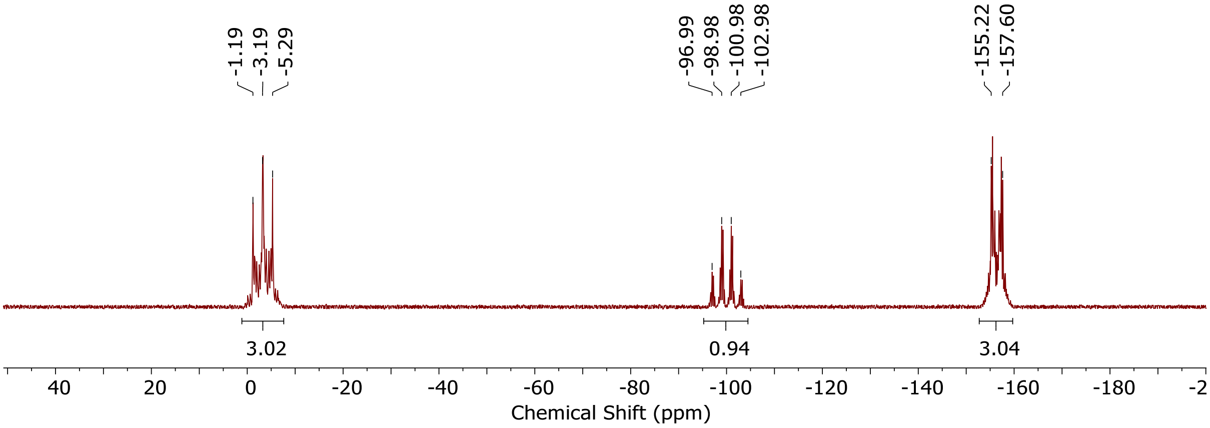

Figure S18. ^{31}P NMR spectrum (C_6D_6) of **5**.

2.5. Synthesis of ($\{(C_6F_5)_2B\}CH_2CH_2SiMe_2)_3P_7$ (6)

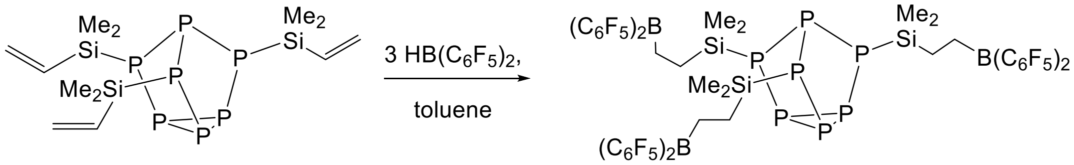

6

Scheme S5. Synthesis of ($\{(C_6F_5)_2B\}CH_2CH_2SiMe_2)_3P_7$ (**6**).

A Schlenk flask was charged with a stir bar, $(CH_2CHSiMe_2)_3P_7$ (1.000 g, 2.12 mmol), and $HB(C_6F_5)_2$ (2.197 g, 6.35 mmol) was cooled to –78 °C. Toluene (15 mL) was added, and the reaction mixture stirred at –78 °C for 10 minutes before warming to room temperature. The yellow solution was stirred for 18 hours, and then all volatiles removed under reduced pressure yielding ($\{(C_6F_5)_2B\}CH_2CH_2SiMe_2)_3P_7$ (**6**) as a yellow solid.

^{1}H NMR (400 MHz, 298 K, C_6D_6): δ = 1.90 (s, br, 6H, CH_2), 0.72 (s, br, 6H, CH_2), 0.24 (s, 18H, SiCH_3) ppm.

$^{19}F\{^{1}H\}$ NMR (376 MHz, 298 K, C_6D_6): δ = –129.15 (m, *ortho-F*), –149.32 (m, *para-F*), –161.23 (m, *meta-F*).

$^{29}Si\{^{1}H\}$ NMR (79 MHz, 298 K, C_6D_6): δ = 15.59 (d, $^{1}J_{SiP}$ = 47.2 Hz) ppm.

^{31}P NMR (162 MHz, 298 K, C_6D_6): δ = –128.54 – –134.63 (m, 3P, *basal*), –73.04 – –79.88 (qq, $^{1}J_{PP}$ = 335.9 Hz, $^{2}J_{PP}$ = 38.3 Hz, 1P, *apical*), 19.05 – –25.68 (m, 3P, *linking*) ppm.

Elemental analysis: Expected/Found for **6**•toluene: C = 41.23/40.79; H = 2.39/2.53; N = 0/0.

Mass spectrometry (APCI): *Could not be obtained despite multiple efforts.*

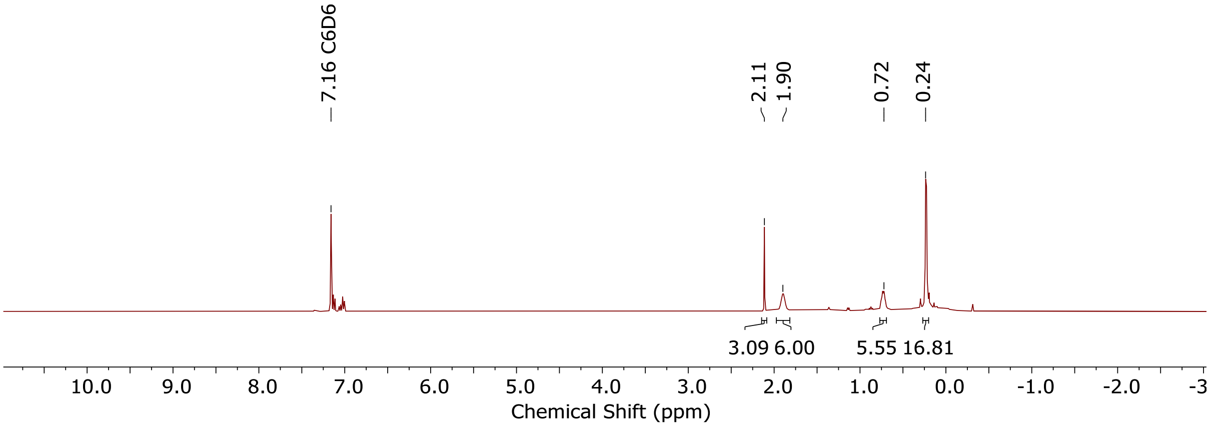

Figure S19. ^{1}H NMR spectrum (C_6D_6) of **6**. PhCH_3 region integrated to show one molecule of toluene per molecule of compound **6**.

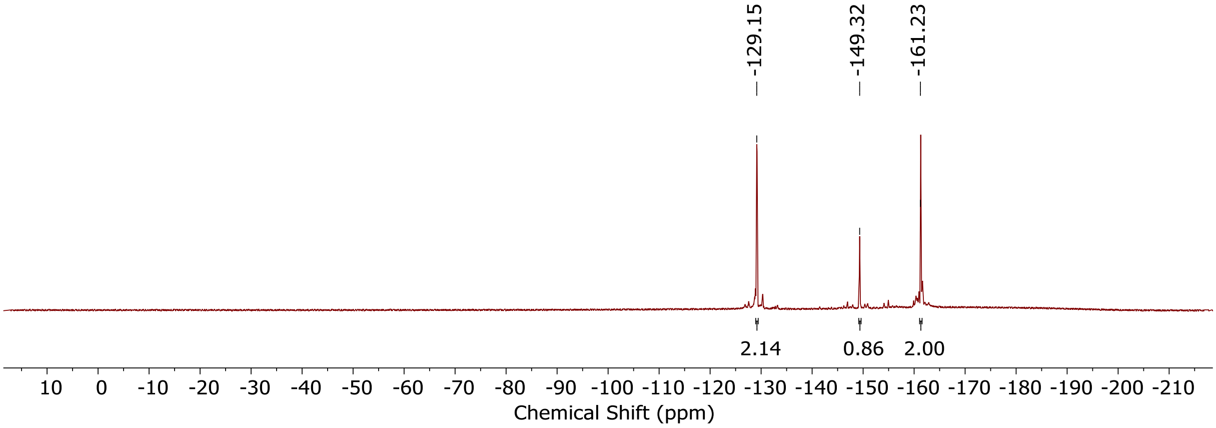

Figure S20. $^{19}F\{^{1}H\}$ NMR spectrum (C_6D_6) of **6**.

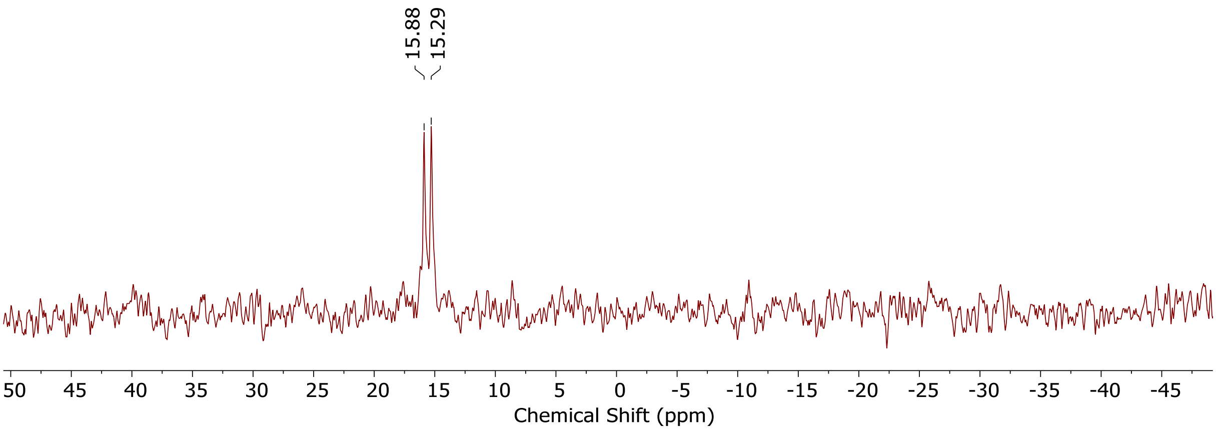

Figure S21. $^{29}Si\{^1H\}$ NMR spectrum (C_6D_6) of **6**.

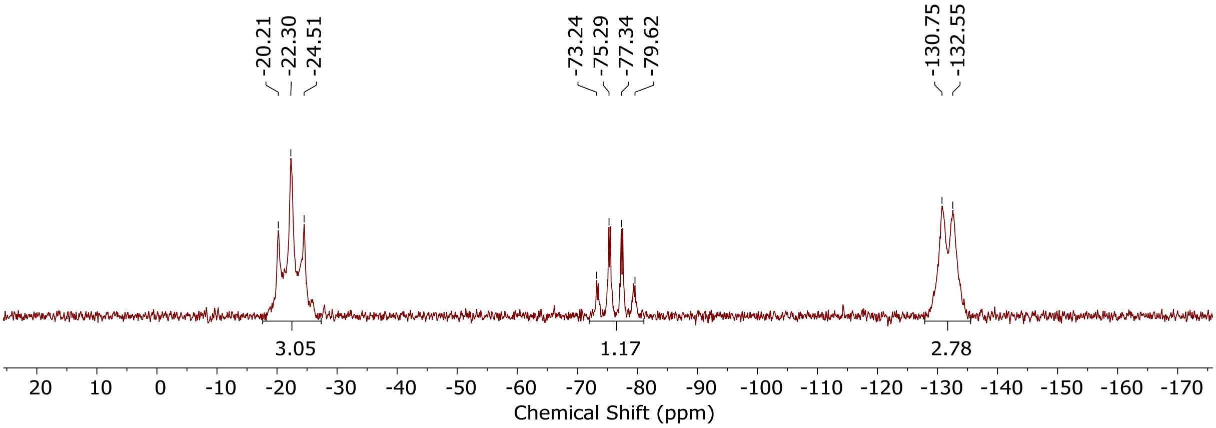

Figure S22. ^{31}P NMR spectrum (C_6D_6) of **6**.

2.6. Synthesis of ($\{C_8H_{14}\}BCH_2CH_2SiMe_2)_3As_7$ (7)

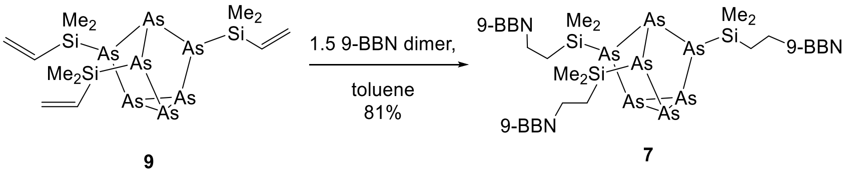

Scheme S6. Synthesis of ($\{C_8H_{14}\}BCH_2CH_2SiMe_2)_3As_7$ (**7**).

To a Schlenk flask charged with a stir bar, $(CH_2CHSiMe_2)_3(As_7)$ (**9**, 0.300 g, 0.39 mmol), 9-BBN dimer (0.141 g, 0.58 mmol) and toluene (5 mL) were added at room temperature. The suspension was stirred for three days, and then all volatiles were removed under reduced pressure and the residue washed with Et_2O (5 mL). The resulting pale red powder was dried under reduced pressure for five hours yielding ($\{C_8H_{14}\}BCH_2CH_2SiMe_2)_3As_7$ (**7**).

Isolated Yield: 0.335 mg, 0.29 mmol, 81%

^{1}H NMR (400 MHz, 298 K, C_6D_6): δ = 1.70–1.92 (m, 36H, BBN-C*H*$_2$), 1.47–1.51 (m, 6H, C*H*$_2$), 1.20–1.29 (m, 6H, BBN-C*H*), 0.97–1.01 (m, 6H, C*H*$_2$), 0.48 (s, 9H, SiC*H*$_3$), 0.45 (s, 9H, SiC*H*$_3$) ppm.

^{11}B NMR (128 MHz, 298 K, C_6D_6): δ = 86.65 (s) ppm.

^{13}C{^{1}H} NMR (101 MHz, 298 K, C_6D_6): δ = 33.73 (s, BBN-CH_2), 31.56 (s, BBN-CH), 23.71 (s, BBN-CH_2), 21.68 (s, CH_2), 13.42 (s, CH_2), 3.27 (s, SiCH_3), 2.94 (s, SiCH_3) ppm.

^{29}Si{^{1}H} NMR (79 MHz, 298 K, C_6D_6): δ = 17.32 (s) ppm.

Elemental analysis: Expected/Found: C = 37.73/38.52; H = 6.33/6.64; N = 0/0.

Mass spectrometry (APCI): $C_{36}H_{72}As_7B_3Si_3$+Na ([M+Na]$^+$); Calcd. = 1168.9630, Found = 1168.9650.

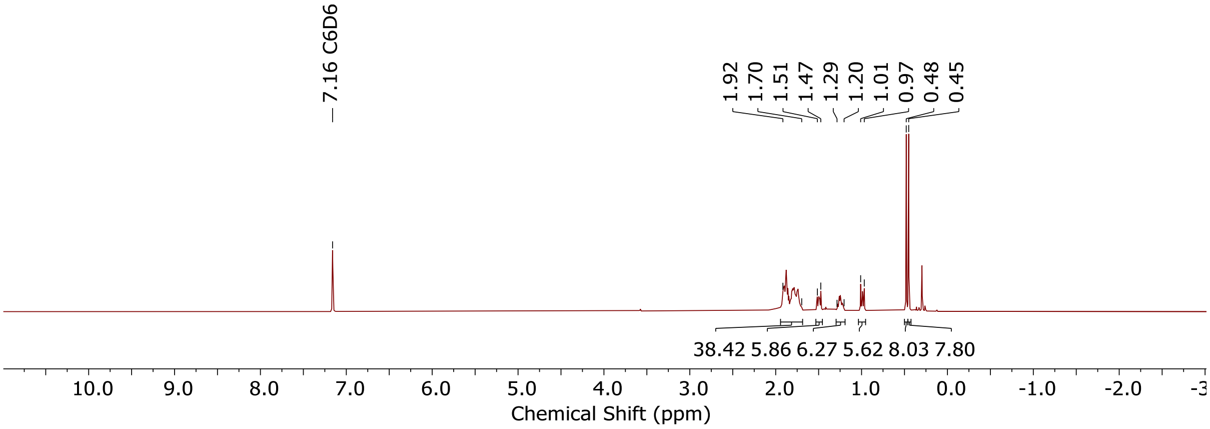

Figure S23. ^{1}H NMR spectrum (C_6D_6) of **7**.

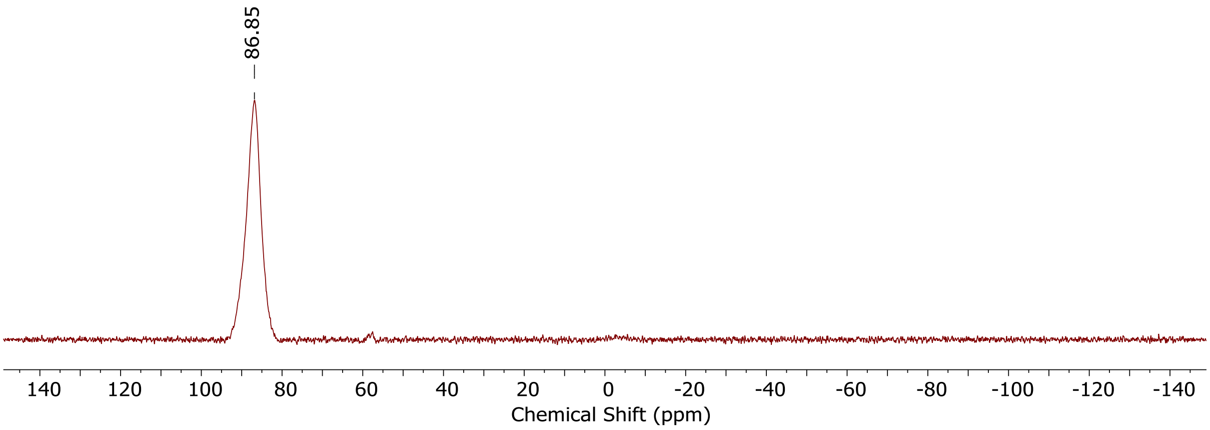

Figure S24. ^{11}B NMR spectrum (C_6D_6) of **7**.

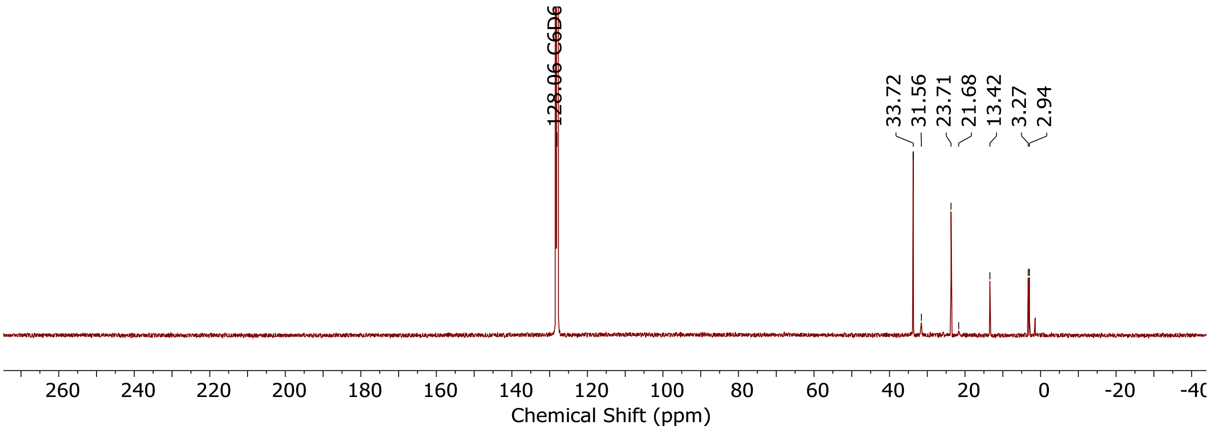

Figure S25. $^{13}C\{^1H\}$ NMR spectrum (C_6D_6) of **7**.

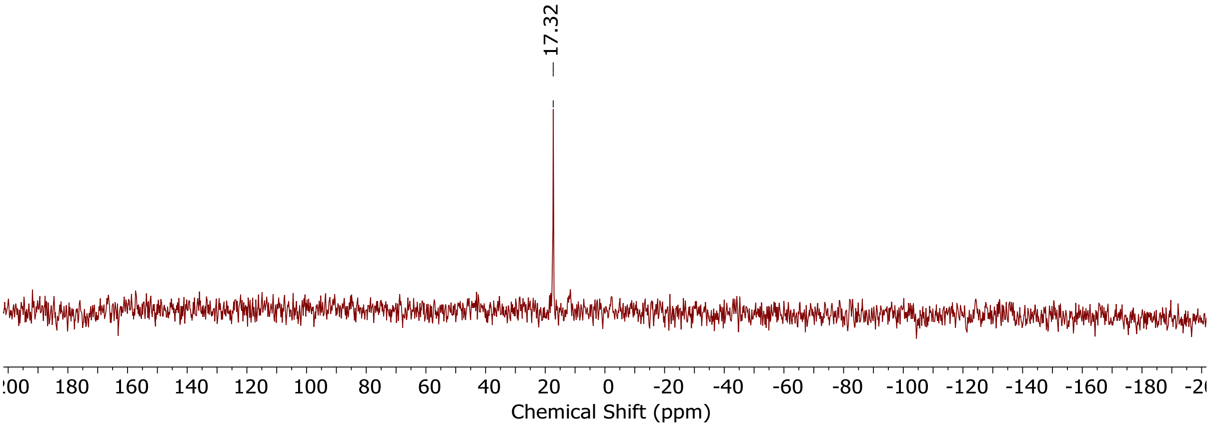

Figure S26. $^{29}Si\{^1H\}$ NMR spectrum (C_6D_6) of **7**.

2.7. Synthesis of $(CH_2CHSiMe_2)_3(As_7)$ (9)

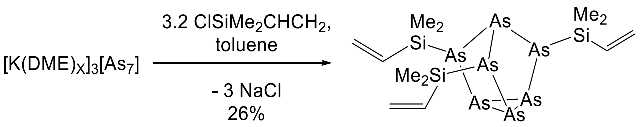

9

Scheme S7. Synthesis of $(CH_2CHSiMe_2)_3As_7$ (**9**).

A Schlenk flask was charged with a stir bar and $[K(DME)]_3[As_7]$ (2 g, ~ 3.1 mmol), toluene (20 mL) was added and the suspension cooled to –78 °C. $ClSiMe_2CHCH_2$ (1.316 g, 10.9 mmol) in toluene (10 mL) was added dropwise. The reaction mixture was warmed to room temperature and the resulting dark purple solution was stirred for two days. The mixture was filtered yielding a clear red solution and the residue further extracted with toluene (2 x 20 mL). The solvent was removed under reduced pressure resulting in a red powder of $(CH_2CHSiMe_2)_3As_7$ (**9**). Crystals suitable for single crystal XRD analysis were obtained from a concentrated toluene solution at –35 °C after several days.

Isolated Yield: 0.627 g, 0.80 mmol, 26 %.

^{1}H NMR (400 MHz, 298 K, C_6D_6): δ = 6.30 (dd, $^3J_{HH}$ = 20.4 Hz, $^3J_{HH}$ = 14.4 Hz, 3H, C*H*), 5.84 (dd, $^3J_{HH}$ = 14.4 Hz, $^2J_{HH}$ = 3.0 Hz, 3H, *cis*-C*H*$_2$), 5.64 (dd, $^3J_{HH}$ = 20.4 Hz, $^2J_{HH}$ = 3.0 Hz, 3H, *trans*-C*H*$_2$), 0.44 (s, 18H, SiC*H*$_3$) ppm.

^{13}C{^{1}H} NMR (101 MHz, 298 K, C_6D_6): δ = 139.97 (s, *C*H), 132.73 (s, *C*H$_2$), 3.09 (s, Si*C*H$_3$), 2.75 (s, Si*C*H$_3$) ppm.

^{29}Si{^{1}H} NMR (79 MHz, 298 K, C_6D_6): δ = 2.22 (s) ppm.

Elemental analysis: Expected/Found: C = 18.48/20.03; H = 3.49/3.99; N = 0/0.

Mass spectrometry (APCI): $C_{12}H_{27}As_7Si_3$+H ([M+H]$^+$); Calcd. = 780.6005, Found = 780.5999.

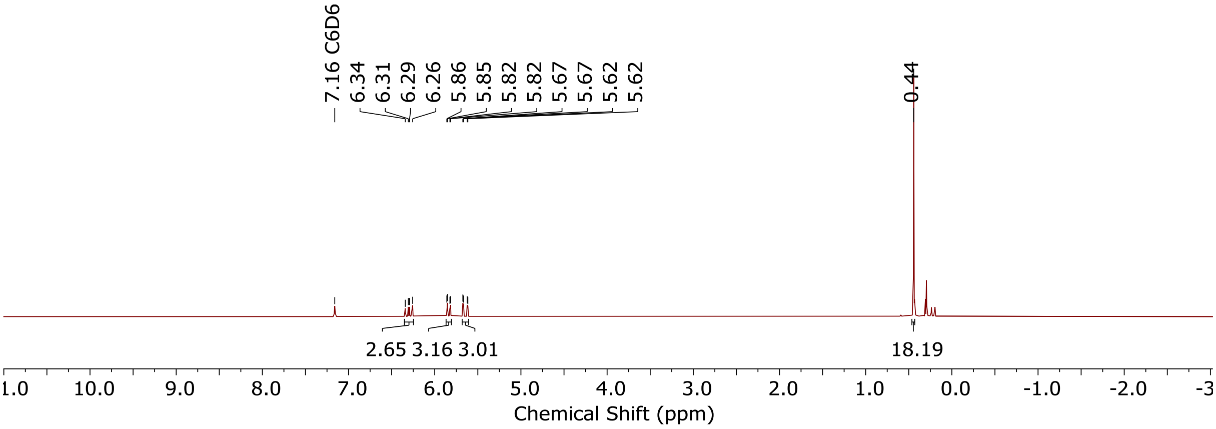

Figure S27. ^{1}H NMR spectrum (C_6D_6) of **9**.

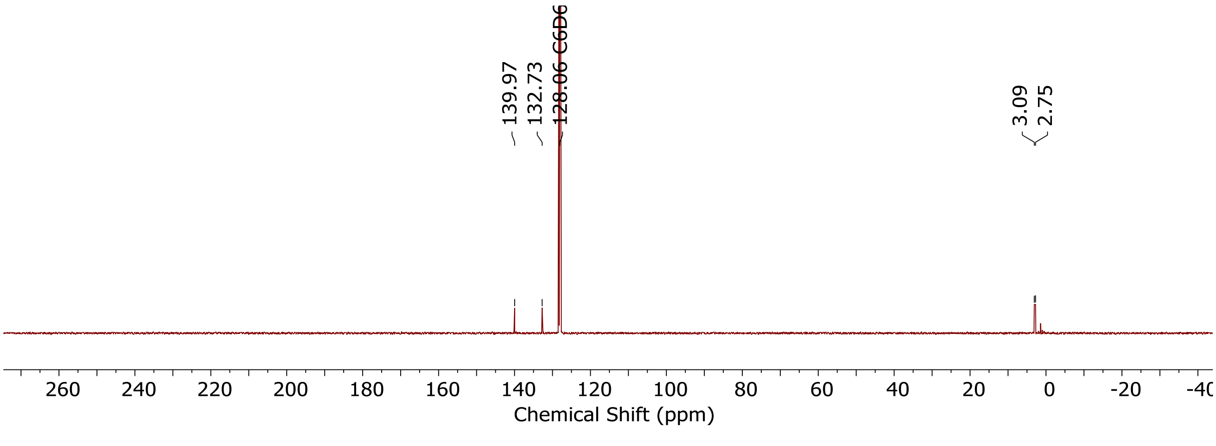

Figure S28. ^{13}C{^{1}H} NMR spectrum (C_6D_6) of **9**.

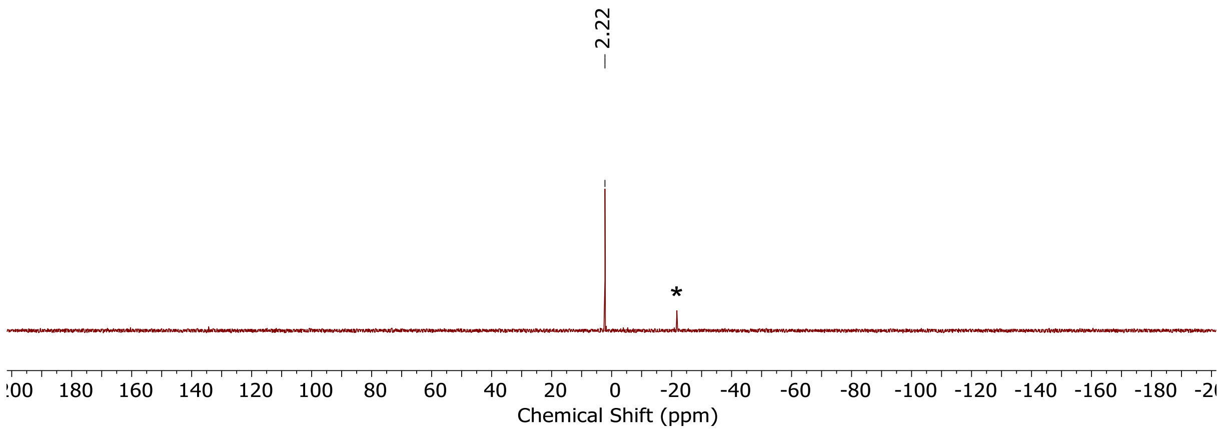

Figure S29. $^{29}Si\{^1H\}$ NMR spectrum (C_6D_6) of **9**. * denotes silicon grease impurity.

2.8. Synthesis of (Mes{(C_8H_{14})BO}CH)$_3$P$_7$ (10)

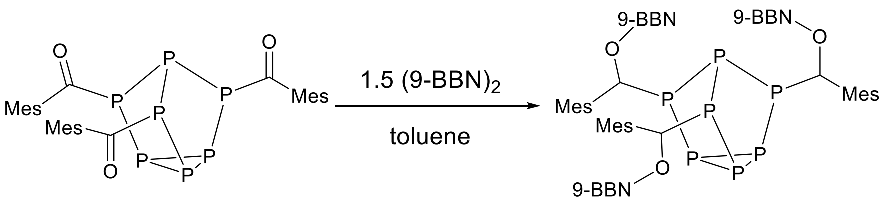

Scheme S8. Synthesis of (Mes{(C_8H_{14})BO}CH)$_3$P$_7$ (**10**).

A Schlenk flask was charged with a stir bar, (MesCO)$_3$(P$_7$) (0.700 g, 1.06 mmol), and 9-BBN dimer (0.389 g, 1.59 mmol). Toluene (10 mL) was added and the reaction heated to 50 °C for 24 hours. The amber solution was cooled to room temperature and volatiles removed under reduced pressure. The yellow solid was extracted with pentane (3 x 10 mL), and removal of volatiles under reduced pressure yielded (Mes{(C_8H_{14})BO}CH)$_3$P$_7$ (**10**) as a yellow solid. Crystals suitable for single crystal XRD analysis were obtained from a concentrated Et_2O solution at –35 °C after several days. Despite multiple recrystallizations, clean characterization by NMR spectroscopy and elemental analysis could not be obtained. However, for completeness, we have provided NMR spectral data for **10**.

Mass spectrometry (APCI): $C_{54}H_{78}B_3O_3P_7$+H ([M+H]$^+$); Calcd. = 1025.4466, Found = 1025.4486.

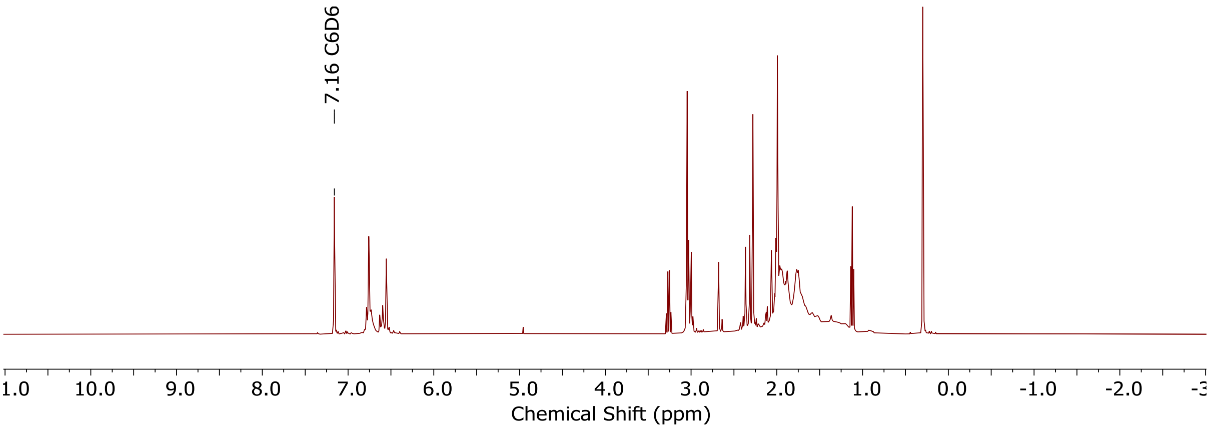

Figure S30. ^{1}H NMR spectrum (C_6D_6) of **10**.

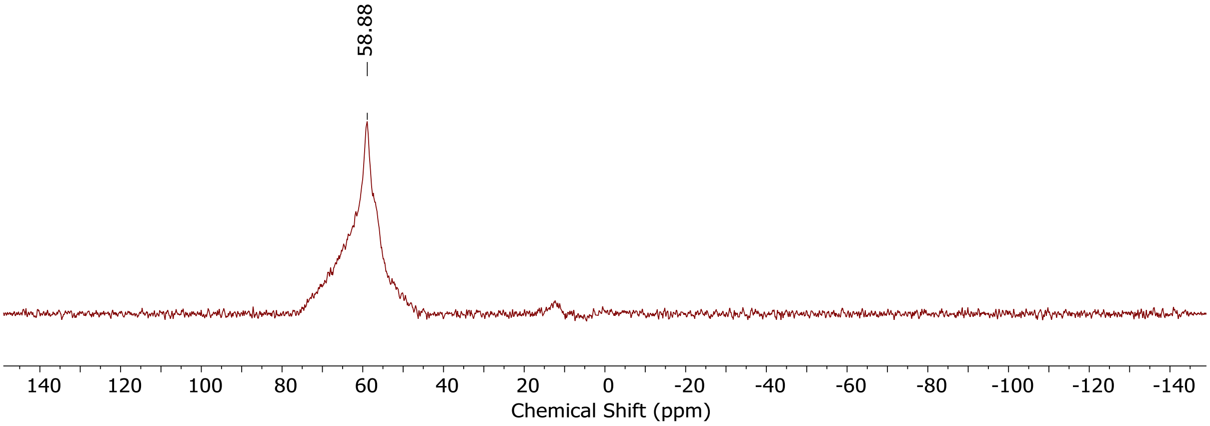

Figure S31. ^{11}B NMR spectrum (C_6D_6) of **10**.

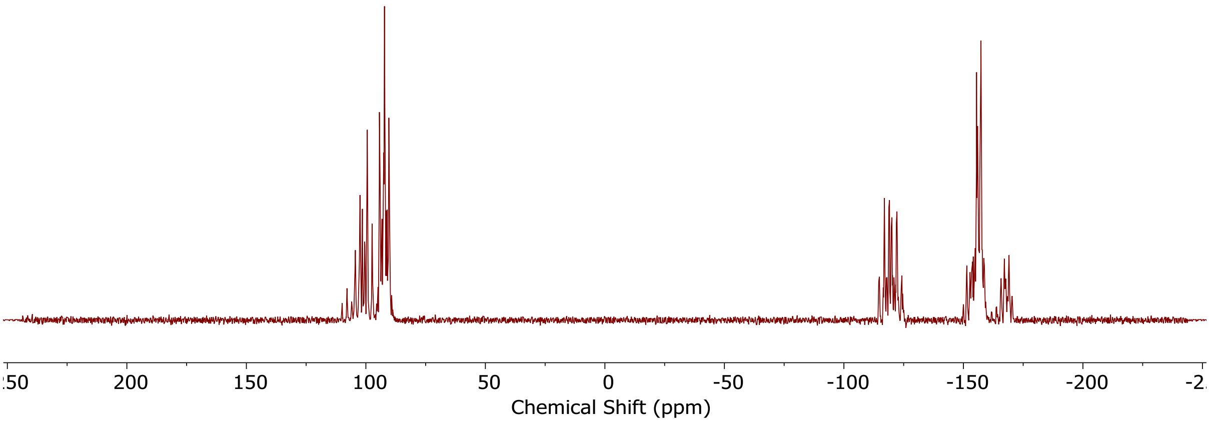

Figure S32. ^{31}P NMR spectrum (C_6D_6) of **6**.

3. Lewis Acidity Examination & Solution Stability

3.1. Gutmann-Beckett Testing

The Gutmann-Beckett method assesses Lewis acidity by complexation of Et_3PO to the Lewis acid and the analyzing the difference in the ^{31}P NMR chemical shift from free Et_3PO and complexed Et_3PO. From this data, an Acceptor Number (AN) is calculated based on Equation 1, where the larger the AN the greater the Lewis acidity.[8]

The Lewis acids (**1–7**) were dissolved in C_6D_6 (0.6 mL) and Et_3PO (1eq per LA site) was added. As an internal reference, a capillary containing PPh_3 in C_6D_6 was added to the NMR tube, which has a chemical shift of –5.33 ppm (verified by independently obtaining the ^{31}P NMR spectrum of PPh_3 in C_6D_6). Due the new method of this data collection, originally reported by Melan and co-workers,[9] both **1** and **8** were reassessed. We internally assessed the method with $B(C_6F_5)_3$ – also reported.

$$\text{LA} + \text{Et}_3\text{PO} \rightleftharpoons \text{LA-OPEt}_3$$

Scheme S9. Complexation of Et_3PO to the Lewis Acid.

$$\text{AN} = 2.21 \times \left(\delta_{\text{P(LA-OPEt}_3)} - 41\right) \quad \text{Eqn(1).}$$

Table S1. Acceptor Numbers (ANs) for **1–7** and $B(C_6F_5)_3$ in C_6D_6.

Catalyst	δ_P(LA-OPEt$_3$)/ ppm	Acceptor Number (AN)
$B(C_6F_5)_3$	75.92	77.2
$(\{C_8H_{14}\}BCH_2CH_2SiMe_2)_3P_7$ (**1**)	54.04	28.8[a]
$ClSiMe_2CH_2CH_2CH_2B\{C_8H_{14}\}$ (**2**)	48.38	16.3
$(\{C_8H_{14}\}BCH_2CH_2CH_2SiMe_2)_3P_7$ (**3**)	50.28	20.5
$ClSiMe_2CH_2CH_2BCy_2$ (**4**)	46.46	12.1
$(\{Cy_2B\}CH_2CH_2SiMe_2)_3P_7$ (**5**)	55.55	18.6
$(\{C_8H_{14}\}BCH_2CH_2SiMe_2)_3As_7$ (**7**)	49.15	18.0
$ClSiMe_2CH_2CH_2B\{C_8H_{14}\}$ (**8**)	49.80	19.4[a]

[a] Reported here, AN readjusted with the new method described above.

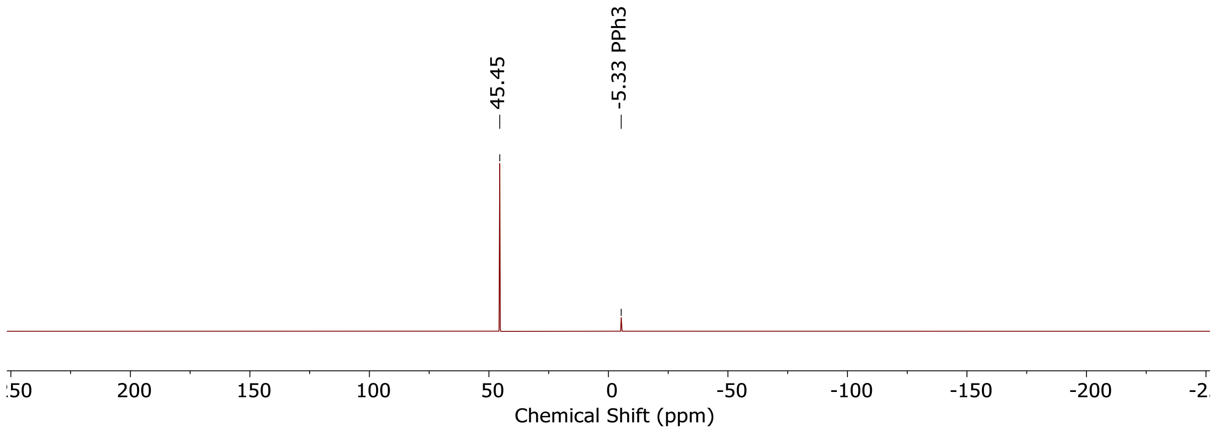

Figure S33. ^{31}P NMR spectrum (C_6D_6) of Et_3PO with PPh_3 insert.

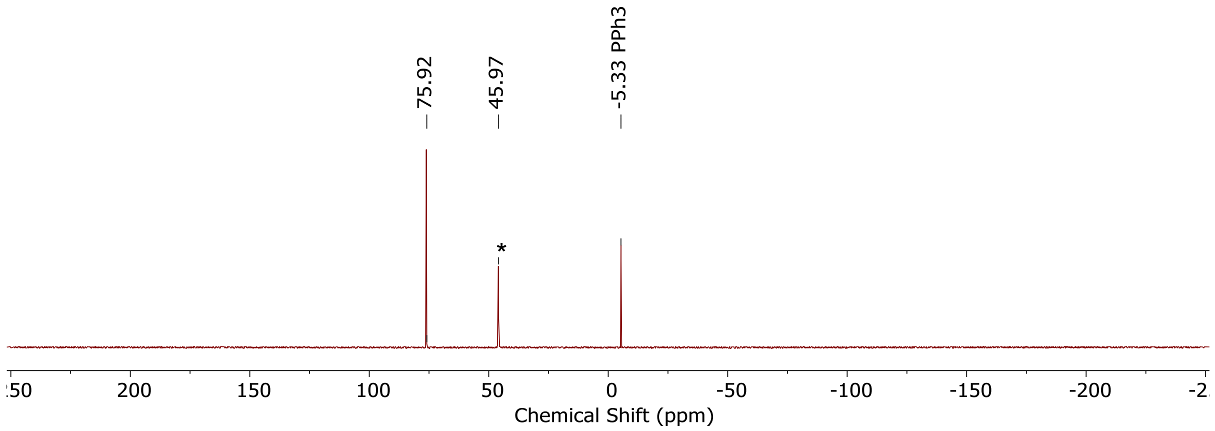

Figure S34. ^{31}P NMR spectrum (C_6D_6) of $B(C_6F_5)_3$ + 1 eq Et_3PO with PPh_3 insert. * denotes free-Et_3PO

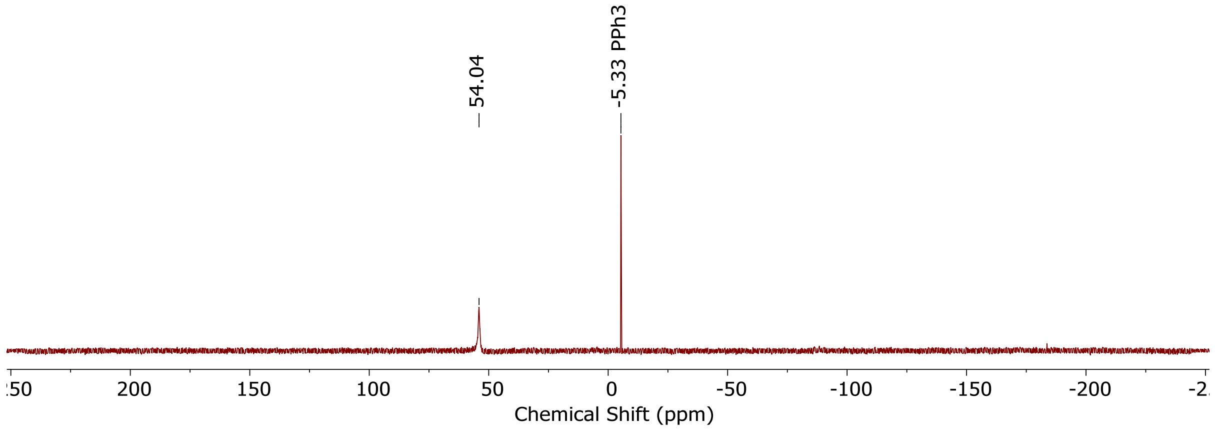

Figure S35. ^{31}P NMR spectrum (C_6D_6) of **1** + 3 eq Et_3PO with PPh_3 insert.

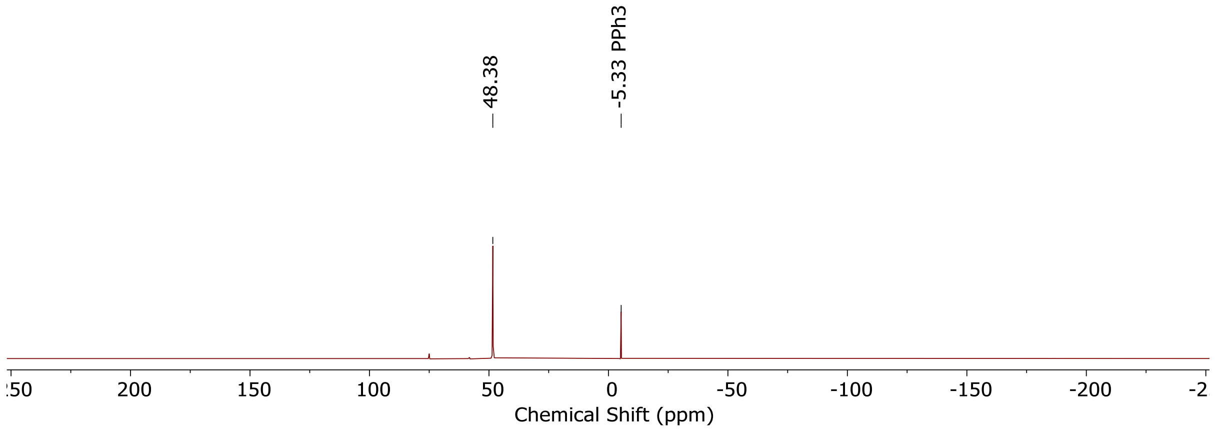

Figure S36. ^{31}P NMR spectrum (C_6D_6) of **2** + Et_3PO with PPh_3 insert.

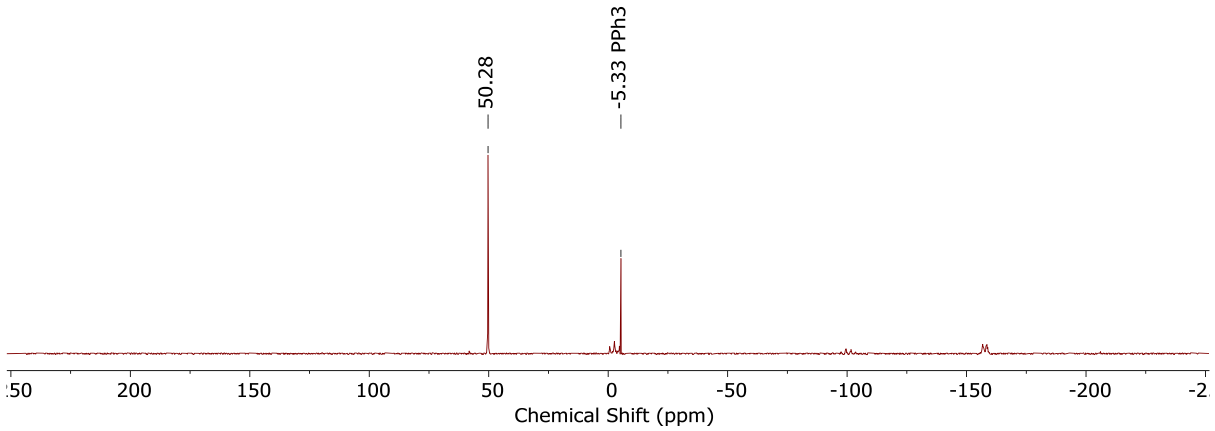

Figure S37. ^{31}P NMR spectrum (C_6D_6) of **3** + 3 eq Et_3PO with PPh_3 insert.

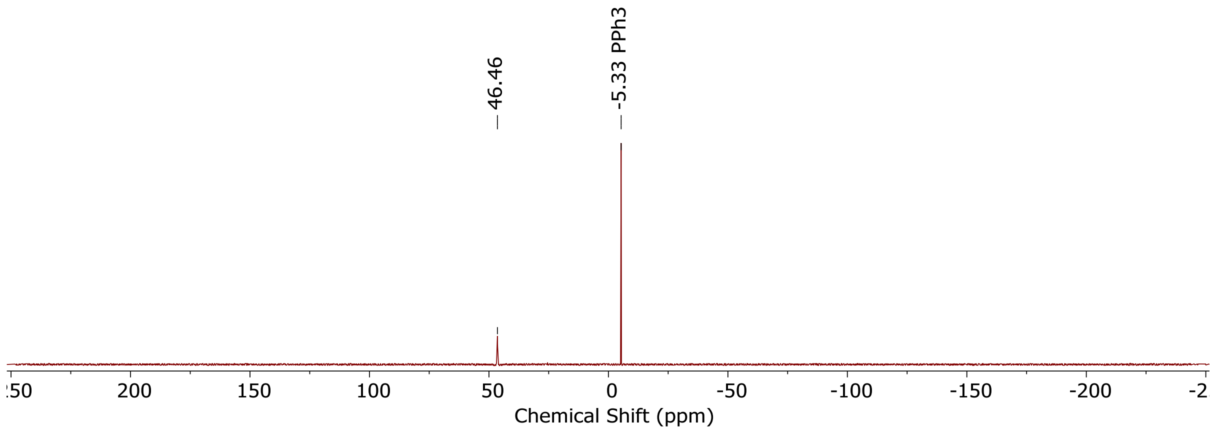

Figure S38. ^{31}P NMR spectrum (C_6D_6) of **4** + Et_3PO with PPh_3 insert.

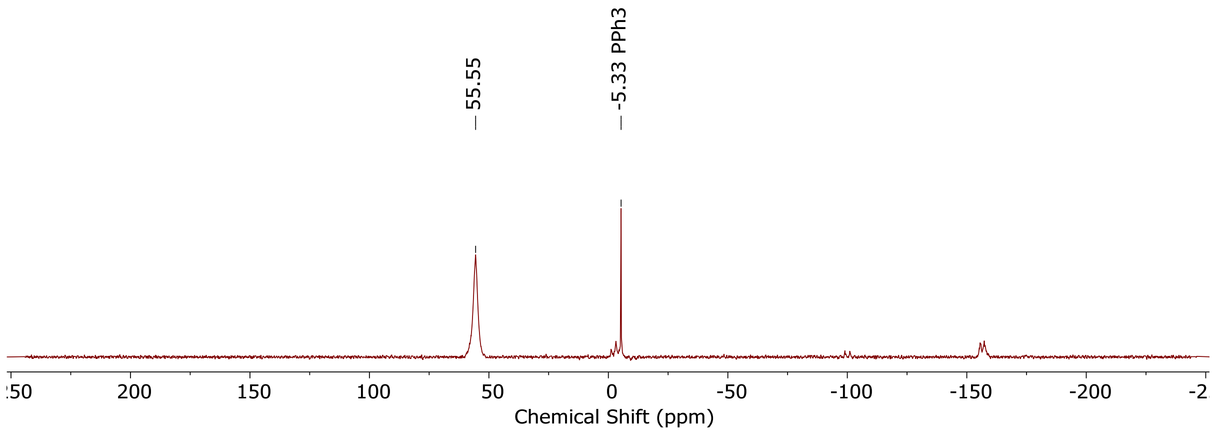

Figure S39. ^{31}P NMR spectrum (C_6D_6) of **5** + 3 eq Et_3PO with PPh_3 insert.

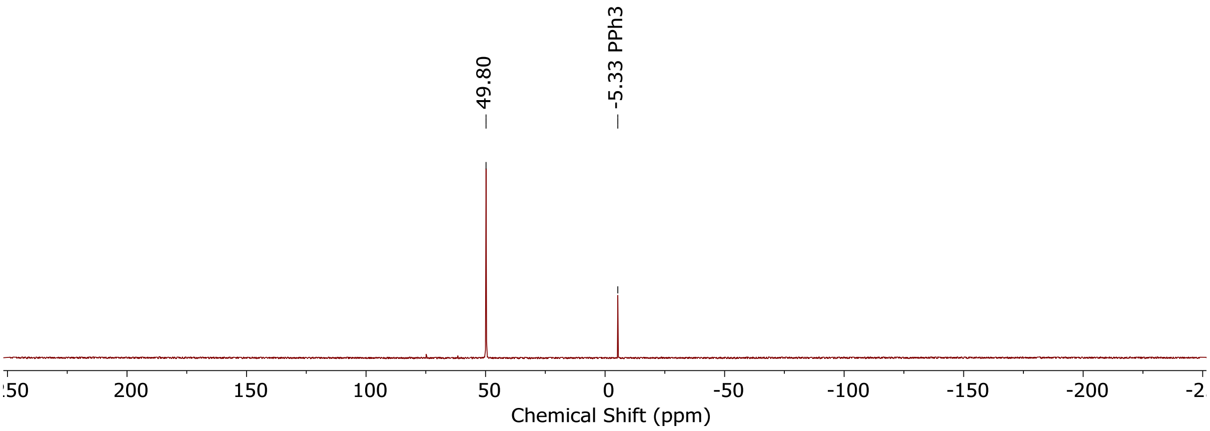

Figure S40. ^{31}P NMR spectrum (C_6D_6) of **8** + 3 eq Et_3PO with PPh_3 insert.

The reaction of ($\{C_8H_{14}\}BCH_2CH_2SiMe_2)_3As_7$ (**7**) with three equivalents of Et_3PO in C_6D_6 showed the expected Lewis Acid-Base adduct formation (allowing for the AN to be calculated), along with secondary reactivity. Full NMR spectra for this reaction provided below.

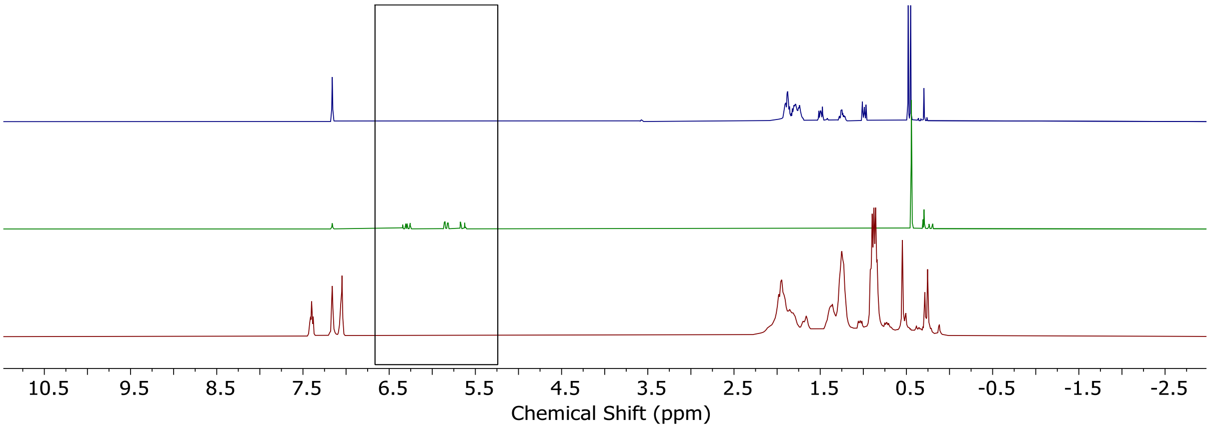

Figure S41. ^{1}H NMR spectrum (C_6D_6) of **7** (top), **9** (middle), and **7** + 3 eq Et_3PO (bottom), boxed region indicating no vinyl formation (*via* retrohydroboration) when Et_3PO is added to **7**.

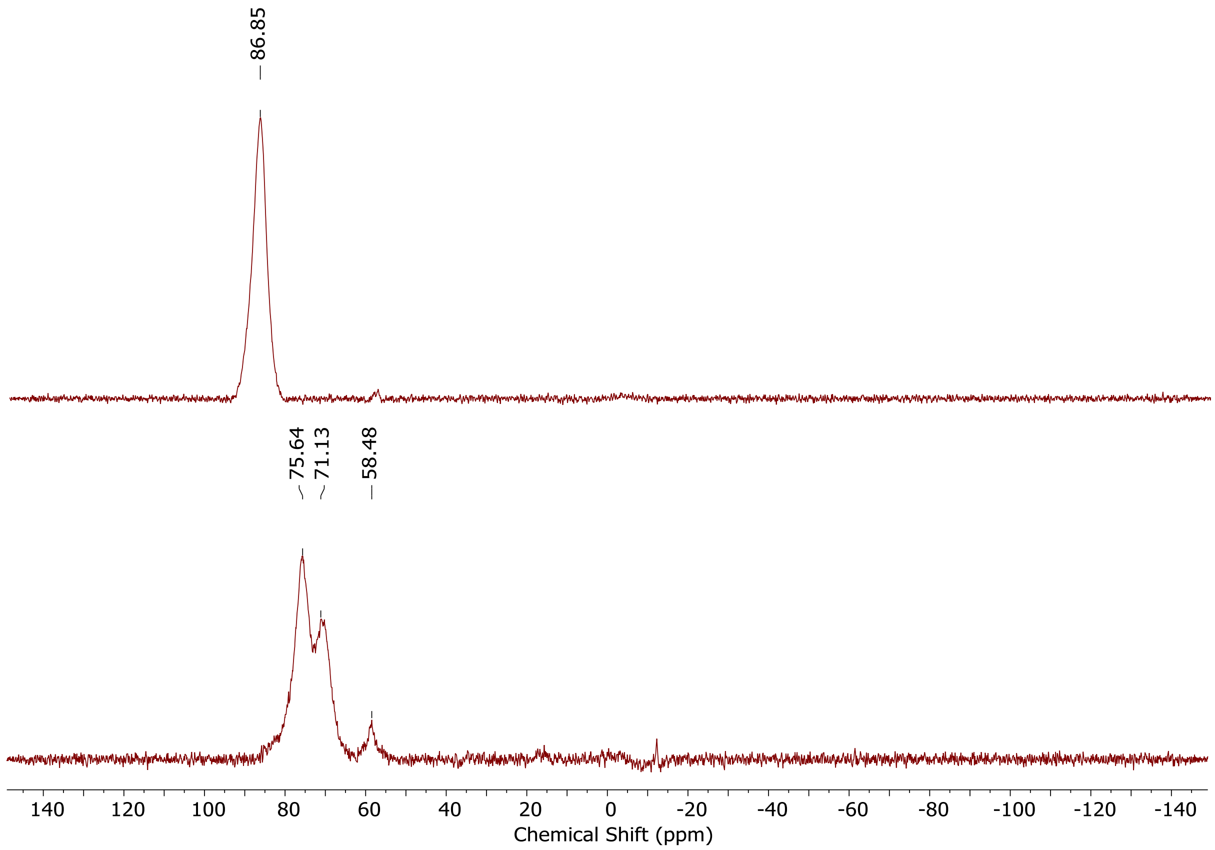

Figure S42. ^{11}B NMR spectrum (C_6D_6) of **7** (top) and **7** + 3 eq Et_3PO (bottom).

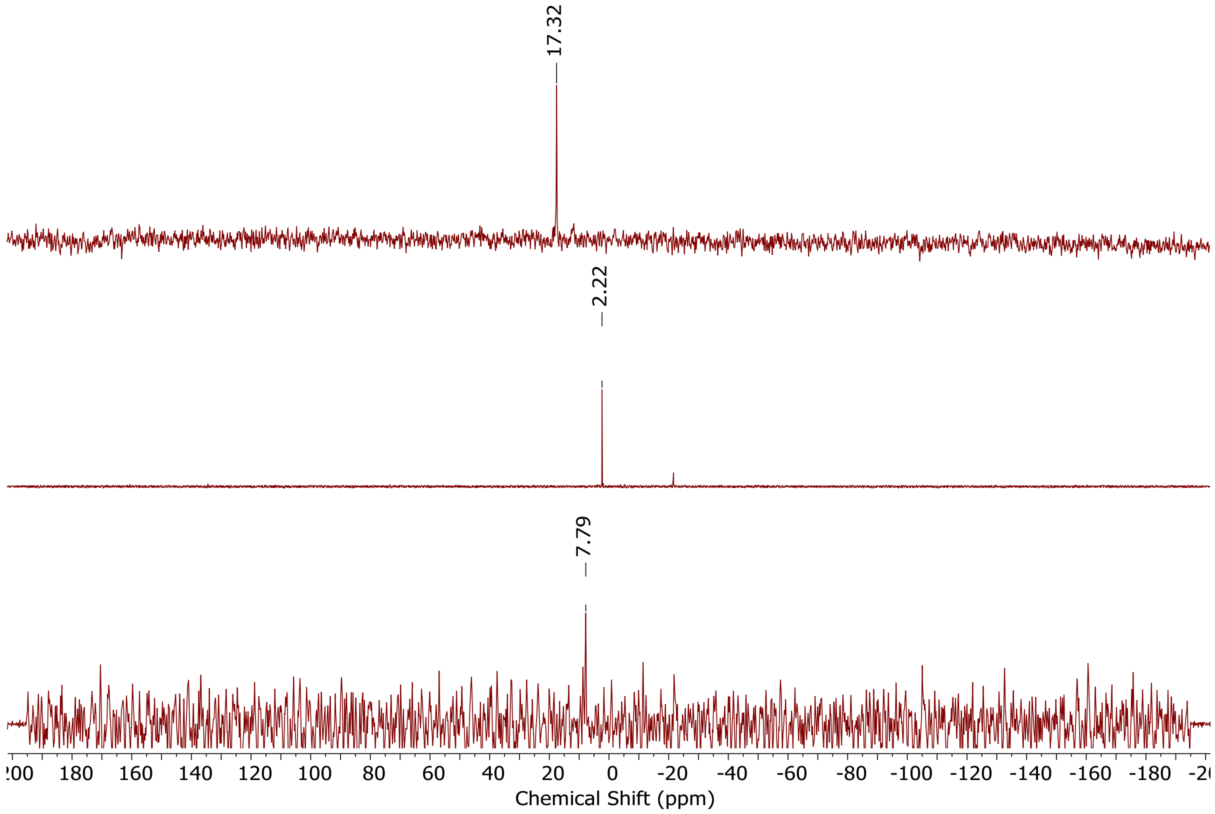

Figure S43. $^{29}Si\{^1H\}$ NMR spectrum (C_6D_6) of **7** (top), **9** (middle), and **7** + 3 eq Et_3PO (bottom).

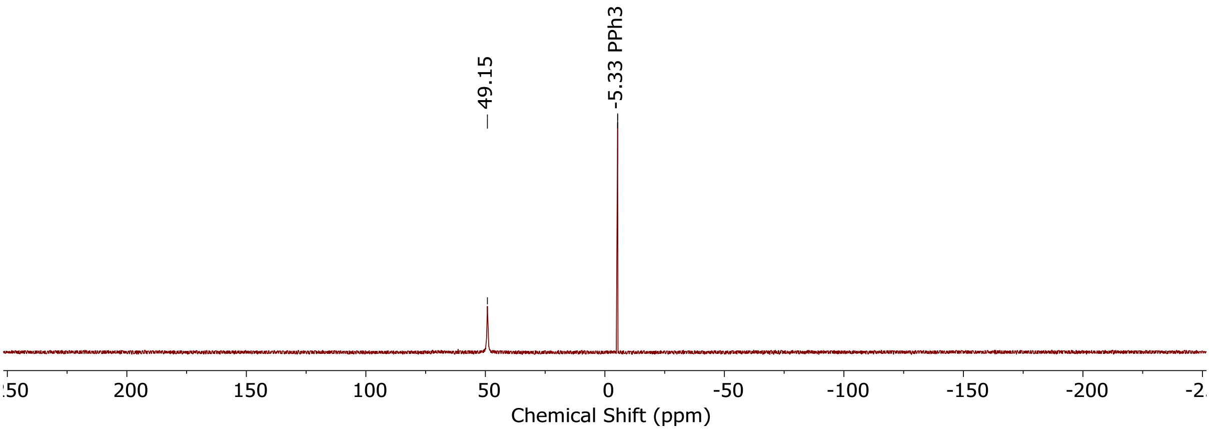

Figure S44. ^{31}P NMR spectrum (C_6D_6) of **7** + 3 eq Et_3PO with PPh_3 insert.

Similarly, addition of three equivalents of Et_3PO to $(CH_2CHSiMe_2)_3As_7$ (**9**) in C_6D_6 showed similar changes by NMR spectroscopy, including visually a dark-brown solution formation. Consistent with this secondary reactivity being based at the $[As_7]$ rather than the Lewis acidic sites. Full NMR spectra for this reaction provided below.

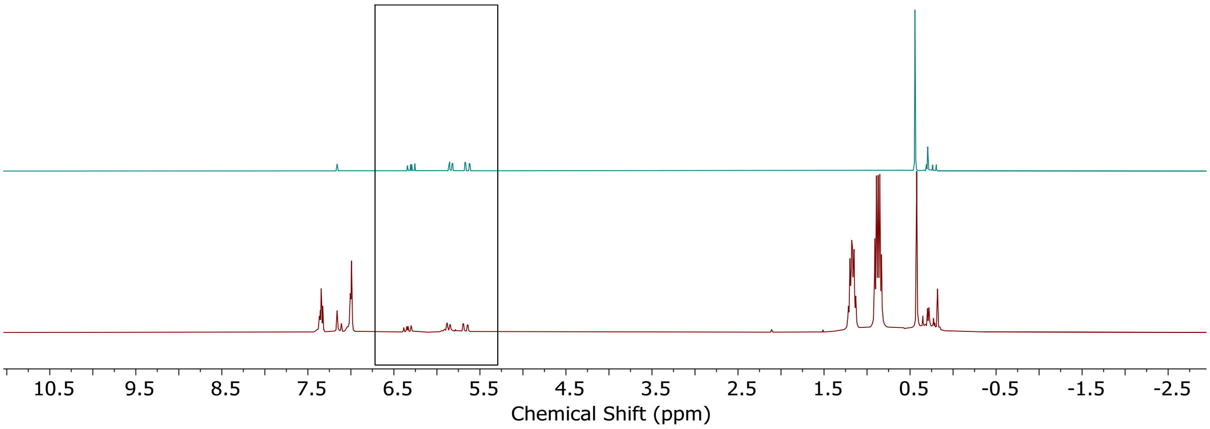

Figure S45. ^{1}H NMR spectrum (C_6D_6) of **9** (top), and **9** + 3 eq Et_3PO (bottom), boxed region indicating vinyl region of **9**.

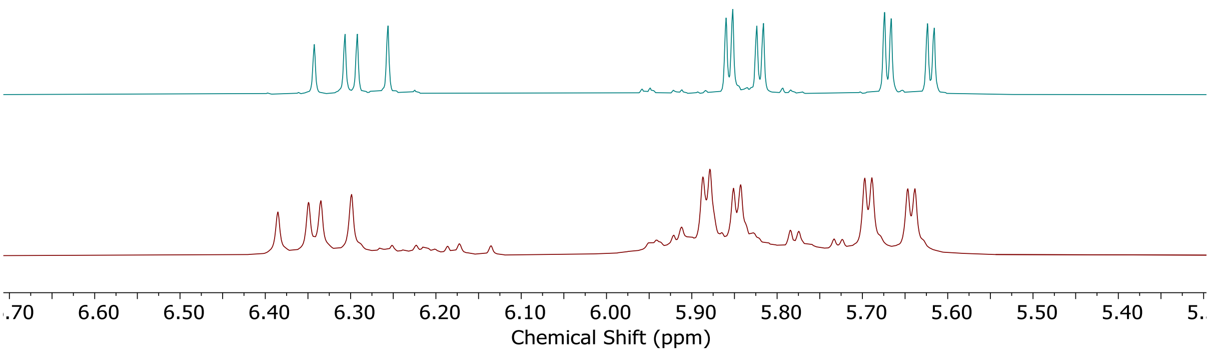

Figure S46. Zoomed ^{1}H NMR spectrum (C_6D_6) of **9** (top), and **9** + 3 eq Et_3PO (bottom).

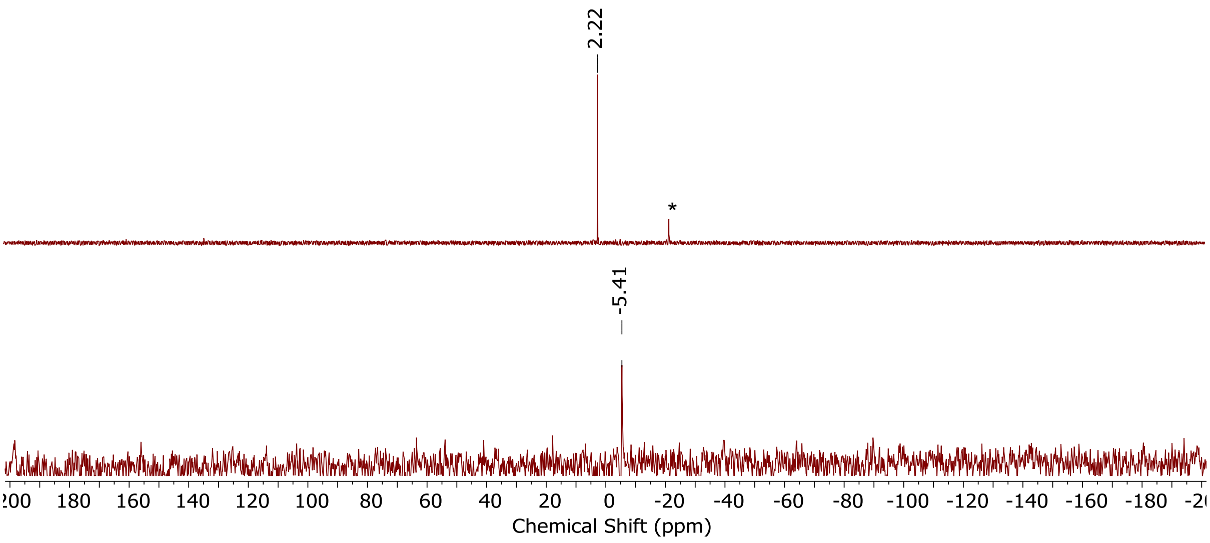

Figure S47. $^{29}Si\{^1H\}$ NMR spectrum (C_6D_6) of **9** (top), and **9** + 3 eq Et_3PO (bottom). * denotes silicon grease

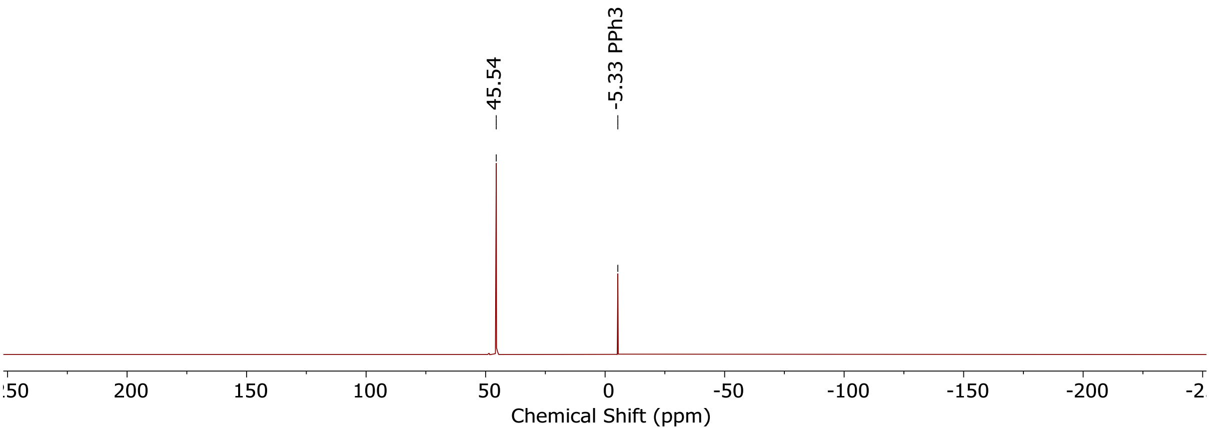

Figure S48. ^{31}P NMR spectrum (C_6D_6) of **9** + 3 eq Et_3PO with PPh_3 insert.

3.2. Fluoride Ion Affinity (FIA)

The Lewis acidity of selected Lewis acids was probed by calculation of its Fluoride Ion Affinity (FIA) using the CF_2O-CF_3O^- reference system (also known as Christe's method).[10] This method has been extensively used in literature using the BP86/SV(p) level of theory. The calculated FIA of $B(C_6F_5)_3$, SbF_5 and Me_3Si^+ are consistent with literature values and used as benchmarks.[11]

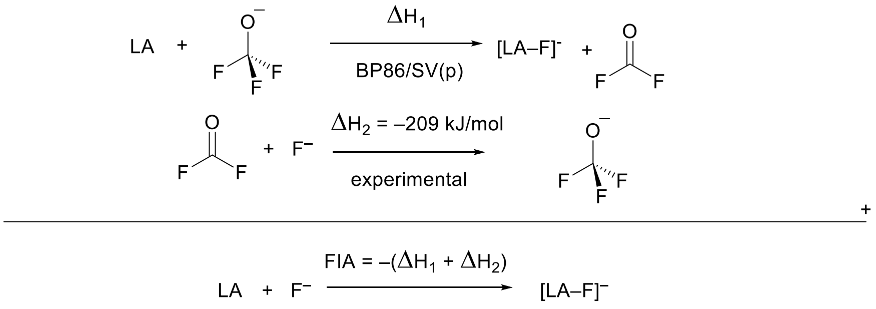

Scheme S10. FIA calculation.

Table S2. FIA values of selected Lewis Acids and **1**–**8**, **10**. Calculatated at the BP86/SV(p) level of theory.

Compound	1st FIA (kJ/mol)	2nd FIA (kJ/mol)	3rd FIA (kJ/mol)
$[Me_3Si]^+$	948	-	-
SbF_5	489	-	-
$B(C_6F_5)_3$	444	-	-
HBBN	328	-	-
$(\{C_8H_{14}\}BCH_2CH_2SiMe_2)_3P_7$ (**1**)[2]	350	220	113
$ClSiMe_2CH_2CH_2CH_2B\{C_8H_{14}\}$ (**2**)	340	-	-
$(\{C_8H_{14}\}BCH_2CH_2CH_2SiMe_2)_3P_7$ (**3**)	321	219	118
$ClSiMe_2CH_2CH_2BCy_2$ (**4**)	344	-	-
$(\{Cy_2B\}CH_2CH_2SiMe_2)_3P_7$ (**5**)	356	220	99
$(\{(C_6F_5)_2B\}CH_2CH_2SiMe_2)_3P_7$ (**6**)	438	323	215
$(\{C_8H_{14}\}BCH_2CH_2SiMe_2)_3As_7$ (**7**)	348	225	130
$ClSiMe_2CH_2CH_2B\{C_8H_{14}\}$ (**8**)[2]	340	-	-
$(Mes\{(C_8H_{14})BO\}CH)_3P_7$ (**10**)	344	166	–7

3.3. Hydride Ion Affinity (HIA)

Additionally, Lewis acidity was assessed by calculation of the Hydride Ion Affinity (HIA) using the $Me_3Si^+ \rightarrow Me_3SiH$ reaction as a reference.[12] The calculated HIA of $B(C_6F_5)_3$, SbF_5 and Me_3Si^+ are consistent with literature values and used as benchmarks.

$$\text{LA} + Me_3SiH \xrightarrow[\text{BP86/SV(p)}]{\Delta H_1} [\text{LA–H}]^- + Me_3Si^+$$

$$Me_3Si^+ + H^- \xrightarrow[\text{G3}]{\Delta H_2} Me_3SiH$$

$$\text{LA} + H^- \xrightarrow{\text{HIA} = -(\Delta H_1 + \Delta H_2)} [\text{LA–H}]^-$$

Scheme S11. HIA calculation.

Table S3. HIA values of selected Lewis Acids and **1**–**7**, **9**. Calculatated at the BP86/SV(p) level of theory.

Compound	1st HIA (kJ/mol)	2nd HIA (kJ/mol)	3rd HIA (kJ/mol)
$[Me_3Si]^+$	819	-	-
SbF_5	545	-	-
$B(C_6F_5)_3$	487	-	-
$(\{C_8H_{14}\}BCH_2CH_2SiMe_2)_3P_7$ (**1**)[2]	352	[a]	[a]
$ClSiMe_2CH_2CH_2CH_2B\{C_8H_{14}\}$ (**2**)	341	-	-
$(\{C_8H_{14}\}BCH_2CH_2CH_2SiMe_2)_3P_7$ (**3**)	323	234	104
$ClSiMe_2CH_2CH_2BCy_2$ (**4**)	347	-	-
$(\{Cy_2B\}CH_2CH_2SiMe_2)_3P_7$ (**5**)	358	223	99
$(\{(C_6F_5)_2B\}CH_2CH_2SiMe_2)_3P_7$ (**6**)	465	352	241
$(\{C_8H_{14}\}BCH_2CH_2SiMe_2)_3As_7$ (**7**)	346	229	129
$ClSiMe_2CH_2CH_2B\{C_8H_{14}\}$ (**8**)[2]	341	-	-
$(Mes\{(C_8H_{14})BO\}CH)_3P_7$ (**10**)	334	153	–20

[a] Global minima could not be reached repeatedly thus 2nd/3rd HIA could not be determined.

3.4. Energies & Enthalpies of selected Lewis Acids

Table S4. Energies and Enthalpies of selected Lewis Acids and **1**–**7**, **9**.

**Compound**	**FIA (kJ/mol)**	**HIA (kJ/mol)**	**G [a.u.]**	**E [a.u.]**
CF_2O	0		–312.7854163	–312.76742
$[CF_3O]^-$			–412.6141114	–412.594474
$[Me_3Si]^+$	948	819	–408.703695	–-408.817035912
Me_3SiF			–508.9311529	–508.81189
Me_3SiH			–409.575699	–409.699318773
H^-			–0.486377	–0.488737487818
SbF_5	489	545	–6814.717684	–6814.698435
$[SbF_6]^-$			–6914.652886	–6914.631992
$[HSbF_5]^-$			–6815.412861	–6815.43952664
$B(C_6F_5)_3$	444	487	–2206.628214	–2206.448141
$[FB(C_6F_5)_3]^-$			–2306.547159	–2306.364788
$[HB(C_6F_5)_3]^-$			–2207.14043	–2207.3285042
$(\{C_8H_{14}\}BCH_2CH_2SiMe_2)_3P_7$ (**1**)[REF]	350	352	–4745.076172	–4746.14535287
$[(\{C_8H_{14}\}BCH_2CH_2SiMe_2)_2(\{C_8H_{14}\}BFCH_2CH_2SiMe_2)P_7]^-$	220		–4844.957021	–4846.02791784
$[(\{C_8H_{14}\}BCH_2CH_2SiMe_2)(\{C_8H_{14}\}BFCH_2CH_2SiMe_2)_2P_7]^{2-}$	113		–4944.788136	–4945.85997428
$[(\{C_8H_{14}\}BFCH_2CH_2SiMe_2)_3P_7]^{3-}$			–5044.578445	–5045.65104455
$[(\{C_8H_{14}\}BCH_2CH_2SiMe_2)_2(\{C_8H_{14}\}BHCH_2CH_2SiMe_2)P_7]^-$		[a]	–4745.716907	–4746.79173881

$[(\{C_8H_{14}\}BCH_2CH_2SiMe_2)(\{C_8H_{14}\}BHCH_2CH_2SiMe_2)_2P_7]^{2-}$		[a]	[a]	[a]
$[(\{C_8H_{14}\}BHCH_2CH_2SiMe_2)_3P_7]^{3-}$			–4746.850998	–4747.93741324
$ClSiMe_2CH_2CH_2CH_2B\{C_8H_{14}\}$ (**2**)	340	341	–1284.794475	–1285.10201924
$[ClSiMe_2CH_2CH_2CH_2FB\{C_8H_{14}\}]^-$			–1384.599320	–1384.97994664
$[ClSiMe_2CH_2CH_2CH_2HB\{C_8H_{14}\}]^-$			–1285.358856	–1285.74416536
$(\{C_8H_{14}\}BCH_2CH_2CH_2SiMe_2)_3P_7$ (**3**)	321	323	–4863.020371	–4863.98826358
$[(\{C_8H_{14}\}BCH_2CH_2CH_2SiMe_2)_2(\{C_8H_{14}\}BFCH_2CH_2CH_2SiMe_2)P_7]^-$	219		–4962.894003	–4963.86067138
$[(\{C_8H_{14}\}BCH_2CH_2CH_2SiMe_2)(\{C_8H_{14}\}BFCH_2CH_2CH_2SiMe_2)_2P_7]^{2-}$	118		–5062.727301	–5063.69311020
$[(\{C_8H_{14}\}BFCH_2CH_2CH_2SiMe_2)_3P_7]^{3-}$			–5162.514419	–5163.48500458
$[(\{C_8H_{14}\}BCH_2CH_2CH_2SiMe_2)_2(\{C_8H_{14}\}BHCH_2CH_2CH_2SiMe_2)P_7]^-$		234	–4863.465496	–4864.62512850
$[(\{C_8H_{14}\}BCH_2CH_2CH_2SiMe_2)(\{C_8H_{14}\}BHCH_2CH_2CH_2SiMe_2)_2P_7]^{2-}$		104	–4864.061499	–4865.22725733
$[(\{C_8H_{14}\}BHCH_2CH_2CH_2SiMe_2)_3P_7]^{3-}$			–4864.607785	–4865.77856395
$ClSiMe_2CH_2CH_2BCy_2$ (**4**)	344	347	–1402.493194	–1402.95972201
$[ClSiMe_2CH_2CH_2FBCy_2]^-$			–1502.371777	–1502.83892335
$[ClSiMe_2CH_2CH_2HBCy_2]^-$			–1403.132163	–1403.60390285
$(\{Cy_2B\}CH_2CH_2CH_2SiMe_2)_3P_7$ (**5**)	356	358	–5216.144645	–5217.56122576
$[(\{Cy_2B\}CH_2CH_2CH_2SiMe_2)_2$ $(\{Cy_2B\}FCH_2CH_2CH_2SiMe_2)P_7]^-$	220		–5316.027579	–5317.23115040
$[(\{Cy_2B\}CH_2CH_2CH_2SiMe_2)$ $(\{Cy_2B\}FCH_2CH_2CH_2SiMe_2)_2P_7]^{2-}$	99		–5415.858642	–5417.09385849
$[(\{Cy_2B\}FCH_2CH_2CH_2SiMe_2)_3P_7]^{3-}$			–5515.643876	–5517.06201541

$[(\{Cy_2B\}CH_2CH_2CH_2SiMe_2)_2(\{Cy_2B\}HCH_2CH_2CH_2SiMe_2)P_7]^-$		223	–5216.787851	–5218.20988692
$[(\{Cy_2B\}CH_2CH_2CH_2SiMe_2)(\{Cy_2B\}HCH_2CH_2CH_2SiMe_2)_2P_7]^{2-}$		99	–5217.379519	–5218.80662706
$[(\{Cy_2B\}HCH_2CH_2CH_2SiMe_2)_3P_7]^{3-}$			–5217.923975	–5219.35608544
$(\{(C_6F_5)_2B\}CH_2CH_2SiMe_2)_3P_7$ (**6**)	438	465	–8169.793675	–8170.057647
$[(\{(C_6F_5)_2B\}CH_2CH_2CH_2SiMe_2)_2(\{(C_6F_5)_2B\}FCH_2CH_2CH_2SiMe_2)P_7]^-$	323		–8269.708108	–8269.974419
$[(\{(C_6F_5)_2B\}CH_2CH_2CH_2SiMe_2)(\{(C_6F_5)_2B\}FCH_2CH_2CH_2SiMe_2)_2P_7]^{2-}$	215		–8369.578699	–8369.843846
$[(\{(C_6F_5)_2B\}FCH_2CH_2CH_2SiMe_2)_3P_7]^{3-}$			–8469.408055	–8469.678889
$[(\{(C_6F_5)_2B\}CH_2CH_2CH_2SiMe_2)_2(\{(C_6F_5)_2B\}HCH_2CH_2CH_2SiMe_2)P_7]^-$		352	–8170.477503	–8170.741738
$[(\{(C_6F_5)_2B\}CH_2CH_2CH_2SiMe_2)(\{(C_6F_5)_2B\}HCH_2CH_2CH_2SiMe_2)_2P_7]^{2-}$		241	–8171.118433	–8171.380015
$[(\{(C_6F_5)_2B\}HCH_2CH_2CH_2SiMe_2)_3P_7]^{3-}$			–8171.716893	–8171.982767
$(\{C_8H_{14}\}BCH_2CH_2SiMe_2)_3As_7$ (**7**)	348	346	–18007.305056	–18008.3710040
$[(\{C_8H_{14}\}BCH_2CH_2SiMe_2)_2(\{C_8H_{14}\}BFCH_2CH_2SiMe_2)As_7]^-$	225		–18107.184909	–18108.2507468
$[(\{C_8H_{14}\}BCH_2CH_2SiMe_2)(\{C_8H_{14}\}BFCH_2CH_2SiMe_2)_2As_7]^{2-}$	130		–18207.017921	–18208.0861601
$[(\{C_8H_{14}\}BFCH_2CH_2SiMe_2)_3As_7]^{3-}$			–18306.814958	–18307.8837181
$[(\{C_8H_{14}\}BCH_2CH_2SiMe_2)_2(\{C_8H_{14}\}BHCH_2CH_2SiMe_2)As_7]^-$		229	–18007.943562	–18009.0150699

$[(\{C_8H_{14}\}BCH_2CH_2SiMe_2)(\{C_8H_{14}\}BHCH_2CH_2SiMe_2)_2As_7]^{2-}$		129	–18008.537643	–18009.6151870
$[(\{C_8H_{14}\}BHCH_2CH_2SiMe_2)_3As_7]^{3-}$			–18009.093705	–18010.1763231
$ClSiMe_2CH_2CH_2B\{C_8H_{14}\}$ (**8**)[REF]	340	341	–1245.470520	–1245.82099496
$[ClSiMe_2CH_2CH_2FB\{C_8H_{14}\}]^-$			–1345.347565	–1345.69956868
$[ClSiMe_2CH_2CH_2HB\{C_8H_{14}\}]^-$			–1246.107217	–1246.46379467
$(\{C_8H_{14}\}BO(Mes)CH)_3P_7$ (**10**)	344	334	–4791.157678	–4792.42601356
$[(\{C_8H_{14}\}BO(Mes)CH)_2(\{C_8H_{14}\}BFO(Mes)CH)P_7]^-$	166		–4891.036334	–4892.30523062
$[(\{C_8H_{14}\}BO(Mes)CH)(\{C_8H_{14}\}BFO(Mes)CH)_2P_7]^{2-}$	–7		–4990.846968	–4992.11721777
$[(\{C_8H_{14}\}BFO(Mes)CH)_3P_7]^{3-}$			–5090.591857	–5091.86136678
$[(\{C_8H_{14}\}BO(Mes)CH)_2(\{C_8H_{14}\}BHO(Mes)CH)P_7]^-$		153	–4791.791445	–4793.06521808
$[(\{C_8H_{14}\}BO(Mes)CH)(\{C_8H_{14}\}BHO(Mes)CH)_2P_7]^{2-}$		–20	–4792.356521	–4793.63641032
$[(\{C_8H_{14}\}BHO(Mes)CH)_3P_7]^{3-}$			–4792.855622	–4794.14048889

[a] Global minima could not be reached repeatedly thus 2nd/3rd HIA could not be determined.

3.5. HOMO and LUMO Representations

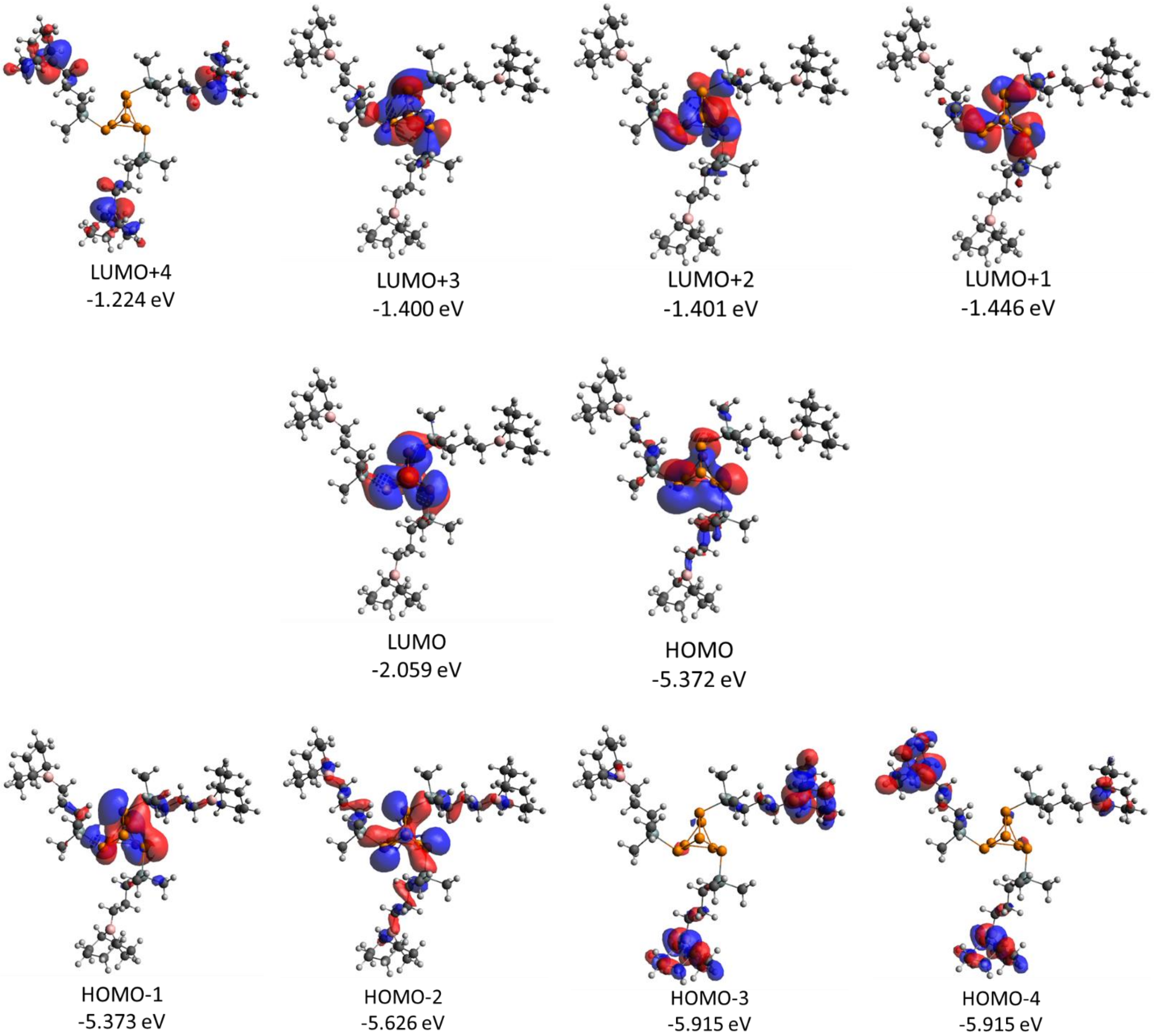

Figure S49. HOMO and LUMO Kohn-Sham images of **3**.

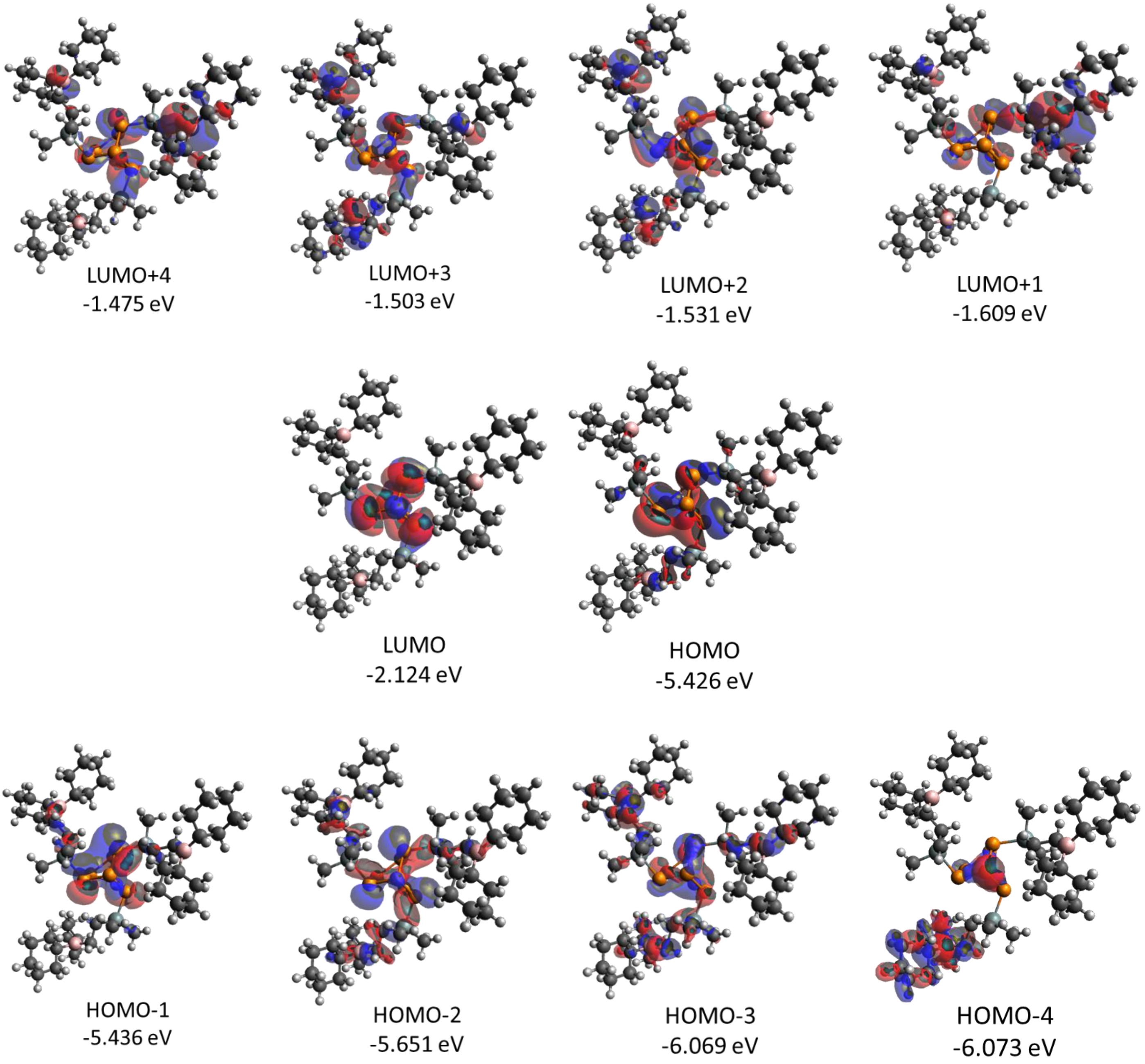

Figure S50. HOMO and LUMO Kohn-Sham images of **5**.

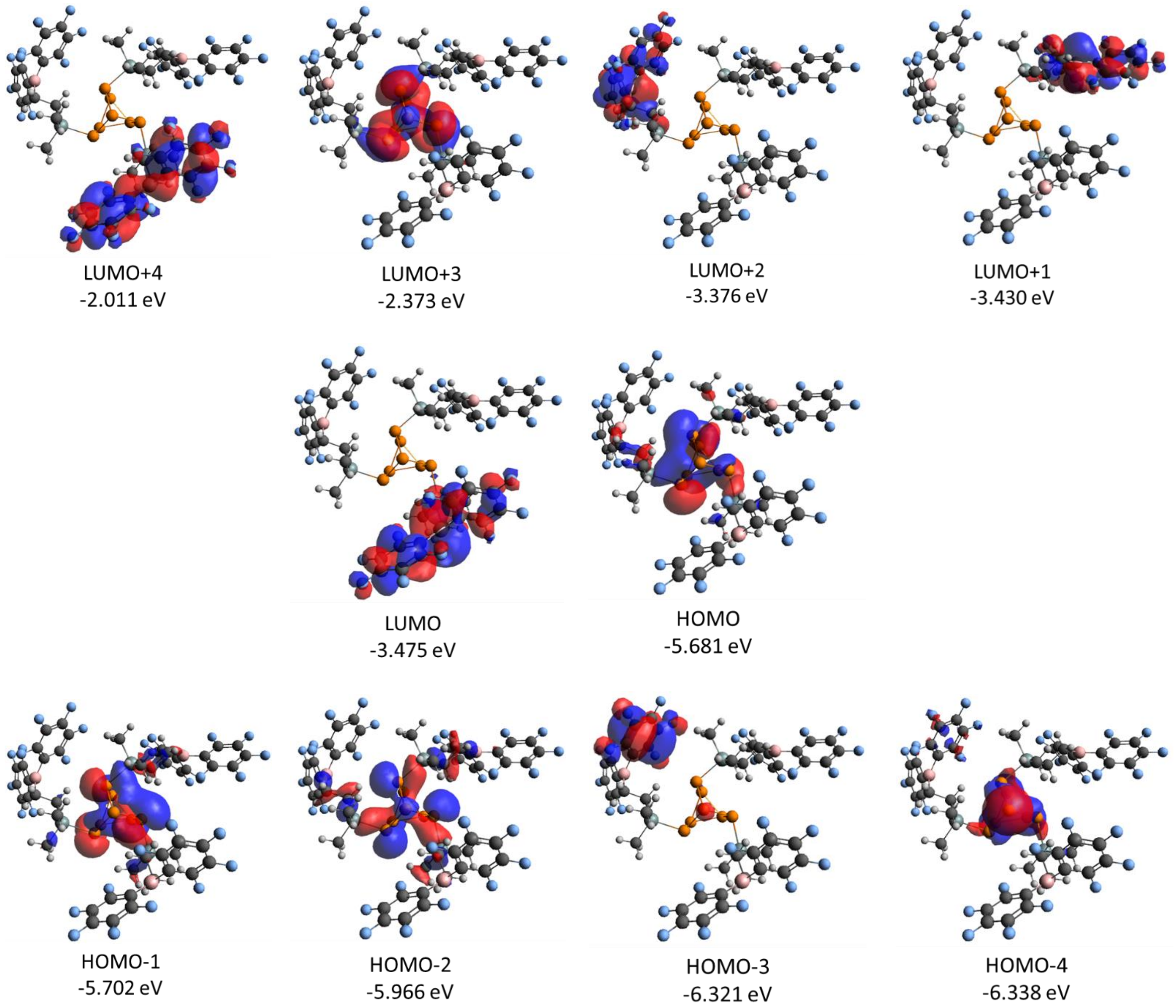

Figure S51. HOMO and LUMO Kohn-Sham images of **6**.

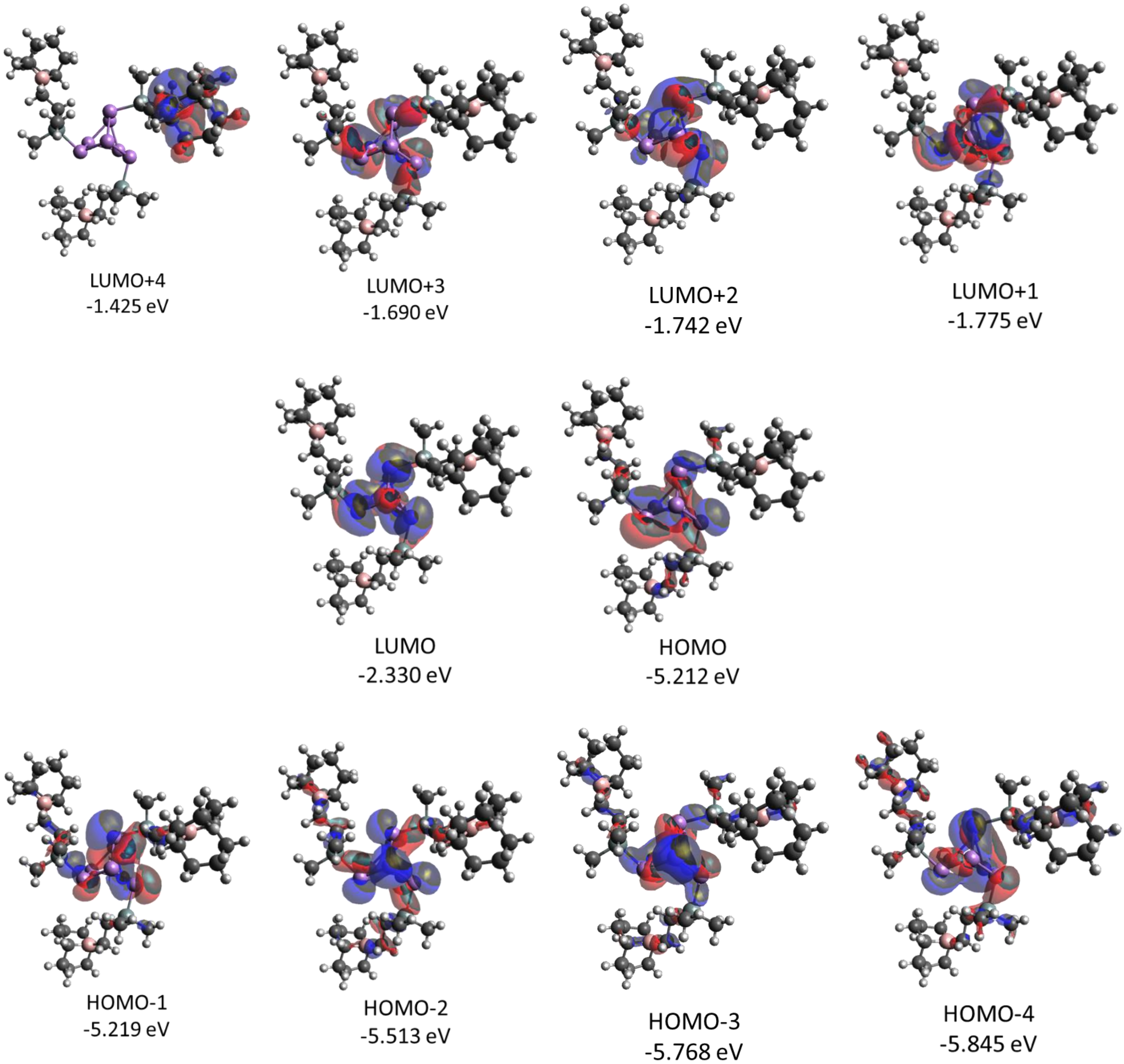

Figure S52. HOMO and LUMO Kohn-Sham images of **7**.

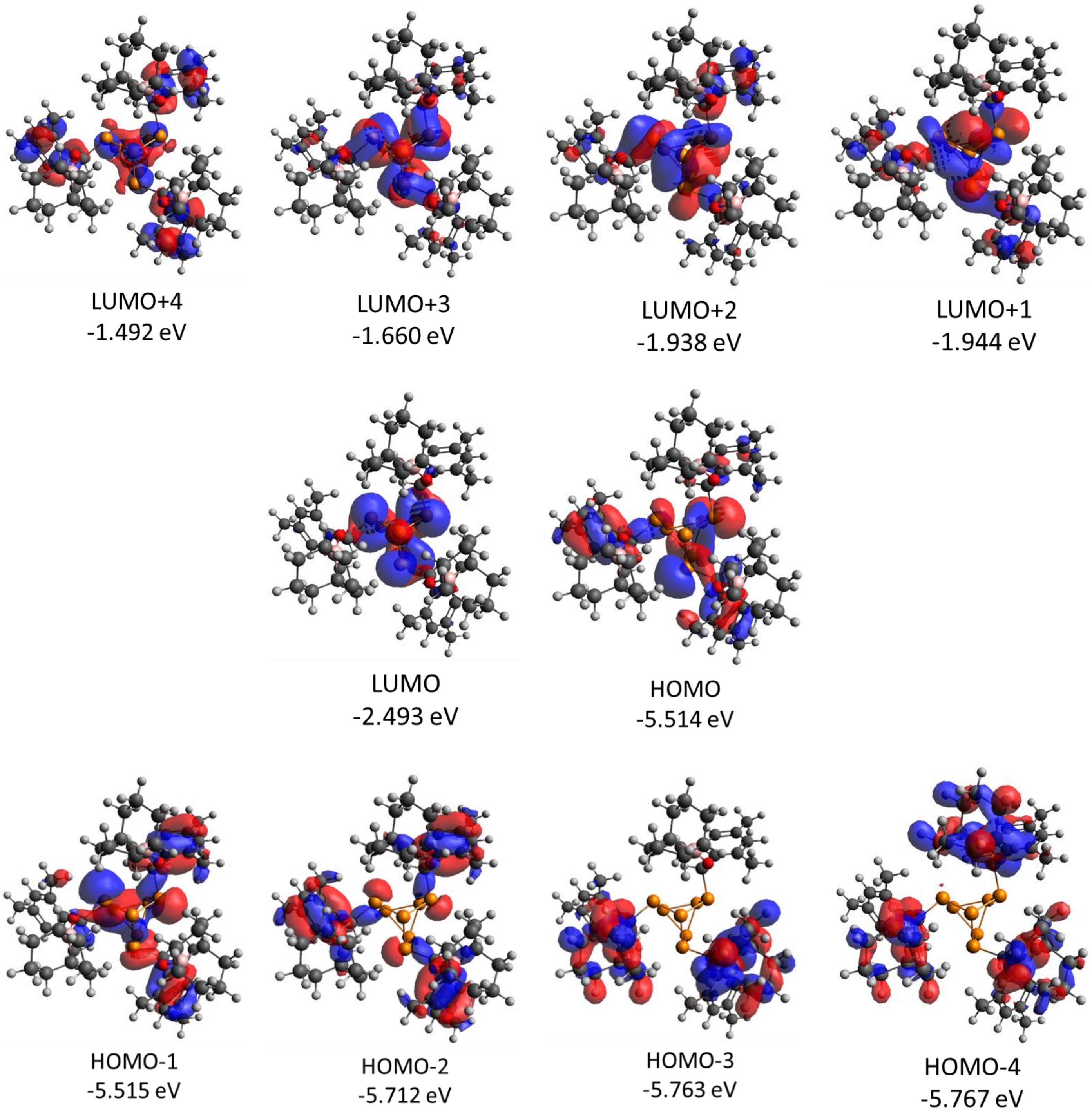

Figure S53. HOMO and LUMO Kohn-Sham images of **10**.

3.6. Solution Stability of Catalysts 3, 5, and 7

10 mg of catalysts **3**, **5**, and **7** were dissolved in 0.5 mL C_6D_6 and monitored by ^{1}H, ^{11}B, ^{29}Si{^{1}H} and ^{31}P NMR spectroscopy after 120 hours at room temperature (RT), 50 °C, and 110 °C. No evidence for decomposition was observed over this period confirming catalyst stability. For **3** we had difficulties in obtaining ^{29}Si{^{1}H} NMR spectroscopy after heating the sample for 120 hours at 110 °C.

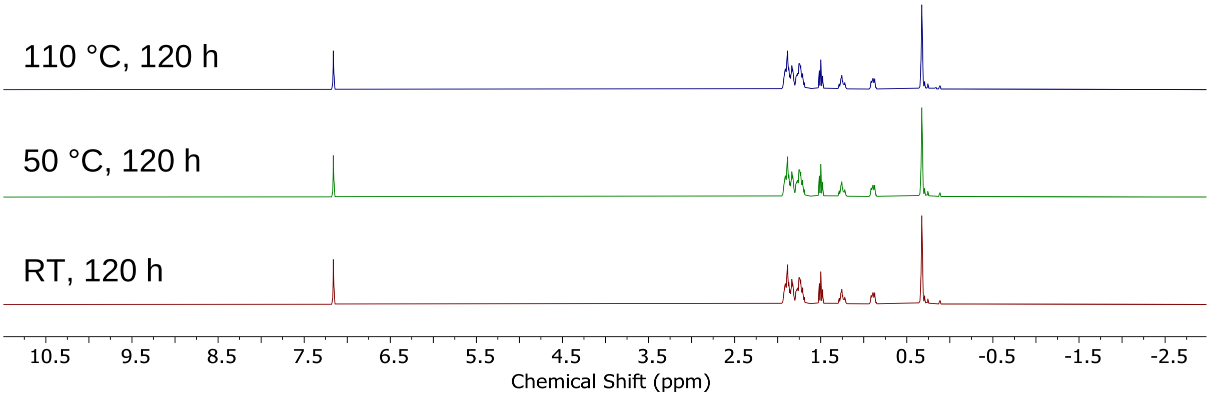

Figure S54. Stacked ^{1}H NMR spectrum (C_6D_6) of **3** after prolonged heating.

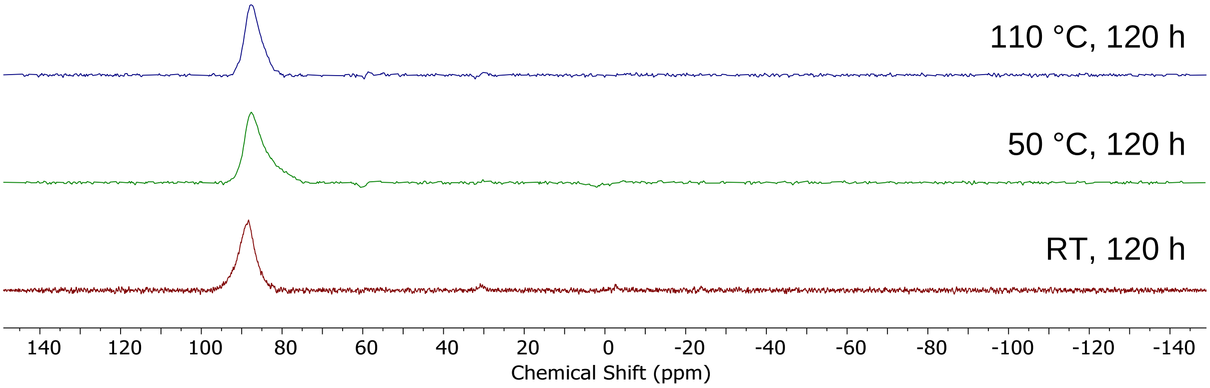

Figure S55. Stacked ^{11}B NMR spectrum (C_6D_6) of **3** after prolonged heating.

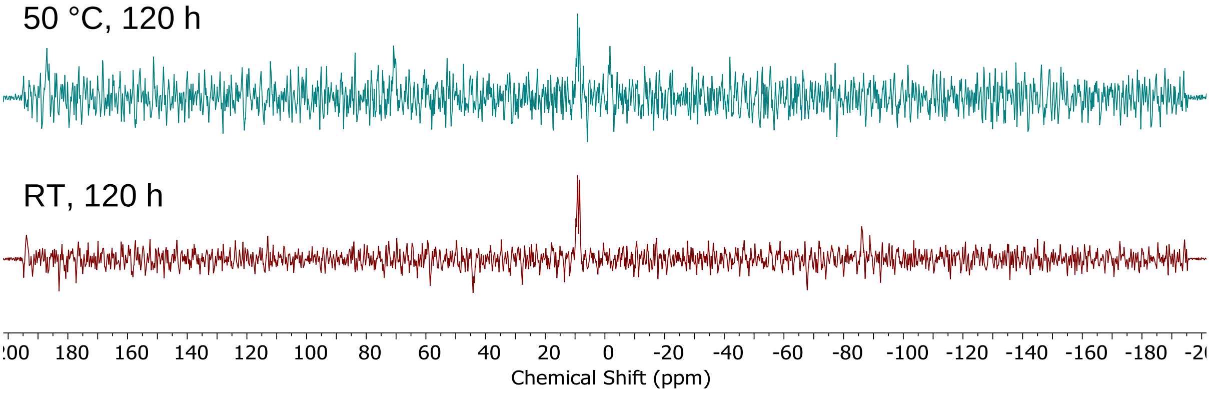

Figure S56. Stacked $^{29}Si\{^{1}H\}$ NMR spectrum (C_6D_6) of **3** after prolonged heating.

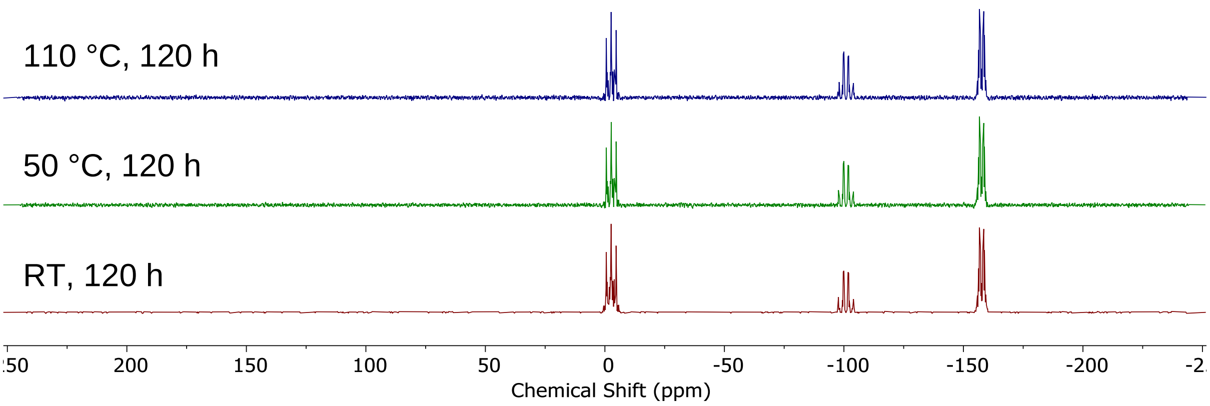

Figure S57. Stacked ^{31}P NMR spectrum (C_6D_6) of **3** after prolonged heating.

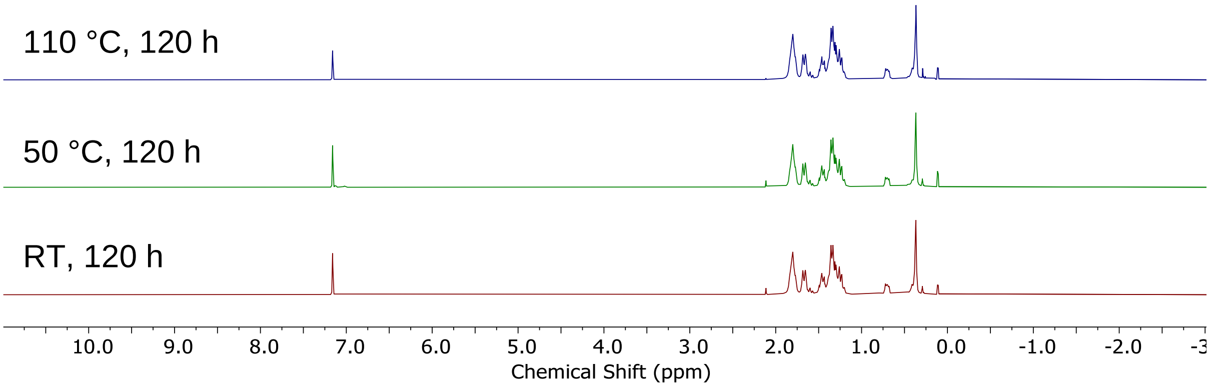

Figure S58. Stacked ^{1}H NMR spectrum (C_6D_6) of **5** after prolonged heating.

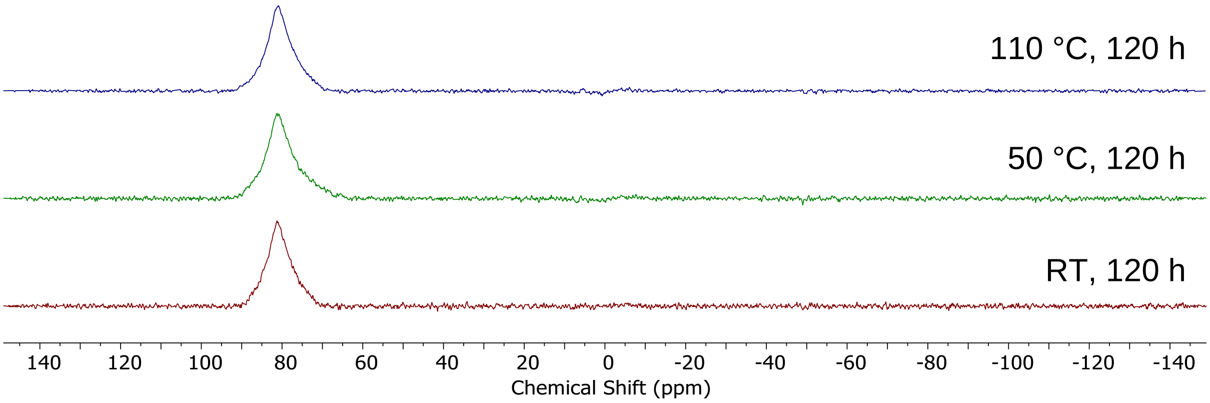

Figure S59. Stacked ^{11}B NMR spectrum (C_6D_6) of **5** after prolonged heating.

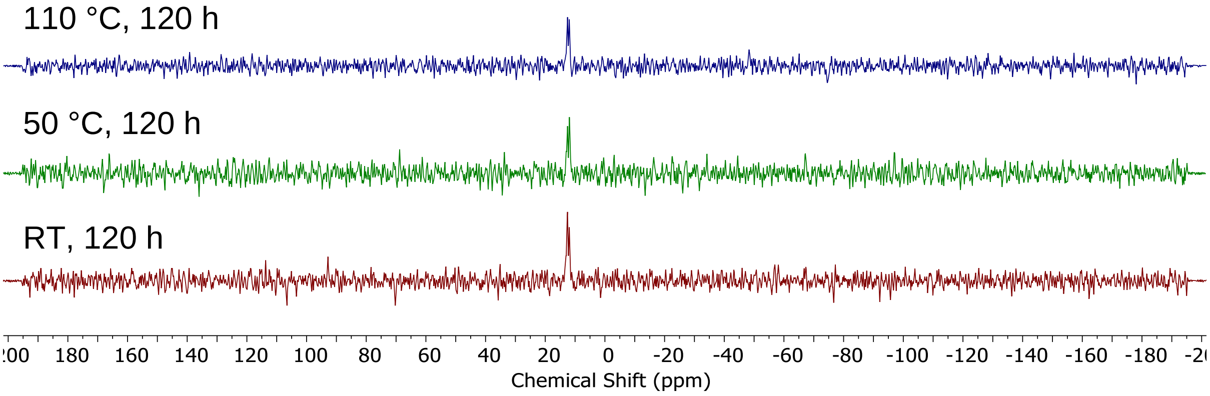

Figure S60. Stacked $^{29}Si\{^1H\}$ NMR spectrum (C_6D_6) of **5** after prolonged heating.

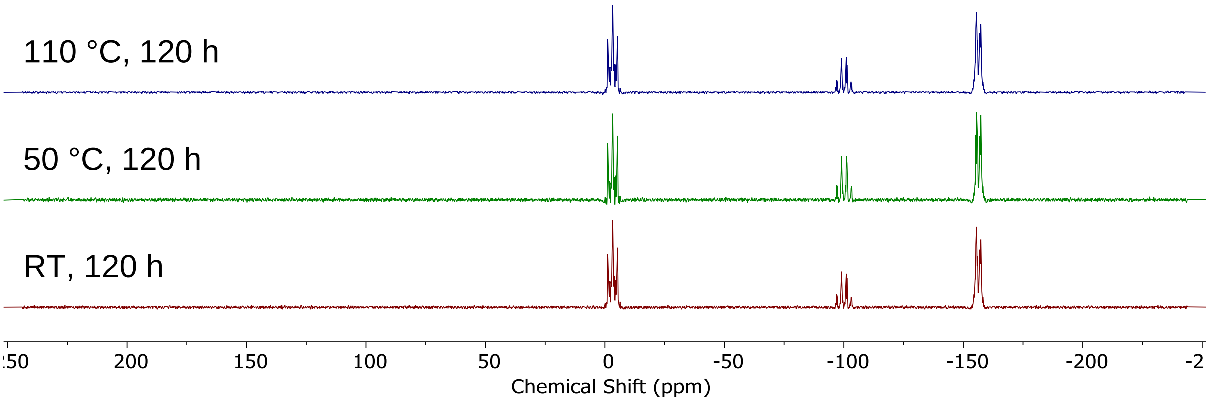

Figure S61. Stacked ^{31}P NMR spectrum (C_6D_6) of **5** after prolonged heating.

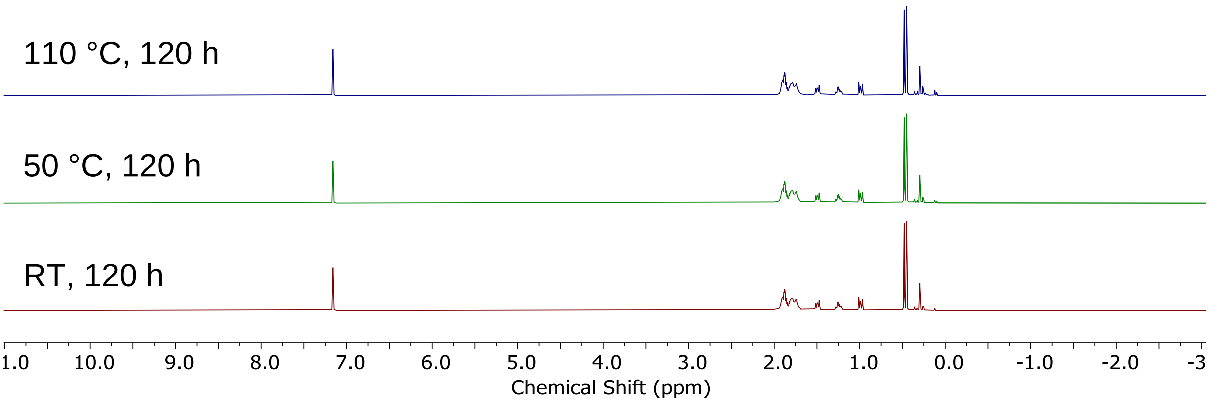

Figure S62. Stacked ^{1}H NMR spectrum (C_6D_6) of **7** after prolonged heating.

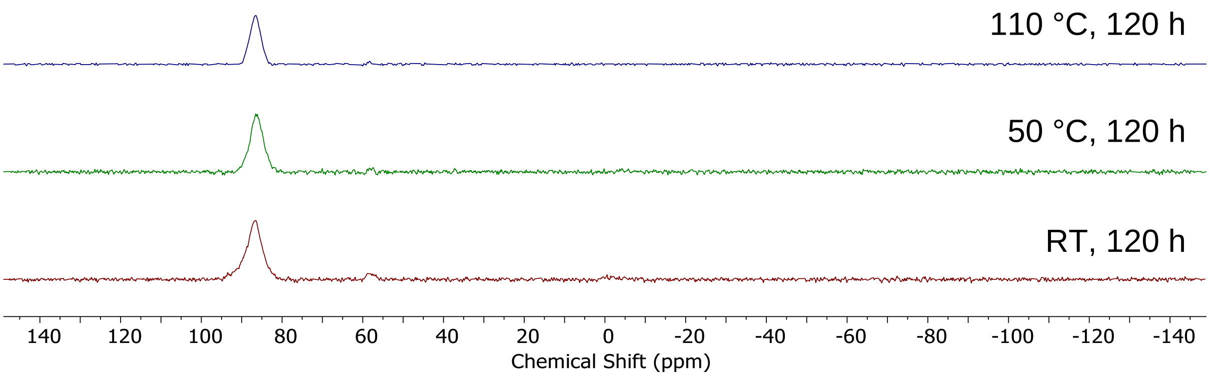

Figure S63. Stacked ^{11}B NMR spectrum (C_6D_6) of **7** after prolonged heating.

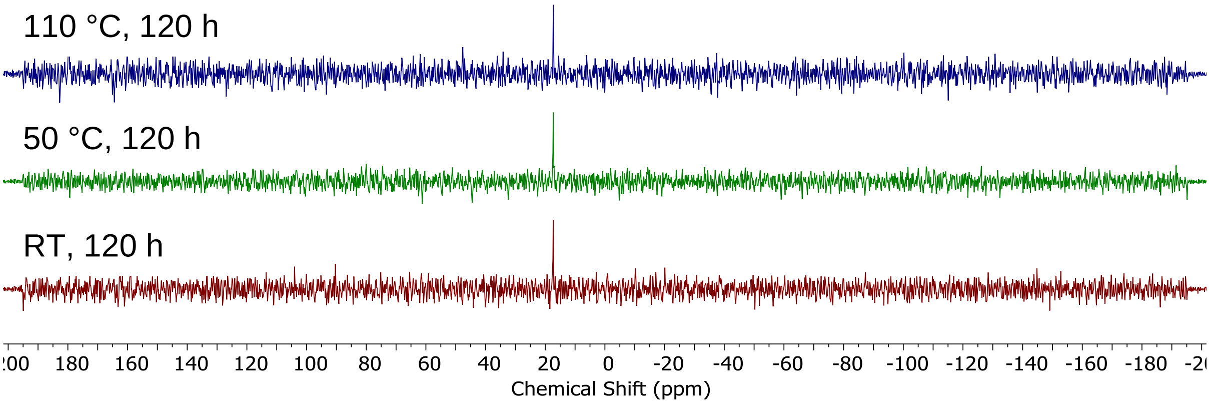

Figure S64. Stacked $^{29}Si\{^1H\}$ NMR spectrum (C_6D_6) of **7** after prolonged heating.

3.7. Retrohydroboration Examination of Catalysts 3, 5, and 7

The solution stability studies in section 3.6 may not identify retrohydroboration, cooling down to RT before collecting the NMR spectra may result in re-hydroboration of the vinyl-/ allyl-group. To study this, 10 mg of catalysts **3**, **5**, and **7** was dissolved in 0.5 mL toluene-d_8, the sample was heated in the spectrometer to 100 °C and ^1H, ^{11}B, and ^{31}P analysis recorded. The temperature was maintained at 100 °C with the sample in the spectrometer and analysis recorded again. No changes in the NMR spectra (most easily characterized by the alkenyl region in the ^1H NMR spectrum) was observed, consistent with no retrohydroboration in solution.

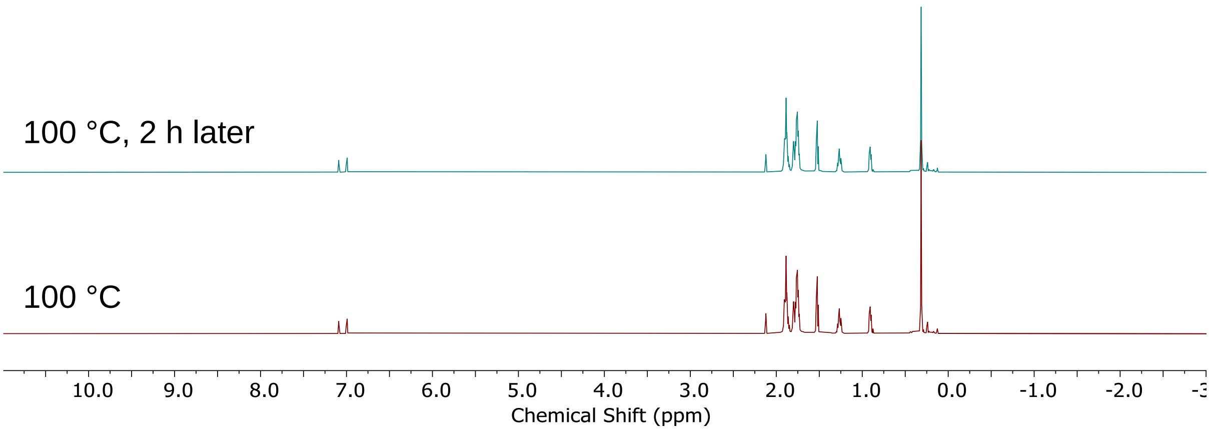

Figure S65. Stacked ^{1}H NMR spectrum (toluene-d_8) of **3** at 100 °C.

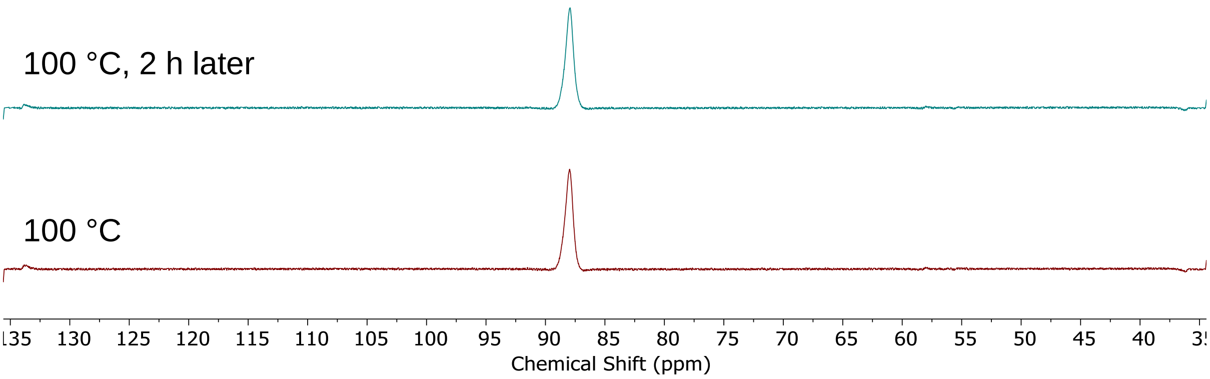

Figure S66. Stacked ^{11}B NMR spectrum (toluene-d_8) of **3** at 100 °C.

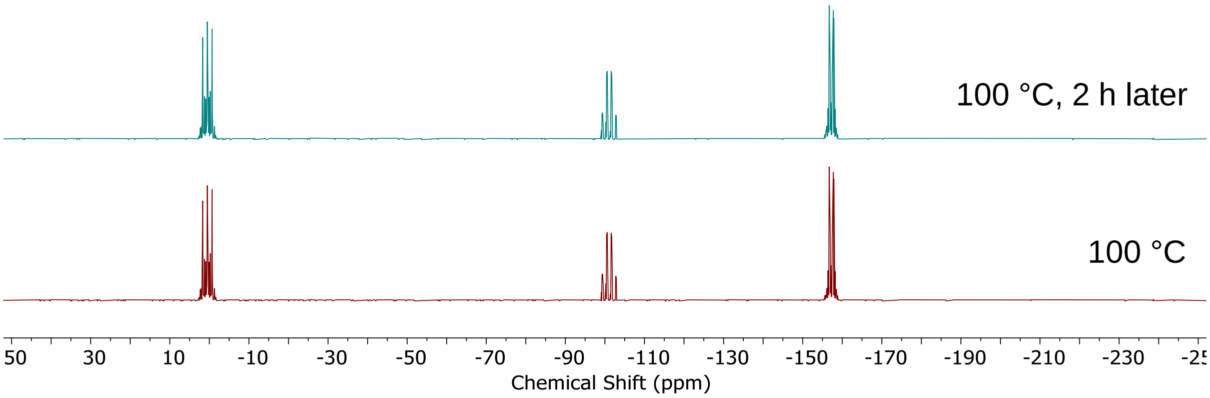

Figure S67. Stacked ^{31}P NMR spectrum (toluene-d_8) of **3** at 100 °C.

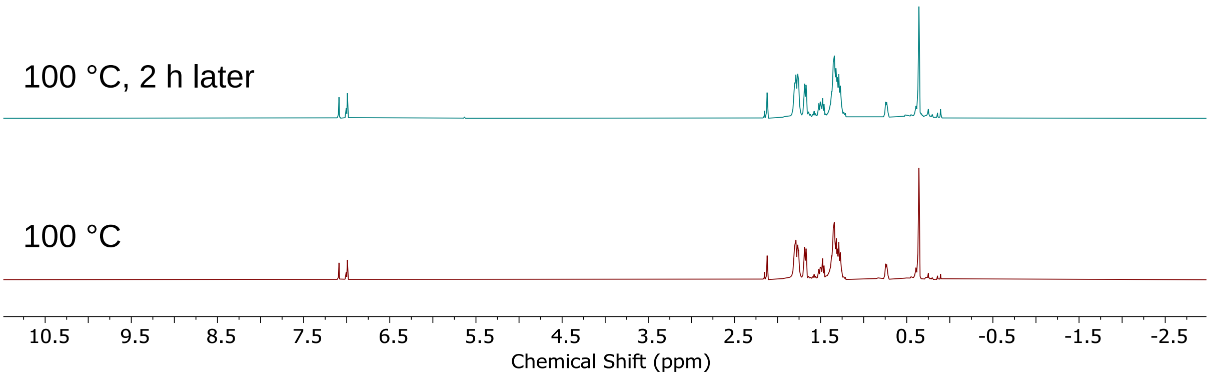

Figure S68. Stacked ^{1}H NMR spectrum (toluene-*d*$_8$) of **5** at 100 °C.

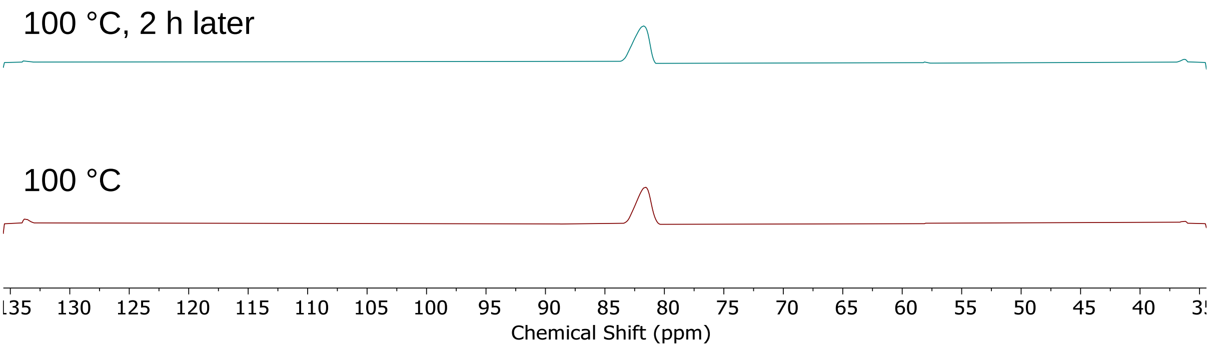

Figure S69. Stacked ^{11}B NMR spectrum (toluene-*d*$_8$) of **5** at 100 °C.

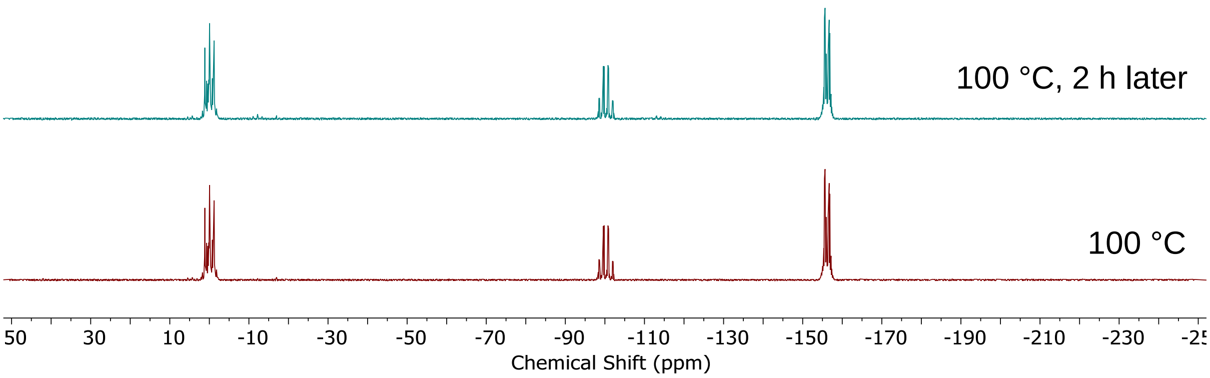

Figure S70. Stacked ^{31}P NMR spectrum (toluene-*d*$_8$) of **5** at 100 °C.

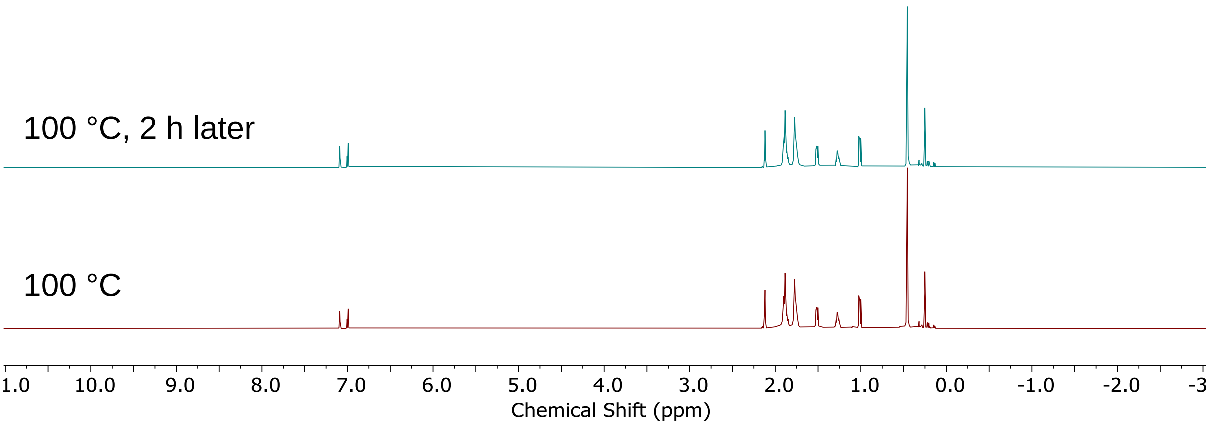

Figure S71. Stacked ^{1}H NMR spectrum (toluene-d_8) of **7** at 100 °C.

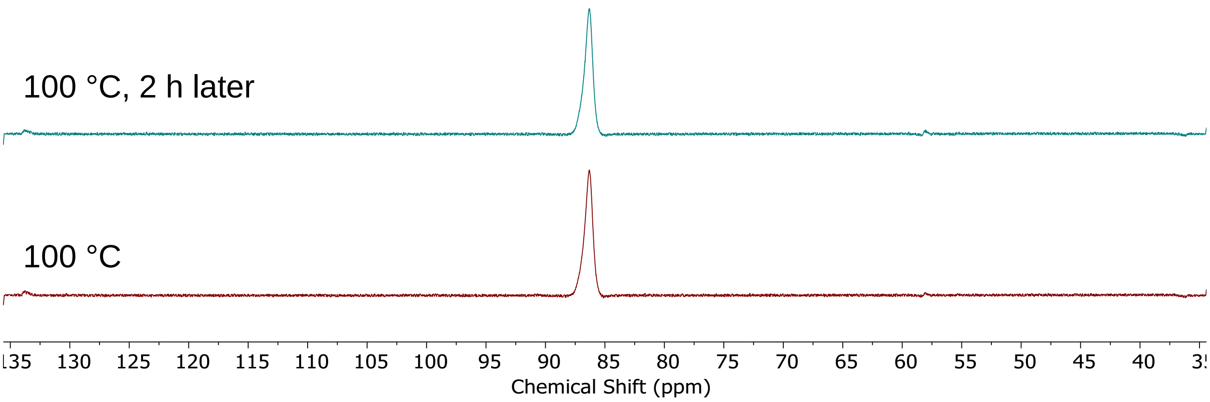

Figure S72. Stacked ^{11}B NMR spectrum (toluene-d_8) of **7** at 100 °C.

4. General Procedure for Hydroboration

Catalyst (5 mol%; **3** = 4.4 mg; **5** = 5.0 mg; **7** = 5.7 mg) was dissolved in 0.5 mL C_6D_6 and transferred to a J Young NMR tube. One (14.5 µL, 0.1 mmol) or two (29 µL, 0.2 mmol) equivalents of pinacol borane (HB(pin)) and substrate (0.1 mmol) were subsequently added *via* microsyringe. The reaction progress was monitored by ^{1}H and ^{11}B NMR spectroscopy and conversion was determined by ^{1}H NMR spectroscopy based on the ratio of target hydroborated product and toluene (10.7 µL, 0.1 mmol, 1 equivalent) as an internal standard. The NMR resonance used to determine conversion is underlined under the substrate characterisation data.

For the chloro-tethers **2** (3.9 mg) and **4** (3.7 mg) 15 mol% loading was used, and procedure followed as outlined above.

Integrations for aromatic signals in the ^{1}H NMR spectra were on occasion difficult to assign to crude compounds due to a mixture of hydroborated product, substrate, and toluene internal standard. All key assignments have been successfully made.

5. Comparative Hydroboration Characterisation Data

5.1. Carbodiimide – 11b – N,N'-diisopropyl-N-(4,4,5,5-tetramethyl-1,3,2-dioxaborolan-2- yl)formimidamide

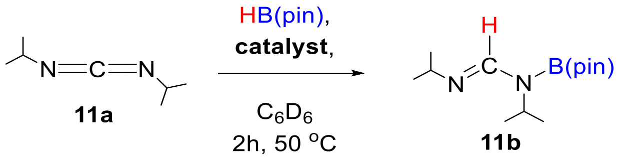

Table S5. Hydroboration of **11a** catalyzed by **1–5**, **7**, **8** in C_6D_6. Temperature = 50 °C. Time = 2 hours.

Catalyst	1[2]	2	3	4	5	7	8[2]
**Conversion/ %**	>99	>99	>99	>99	>99	>99	>99

*Annotated spectral data provided from the reaction mixture where **11a** is hydroborated with **5**.*

^{1}H NMR (400 MHz, 298 K, C_6D_6): δ = 8.26 (s, 1H, *H*C=N), 4.99 (sept, $^3J_{HH}$ = 6.73 Hz, 1H, C*H*(CH$_3$)$_2$), 3.32 (sept, $^3J_{HH}$ = 6.11 Hz, 1H, C*H*(CH$_3$)$_2$), 1.45 (d, $^3J_{HH}$ = 6.73 Hz, 6H, CH(C*H*$_3$)$_2$), 1.20 (d, $^3J_{HH}$ = 6.11 Hz, 6H, CH(C*H*$_3$)$_2$), 1.02 (s, 12H, B*pin*) ppm.

^{11}B NMR (128 MHz, 298 K, C_6D_6): δ = 25.29 (s) ppm.

Mass spectrometry (ESI): $C_{13}H_{27}BN_2O_2$+H ([M+H]$^+$); Calcd. = 255.2238, Found = 255.2240.

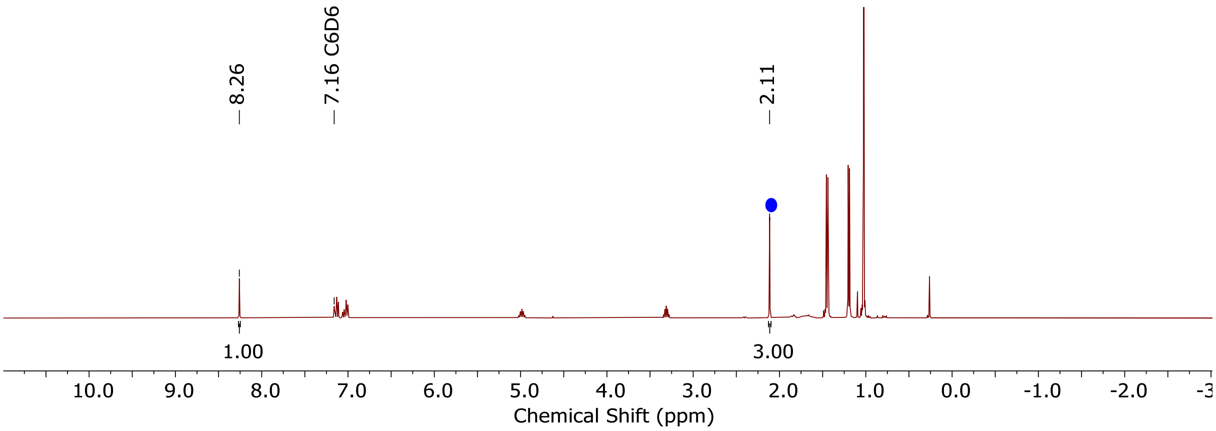

Figure S73. ^{1}H NMR spectrum (C_6D_6) of reaction mixture where **11a** is hydroborated with **2.** ● = toluene internal standard resonance.

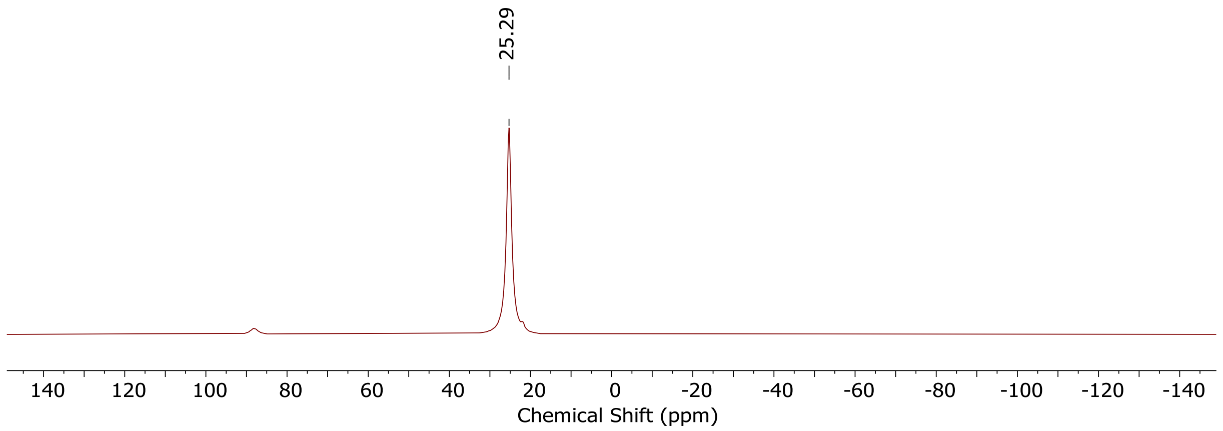

Figure S74. ^{11}B NMR spectrum (C_6D_6) of reaction mixture where **11a** is hydroborated with **2**.

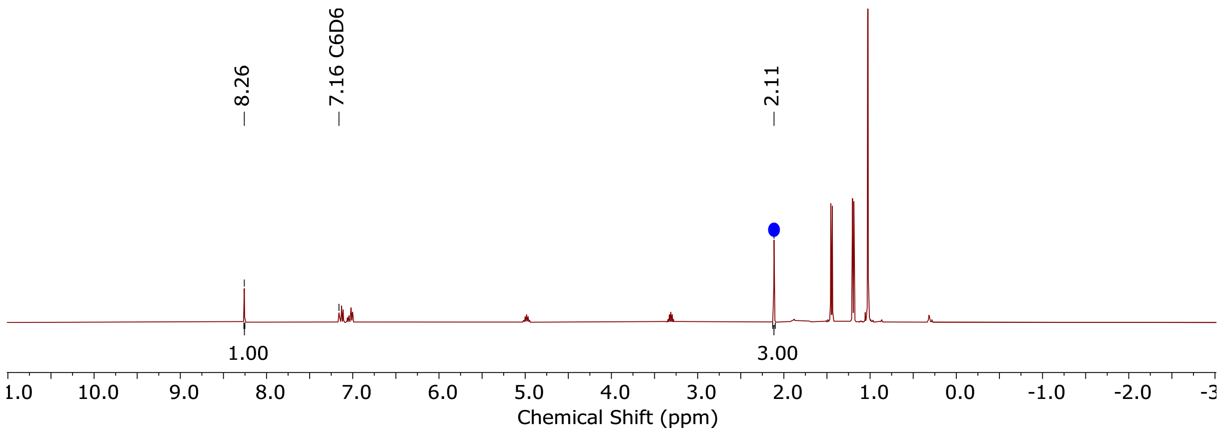

Figure S75. ^{1}H NMR spectrum (C_6D_6) of reaction mixture where **11a** is hydroborated with **3**. ● = toluene internal standard resonance.

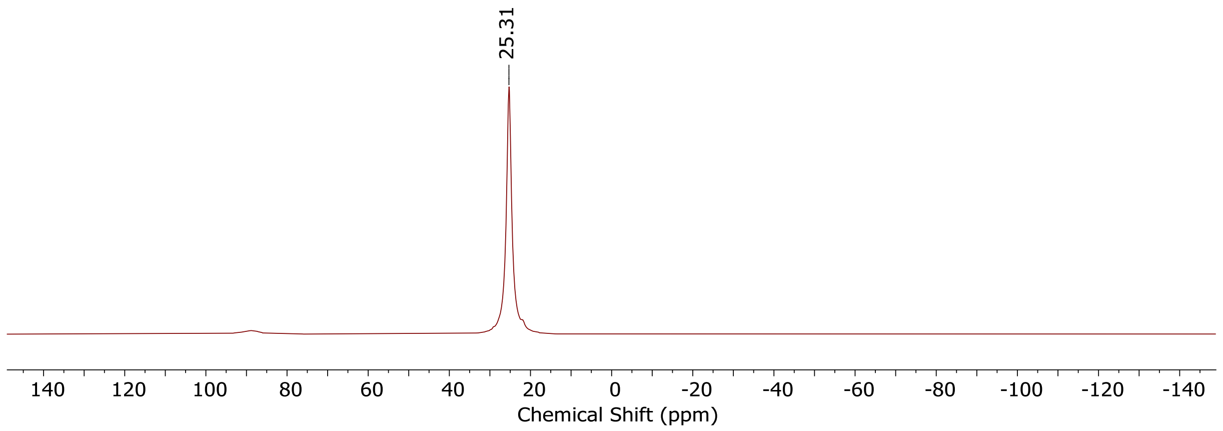

Figure S76. ^{11}B NMR spectrum (C_6D_6) of reaction mixture where **11a** is hydroborated with **3**.

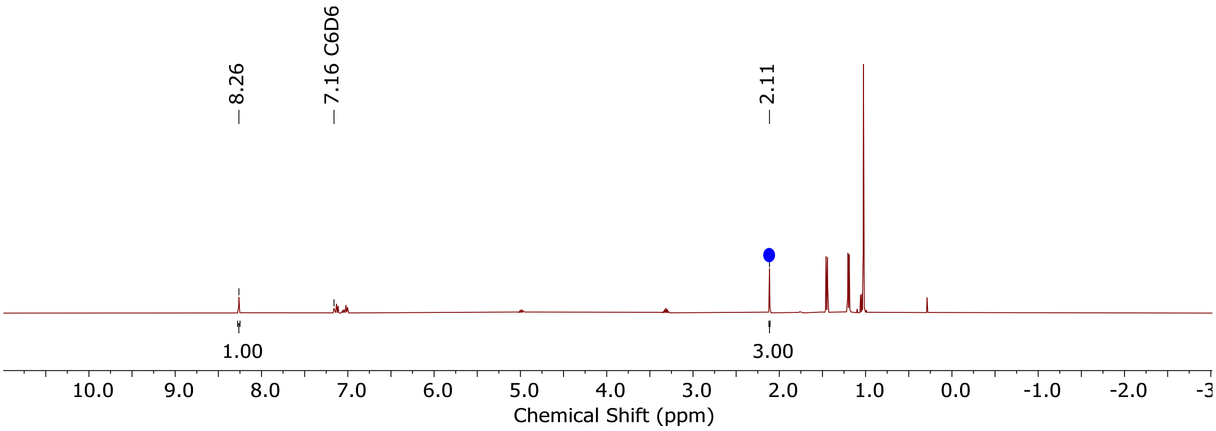

Figure S77. ^{1}H NMR spectrum (C_6D_6) of reaction mixture where **11a** is hydroborated with **4**. ● = toluene internal standard resonance.

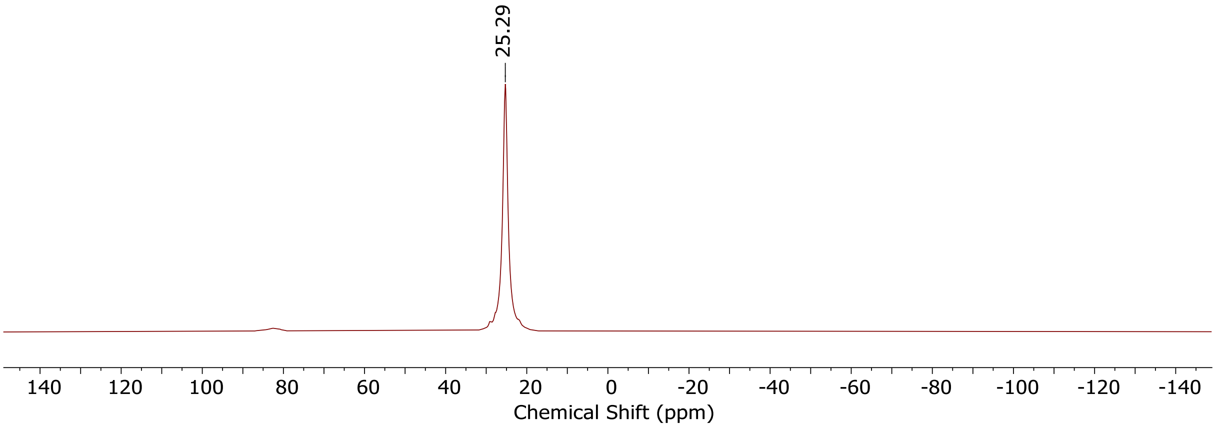

Figure S78. ^{11}B NMR spectrum (C_6D_6) of reaction mixture where **11a** is hydroborated with **4**.

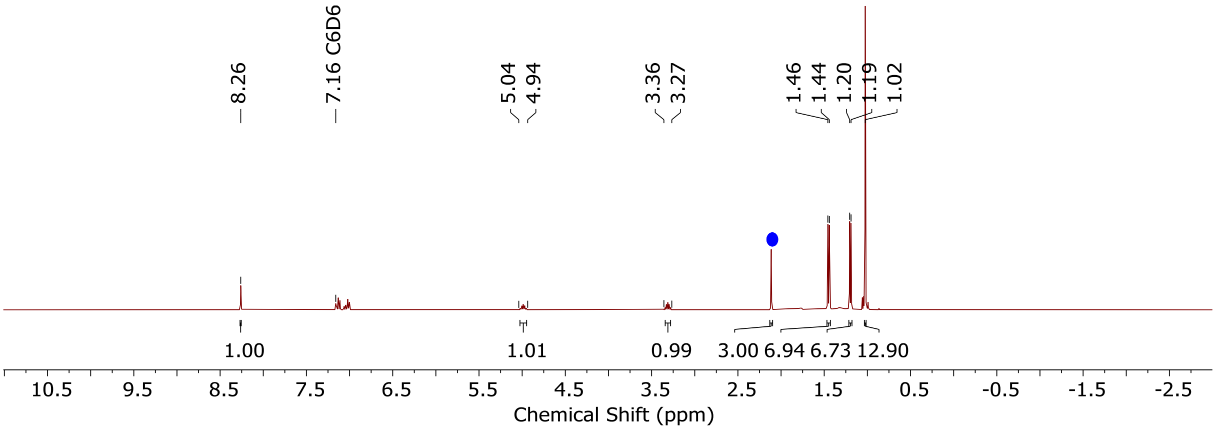

Figure S79. ^{1}H NMR spectrum (C_6D_6) of reaction mixture where **11a** is hydroborated with **5**. ● = toluene internal standard resonance.

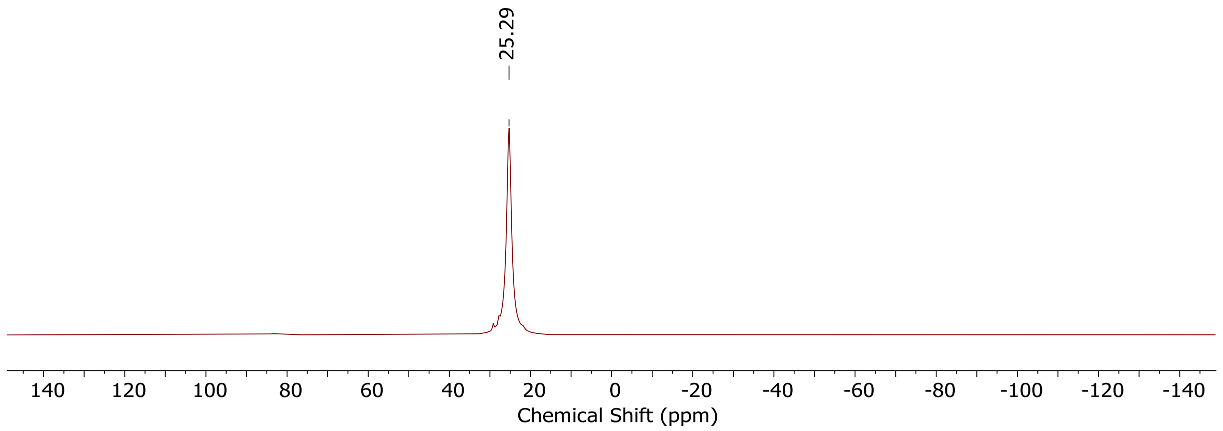

Figure S80. ^{11}B NMR spectrum (C_6D_6) of reaction mixture where **11a** is hydroborated with **5**.

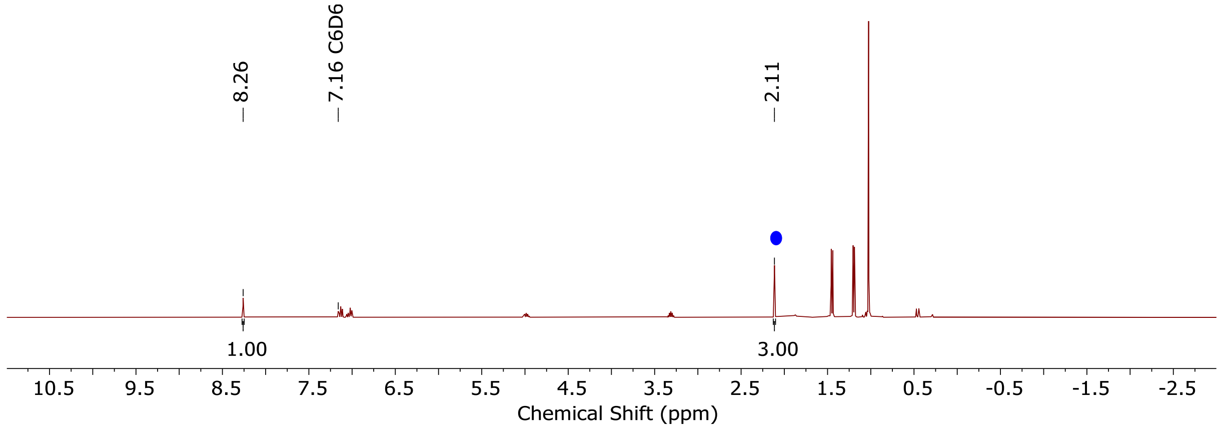

Figure S81. ^{1}H NMR spectrum (C_6D_6) of reaction mixture where **11a** is hydroborated with **7**. ● = toluene internal standard resonance.

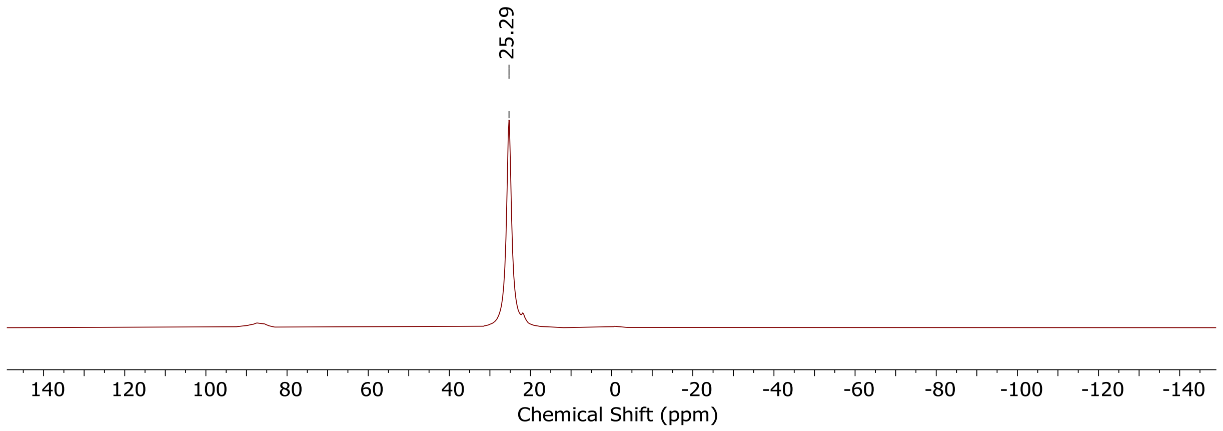

Figure S82. ^{11}B NMR spectrum (C_6D_6) of reaction mixture where **11a** is hydroborated with **7**.

5.2. Isocyanate – 12b – N-phenyl-N-(4,4,5,5-tetramethyl-1,3,2-dioxaborolan-2-yl)formamide

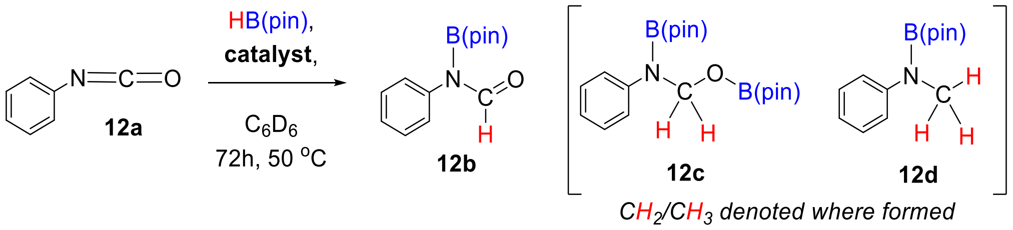

Table S6. Hydroboration of **12a** catalyzed by **1–5**, **7**, **8** in C_6D_6. Temperature = 50 °C. Time = 72 hours.

**Catalyst**	**1**[2]	**2**	**3**	**4**	**5**	**7**	**8**[2]
**Conversion/ %**	43[a]	27[b]	33[c]	77	68	32[d]	31[e]

[a] Additionally 8% bis-hydroboration (**12c**) and 3% hydrodeoxygenation (**12d**) observed. [b] Additionally 9% bis-hydroboration (**12c**) and 2% hydrodeoxygenation (**12d**) observed. [c] Additionally 6% bis-hydroboration (**12c**) observed. [d] Additionally 6% bis-hydroboration (**12c**) and 1% hydrodeoxygenation (**12d**) observed [e] Additionally 8% bis-hydroboration (**12c**) and 3% hydrodeoxygenation (**12d**) observed.

*Annotated spectral data provided from the reaction mixture where **12a** is hydroborated with **5**.*

^{1}H NMR (400 MHz, 298 K, C_6D_6): δ = 9.17 (s, 1H(68%) observed integration 0.68, *CH*), 7.21–7.23 (m, 2H, *Ar*), 7.09–7.14 (m, 3H, *Ar*), 0.92 (s, 12H, B*pin*) ppm. *Note overlapping toluene Ar resonances with Ar resonances of **11b**.*

^{11}B NMR (128 MHz, 298 K, C_6D_6): δ = 25.69 (s) ppm.

Mass spectrometry (ESI): $C_{13}H_{18}BNO_3$ ($[M]^+$); Calcd. = 247.1379, Found = 247.1375.

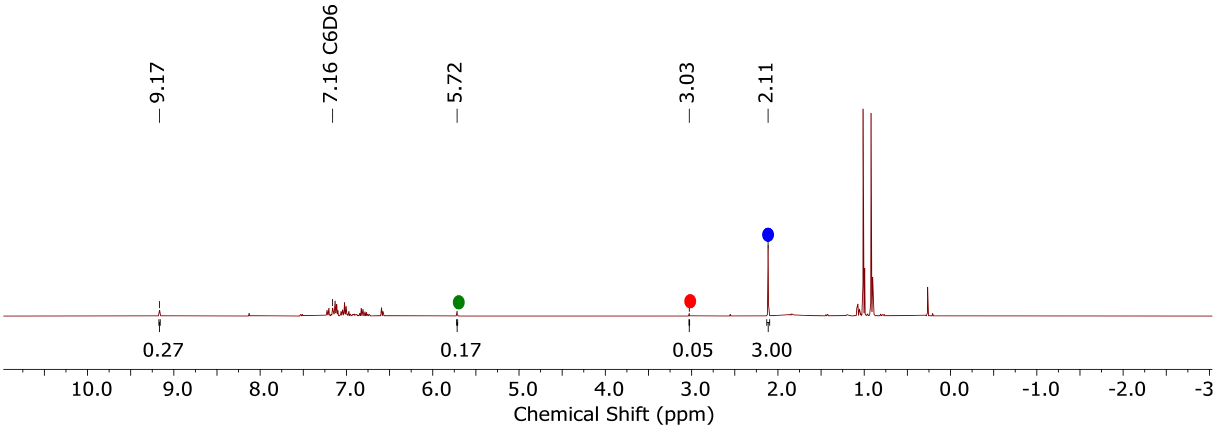

Figure S83. ^{1}H NMR spectrum (C_6D_6) of reaction mixture where **12a** is hydroborated with **2**. ● = toluene internal standard resonance. ● = **12c** C*H*$_2$. ● = **12d** C*H*$_3$.

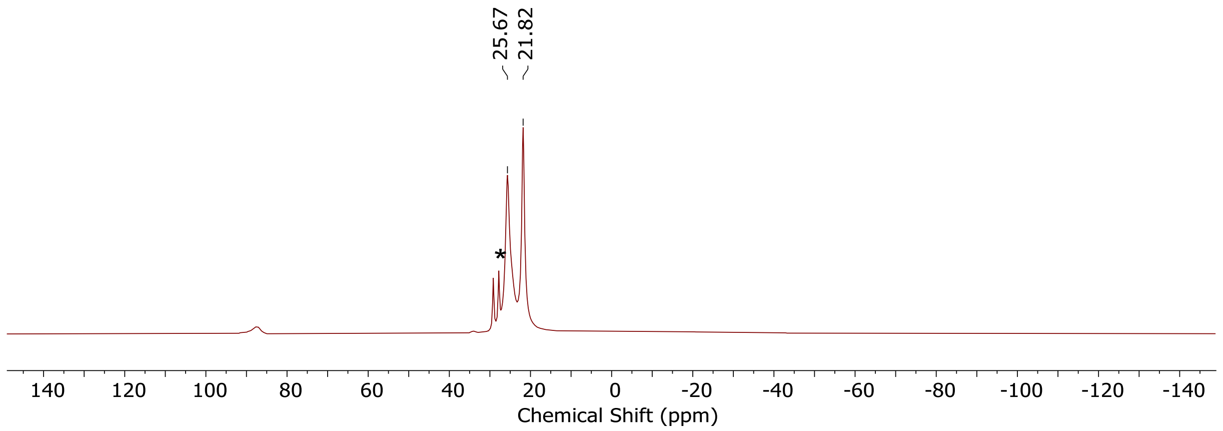

Figure S84. ^{11}B NMR spectrum (C_6D_6) of reaction mixture where **12a** is hydroborated with **2**. * denotes unreacted HB(pin).

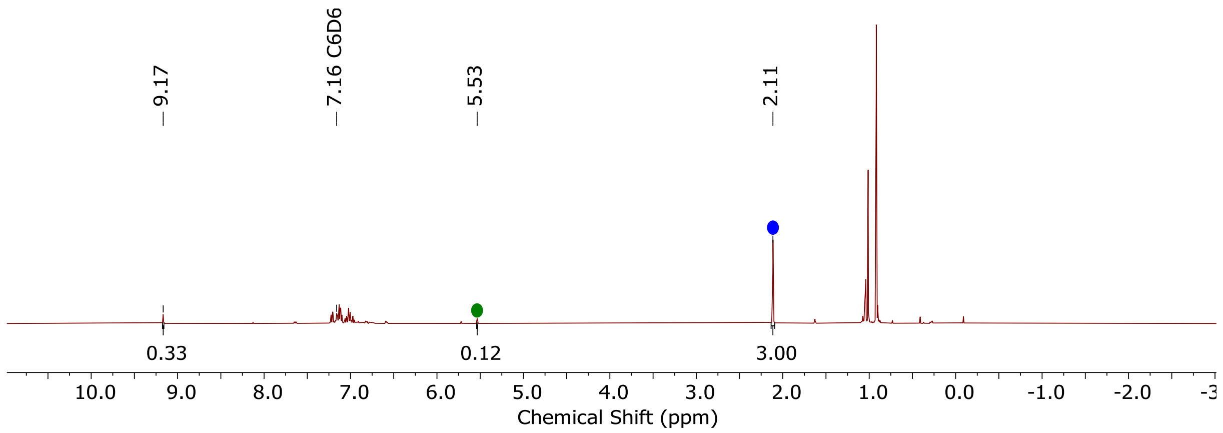

Figure S85. ^{1}H NMR spectrum (C_6D_6) of reaction mixture where **12a** is hydroborated with **3**. ● = toluene internal standard resonance. ● = **12c** CH_2.

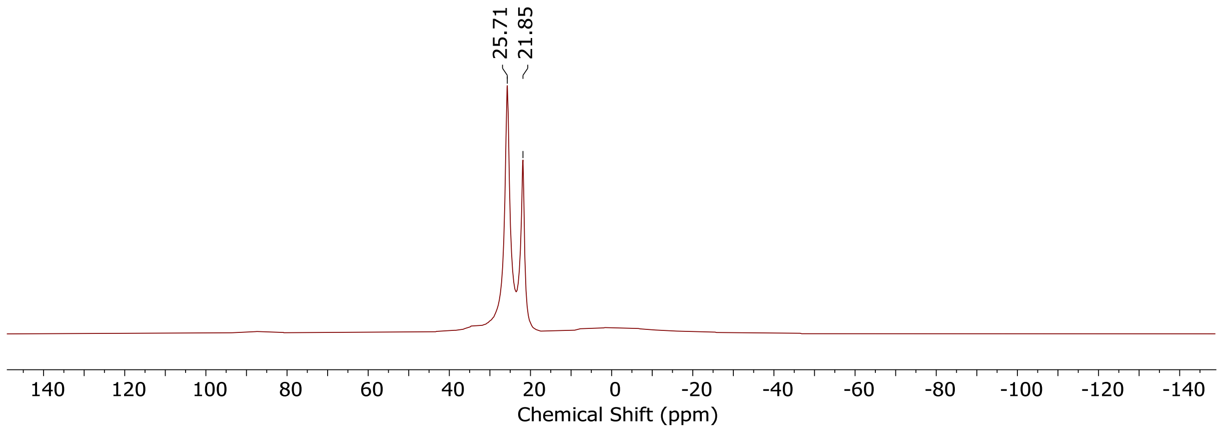

Figure S86. ^{11}B NMR spectrum (C_6D_6) of reaction mixture where **12a** is hydroborated with **3**.

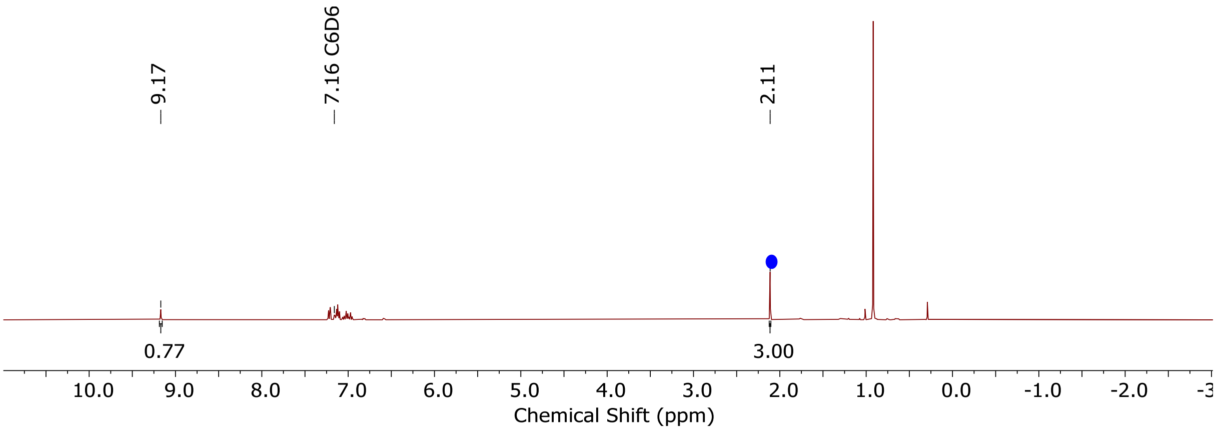

Figure S87. ^{1}H NMR spectrum (C_6D_6) of reaction mixture where **12a** is hydroborated with **4**. ● = toluene internal standard resonance.

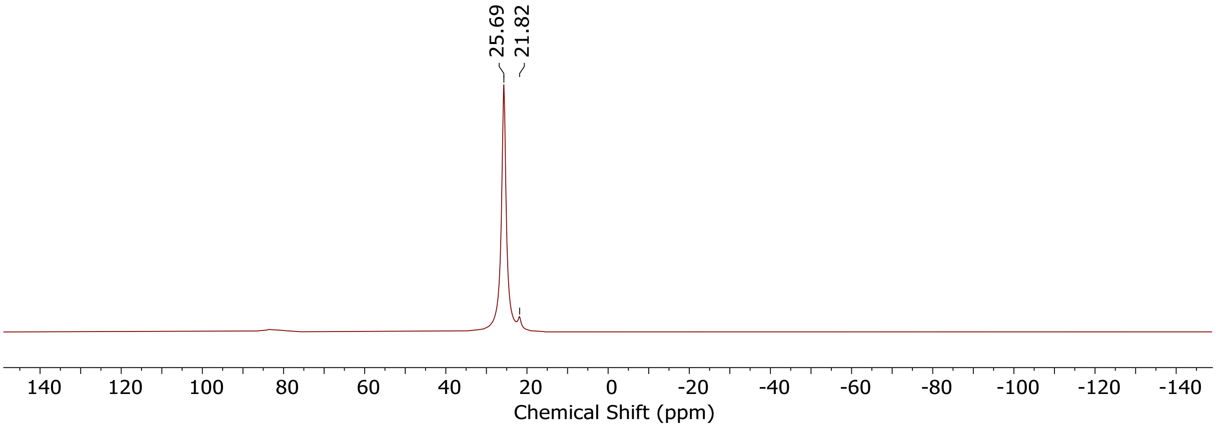

Figure S88. ^{11}B NMR spectrum (C_6D_6) of reaction mixture where **12a** is hydroborated with **4**.

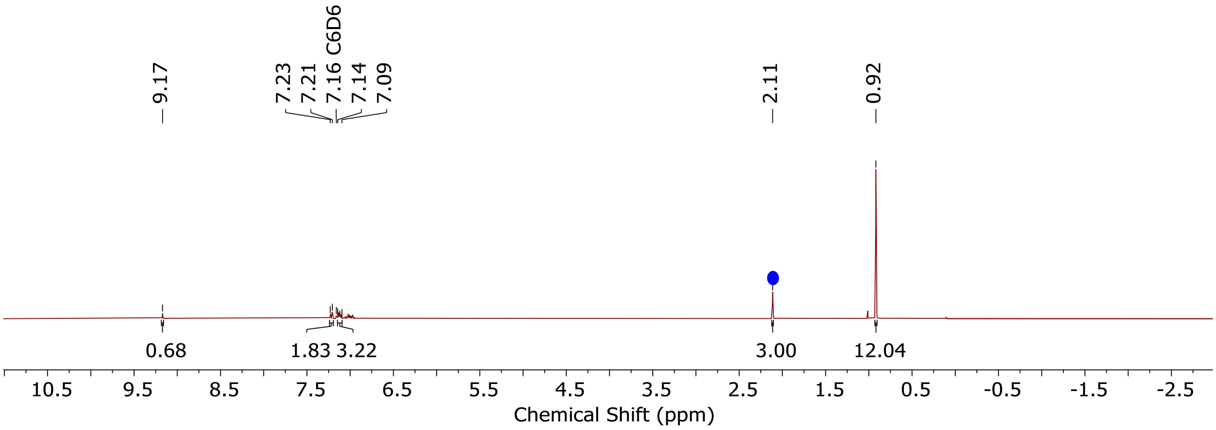

Figure S89. ^{1}H NMR spectrum (C_6D_6) of reaction mixture where **12a** is hydroborated with **5**. ● = toluene internal standard resonance.

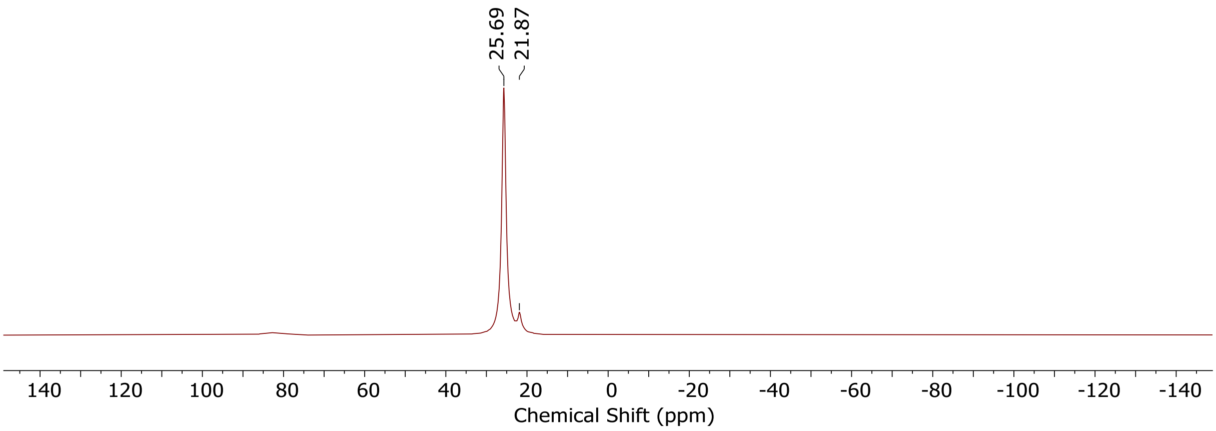

Figure S90. ^{11}B NMR spectrum (C_6D_6) of reaction mixture where **12a** is hydroborated with **5**.

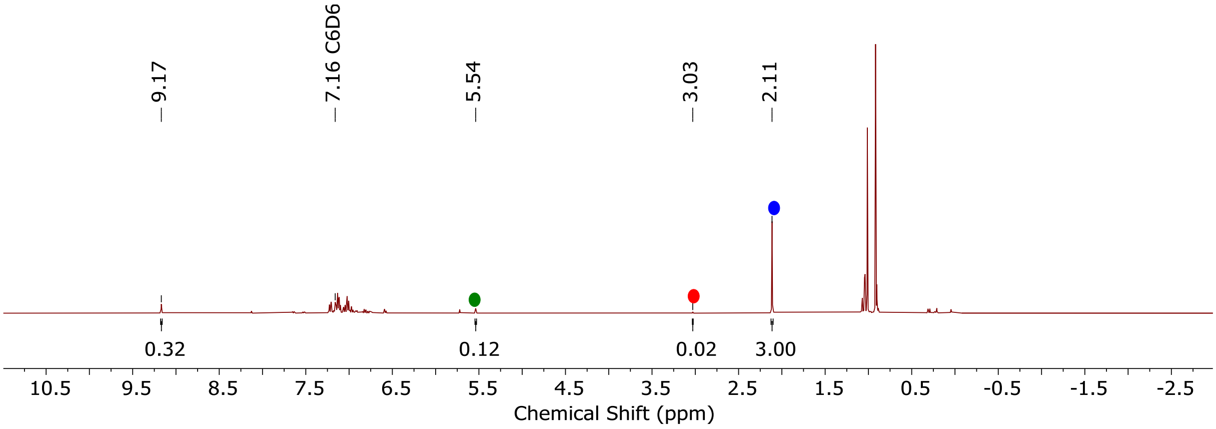

Figure S91. ^{1}H NMR spectrum (C_6D_6) of reaction mixture where **12a** is hydroborated with **7**. ● = toluene internal standard resonance. ● = **12c** CH_2. ● = **12d** CH_3.

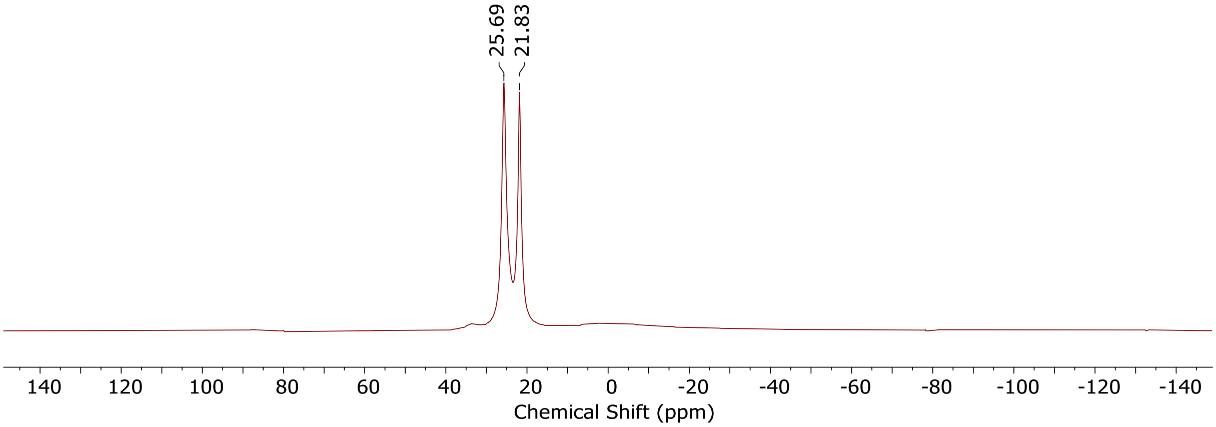

Figure S92. ^{11}B NMR spectrum (C_6D_6) of reaction mixture where **12a** is hydroborated with **7**.

5.3. Ketone – 13b – 4,4,5,5-tetramethyl-2-(1-phenylethoxy)-1,3,2-dioxaborolane

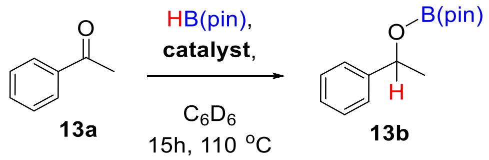

Table S7. Hydroboration of **13a** catalyzed by **1–5**, **7**, **8** in C_6D_6. Temperature = 110 °C. Time = 15 hours.

Catalyst	1[2]	2	3	4	5	7	8[2]
Conversion/ %	91	89	93	20	39	49	96

Note – Conversions were either determined from the C*H* or C*H*$_3$ resonance.

*Annotated spectral data provided from the reaction mixture where **13a** is hydroborated with **3**.*

^{1}H NMR (400 MHz, 298 K, C_6D_6): δ = 7.37 (d, $^3J_{HH}$ = 7.97 Hz, 2H, *Ar*), 7.00–7.07 (m, 3H, *Ar*), 5.42 (q, $^3J_{HH}$ = 6.53 Hz, 1H(80%) observed integration, C*H*), 5.20 (q, $^3J_{HH}$ = 6.53 Hz, 1H(13%) observed integration 0.13, C*H* **13b** coordinated to **3**), 1.46 (d, $^3J_{HH}$ = 6.53 Hz, 3H, C*H*$_3$), 1.03 (s, 6H, B*pin*), 1.00 (s, 6H, B*pin*) ppm.

^{11}B NMR (128 MHz, 298 K, C_6D_6): δ = 22.6 (s) ppm.

Mass spectrometry (ESI): *Despite multiple efforts, accurate mass spectrometry analysis was unsuccessful.*

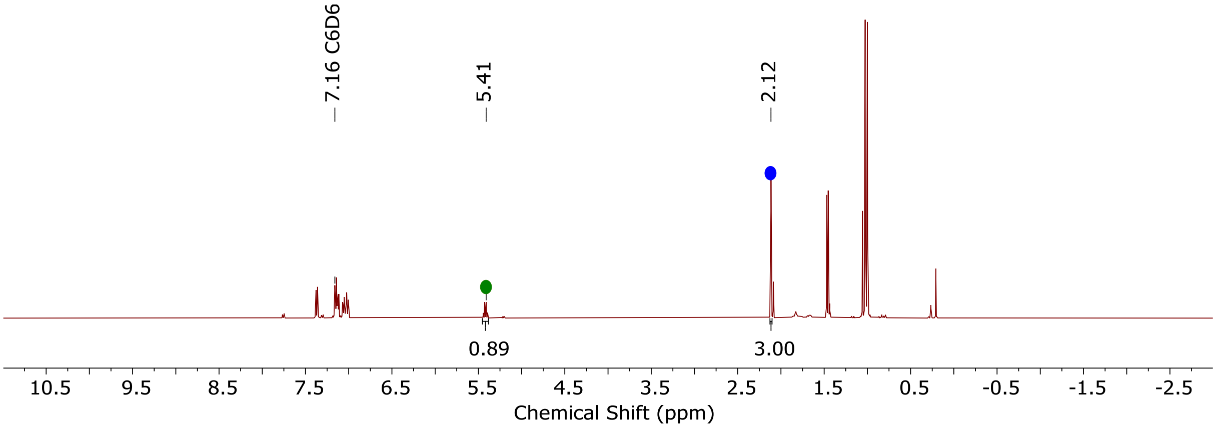

Figure S93. ^{1}H NMR spectrum (C_6D_6) of reaction mixture where **13a** is hydroborated with **2**. ● = toluene internal standard resonance. ● = "free" **13b**.

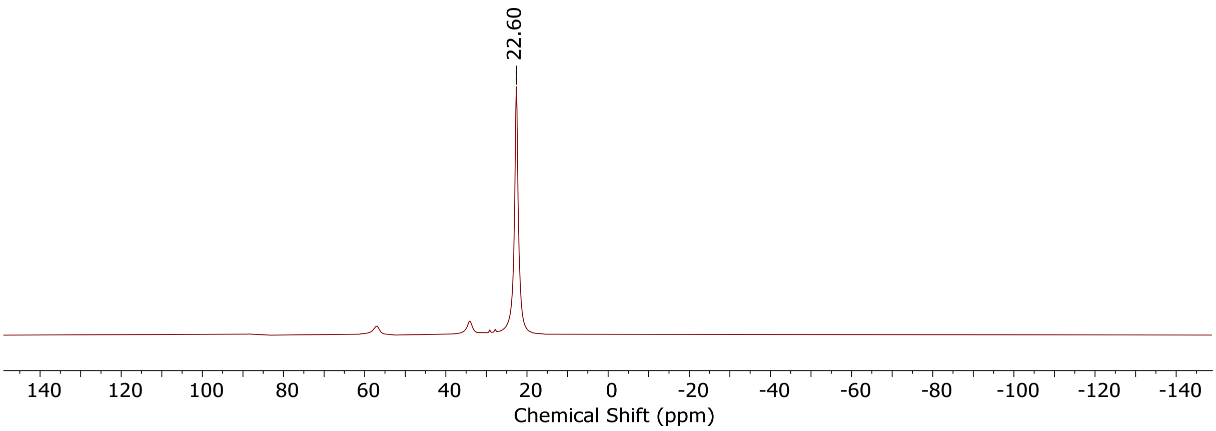

Figure S94. ^{11}B NMR spectrum (C_6D_6) of reaction mixture where **13a** is hydroborated with **2**.

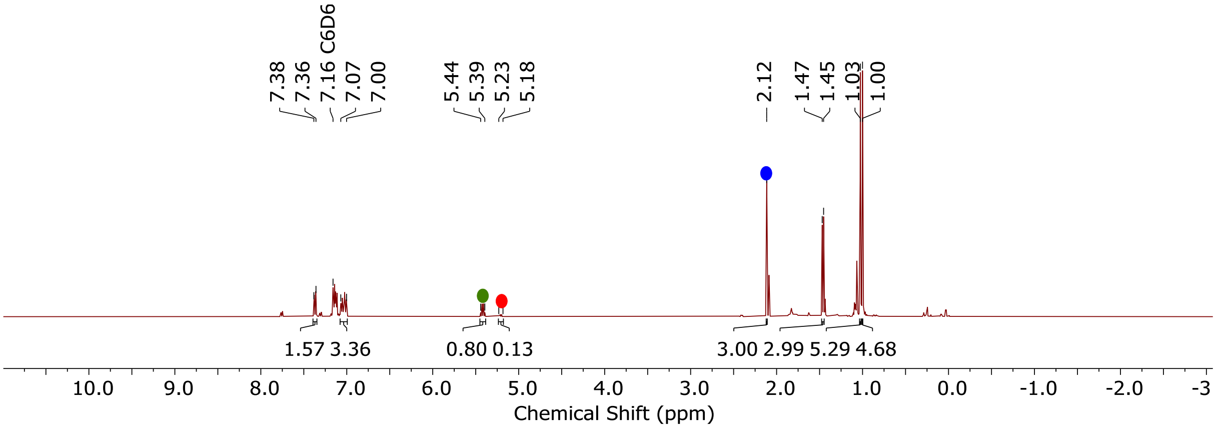

Figure S95. ^{1}H NMR spectrum (C_6D_6) of reaction mixture where **13a** is hydroborated with **3**. ● = toluene internal standard resonance. ● = "free" **13b**. ● = **13b** coordinated to **3**.

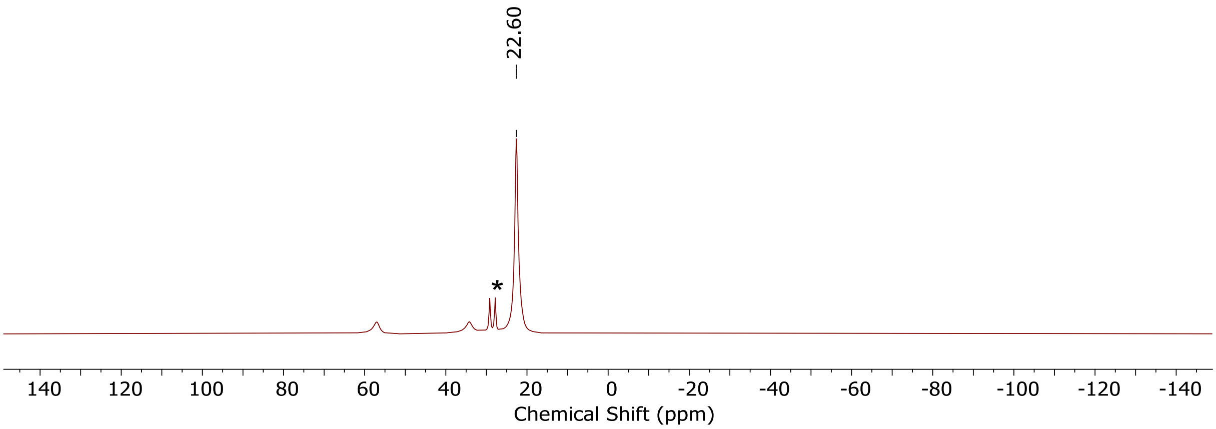

Figure S96. ^{11}B NMR spectrum (C_6D_6) of reaction mixture where **13a** is hydroborated with **3**. * denotes unreacted HB(pin).

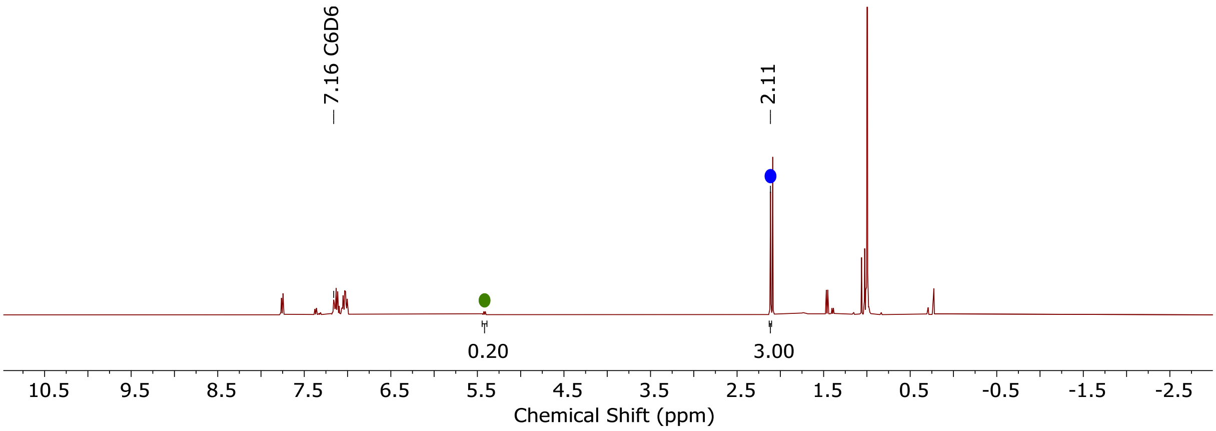

Figure S97. ^{1}H NMR spectrum (C_6D_6) of reaction mixture where **13a** is hydroborated with **4**. ● = toluene internal standard resonance. ● = “free” **13b**.

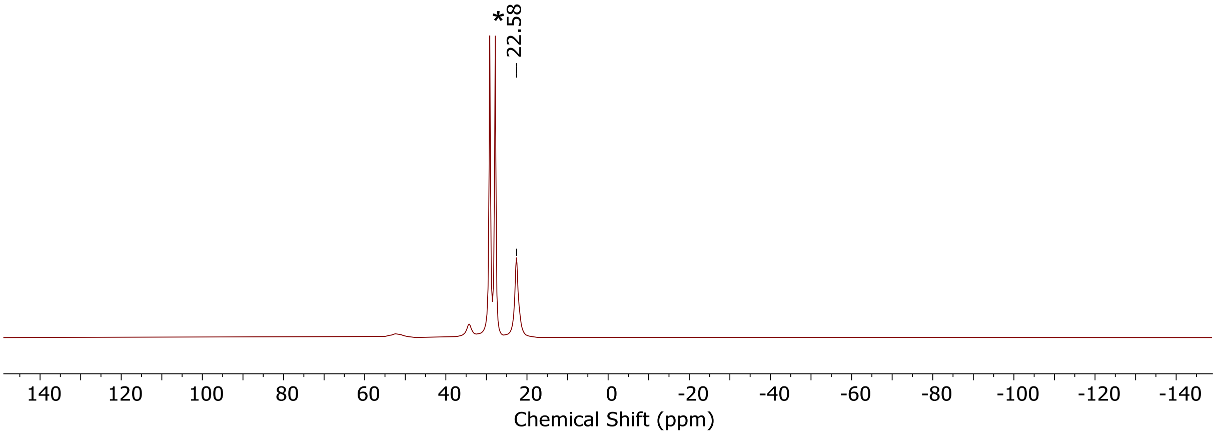

Figure S98. ^{11}B NMR spectrum (C_6D_6) of reaction mixture where **13a** is hydroborated with **4**. * denotes unreacted HB(pin).

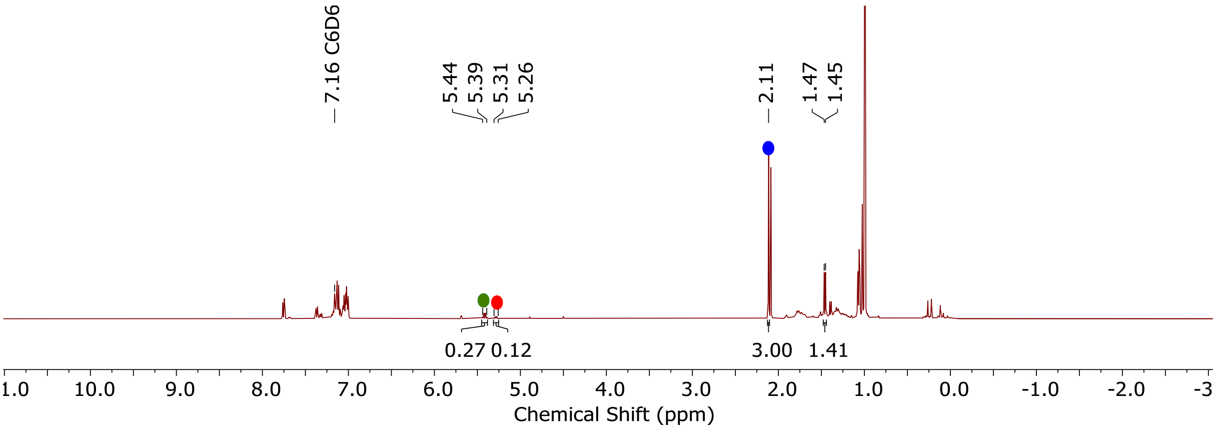

Figure S99. ^{1}H NMR spectrum (C_6D_6) of reaction mixture where **13a** is hydroborated with **5**. ● = toluene internal standard resonance. ● = “free” **13b**. ● = **13b** coordinated to **5**.

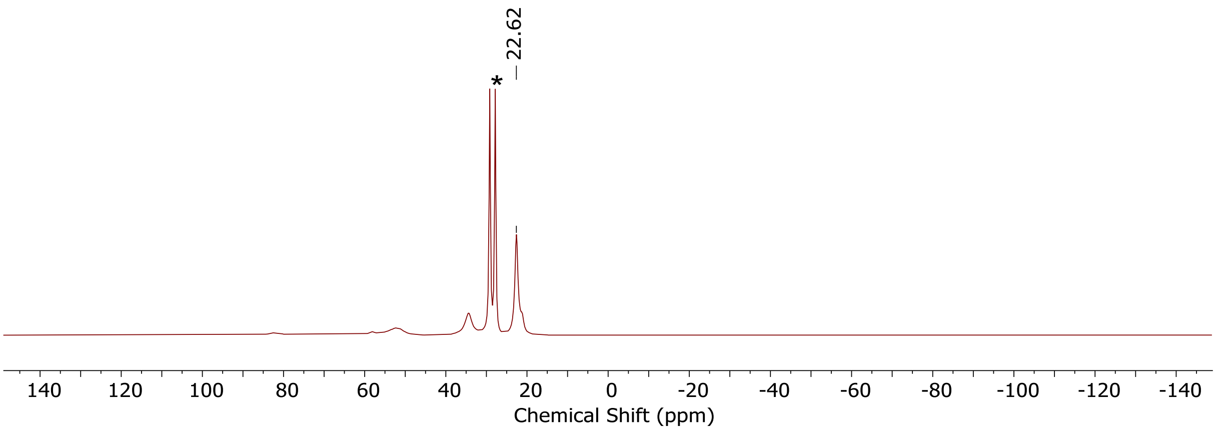

Figure S100. ^{11}B NMR spectrum (C_6D_6) of reaction mixture where **13a** is hydroborated with **5**. * denotes unreacted HB(pin).

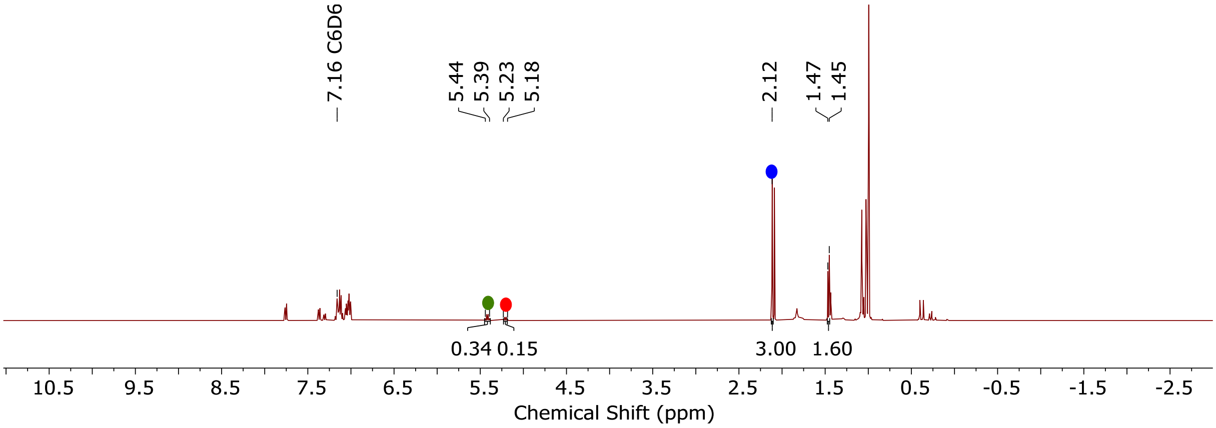

Figure S101. ^{1}H NMR spectrum (C_6D_6) of reaction mixture where **13a** is hydroborated with **7**. ● = toluene internal standard resonance. ● = "free" **13b**. ● = **13b** coordinated to **7**.

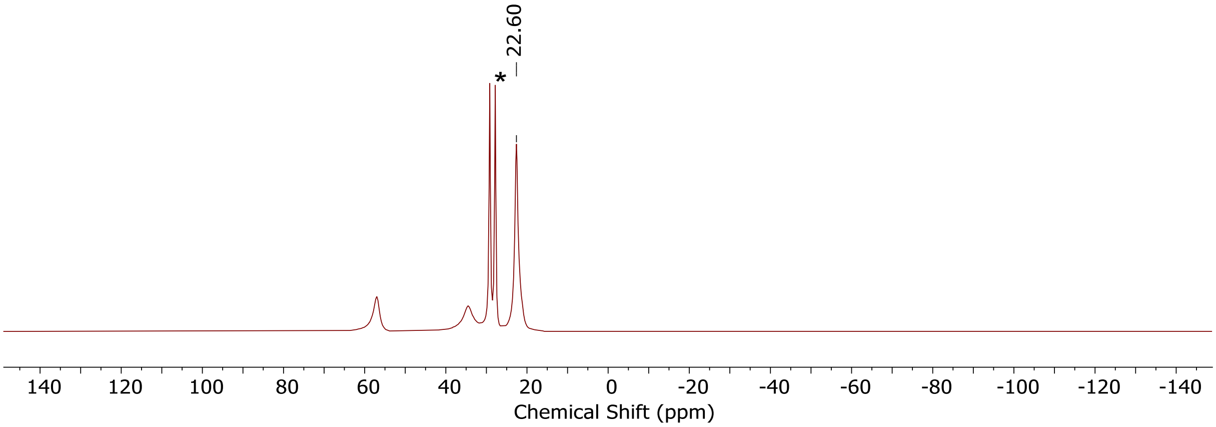

Figure S102. ^{11}B NMR spectrum (C_6D_6) of reaction mixture where **13a** is hydroborated with **7**. * denotes unreacted HB(pin).

5.4. Alkene – 14b – 4,4,5,5-tetramethyl-2-phenethyl-1,3,2-dioxaborolane

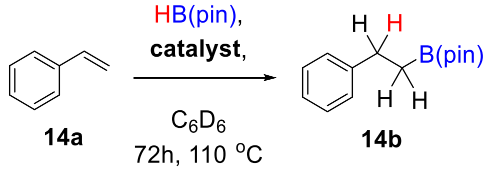

Table S8. Hydroboration of **14a** catalyzed by **1–5**, **7**, **8** in C_6D_6. Temperature = 110 °C. Time = 72 hours.

**Catalyst**	**1**[2]	**2**	**4**	**3**	**5**	**7**	**8**[2]
**Conversion/ %**	80	70	89	73	91	65	68

Annotated spectral data provided from the reaction mixture where ***14a*** *is hydroborated with* ***5****.*

^{1}H NMR (400 MHz, 298 K, C_6D_6): δ = 7.17–7.19 (m, 2H, *Ar*), 7.10–7.14 (m, 3H, *Ar*), 2.86 (t, $^3J_{HH}$ = 8.0 Hz, 2H(91%) observed integration 1.82, C*H*$_2$), 1.27 (t, $^3J_{HH}$ = 8.0 Hz, 2H(91%) observed integration 1.82, C*H*$_2$), 1.00 (s, 12H(91%) observed integration 10.92, B*pin*) ppm.

^{11}B NMR (128 MHz, 298 K, C_6D_6): δ = 34.08 (s) ppm.

Mass spectrometry (ESI): $C_{14}H_{21}BO_2$+H ([M+H]$^+$); Calcd. = 233.1713, Found = 233.1710.

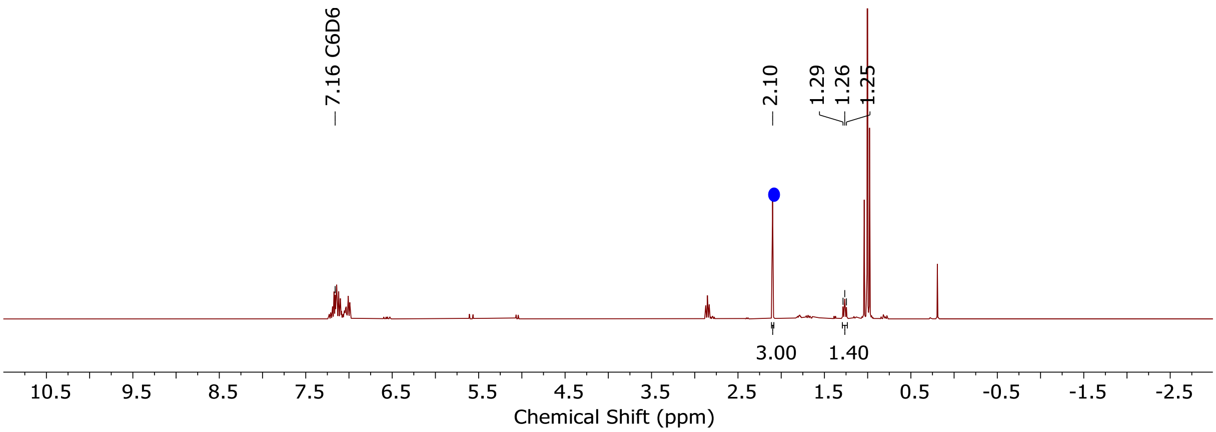

Figure S103. ^{1}H NMR spectrum (C_6D_6) of reaction mixture where **14a** is hydroborated with **2**. ● = toluene internal standard resonance.

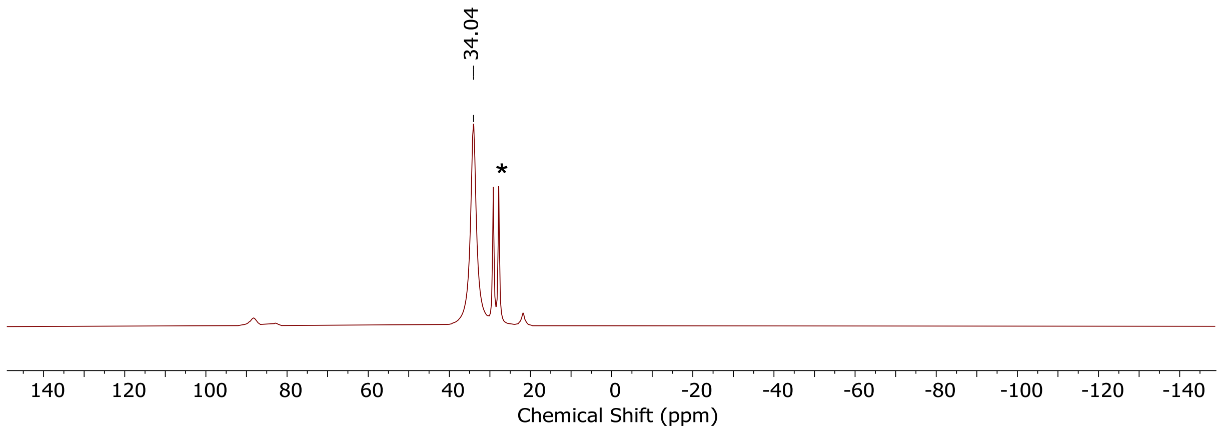

Figure S104. ^{11}B NMR spectrum (C_6D_6) of reaction mixture where **14a** is hydroborated with **2**. * denotes unreacted HB(pin).

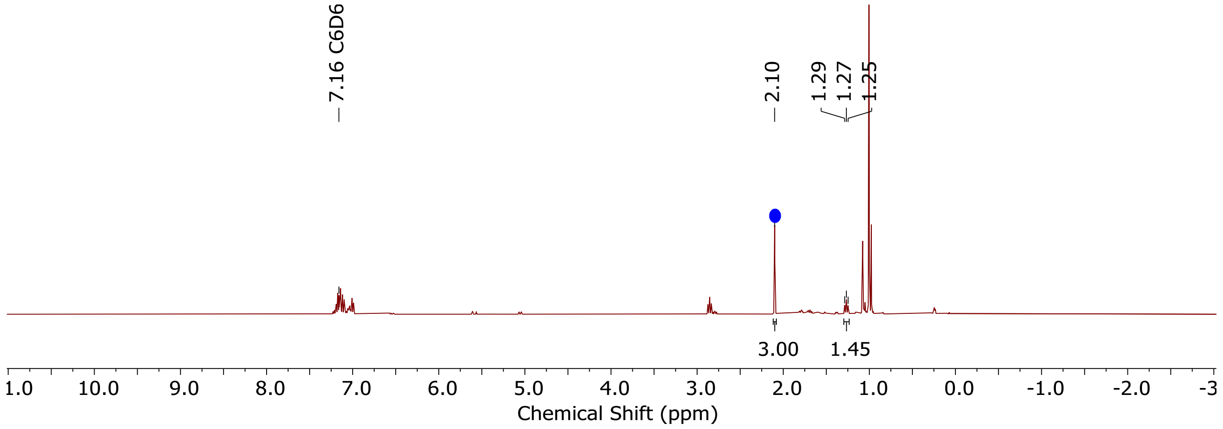

Figure S105. ^{1}H NMR spectrum (C_6D_6) of reaction mixture where **14a** is hydroborated with **3**. ● = toluene internal standard resonance.

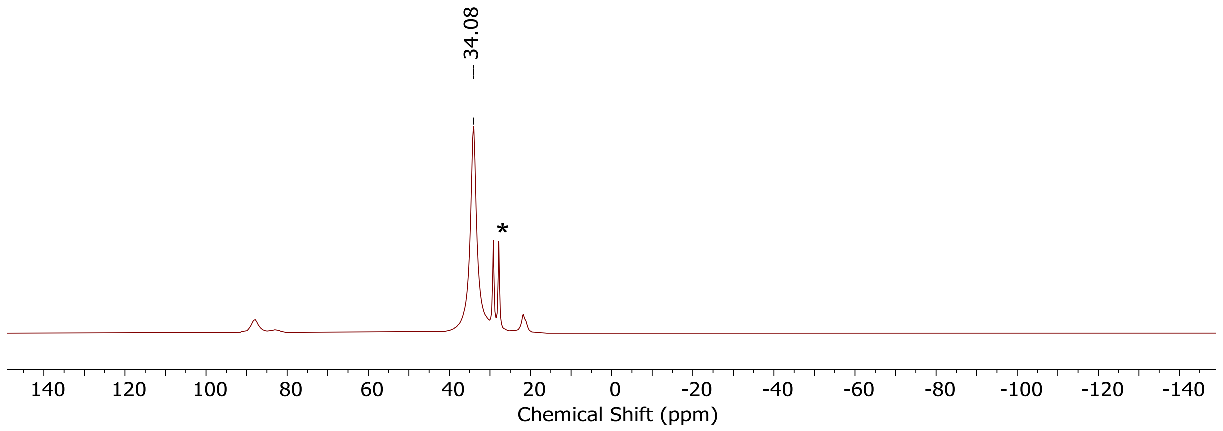

Figure S106. ^{11}B NMR spectrum (C_6D_6) of reaction mixture where **14a** is hydroborated with **3**. * denotes unreacted HB(pin).

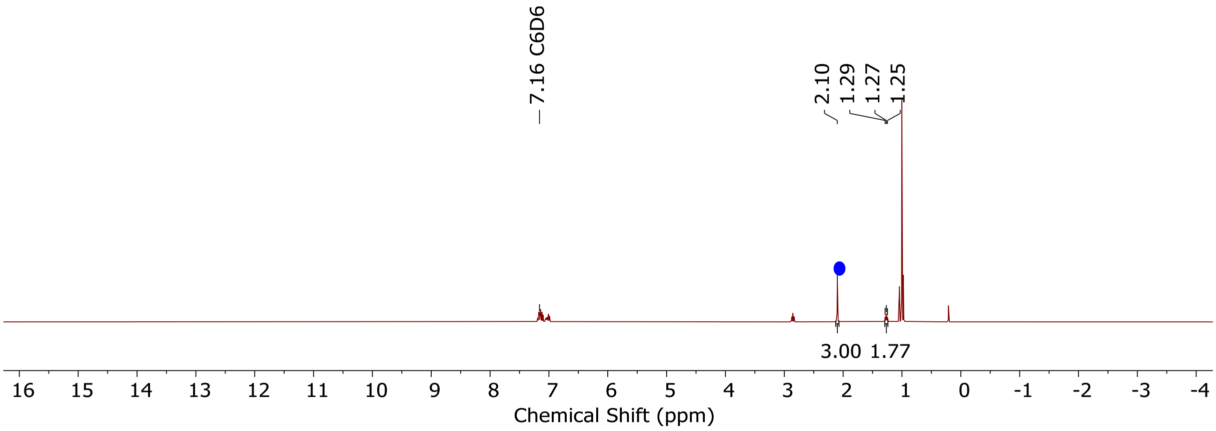

Figure S107. ^{1}H NMR spectrum (C_6D_6) of reaction mixture where **14a** is hydroborated with **4**. ● = toluene internal standard resonance.

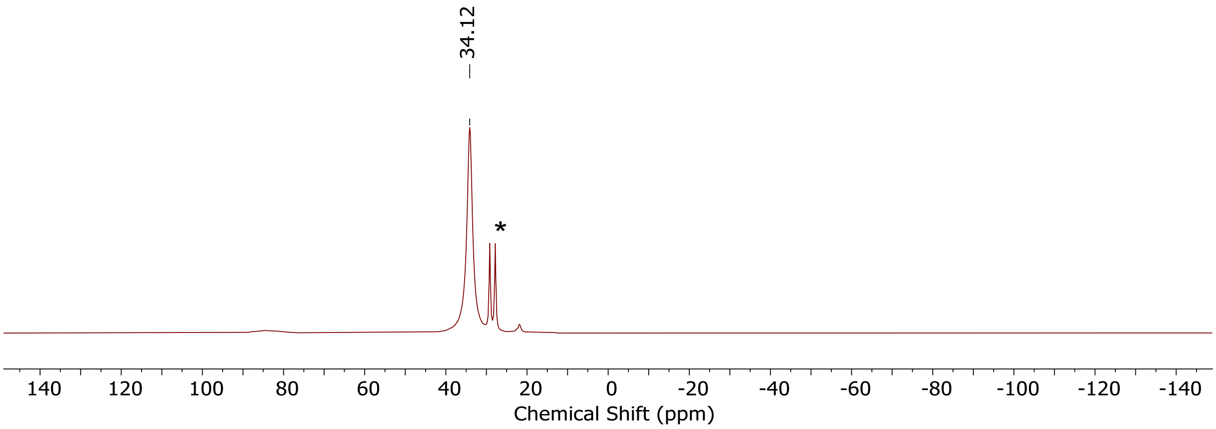

Figure S108. ^{11}B NMR spectrum (C_6D_6) of reaction mixture where **14a** is hydroborated with **4**. * denotes unreacted HB(pin).

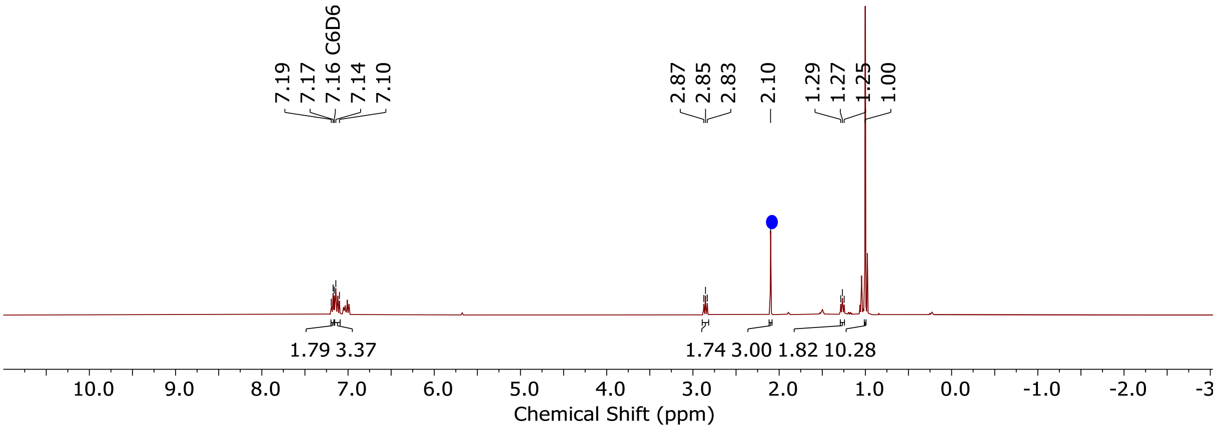

Figure S109. ^{1}H NMR spectrum (C_6D_6) of reaction mixture where **14a** is hydroborated with **5**. ● = toluene internal standard resonance.

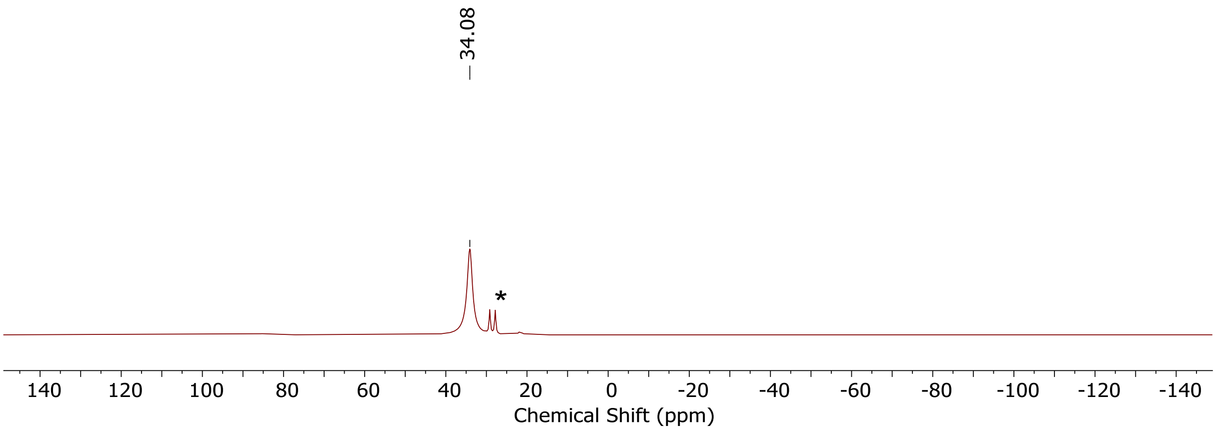

Figure S110. ^{11}B NMR spectrum (C_6D_6) of reaction mixture where **14a** is hydroborated with **5**. * denotes unreacted HB(pin).

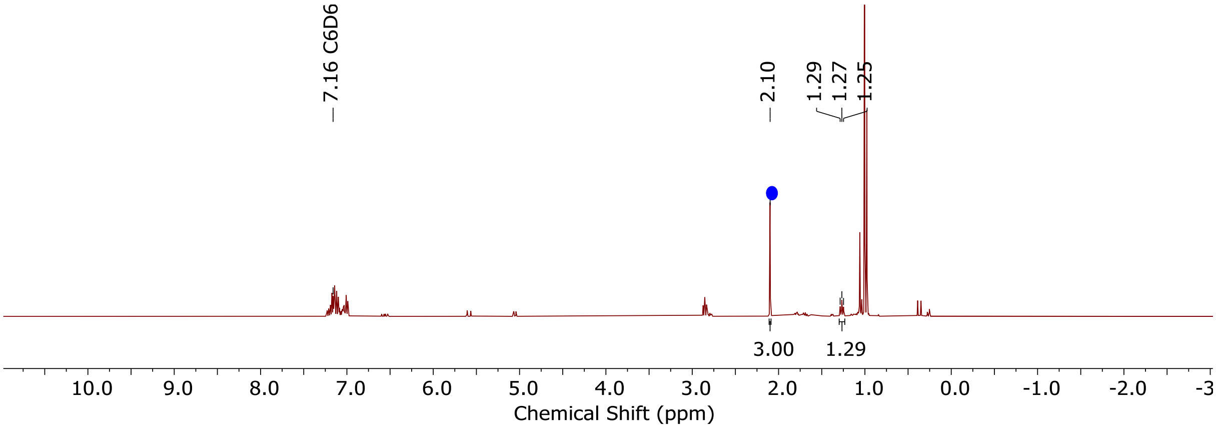

Figure S111. ^{1}H NMR spectrum (C_6D_6) of reaction mixture where **14a** is hydroborated with **7**. ● = toluene internal standard resonance.

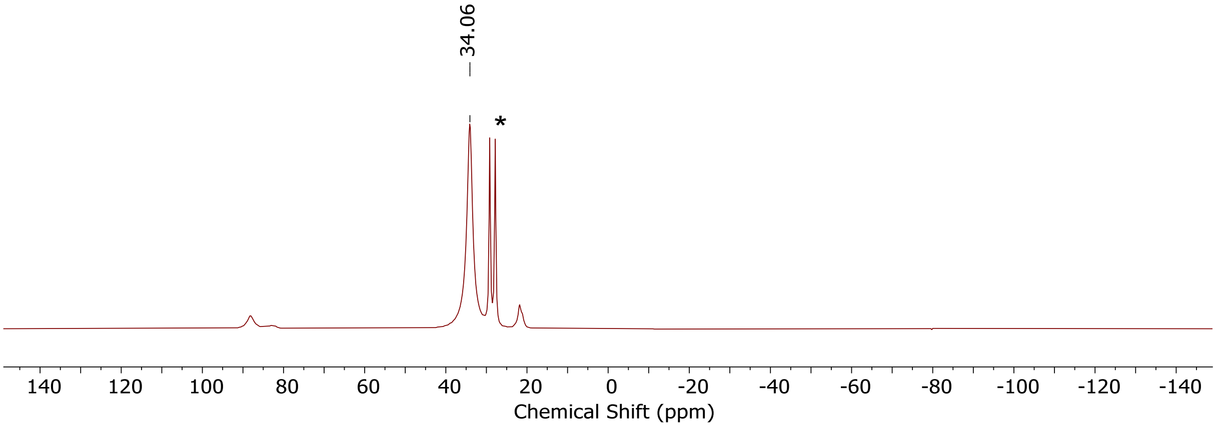

Figure S112. ^{11}B NMR spectrum (C_6D_6) of reaction mixture where **14a** is hydroborated with **7**. * denotes unreacted HB(pin).

5.5. Alkyne – 15b – (*E*)-4,4,5,5-tetramethyl-2-styryl-1,3,2-dioxaborolane

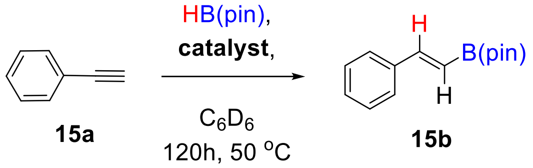

Table S9. Hydroboration of **15a** catalyzed by **1–5**, **7**, **8** in C_6D_6. Temperature = 50 °C. Time = 120 hours.

**Catalyst**	**1**[2]	**2**	**3**	**4**	**5**	**7**	**8**[2]
**Conversion/ %**	79	57	59	97	95	61	68

*Annotated spectral data provided from the reaction mixture where **15a** is hydroborated with **5**.*

^{1}H NMR (400 MHz, 298 K, C_6D_6): δ = 7.77 (d, $^3J_{HH}$ = 18.28 Hz, 1H(95%) observed integration 0.95, C*H*), 7.33 (d, $^3J_{HH}$ = 7.44 Hz, 2H, *Ar*), 7.00–7.03 (m, 3H, *Ar*), 6.47 (d, $^3J_{HH}$ = 18.28 Hz, 1H(95%) observed integration 0.95, C*H*), 1.13 (s, 12H, B*pin*) ppm.

^{11}B NMR (128 MHz, 298 K, C_6D_6): δ = 30.60 (s) ppm.

Mass spectrometry (APCI): $C_{14}H_{19}BO_2$+H ([M+H]$^+$); Calcd. = 231.1551, Found = 231.1551.

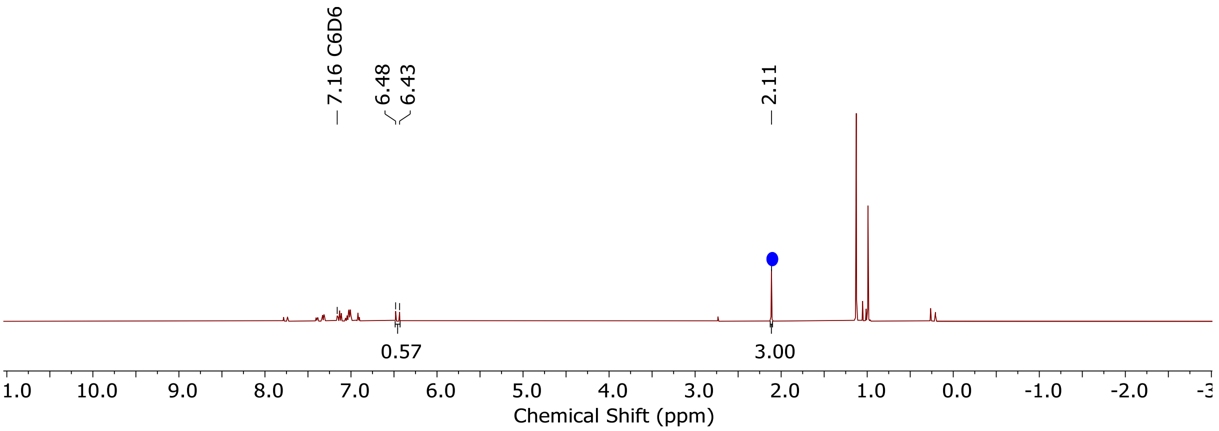

Figure S113. ^{1}H NMR spectrum (C_6D_6) of reaction mixture where **15a** is hydroborated with **2**. ● = toluene internal standard resonance.

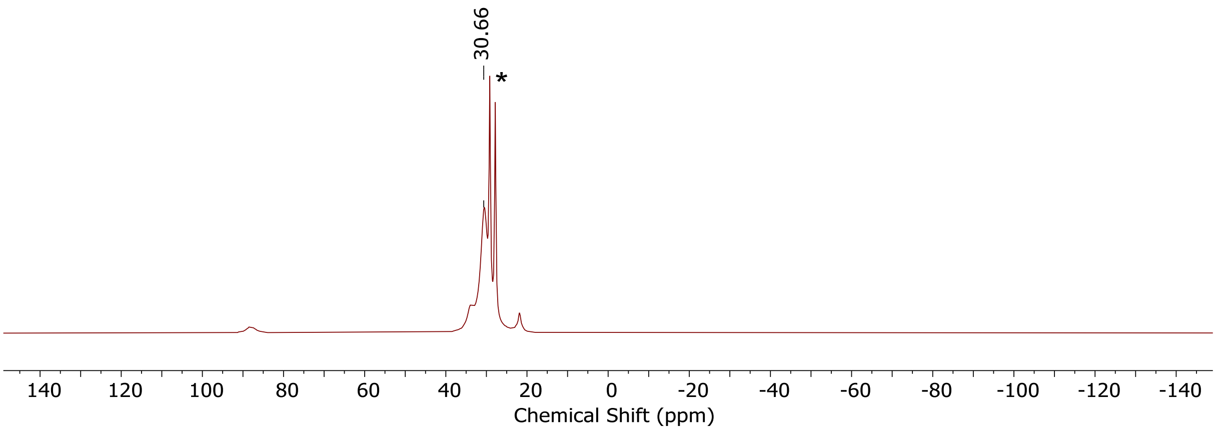

Figure S114. ^{11}B NMR spectrum (C_6D_6) of reaction mixture where **15a** is hydroborated with **2**. * denotes unreacted HB(pin).

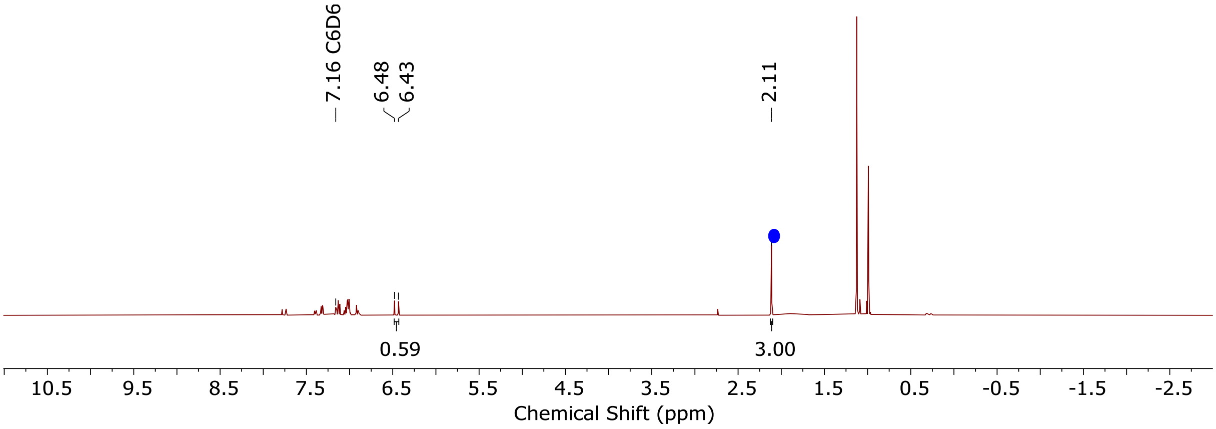

Figure S115. ^{1}H NMR spectrum (C_6D_6) of reaction mixture where **15a** is hydroborated with **3**. ● = toluene internal standard resonance.

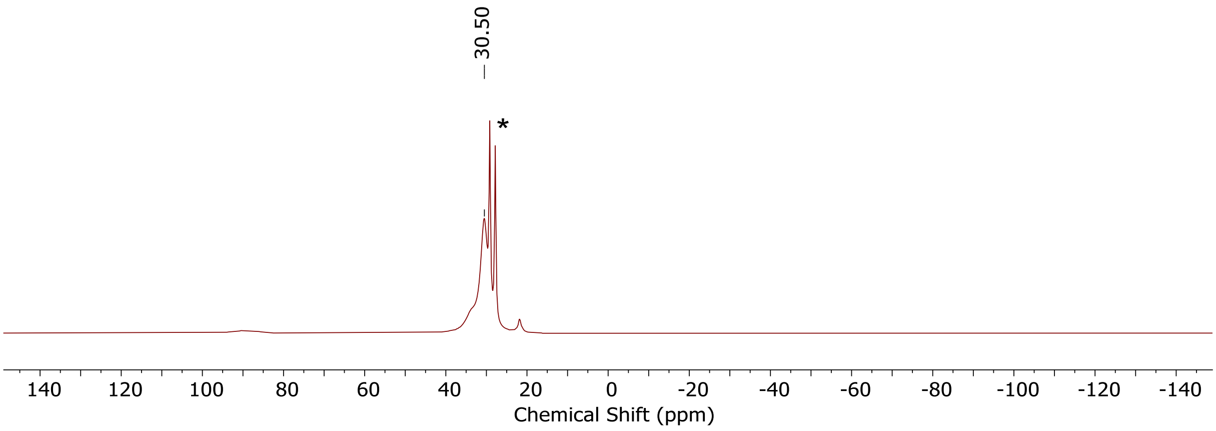

Figure S116. ^{11}B NMR spectrum (C_6D_6) of reaction mixture where **15a** is hydroborated with **3**. * denotes unreacted HB(pin).

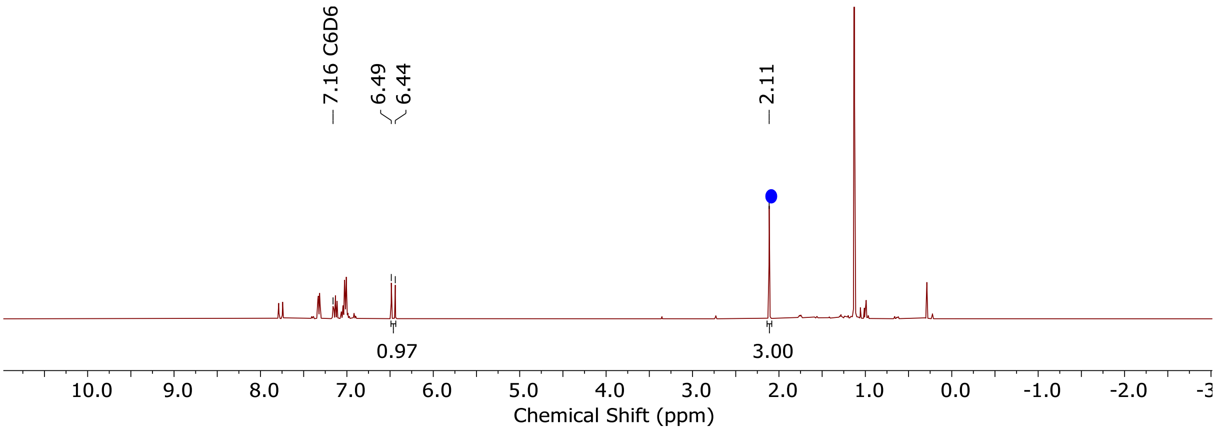

Figure S117. ^{1}H NMR spectrum (C_6D_6) of reaction mixture where **15a** is hydroborated with **4**. ● = toluene internal standard resonance.

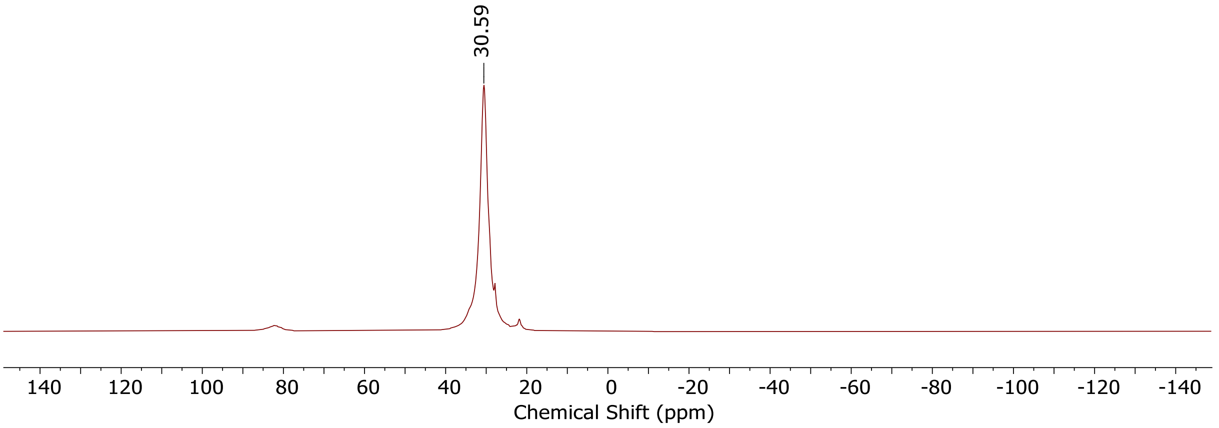

Figure S118. ^{11}B NMR spectrum (C_6D_6) of reaction mixture where **15a** is hydroborated with **4**.

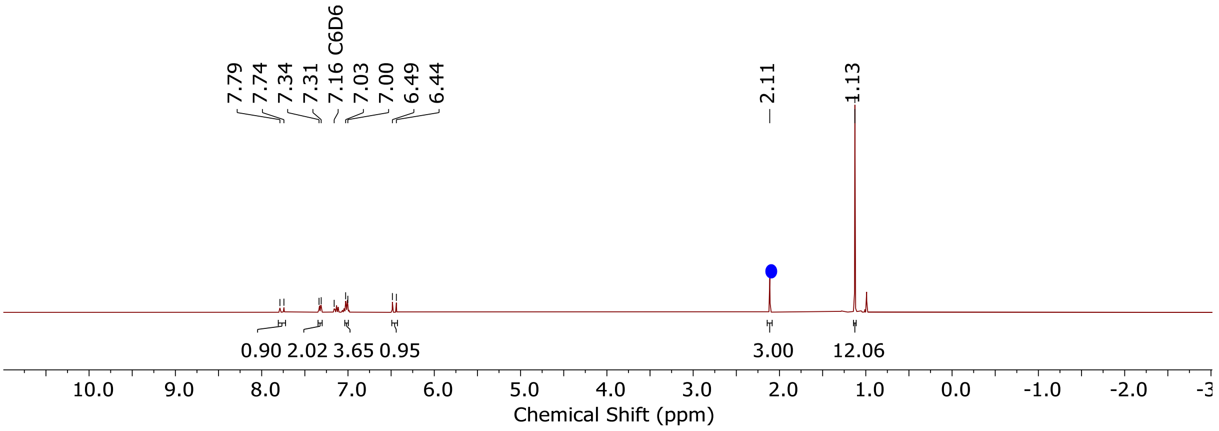

Figure S119. ^{1}H NMR spectrum (C_6D_6) of reaction mixture where **15a** is hydroborated with **5**. ● = toluene internal standard resonance.

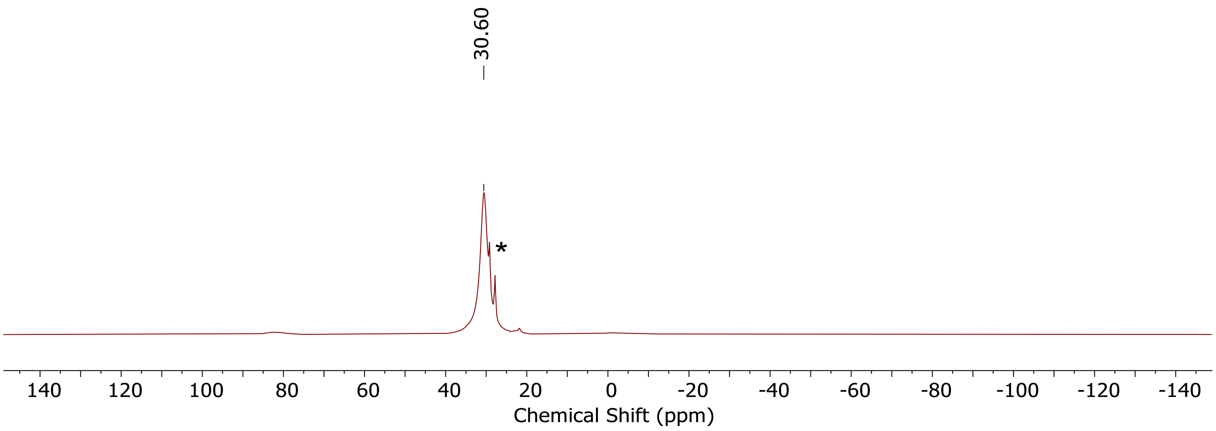

Figure S120. ^{11}B NMR spectrum (C_6D_6) of reaction mixture where **15a** is hydroborated with **5**. * denotes unreacted HB(pin).

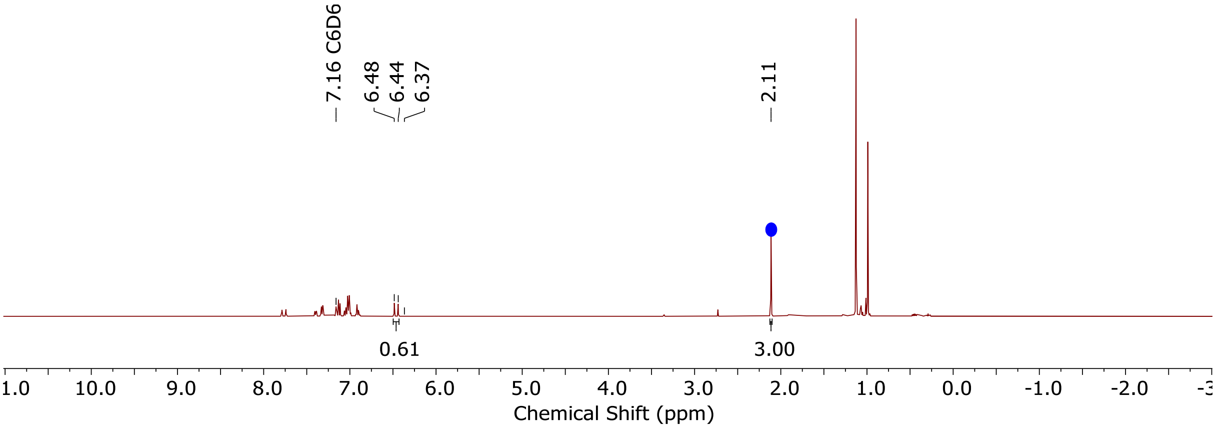

Figure S121. ^{1}H NMR spectrum (C_6D_6) of reaction mixture where **15a** is hydroborated with **7**. ● = toluene internal standard resonance.

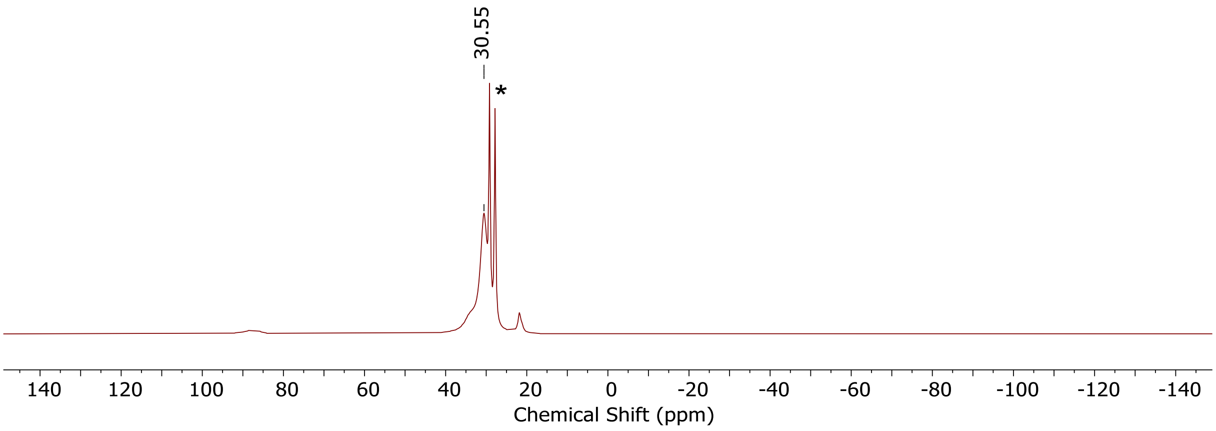

Figure S122. ^{11}B NMR spectrum (C_6D_6) of reaction mixture where **15a** is hydroborated with **7**. * denotes unreacted HB(pin).

5.6. Nitrile – 16b – N-benzyl-4,4,5,5-tetramethyl-N-(4,4,5,5-tetramethyl-1,3,2-dioxaborolan-2-yl)-1,3,2-dioxaborolan-2-amine

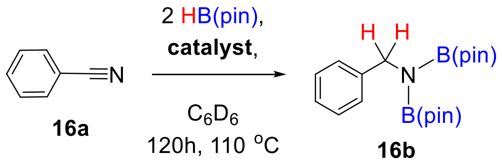

Table S10. Hydroboration of **16a** catalyzed by **1–5**, **7**, **8** in C_6D_6. Temperature = 110 °C. Time = 120 hours.

Catalyst	1[2]	2	3	4	5	7	8[2]
**Conversion/ %**	84	89	83	>99	>99	81	70

Annotated spectral data provided from the reaction mixture where ***16a*** *is hydroborated with* ***5****.*

^{1}H NMR (400 MHz, 298 K, C_6D_6): δ = 7.60 (d, $^3J_{HH}$ = 7.65 Hz, 2H, *Ar*), 7.26 (t, $^3J_{HH}$ = 7.65 Hz, 2H, *Ar*), 7.13–7.14 (m, 1H, *Ar*), 4.62 (s, 2H, CH_2), 1.03 (s, 24H, B*pin*) ppm.

^{11}B NMR (128 MHz, 298 K, C_6D_6): δ = 26.46 (s) ppm.

Mass spectrometry (APCI): $C_{19}H_{31}B_2NO_4$+H ([M+H]$^+$); Calcd. = 360.2512, Found = 360.2511.

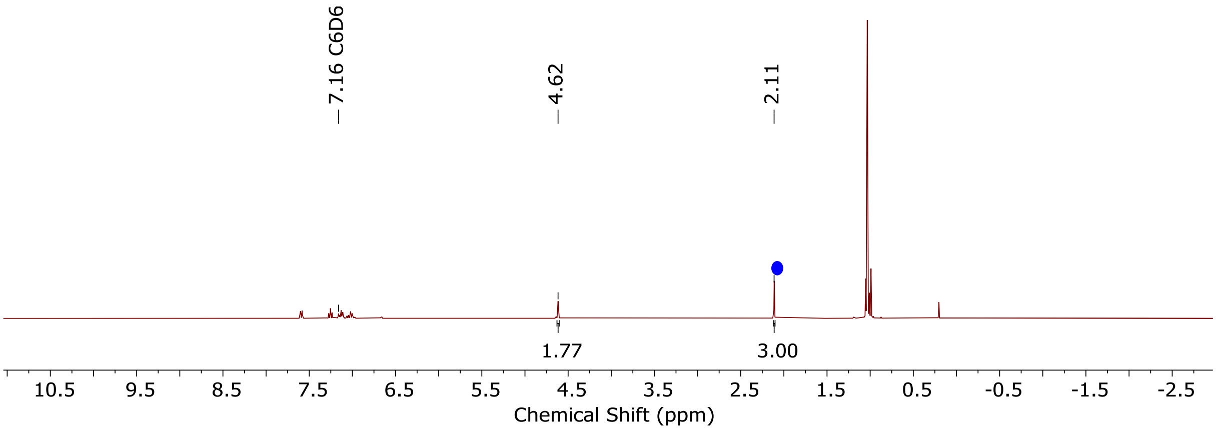

Figure S123. ^{1}H NMR spectrum (C_6D_6) of reaction mixture where **16a** is hydroborated with **2.** ● = toluene internal standard resonance.

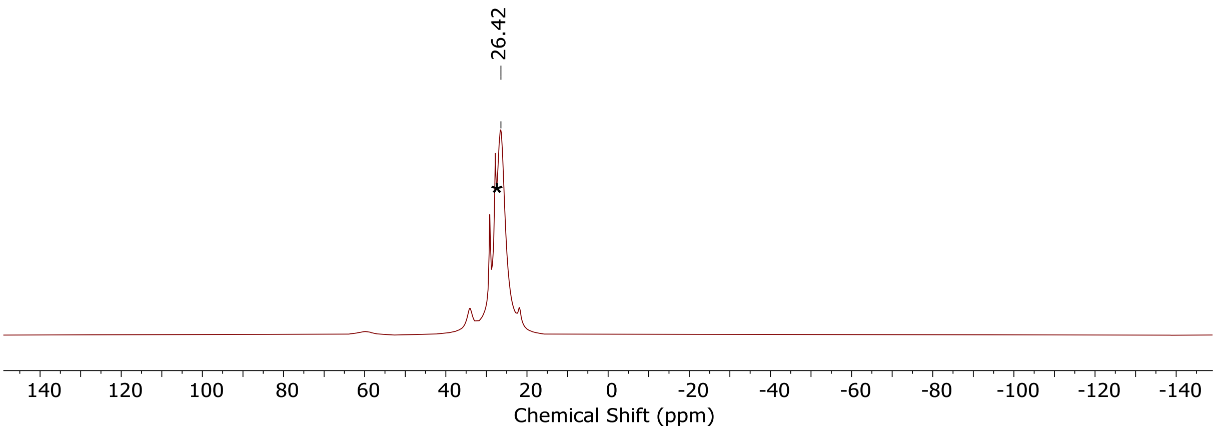

Figure S124. ^{11}B NMR spectrum (C_6D_6) of reaction mixture where **16a** is hydroborated with **2**. * denotes unreacted HB(pin).

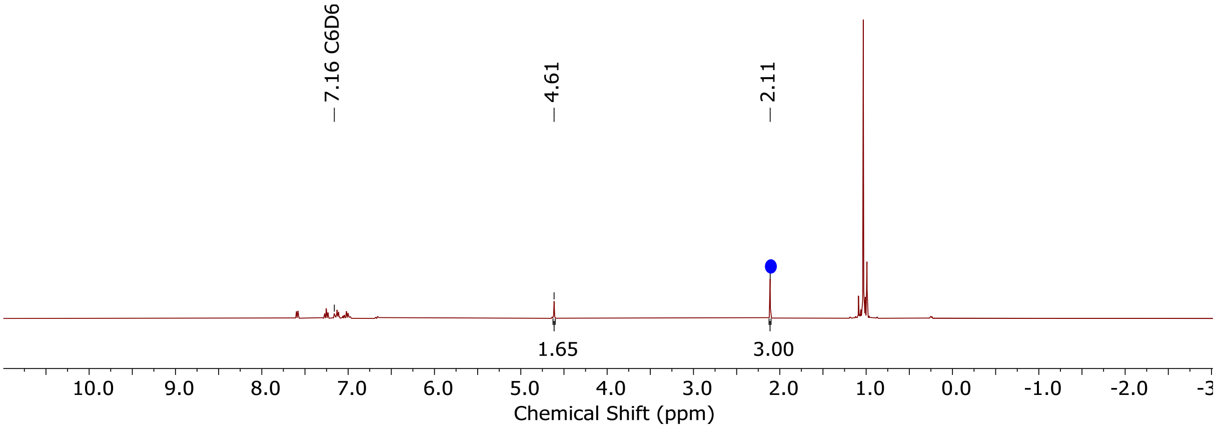

Figure S125. ^{1}H NMR spectrum (C_6D_6) of reaction mixture where **16a** is hydroborated with **3**. ● = toluene internal standard resonance.

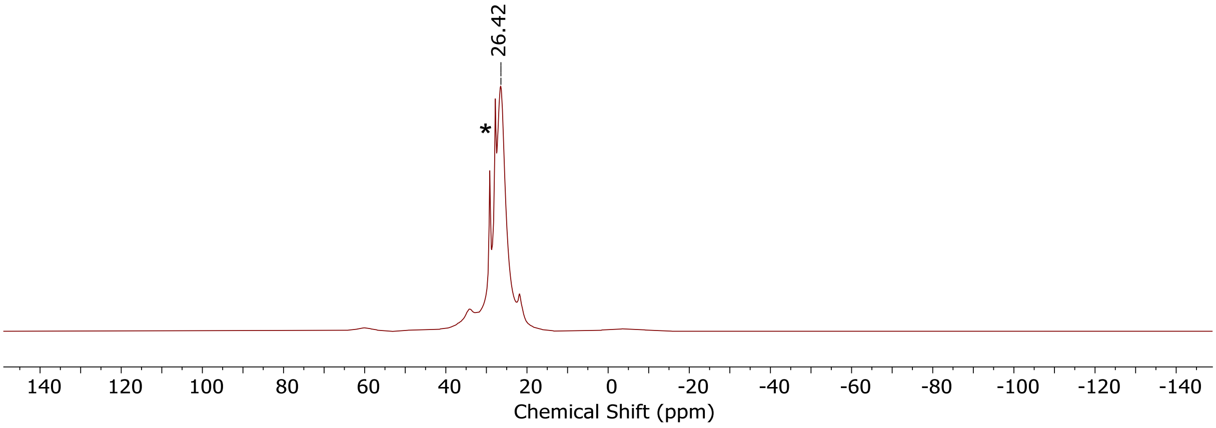

Figure S126. ^{11}B NMR spectrum (C_6D_6) of reaction mixture where **16a** is hydroborated with **3**. * denotes unreacted HB(pin).

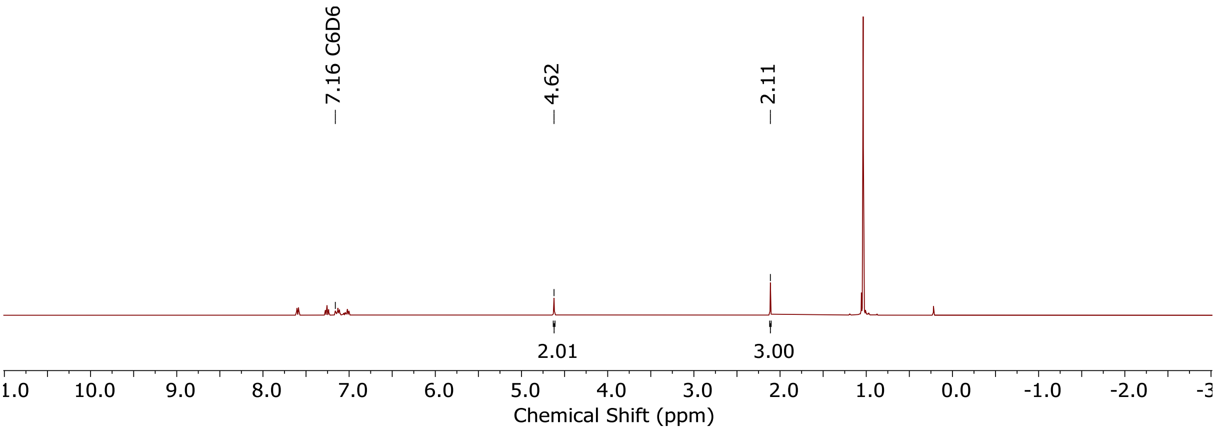

Figure S127. ^{1}H NMR spectrum (C_6D_6) of reaction mixture where **16a** is hydroborated with **4**. ● = toluene internal standard resonance.

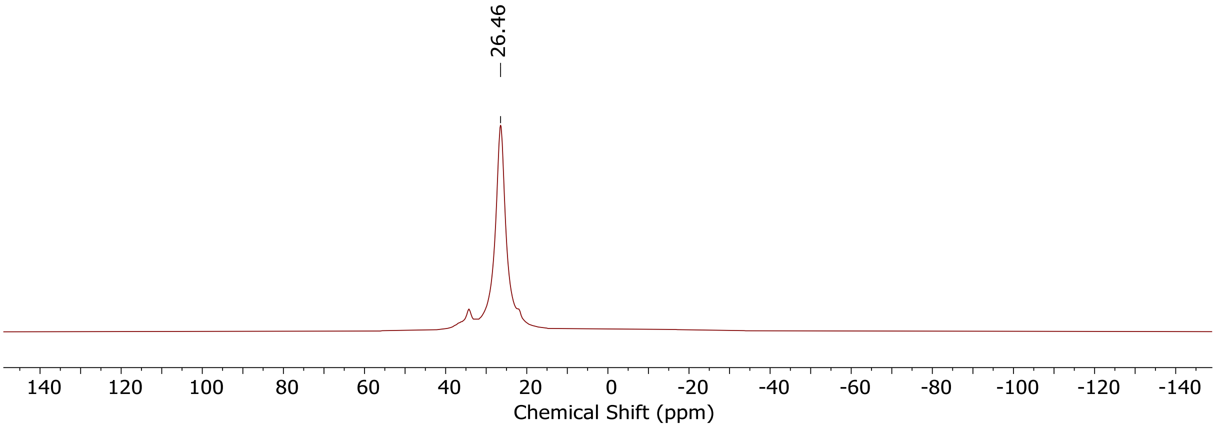

Figure S128. ^{11}B NMR spectrum (C_6D_6) of reaction mixture where **16a** is hydroborated with **4**.

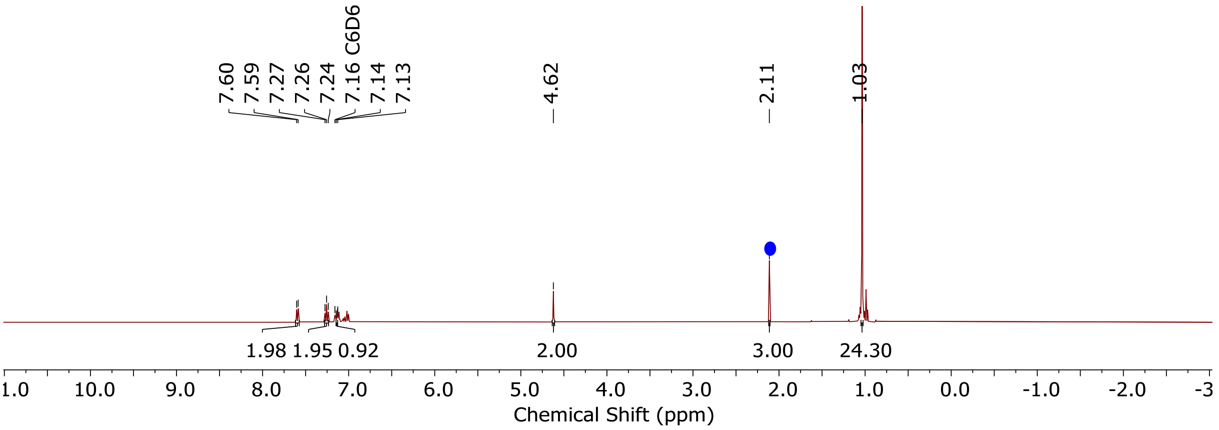

Figure S129. ^{1}H NMR spectrum (C_6D_6) of reaction mixture where **16a** is hydroborated with **5**. ● = toluene internal standard resonance.

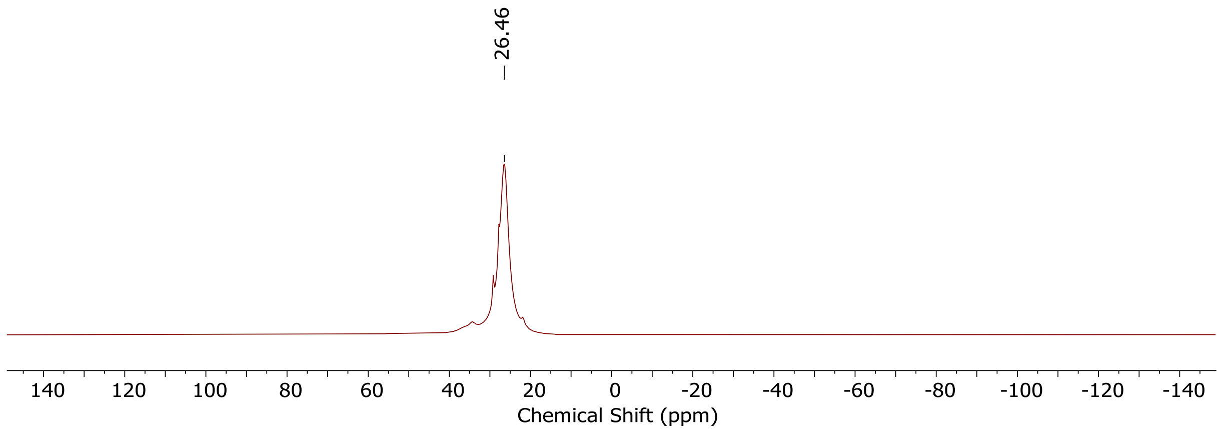

Figure S130. ^{11}B NMR spectrum (C_6D_6) of reaction mixture where **16a** is hydroborated with **5**.

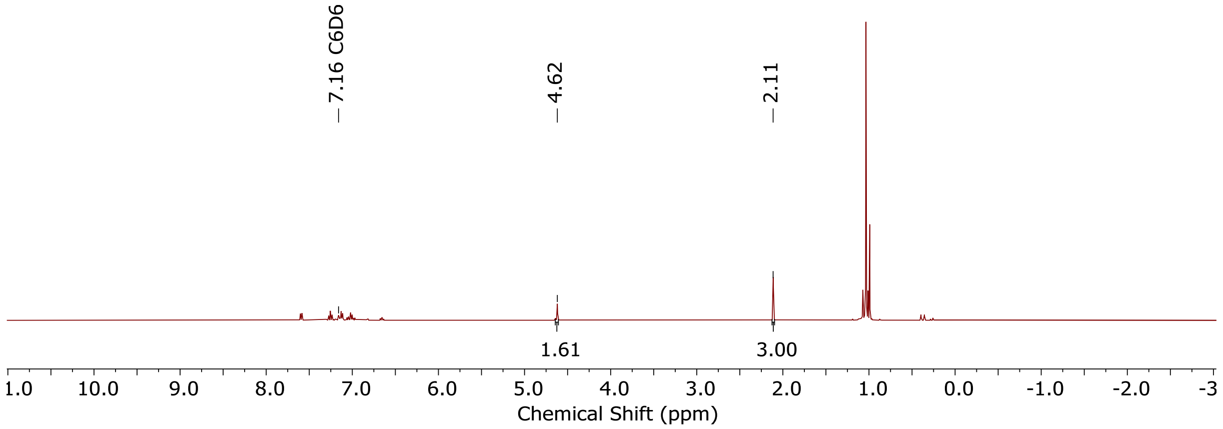

Figure S131. ^{1}H NMR spectrum (C_6D_6) of reaction mixture where **16a** is hydroborated with **7**. ● = toluene internal standard resonance.

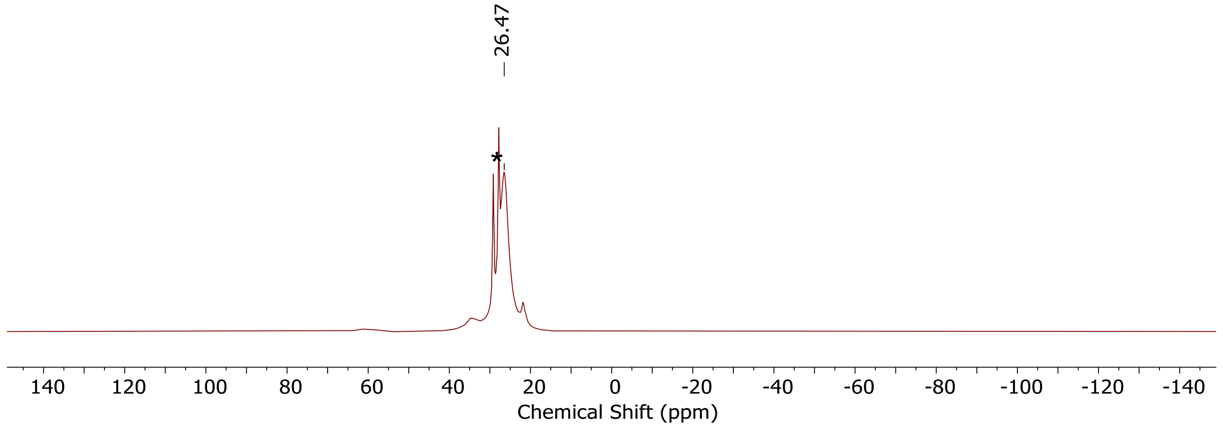

Figure S132. ^{11}B NMR spectrum (C_6D_6) of reaction mixture where **16a** is hydroborated with **7**. * denotes unreacted HB(pin).

6. Studies into Catalyst–Substrate Interactions

Substrate coordination to the Lewis acid site was probed by NMR spectroscopy. Catalyst **3**, **5**, and **7** (0.02 mmol) in C_6D_6 (0.5 mL) was reacted with three equivalents of substrate (0.06 mmol). The reaction was monitored by ^{1}H, ^{11}B, $^{29}Si\{^{1}H\}$, and ^{31}P NMR spectroscopy at room temperature.

However, while we previously observed substrate coordination of carbodiimide **11a** and nitrile **16a** to catalyst **1**,[2] we detected no coordination of substrates **11a–16a** or HB(pin) to catalysts **3**, **5**, and **7**.

7. Testing for Hidden Catalysis

It has previously been reported by Thomas and co-workers that BH_3 can play a 'hidden role' in hydroboration catalysis when HB(pin) is employed as the hydroborating agent.[13] Following from our previous assessment of 'hidden catalysis' when catalyst **1** is employed, we further assessed catalysts **3**, **5**, and **7**. Benzonitrile (**16a**, 0.1 mmol) and two equivalents of HB(pin) were reacted at 110 °C for 120 hours, catalyzed by 5 mol% **3**, **5**, and **7** to generate **16b** to give the conversions reported in section 6.6. The reaction mixture was investigated by NMR spectroscopy. Next, tetramethylethylenediamine (TMEDA, 0.2 mmol) was added to the reaction mixture and reinvestigated by NMR spectroscopy. Comparison of the ^{11}B NMR spectra to independently prepared ^{11}B NMR spectra of TMEDA●BH_3 confirmed that BH_3 or any other borohydride are not detected during the catalysis.

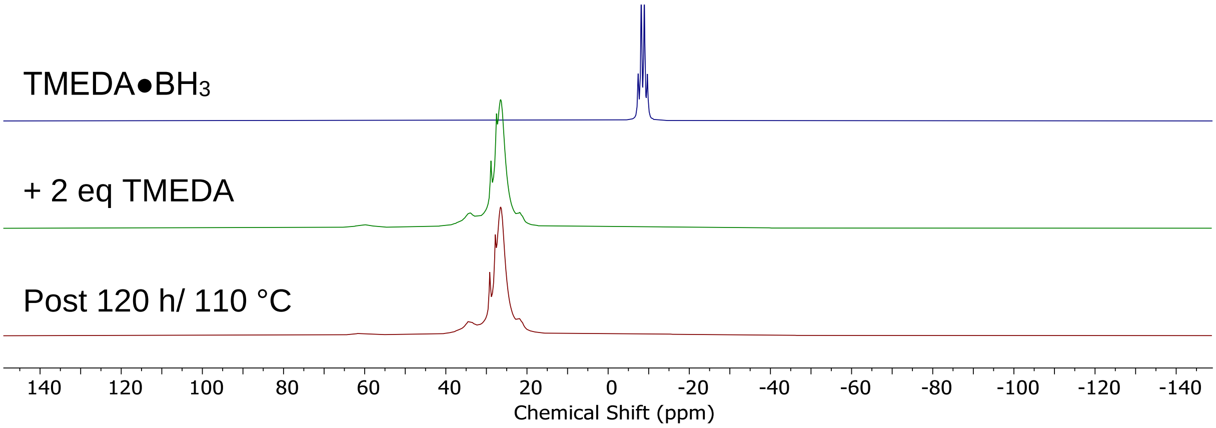

Figure S133. ^{11}B NMR spectra (C_6D_6) of **16a** catalyzed to **16b** by catalyst **3** (5 mol%, red); subsequent addition of TMEDA (green); independently prepared TMEDA●BH_3 (blue).

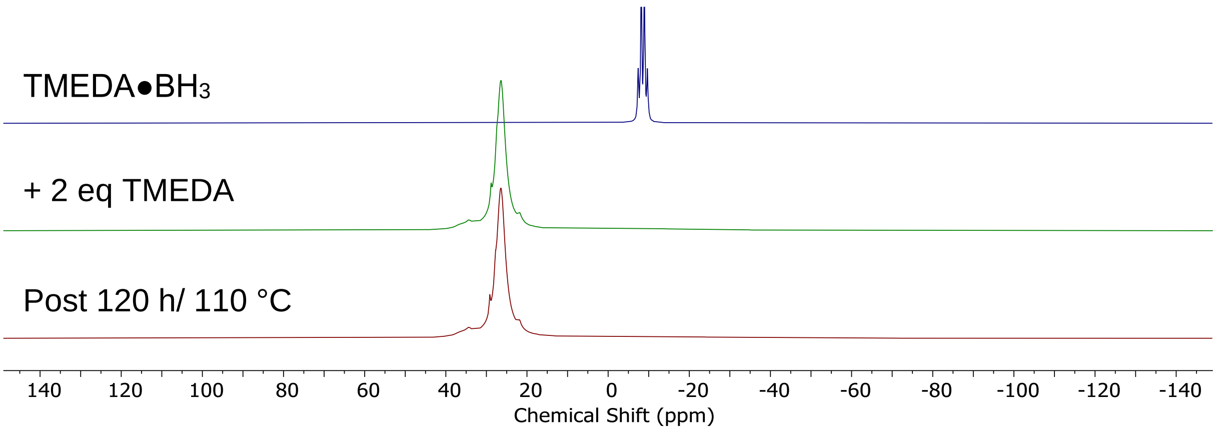

Figure S134. ^{11}B NMR spectra (C_6D_6) of **16a** catalyzed to **16b** by catalyst **5** (5 mol%, red); subsequent addition of TMEDA (green); independently prepared TMEDA●BH_3 (blue).

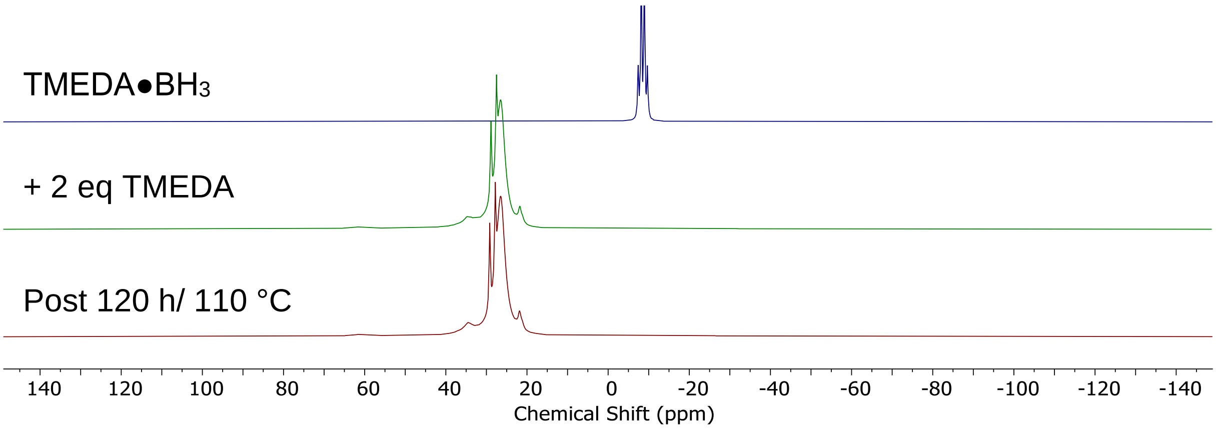

Figure S135. ^{11}B NMR spectra (C_6D_6) of **16a** catalyzed to **16b** by catalyst **7** (5 mol%, red); subsequent addition of TMEDA (green); independently prepared TMEDA●BH_3 (blue).

8. Crystallographic Data

Table S11. Crystallographic data for **3**, **9**, and **10**.

	({C_8H_{14}}$BCH_2CH_2CH_2SiMe_2$)$_3P_7$ (**3**)	($CH_2CHSiMe_2$)$_3As_7$ (**9**)	(Mes{(C_8H_{14})BO}CH)$_3P_7$ (**10**)
Formula	C39H78B3P7Si3	C12H27As7Si3	C58H88B3O4P7
Fw	880.50	780.04	1098.50
cryst size, mm	0.227 x 0.031 x 0.017	0.29 x 0.08 x 0.03	0.22 x 0.039 x 0.034
cryst syst	hexagonal	trigonal	monoclinic
space group	P63	R-3	P121/c1
collection temperature, K	99.99(10)	99.99(10)	100(2)
a, Å	20.7398(7)	11.0090(3)	19.5326(5)
b, Å	20.7398(7)	11.0090(3)	15.9350(2)
c, Å	6.8497(4)	35.757(2)	20.2064(5)
α, °	90	90	90
β, °	90	90	107.576(3)
γ, °	120	120	90
V, $Å^3$	2551.6(2)	3753.1(3)	5995.7(2)
Z	2	6	4
ρ_{calcd}, g cm^{-3}	1.146	2.071	1.217
μ, mm^{-1}	3.116	11.996	2.253
no. of reflections made	10469	12294	59217
no. of unique reflns, R_{int}	2742, 0.0884	1716, 0.0726	12265, 0.0877
no. of reflns with $F^2 > 2\sigma(F^2)$	2014	1315	8653
transmn coeff range	0.488-1.000	0.52016-1.00000	0.586-1.000
R, $R_w{}^a$ ($F^2 > 2\sigma(F^2)$)	0.0510, 0.1199	0.0765, 0.2039	0.0583, 0.1416
R, $R_w{}^a$ (all data)	0.0753, 0.1395	0.0899, 0.2126	0.0894, 0.1603
S^a	0.986	1.132	1.040
Parameters	233, 538	86, 59	670, 2
max., min. diff map, e $Å^{-3}$	0.390, -0.531	1.404, -1.720	1.437, -0.444
CCDC	2328547	2328549	2328550

[a] Conventional $R = \Sigma||F_o| - |F_c||/\Sigma|F_o|$; $R_w = [\Sigma w(F_o{}^2 - F_c{}^2)^2/\Sigma w(F_o{}^2)^2]^{1/2}$; $S = [\Sigma w(F_o{}^2 - F_c{}^2)^2/\text{no. data} - \text{no. params})]^{1/2}$ for all data.

9. Geometry Optimized Structures

9.1. CF_2O

Charge = 0 Multiplicity = 1

O -0.0466230000 -0.7269130000 0.7078620000

C -0.0198320000 0.4570640000 0.6604760000

F -1.0755860000 1.2641720000 0.6177690000

F 1.0710410000 1.2166780000 0.6398920000

9.2. $[CF_3O]^-$

Charge = –1 Multiplicity = 1

O -0.0024660000 -0.0727380000 0.7919310000

C -0.0077480000 -1.2197580000 1.2200720000

F -0.0372870000 -1.3904350000 2.6566080000

F 1.1166540000 -2.0616680000 0.8724600000

F -1.1111530000 -2.0694010000 0.8259280000

9.3. $[Me_3Si]^+$

Charge = +1 Multiplicity = 1

Si -0.0002000000 -0.0001130000 -0.0000860000

C -1.8306730000 -0.2180430000 -0.0001470000

C 1.1043370000 -1.4757570000 0.0000570000

C 0.7265010000 1.6938980000 0.0000840000

H 1.7763700000 -1.4347240000 -0.8884030000

H 0.5543200000 -2.4369350000 0.0008830000

H 1.7777070000 -1.4337300000 0.8874080000

H -2.1314140000 -0.8264600000 -0.8843510000

H -2.3870780000 0.7394000000 -0.0054770000

H -2.1314250000 -0.8157220000 0.8914840000

H 0.3534440000 2.2567190000 0.8870480000

H 0.3559000000 2.2553790000 -0.8888160000

H 1.8339950000 1.6970620000 0.0014590000

9.4. Me_3SiF

Charge = 0 Multiplicity = 1

F -2.4514360000 -0.5755960000 -1.3668210000

Si -3.2706670000 0.5824720000 -2.2238730000

C -2.7366820000 2.2546910000 -1.5424270000

C -5.1083400000 0.2732700000 -1.9531520000

C -2.7852850000 0.3766130000 -4.0314760000

H -5.7247850000 1.0162240000 -2.5030130000

H -5.3718330000 0.3455110000 -0.8770980000
H -5.4010510000 -0.7374200000 -2.3071960000
H -3.2481490000 3.0850840000 -2.0745550000
H -1.6423760000 2.4030790000 -1.6563780000
H -2.9807280000 2.3435440000 -0.4630480000
H -1.6909750000 0.5038270000 -4.1688000000
H -3.2961870000 1.1270910000 -4.6718380000
H -3.0595060000 -0.6313930000 -4.4073280000

9.5. Me_3SiH

Charge = 0 Multiplicity = 1
Si 4.0525710000 -0.9204570000 0.3574060000
C 4.1847450000 -1.4995980000 2.1604720000
C 5.3954250000 0.3656530000 -0.0264160000
C 4.1916850000 -2.3984650000 -0.8254150000
H 4.0814320000 -0.6470970000 2.8645360000
H 4.0925040000 -2.0769450000 -1.8836420000
H 6.4114740000 -0.0636060000 0.1062170000
H 2.7007650000 -0.2742770000 0.1601330000
H 5.1729510000 -2.9077000000 -0.7165120000
H 3.3972710000 -3.1483440000 -0.6259510000
H 3.3897840000 -2.2354850000 2.4046640000
H 5.1656490000 -1.9832810000 2.3558240000
H 5.3102770000 1.2472950000 0.6433060000
H 5.3154660000 0.7293060000 -1.0726210000

9.6. $[H]^-$

Charge = -1 Multiplicity = 1
H 5.2740000000 0.7150000000 -1.0680000000

9.7. SbF_5

Charge = 0 Multiplicity = 1
F -1.9354430000 0.7880830000 0.0118850000
F 0.1865520000 1.0762600000 -1.6358360000
F -1.0232240000 -1.7224270000 -0.3860040000
F 0.2213570000 0.5881340000 1.6245680000
F 1.5235260000 -0.8318660000 -0.2673920000
Sb -0.2057670000 -0.0211940000 -0.1292210000

9.8. $[SbF_6]^-$

Charge = –1 Multiplicity = 1

F 0.6934610000 1.8491390000 0.2844740000

F 0.2883260000 -0.4182980000 1.7840280000

F -1.8501630000 0.8765200000 0.6436940000

F -0.6454980000 0.6963010000 -1.8202990000

F 1.4930270000 -0.5985220000 -0.6799630000

F -1.0505750000 -1.5711420000 -0.3207950000

Sb -0.1785690000 0.1390010000 -0.0181390000

9.9. $[HSbF_5]^-$

Charge = -1 Multiplicity = 1

F -1.9858220000 0.8997830000 0.3130360000

F 0.3346960000 1.6056330000 -1.0594700000

F -1.0310680000 -1.7040220000 0.5537970000

F 0.4494240000 0.4496570000 1.4417800000

F 1.3118730000 -0.9606060000 -0.7175670000

Sb -0.3612480000 -0.0442780000 -0.2546640000

H -1.0848550000 -0.4851660000 -1.7689130000

9.10. $B(C_6F_5)_3$

Charge = 0 Multiplicity = 1

B -0.1527200000 0.4557970000 -0.1605200000

C -0.5860590000 1.3581700000 -1.3766080000

C -0.1083040000 -1.1107320000 -0.3176840000

C 0.2355380000 1.1209860000 1.2133700000

C -0.0238350000 -3.9535890000 -0.6022600000

C 0.3991710000 -3.1282370000 -1.6617220000

C 0.3396250000 -1.7339100000 -1.5065250000

C -0.5130710000 -1.9823330000 0.7209530000

C -0.4887670000 -3.3807870000 0.5971040000

F 0.0160700000 -5.2789060000 -0.7349120000

F 0.8493990000 -3.6771200000 -2.7944170000

F 0.7672890000 -0.9904480000 -2.5413400000

F -0.9782470000 -1.4844150000 1.8795460000

F -0.8990550000 -4.1686170000 1.5960760000

C 0.9379580000 2.3272340000 3.7079460000

C 1.6577230000 1.2114750000 3.2392850000

C 1.2910990000 0.6273090000 2.0161560000

C -0.4498370000 2.2498640000 1.7217490000

C -0.1271350000 2.8499490000 2.9495890000
F 1.2651870000 2.8894560000 4.8708630000
F 2.6748620000 0.7255680000 3.9577470000
F 2.0184480000 -0.4257380000 1.6056550000
F -1.4829100000 2.7773710000 1.0427140000
F -0.8136720000 3.9032240000 3.4036760000
C -1.3737740000 2.9974720000 -3.5815760000
C -2.0361030000 1.7786450000 -3.3397880000
C -1.6274720000 0.9825950000 -2.2575660000
C 0.0416280000 2.5951620000 -1.6551120000
C -0.3235410000 3.4096670000 -2.7390890000
F -1.7413530000 3.7617730000 -4.6093870000
F -3.0391770000 1.3964250000 -4.1365090000
F -2.3001260000 -0.1641980000 -2.0603520000
F 1.0588850000 3.0253600000 -0.8892410000
F 0.3092550000 4.5625400000 -2.9788190000

9.11. $[FB(C_6F_5)_3]^-$

Charge = –1 Multiplicity = 1

B -1.0312930000 0.2232650000 0.0282600000
C -0.9890050000 1.2531340000 -1.2823180000
C -0.3707440000 -1.2773630000 -0.2797500000
C -0.2759520000 1.0317420000 1.2858070000
C 0.5070170000 -3.9914620000 -0.6547500000
C 0.4773630000 -3.0981990000 -1.7352940000
C 0.0361790000 -1.7742170000 -1.5287690000
C -0.3270790000 -2.2166340000 0.7693280000
C 0.1011300000 -3.5465250000 0.6148250000
F 0.9186910000 -5.2624450000 -0.8310130000
F 0.8572570000 -3.5187830000 -2.9597680000
F 0.0221920000 -0.9984240000 -2.6379190000
F -0.6982780000 -1.8584350000 2.0180080000
F 0.1283960000 -4.3989990000 1.6599830000
C 0.8329230000 2.5318650000 3.4745310000
C 1.5555730000 1.4758250000 2.8979060000
C 0.9954700000 0.7624780000 1.8202290000
C -0.9522780000 2.1116220000 1.8861300000
C -0.4358920000 2.8532150000 2.9663140000
F 1.3509140000 3.2296770000 4.5048280000
F 2.7781100000 1.1653470000 3.3773000000

F 1.7704130000 -0.2248360000 1.3162040000
F -2.1605890000 2.5039580000 1.4302660000
F -1.1318830000 3.8755200000 3.5062330000
C -0.8508750000 3.1531150000 -3.4367820000
C -2.0824890000 2.6064350000 -3.0465420000
C -2.1310160000 1.6724020000 -1.9899130000
C 0.2195170000 1.8229440000 -1.7215220000
C 0.3193290000 2.7532990000 -2.7702760000
F -0.7891280000 4.0449120000 -4.4458020000
F -3.2081690000 2.9781200000 -3.6917710000
F -3.3572600000 1.1913170000 -1.7056160000
F 1.3867140000 1.4601540000 -1.1373620000
F 1.5113150000 3.2615440000 -3.1462110000
F -2.3785710000 -0.0295660000 0.4192040000

9.12. $[HB(C_6F_5)_3]^-$

Charge = -1 Multiplicity = 1

B -0.8351440000 0.4097510000 0.1454970000
C -0.9128380000 1.2985030000 -1.2384890000
C -0.3821490000 -1.1441140000 -0.1514660000
C 0.0158010000 1.1509140000 1.3427450000
C 0.2564940000 -3.8744060000 -0.6694120000
C 0.8865760000 -2.8594650000 -1.3782350000
C 0.5605020000 -1.5319060000 -1.1030320000
C -0.9783850000 -2.2019970000 0.5364870000
C -0.6826410000 -3.5452400000 0.2992220000
F 0.5559290000 -5.1569520000 -0.9138370000
F 1.8029000000 -3.1718720000 -2.3057280000
F 1.2329190000 -0.6071590000 -1.8082340000
F -1.8902590000 -1.9656980000 1.4924740000
F -1.2907490000 -4.5217170000 0.9890550000
C 1.3043530000 2.5140540000 3.5251700000
C 1.8979470000 1.3863420000 2.9412050000
C 1.2528190000 0.7411730000 1.8689720000
C -0.5339020000 2.2895500000 1.9631380000
C 0.0725980000 2.9714370000 3.0319030000
F 1.9071620000 3.1500350000 4.5487290000
F 3.0841410000 0.9431720000 3.4049690000
F 1.9098250000 -0.3225140000 1.3530720000
F -1.7163800000 2.7871310000 1.5380550000

F -0.5071170000 4.0553660000 3.5873090000
C -1.2174130000 2.8299100000 -3.6512060000
C -2.1860690000 1.8830380000 -3.2867370000
C -2.0130780000 1.1448070000 -2.1025630000
C 0.0324660000 2.2508250000 -1.6554170000
C -0.0982080000 3.0182200000 -2.8273750000
F -1.3602880000 3.5495260000 -4.7811440000
F -3.2647020000 1.6958530000 -4.0746250000
F -2.9830680000 0.2495170000 -1.8173070000
F 1.1505860000 2.4744200000 -0.9301120000
F 0.8408660000 3.9206490000 -3.1769340000
H -1.9904910000 0.3508480000 0.5658520000

9.13. ($\{C_8H_{14}\}BCH_2CH_2SiMe_2)_3P_7$ (**1**)
Charge = 0 Multiplicity = 1
P -0.6739050000 0.7135590000 0.5579860000
P -1.2933050000 -1.0028710000 -0.7200560000
P -0.3013540000 2.1986540000 -1.0606950000
P 1.4369510000 0.0951840000 0.9047810000
P 1.1122900000 1.1036760000 -2.4121640000
P 2.1197740000 -0.4892570000 -1.1485310000
P 0.2543570000 -0.9951630000 -2.3385080000
Si -3.2252910000 -0.3225750000 -1.8219680000
Si 1.0572970000 3.8463500000 -0.1309610000
Si 1.3425460000 -1.9430580000 2.0310830000
C -0.0793020000 -1.8128280000 3.2812880000
C 3.0034050000 -2.0147930000 2.9513840000
C 1.1586390000 -3.4546620000 0.8887680000
C 0.4286990000 4.1624320000 1.6299640000
C 0.7354860000 5.3650920000 -1.2247130000
C 2.9167420000 3.4185870000 -0.1451410000
C -4.2850820000 0.5660710000 -0.5088940000
C -4.0319550000 -1.9575730000 -2.3578810000
C -2.9362710000 0.7658100000 -3.3451620000
H -4.3171470000 -0.0770770000 0.3991270000
C -5.7190310000 0.9331640000 -0.9556160000
H -3.7318830000 1.4805200000 -0.1972370000
H -2.4309420000 1.7132260000 -3.0676930000
H -3.9085840000 1.0092570000 -3.8267260000
H -2.3066810000 0.2449250000 -4.0961300000

H -4.2311030000 -2.6171190000 -1.4879030000
H -3.3785080000 -2.5124770000 -3.0632740000
H -4.9966250000 -1.7649450000 -2.8752460000
H 1.2946920000 6.2459940000 -0.8426180000
H -0.3434410000 5.6239860000 -1.2394020000
H 1.0570750000 5.1855570000 -2.2720310000
H 3.1871700000 3.1193160000 -1.1833860000
H 3.0525980000 2.5074920000 0.4798480000
C 3.8373910000 4.5855820000 0.3466300000
H 0.9602200000 5.0315840000 2.0738770000
H 0.6019830000 3.2812420000 2.2811310000
H -0.6576810000 4.3882670000 1.6355460000
H 3.0652900000 -2.9329730000 3.5746560000
H 3.1272900000 -1.1388130000 3.6211220000
H 3.8584400000 -2.0257580000 2.2433490000
H -0.0747510000 -2.6951840000 3.9572210000
H -1.0631050000 -1.7740360000 2.7702870000
H 0.0198320000 -0.9037790000 3.9098690000
H 0.2274070000 -3.3180920000 0.2960360000
C 1.1433450000 -4.8231100000 1.6095370000
H 1.9873420000 -3.4159500000 0.1461700000
H -6.2904180000 0.0211030000 -1.2620490000
H -5.7056570000 1.5331620000 -1.8999610000
H 3.5513690000 4.8806950000 1.3802960000
H 3.7046140000 5.4703420000 -0.3143170000
H 0.3316390000 -4.8709370000 2.3750980000
H 2.0689420000 -4.9615800000 2.2276870000
B 1.0737620000 -6.1430180000 0.7381240000
C 0.8067370000 -7.5698660000 1.3796640000
H 0.7404110000 -7.5343410000 2.4907980000
C -0.5943150000 -8.0141450000 0.8475170000
H -0.8341590000 -9.0354790000 1.2244670000
H -1.3603400000 -7.3440970000 1.3029570000
C -0.7557580000 -7.9762350000 -0.6889050000
H -1.8345430000 -8.0747440000 -0.9427190000
H -0.2720640000 -8.8673880000 -1.1379250000
C -0.1991460000 -6.6931920000 -1.3464050000
H -0.1724260000 -6.8205000000 -2.4534290000
H -0.9148460000 -5.8586870000 -1.1629980000
C 1.2079580000 -6.2368510000 -0.8363060000

H 1.4241400000 -5.2564110000 -1.3135680000
C 2.3749240000 -7.2060810000 -1.1918310000
H 2.4289130000 -7.3466940000 -2.2962930000
H 3.3288810000 -6.7016310000 -0.9127800000
C 2.3241480000 -8.5857470000 -0.4993350000
H 3.3074360000 -9.0910770000 -0.6249490000
H 1.5977840000 -9.2406330000 -1.0225740000
C 1.9789290000 -8.5242510000 1.0051690000
H 2.8819420000 -8.1846970000 1.5638620000
H 1.7697230000 -9.5528770000 1.3808020000
B -6.6603680000 1.7164020000 0.0473210000
C -6.2944850000 2.1921960000 1.5129560000
H -5.2808830000 1.8678210000 1.8350820000
C -6.2866230000 3.7551640000 1.4714180000
H -6.0783730000 4.1564150000 2.4903510000
H -5.4205230000 4.0747060000 0.8465060000
C -7.5692690000 4.4078390000 0.9095190000
H -7.3681970000 5.4823080000 0.7023760000
H -8.3602740000 4.4088970000 1.6867050000
C -8.1026400000 3.7386660000 -0.3772220000
H -9.1217940000 4.1279610000 -0.6061480000
H -7.4657340000 4.0577430000 -1.2348590000
C -8.1244530000 2.1764860000 -0.3560350000
H -8.4149890000 1.8365440000 -1.3758930000
C -9.1347870000 1.5532750000 0.6539870000
H -10.1655600000 1.9171910000 0.4354270000
H -9.1646360000 0.4545150000 0.4677950000
C -8.8091030000 1.7925060000 2.1450370000
H -9.4373090000 1.1163300000 2.7662810000
H -9.1183200000 2.8171980000 2.4355370000
C -7.3237550000 1.5734480000 2.5073530000
H -7.1277470000 0.4773580000 2.5578270000
H -7.1318420000 1.9546140000 3.5371860000
B 5.2863560000 3.9663390000 0.2589380000
C 6.0329520000 3.2593260000 1.4640810000
H 5.3877600000 3.1454710000 2.3638470000
C 6.5365190000 1.8469780000 1.0504440000
H 7.1533420000 1.4041830000 1.8670450000
H 5.6429390000 1.1875620000 0.9625090000
C 7.3264500000 1.7859530000 -0.2765250000

H 7.4114700000 0.7241690000 -0.5974980000
H 8.3716660000 2.1170620000 -0.1061830000
C 6.7003020000 2.6078740000 -1.4255280000
H 7.4269060000 2.6755230000 -2.2686240000
H 5.8287050000 2.0445890000 -1.8304370000
C 6.1994700000 4.0273810000 -1.0343610000
H 5.6738470000 4.4618110000 -1.9142190000
C 7.3330270000 5.0330170000 -0.6356040000
H 8.0587280000 5.1311970000 -1.4759730000
H 6.8728900000 6.0421870000 -0.5220010000
C 8.0888050000 4.6844820000 0.6666620000
H 8.7007780000 5.5606200000 0.9763620000
H 8.8209320000 3.8762810000 0.4685330000
C 7.1665240000 4.2749800000 1.8365910000
H 6.6846960000 5.1918680000 2.2493310000
H 7.7798230000 3.8619590000 2.6708200000

9.14. $[(\{C_8H_{14}\}BCH_2CH_2SiMe_2)_2(\{C_8H_{14}\}BFCH_2CH_2SiMe_2)P_7]^-$

Charge = –1 Multiplicity = 1
P -0.1768540000 0.8965930000 0.4641230000
P -1.1692340000 -0.6307540000 -0.8053860000
P 0.4634900000 2.2877050000 -1.1572530000
P 1.7855690000 -0.1275790000 0.7583610000
P 1.6004650000 0.9443570000 -2.5435670000
P 2.2887600000 -0.8370330000 -1.3093380000
P 0.3212280000 -0.9277470000 -2.4423710000
Si -3.0674370000 0.3698450000 -1.7649930000
Si 2.1175660000 3.6332680000 -0.2565690000
Si 1.2730150000 -2.0974450000 1.8757980000
C 0.0142080000 -1.6464550000 3.2239320000
C 2.9171900000 -2.6065080000 2.6932840000
C 0.6352660000 -3.5109260000 0.7689370000
C 1.5150030000 4.1944260000 1.4526860000
C 2.1853340000 5.1246050000 -1.4352070000
C 3.8529640000 2.8361420000 -0.1437590000
C -4.0474380000 1.1511220000 -0.3572230000
C -3.9405680000 -1.1534280000 -2.4992210000
C -2.6422780000 1.5704000000 -3.1711900000
H -4.1956800000 0.3922020000 0.4441740000
C -5.4325590000 1.6815490000 -0.8071790000

H -3.4417400000 1.9639400000 0.1041380000
H -2.0226490000 2.4150350000 -2.8068380000
H -3.5903090000 1.9818120000 -3.5808090000
H -2.0948630000 1.0650670000 -3.9938600000
H -4.1767240000 -1.8953340000 -1.7086990000
H -3.3252800000 -1.6506150000 -3.2782910000
H -4.8989860000 -0.8360460000 -2.9632570000
H 2.8987470000 5.8916410000 -1.0643020000
H 1.1850330000 5.5976200000 -1.5204930000
H 2.5053670000 4.8202540000 -2.4539060000
H 4.0804300000 2.3791190000 -1.1332750000
H 3.7710250000 1.9845120000 0.5686540000
C 4.9842070000 3.8362330000 0.2741890000
H 2.2020430000 4.9591080000 1.8752450000
H 1.4650110000 3.3409510000 2.1597370000
H 0.5019290000 4.6426010000 1.3874200000
H 2.7759090000 -3.5067490000 3.3301930000
H 3.3107060000 -1.7917900000 3.3361410000
H 3.6904960000 -2.8445940000 1.9328500000
H -0.1749030000 -2.5198890000 3.8851410000
H -0.9526560000 -1.3313950000 2.7799940000
H 0.3859930000 -0.8148740000 3.8578210000
H -0.2949400000 -3.1424480000 0.2829640000
C 0.3828890000 -4.8539380000 1.4900420000
H 1.3636310000 -3.6469550000 -0.0624800000
H -5.9710000000 0.8897220000 -1.3844150000
H -5.3018500000 2.5353460000 -1.5117350000
H 4.7276300000 4.3179000000 1.2434760000
H 5.0722940000 4.6351550000 -0.4949240000
H -0.3377630000 -4.7207720000 2.3385150000
H 1.2995320000 -5.2219580000 2.0145460000
B -0.2073190000 -6.0682640000 0.6681720000
C -0.3797870000 -7.5141880000 1.3051010000
H -0.0539800000 -7.5522530000 2.3696260000
C -1.8939490000 -7.8835880000 1.2640430000
H -2.0442280000 -8.9220730000 1.6424350000
H -2.4218720000 -7.2246660000 1.9917110000
C -2.5717370000 -7.7341780000 -0.1163400000
H -3.6756430000 -7.7770690000 0.0165400000
H -2.3253820000 -8.6105930000 -0.7505770000

C -2.2059680000 -6.4303600000 -0.8603380000
H -2.5663930000 -6.4896100000 -1.9135150000
H -2.7731290000 -5.5874820000 -0.4036280000
C -0.6937320000 -6.0476860000 -0.8390030000
H -0.5965500000 -5.0458920000 -1.3096760000
C 0.2380800000 -7.0240910000 -1.6245930000
H -0.0921770000 -7.0876230000 -2.6875520000
H 1.2554720000 -6.5700060000 -1.6596560000
C 0.3523260000 -8.4472780000 -1.0333660000
H 1.2041930000 -8.9752880000 -1.5176480000
H -0.5456900000 -9.0380080000 -1.3065300000
C 0.5473190000 -8.4766780000 0.4995830000
H 1.6036190000 -8.2021030000 0.7284040000
H 0.4264520000 -9.5215360000 0.8710020000
B 6.2552740000 2.9121110000 0.3525720000
C 6.8404910000 2.2866880000 1.6876680000
H 6.1860290000 2.4688200000 2.5692370000
C 7.0450690000 0.7527830000 1.5474190000
H 7.5646360000 0.3483660000 2.4479500000
H 6.0362830000 0.2814370000 1.5496930000
C 7.7983500000 0.2971790000 0.2769290000
H 7.6607350000 -0.7996150000 0.1499680000
H 8.8906560000 0.4336750000 0.4195670000
C 7.3484010000 1.0113840000 -1.0176980000
H 8.0662250000 0.7725930000 -1.8373780000
H 6.3739420000 0.5794790000 -1.3407560000
C 7.1608140000 2.5498340000 -0.8984600000
H 6.7342690000 2.9201170000 -1.8575540000
C 8.4803820000 3.3528490000 -0.6446840000
H 9.2132340000 3.1430730000 -1.4587350000
H 8.2422080000 4.4385090000 -0.7319310000
C 9.1463250000 3.1032840000 0.7286100000
H 9.9231810000 3.8814530000 0.9022520000
H 9.7000060000 2.1427720000 0.7056960000
C 8.1570920000 3.1042590000 1.9169210000
H 7.8722680000 4.1583890000 2.1430130000
H 8.6738330000 2.7340270000 2.8332150000
C -7.9339070000 2.4750470000 0.0685610000
H -8.3800290000 1.6697000000 -0.5673540000
C -7.2176250000 4.9808230000 -0.1274250000

H -7.8906910000 5.4453660000 0.6246550000
H -7.0598050000 5.7733140000 -0.8976460000
C -8.7881620000 2.5524820000 1.3580690000
H -9.8503910000 2.8311800000 1.1340250000
H -8.8210380000 1.5260730000 1.7866800000
C -8.2480640000 3.5133640000 2.4465110000
H -8.7783300000 3.3119450000 3.4080420000
H -8.5228150000 4.5591470000 2.1867800000
C -7.9374330000 3.7721170000 -0.7756570000
H -7.4394850000 3.5493060000 -1.7479820000
H -8.9792330000 4.0862430000 -1.0438030000
B -6.3912990000 2.0667300000 0.5004550000
F -6.4211030000 0.8562440000 1.3397110000
C -5.8565170000 3.3488190000 1.3969020000
H -4.8024760000 3.1883190000 1.7348640000
C -6.7192740000 3.4262350000 2.6824720000
H -6.4124810000 4.2834130000 3.3358080000
H -6.5111820000 2.5019670000 3.2661020000
C -5.8638810000 4.6417560000 0.5449270000
H -5.0954460000 4.5292920000 -0.2556840000
H -5.5369720000 5.5312360000 1.1427010000

9.15. $[(\{C_8H_{14}\}BCH_2CH_2SiMe_2)(\{C_8H_{14}\}BFCH_2CH_2SiMe_2)_2P_7]^{2-}$

Charge = –2 Multiplicity = 1

P 0.0511980000 0.9239340000 0.5636920000
P -1.3840850000 -0.2168920000 -0.6920310000
P 1.1158770000 2.0812190000 -1.0144450000
P 1.5971600000 -0.6759530000 0.8047300000
P 1.7143830000 0.4837170000 -2.4553280000
P 1.8251090000 -1.4569580000 -1.2877710000
P -0.0877800000 -0.8945290000 -2.3848350000
Si -2.9438140000 1.2431950000 -1.6489100000
Si 3.1669130000 2.7826660000 -0.0851570000
Si 0.5082140000 -2.4224540000 1.8650150000
C -0.6110090000 -1.6678480000 3.2042570000
C 1.9037370000 -3.4069150000 2.7145620000
C -0.4778150000 -3.5977030000 0.7281410000
C 2.8034520000 3.1135980000 1.7537410000
C 3.3872990000 4.4559870000 -0.9747460000
C 4.6802910000 1.6802120000 -0.3535970000

C -4.1338880000 1.8162820000 -0.2924460000
C -3.8311180000 0.0876250000 -2.8799680000
C -2.1658430000 2.6774440000 -2.6166480000
H -4.6096040000 0.9134520000 0.1544230000
C -5.2354300000 2.7674490000 -0.8319370000
H -3.5637220000 2.2978840000 0.5337750000
H -1.5540050000 3.3283520000 -1.9599760000
H -2.9788970000 3.2888460000 -3.0652850000
H -1.5069990000 2.3070630000 -3.4287970000
H -4.2542190000 -0.7989260000 -2.3633430000
H -3.1463340000 -0.2642530000 -3.6796670000
H -4.6733760000 0.6379570000 -3.3519330000
H 4.3016440000 4.9649830000 -0.6020700000
H 2.5155630000 5.1232870000 -0.8103420000
H 3.5075400000 4.3072820000 -2.0686900000
H 4.8152690000 1.5208670000 -1.4479240000
H 4.4837480000 0.6735280000 0.0781780000
C 5.9717590000 2.3062230000 0.2377620000
H 3.6741370000 3.6358820000 2.2054930000
H 2.6518820000 2.1608760000 2.3018960000
H 1.9020190000 3.7456370000 1.8936320000
H 1.4915980000 -4.2437490000 3.3202930000
H 2.4919010000 -2.7517990000 3.3905370000
H 2.6058310000 -3.8346660000 1.9681590000
H -1.0410730000 -2.4614820000 3.8536660000
H -1.4463540000 -1.0997870000 2.7458130000
H -0.0390200000 -0.9705340000 3.8512060000
H -1.2150970000 -2.9715830000 0.1786690000
C -1.1855660000 -4.7688750000 1.4451370000
H 0.2225800000 -3.9799130000 -0.0484050000
H -5.6627630000 2.3498610000 -1.7782730000
H -4.7681500000 3.7368510000 -1.1234090000
H 5.8895140000 2.3414900000 1.3495530000
H 6.0516890000 3.3740500000 -0.0860600000
H -1.8703660000 -4.3988030000 2.2483760000
H -0.4539940000 -5.3875180000 2.0299740000
B -1.9913510000 -5.8260940000 0.5958950000
C -2.8952540000 -6.9463340000 1.2739250000
H -2.8256270000 -6.9327630000 2.3857150000
C -4.3684740000 -6.5863990000 0.8976230000

H -5.0646680000 -7.3588120000 1.3026280000
H -4.6321680000 -5.6424240000 1.4277950000
C -4.6319990000 -6.3909600000 -0.6131040000
H -5.6252270000 -5.9078730000 -0.7477440000
H -4.7202900000 -7.3806110000 -1.1075680000
C -3.5578560000 -5.5426410000 -1.3312330000
H -3.7098410000 -5.6027800000 -2.4341260000
H -3.7141610000 -4.4709130000 -1.0719060000
C -2.0770730000 -5.9078240000 -0.9839660000
H -1.4299940000 -5.1569420000 -1.4859640000
C -1.6269010000 -7.3202130000 -1.4580550000
H -1.7705780000 -7.4179150000 -2.5598040000
H -0.5265180000 -7.3951450000 -1.2974370000
C -2.3105120000 -8.5090960000 -0.7454110000
H -1.7479740000 -9.4430140000 -0.9721320000
H -3.3211900000 -8.6704130000 -1.1738770000
C -2.4216540000 -8.3466030000 0.7872590000
H -1.4204180000 -8.5401080000 1.2387030000
H -3.0889640000 -9.1425410000 1.1958020000
C -7.6929940000 3.9562890000 -0.4262170000
H -7.9996000000 3.5883280000 -1.4377850000
C -6.5207420000 6.0324240000 0.6494360000
H -7.3298980000 6.4177610000 1.3071500000
H -5.9408790000 6.9392980000 0.3527350000
C -8.9421160000 3.8600160000 0.4832830000
H -9.7812310000 4.5043570000 0.1102070000
H -9.3045860000 2.8095110000 0.4220590000
C -8.6934950000 4.1945020000 1.9753560000
H -9.5744800000 3.8626100000 2.5767820000
H -8.6622350000 5.2986270000 2.1072530000
C -7.1522570000 5.3943500000 -0.6133280000
H -6.3780230000 5.3666180000 -1.4149800000
H -7.9462520000 6.0880390000 -0.9962540000
B -6.5196880000 2.9694020000 0.2037720000
F -7.0890620000 1.6269880000 0.4257490000
C -6.1518300000 3.6521130000 1.6657020000
H -5.3500040000 3.0685880000 2.1816780000
C -7.4076050000 3.5656280000 2.5685630000
H -7.2254430000 4.0198120000 3.5774900000
H -7.6026300000 2.4844570000 2.7463050000

C -5.6067220000 5.0859410000 1.4668520000
H -4.6269480000 5.0078790000 0.9409930000
H -5.3766370000 5.5813870000 2.4456220000
C 7.5969870000 0.0179450000 0.2808090000
H 6.7232640000 -0.6260730000 0.0140230000
C 8.7871060000 0.9487950000 2.4169710000
H 9.7843720000 0.4654460000 2.3284650000
H 8.6202150000 1.0540280000 3.5162330000
C 8.8238400000 -0.5782360000 -0.4535890000
H 9.0365670000 -1.6285730000 -0.1235800000
H 8.5542730000 -0.6411750000 -1.5313420000
C 10.1314060000 0.2450220000 -0.3325960000
H 10.8675810000 -0.1244070000 -1.0873300000
H 10.6095500000 0.0394630000 0.6506000000
C 7.7098440000 0.0058200000 1.8240270000
H 6.7188340000 0.3025840000 2.2392440000
H 7.8960080000 -1.0274720000 2.2171010000
B 7.3716190000 1.5637100000 -0.2666760000
F 7.3307630000 1.5586920000 -1.7392910000
C 8.7285840000 2.3767010000 0.2292220000
H 8.6620370000 3.4503350000 -0.0798540000
C 9.9488430000 1.7733320000 -0.5079970000
H 10.9059430000 2.2804470000 -0.2153970000
H 9.8057670000 1.9813510000 -1.5918730000
C 8.8438200000 2.3563290000 1.7718870000
H 8.0050770000 2.9633120000 2.1855120000
H 9.7786140000 2.8663330000 2.1247830000

9.16. $[(\{C_8H_{14}\}BFCH_2CH_2SiMe_2)_3P_7]^{3-}$
Charge = –3 Multiplicity = 1
P 0.0673940000 0.4554300000 0.5499620000
P -0.5288750000 1.8084400000 -1.1179970000
P 1.8680330000 -0.4832480000 -0.3957520000
P -1.3984890000 -1.1919900000 0.1711750000
P 1.0365770000 -1.2370920000 -2.3264060000
P -1.2041000000 -1.4856840000 -2.0374700000
P -0.3752120000 0.4504870000 -2.8912330000
Si 1.0675760000 3.4738840000 -1.5138230000
Si 2.3976310000 -2.4552710000 0.7567880000
Si -3.5787900000 -0.3733770000 0.4363450000

C -4.1207690000 0.8088870000 -0.9484240000
C -3.5185280000 0.5618690000 2.0941430000
C -4.7444890000 -1.8740440000 0.5328600000
C 1.2203180000 -3.8951430000 0.3746120000
C 2.2539980000 -1.9824740000 2.5961740000
C 4.1904310000 -2.8953000000 0.2986830000
C 0.6984370000 4.9440690000 -0.3692380000
C 0.6880280000 3.9601030000 -3.3206730000
C 2.8742380000 2.9035530000 -1.4003110000
H -0.3631150000 5.2392270000 -0.5334580000
C 1.6156800000 6.1730600000 -0.6196520000
H 0.7558020000 4.6162090000 0.6938160000
H 3.1313130000 2.5761390000 -0.3726480000
H 3.5365540000 3.7542470000 -1.6729320000
H 3.0797700000 2.0517350000 -2.0797470000
H -0.3842530000 4.2187220000 -3.4457440000
H 0.9331860000 3.1409340000 -4.0284070000
H 1.2872620000 4.8567270000 -3.5902610000
H 2.6198760000 -2.8237540000 3.2236910000
H 1.2025110000 -1.7638920000 2.8752170000
H 2.8705140000 -1.0893580000 2.8292220000
H 4.8469230000 -2.0386010000 0.5732760000
H 4.2650710000 -2.9779160000 -0.8106200000
C 4.7099200000 -4.1917240000 0.9772970000
H 1.5039170000 -4.7741150000 0.9932680000
H 1.2917630000 -4.1912980000 -0.6929890000
H 0.1675470000 -3.6193820000 0.5897640000
H -4.5454600000 0.8825730000 2.3729530000
H -2.8715030000 1.4610540000 2.0297930000
H -3.1323290000 -0.0888180000 2.9063630000
H -5.1512370000 1.1701010000 -0.7401630000
H -4.1320410000 0.2869070000 -1.9284030000
H -3.4364180000 1.6780480000 -1.0283530000
H -4.6538190000 -2.4482910000 -0.4184760000
C -6.2248660000 -1.4865820000 0.7965850000
H -4.3865600000 -2.5658380000 1.3283260000
H 1.7105800000 6.3486060000 -1.7211320000
H 2.6497600000 5.9351740000 -0.2756650000
H 4.0860400000 -5.0537320000 0.6408930000
H 4.5360790000 -4.1229670000 2.0809270000

H -6.5674760000 -0.7927300000 -0.0074790000
H -6.2821250000 -0.8862030000 1.7389740000
C 1.9707210000 8.9053050000 -0.3714550000
H 2.1041330000 8.9523670000 -1.4818220000
C 3.4098150000 8.5503010000 1.7807510000
H 3.2465360000 9.5188600000 2.3022060000
H 4.4355080000 8.2310110000 2.0862500000
C 1.2005170000 10.1835390000 0.0404800000
H 1.7748590000 11.1164710000 -0.2052400000
H 0.2758340000 10.2142490000 -0.5780400000
C 0.7830070000 10.2455090000 1.5313010000
H 0.0355000000 11.0649800000 1.6691120000
H 1.6567730000 10.5543360000 2.1467260000
C 3.3822570000 8.7862340000 0.2495650000
H 3.9007520000 7.9314910000 -0.2433880000
H 4.0138210000 9.6861000000 0.0222780000
B 1.0398320000 7.5894220000 0.0280130000
F -0.3135710000 7.7847100000 -0.5258050000
C 0.9604300000 7.6389910000 1.682309000
H 0.3628530000 6.7781720000 2.0702870000
C 0.1983480000 8.9239470000 2.0911680000
H 0.1110480000 9.0215950000 3.2053080000
H -0.8403250000 8.8175320000 1.7065400000
C 2.3768400000 7.5155720000 2.2930980000
H 2.7600460000 6.4957690000 2.0591660000
H 2.3517890000 7.5723010000 3.4127480000
C 6.8066820000 -4.7741530000 -0.7953510000
H 6.4963700000 -3.9310370000 -1.4602870000
C 6.3219700000 -7.3265340000 -0.4833180000
H 7.3251470000 -7.7583810000 -0.6928860000
H 5.5999990000 -8.1104960000 -0.8175100000
C 8.3544300000 -4.8110390000 -0.8265080000
H 8.7474070000 -5.0022030000 -1.8601040000
H 8.7051840000 -3.7934560000 -0.5439890000
C 9.0108180000 -5.8295900000 0.1395850000
H 10.1028230000 -5.6054800000 0.2218670000
H 8.9656710000 -6.8473750000 -0.3081640000
C 6.1262650000 -6.0514970000 -1.3420910000
H 5.0333090000 -5.8478660000 -1.4181290000
H 6.4565620000 -6.2824830000 -2.3889340000

B 6.3290190000 -4.4868450000 0.7667290000
F 7.0529270000 -3.3097180000 1.2792590000
C 6.8436290000 -5.8309900000 1.5971900000
H 6.5482510000 -5.7467440000 2.6734000000
C 8.3903340000 -5.8621810000 1.5590790000
H 8.8058590000 -6.7501430000 2.1063270000
H 8.7446550000 -4.9628390000 2.1108060000
C 6.1697790000 -7.1086620000 1.0430260000
H 5.0822780000 -7.0498180000 1.2797620000
H 6.5361270000 -8.0318730000 1.5661480000
C -7.4342570000 -3.7394900000 -0.3531940000
H -6.4370920000 -4.1333040000 -0.6692590000
C -9.3308140000 -2.1908630000 -1.2750140000
H -10.1745010000 -2.9112590000 -1.3520590000
H -9.5146010000 -1.4522280000 -2.0925060000
C -8.2850260000 -4.9696150000 0.0480520000
H -8.4318230000 -5.6743720000 -0.8125140000
H -7.6994320000 -5.5290620000 0.8112900000
C -9.6735540000 -4.6450020000 0.6539860000
H -10.0909600000 -5.5663300000 1.1297610000
H -10.3881650000 -4.4013010000 -0.1635610000
C -7.9993380000 -2.9352320000 -1.5482070000
H -7.2328710000 -2.1828910000 -1.8447410000
H -8.1421110000 -3.5819920000 -2.4534640000
B -7.2747060000 -2.7601030000 0.9746830000
F -6.7996960000 -3.5655550000 2.1135140000
C -8.8271390000 -2.2579100000 1.2908040000
H -8.8292080000 -1.5669400000 2.1711510000
C -9.6654050000 -3.4952940000 1.6919290000
H -10.7289190000 -3.2226470000 1.9269040000
H -9.2280520000 -3.8885350000 2.6369080000
C -9.4030520000 -1.4657090000 0.0931440000
H -8.8357590000 -0.5099190000 0.0119830000
H -10.4687820000 -1.1609560000 0.2709820000

9.17. $[(\{C_8H_{14}\}BCH_2CH_2SiMe_2)_2(\{C_8H_{14}\}BHCH_2CH_2SiMe_2)P_7]^-$

Charge = –1 Multiplicity = 1

P	0.431745893	-0.790138475	0.609979980
P	1.261293862	0.832063018	-0.659877330
P	0.019304598	-2.274247307	-1.003085686

P	-1.652833246	-0.022491538	0.832261164
P	-1.235349122	-1.104489626	-2.443790734
P	-2.184615005	0.587212351	-1.259489029
P	-0.211956458	0.918059581	-2.337281461
Si	3.275727268	0.029838645	-1.579868694
Si	-1.483037338	-3.801398718	-0.122656149
Si	-1.447269253	2.017499226	1.919220301
C	-0.169084313	1.784415878	3.303942454
C	-3.167245244	2.299060872	2.689185168
C	-0.994189243	3.488208671	0.794713450
C	-0.914115253	-4.203331561	1.641859840
C	-1.287472398	-5.334808941	-1.229722121
C	-3.310179401	-3.234123411	-0.139941943
C	4.311516299	-0.685981813	-0.173434444
C	4.015490820	1.639362214	-2.277451173
C	2.985709465	-1.176432479	-3.015138689
H	4.396129095	0.070004383	0.640842528
C	5.725960729	-1.109617443	-0.647894984
H	3.771417022	-1.549257463	0.277912743
H	2.489218426	-2.104200638	-2.665083187
H	3.970867779	-1.450088948	-3.450441307
H	2.361398640	-0.724695124	-3.814054251
H	4.164036796	2.392590930	-1.476181646
H	3.373435905	2.085751386	-3.065580949
H	5.008886948	1.417125486	-2.722858616
H	-1.921084460	-6.171343290	-0.863893003
H	-0.232526976	-5.679229986	-1.236399067
H	-1.579705951	-5.114781830	-2.278009545
H	-3.551200772	-2.911118995	-1.178134970
H	-3.366074848	-2.314465370	0.484886180
C	-4.324113924	-4.323001646	0.353272995
H	-1.508475243	-5.044320069	2.059882726
H	-1.033567100	-3.325344372	2.309388243
H	0.155250099	-4.499565948	1.655523954
H	-3.180300158	3.229456163	3.297635249
H	-3.449087217	1.453803947	3.350935209
H	-3.948636345	2.392499334	1.905795143
H	-0.142997677	2.680383908	3.961574982
H	0.847694872	1.629624977	2.887931649
H	-0.416598222	0.906468499	3.935980822

H	-0.026350480	3.236095459	0.307972739
C	-0.912680500	4.857356763	1.506704002
H	-1.737812386	3.525637320	-0.033399453
H	6.216804431	-0.248530113	-1.165995485
H	5.621645183	-1.903271353	-1.428378832
H	-4.057042913	-4.645179753	1.383703693
H	-4.273208692	-5.211806415	-0.313101275
H	-0.166651805	4.824437937	2.343832564
H	-1.861497636	5.101482489	2.045208746
B	-0.487805794	6.135218582	0.680067263
C	-0.551306124	7.597962555	1.298627412
H	-0.894409200	7.597713524	2.358442635
C	0.881140472	8.209784801	1.267392672
H	0.855478254	9.263826769	1.631649170
H	1.500425706	7.655808772	2.010438525
C	1.592161429	8.155146139	-0.103425537
H	2.671976535	8.382602661	0.039611486
H	1.211522101	8.969312325	-0.754060307
C	1.455848761	6.798148450	-0.829896916
H	1.814634757	6.901448823	-1.880268495
H	2.148900235	6.067359114	-0.354000098
C	0.028250014	6.171446533	-0.816626992
H	0.101234817	5.159998216	-1.271024310
C	-1.041560984	6.969212669	-1.629371241
H	-0.710195514	7.070823062	-2.688997479
H	-1.968954728	6.352130136	-1.669574287
C	-1.398297804	8.362413437	-1.063017253
H	-2.319988272	8.733720285	-1.564566793
H	-0.608142713	9.091044153	-1.337033440
C	-1.614885189	8.382036177	0.467198075
H	-2.614045359	7.939949279	0.690290676
H	-1.673129527	9.437777333	0.822616114
B	-5.691621148	-3.550154578	0.271856689
C	-6.339992820	-2.744122048	1.474402753
H	-5.675788217	-2.699362823	2.366249359
C	-6.669131703	-1.285973059	1.047625091
H	-7.219723908	-0.760187217	1.862914053
H	-5.701698588	-0.744318996	0.944441374
C	-7.456975501	-1.142366234	-0.274244931
H	-7.410135641	-0.082128476	-0.608365714

H	-8.534397260	-1.337487025	-0.092144943
C	-6.948205284	-2.049724486	-1.417241233
H	-7.685152762	-2.035082199	-2.254535539
H	-6.016380136	-1.604327044	-1.833649699
C	-6.623570388	-3.515184768	-1.011992179
H	-6.162009768	-4.020426806	-1.890011045
C	-7.866781687	-4.368747146	-0.592971598
H	-8.610201478	-4.386384963	-1.424176720
H	-7.532088507	-5.425472985	-0.472030446
C	-8.561597765	-3.918664179	0.713204903
H	-9.270150755	-4.712703066	1.039770550
H	-9.195305142	-3.031223868	0.511544668
C	-7.584414929	-3.607155662	1.870460877
H	-7.213922962	-4.570320545	2.292832996
H	-8.137500512	-3.111876979	2.702683998
C	8.261707726	-1.913999126	0.113896846
H	8.687780609	-1.049649871	-0.454711403
C	7.597717440	-4.410303563	-0.307502375
H	8.297080844	-4.936453796	0.377751746
H	7.436539945	-5.124958162	-1.150191394
C	9.147828365	-2.096219009	1.370474987
H	10.209636856	-2.338799994	1.101598127
H	9.180976874	-1.112746077	1.893333601
C	8.649632274	-3.163313928	2.376892215
H	9.193862287	-3.038396627	3.343856865
H	8.945896337	-4.173123256	2.017430322
C	8.281340202	-3.128967097	-0.847417573
H	7.762950655	-2.827056728	-1.787669080
H	9.324917055	-3.394742015	-1.159708235
B	6.714255652	-1.581504545	0.606796635
H	6.730244902	-0.617583230	1.425826430
C	6.234003856	-2.960168508	1.390992274
H	5.183860490	-2.869813297	1.765977512
C	7.123924940	-3.143080905	2.645543179
H	6.858078038	-4.072219606	3.214837118
H	6.902741709	-2.291184805	3.328948783
C	6.253624637	-4.170586830	0.424408966
H	5.463173107	-4.004683301	-0.345210035
H	5.963209835	-5.121516849	0.942253126

9.18. [({C_8H_{14}}$BHCH_2CH_2SiMe_2)_3P_7]^{3-}$

Charge = –3 Multiplicity = 1

P	0.099946309	-0.329506021	0.257312658
P	0.141677565	-1.750948259	-1.457316392
P	-1.498181174	1.055058819	-0.475742892
P	1.888197004	0.892299556	-0.304880460
P	-0.734092645	1.614188379	-2.498384654
P	1.514454025	1.286244045	-2.475074565
P	0.132206827	-0.360761776	-3.212724499
Si	-1.859901537	-2.944085367	-1.667761077
Si	-1.363805004	3.072197316	0.716552473
Si	3.784952603	-0.490639951	-0.321098454
C	3.895211774	-1.588240670	-1.868057915
C	3.593967623	-1.583709632	1.226398766
C	5.301753090	0.649321064	-0.181337951
C	0.091879804	4.167127175	0.180091409
C	-1.127703753	2.532074898	2.527484497
C	-3.025614224	3.973519873	0.472346887
C	-1.882117123	-4.361173990	-0.401208388
C	-1.674826096	-3.653249594	-3.432652299
C	-3.436922532	-1.888580970	-1.607515284
H	-0.909249902	-4.901196468	-0.468745733
C	-3.053481962	-5.361370517	-0.613236330
H	-1.907874942	-3.935466769	0.628108063
H	-3.560141259	-1.398548719	-0.620784938
H	-4.315611707	-2.546058677	-1.787155085
H	-3.423010447	-1.088734342	-2.375883418
H	-0.737052588	-4.239453079	-3.530941872
H	-1.664073013	-2.849411900	-4.198303774
H	-2.526691848	-4.335355009	-3.643611075
H	-1.178654304	3.424431076	3.187929767
H	-0.145363647	2.037280740	2.674300629
H	-1.923975821	1.826093529	2.843525766
H	-3.839566516	3.264798451	0.748206189
H	-3.156302023	4.155388436	-0.619290091
C	-3.123145036	5.301223490	1.282949626
H	0.108857243	5.086493496	0.804391192
H	-0.016742609	4.476038066	-0.880774557
H	1.056923137	3.631116089	0.289398167
H	4.516493455	-2.187892284	1.365660099

H	2.729960519	-2.273520916	1.132609973
H	3.449823958	-0.966788646	2.138009751
H	4.800293918	-2.229669066	-1.796933709
H	3.985368712	-0.969096769	-2.785240306
H	2.996158847	-2.230734935	-1.965322191
H	5.296633459	1.365054487	-1.036042411
C	6.661486573	-0.101172592	-0.136754063
H	5.173176068	1.285751445	0.725057933
H	-3.082226675	-5.663426213	-1.690707203
H	-4.016485118	-4.816506978	-0.447351643
H	-2.253514179	5.941882774	0.992179957
H	-2.935798525	5.056829163	2.358806624
H	6.747032944	-0.740940538	-1.050679957
H	6.638891657	-0.828161903	0.714470277
B	7.999902786	0.881673401	-0.074598269
C	9.430472729	0.044963871	-0.218165948
H	9.418270589	-0.589953378	-1.139761877
C	10.586157316	1.058738501	-0.396125818
H	11.585053776	0.550939277	-0.484665077
H	10.418252921	1.577708370	-1.368142019
C	10.693427313	2.135575107	0.713115668
H	11.372339286	2.953816830	0.367592181
H	11.208485160	1.699424805	1.597727506
C	9.341475276	2.747555403	1.159754659
H	9.524795927	3.348923568	2.090482421
H	9.017207311	3.476842156	0.382097539
C	8.185373167	1.733802932	1.339503359
H	7.271916766	2.337786529	1.564193882
C	8.379206613	0.768306944	2.534921062
H	8.535937537	1.320877098	3.499055599
H	7.427053279	0.205250226	2.671629072
C	9.528384437	-0.259643099	2.378181962
H	9.419155532	-1.056267449	3.154001493
H	10.496618938	0.234086977	2.616196946
C	9.623254064	-0.918566316	0.978815009
H	8.834747683	-1.703472307	0.909877337
H	10.598707866	-1.472365601	0.919777963
B	-4.460373785	6.308099890	1.264558261
C	-4.871394771	7.016433271	-0.181460919
H	-3.965996359	7.438824615	-0.685343916

C	-5.494890245	5.998878763	-1.169699503
H	-5.823098040	6.493475390	-2.122792576
H	-4.697747935	5.283446447	-1.472138673
C	-6.691919028	5.184768627	-0.617586252
H	-6.885264837	4.314309624	-1.289256127
H	-7.615177273	5.802016465	-0.683688486
C	-6.521138289	4.678044597	0.836561639
H	-7.519321788	4.308865115	1.193507812
H	-5.867336940	3.777732525	0.816030599
C	-5.899102075	5.695386032	1.826059318
H	-5.747168354	5.150902702	2.791709201
C	-6.834567275	6.891128385	2.137772023
H	-7.840488563	6.553374021	2.507509453
H	-6.372607549	7.460691383	2.976639293
C	-7.066826821	7.869904679	0.957070031
H	-7.520496033	8.814786747	1.346610485
H	-7.842080434	7.446132024	0.281311978
C	-5.803946745	8.215513886	0.125416581
H	-5.204425154	8.961561519	0.695886445
H	-6.140859188	8.737293978	-0.811170614
C	-4.120837718	-7.871754789	-0.123873772
H	-4.097497837	-8.072308997	-1.225010374
C	-5.742981451	-6.847744635	1.654587506
H	-5.930302789	-7.729038013	2.307345259
H	-6.682973604	-6.247000079	1.713947895
C	-3.793528714	-9.212575583	0.575853688
H	-4.556400193	-10.006130526	0.348166334
H	-2.832050801	-9.578711310	0.146980156
C	-3.634140649	-9.129075805	2.115148024
H	-3.153246765	-10.067411262	2.486631786
H	-4.641558683	-9.124866411	2.587447736
C	-5.535659330	-7.329338213	0.196408658
H	-5.729235702	-6.468362172	-0.484871761
H	-6.336809343	-8.080277719	-0.040393552
B	-2.956606812	-6.756239887	0.285201605
H	-1.816510897	-7.260086215	0.063264869
C	-3.154025677	-6.564028793	1.922208723
H	-2.428813224	-5.815867097	2.326391158
C	-2.827553408	-7.906342449	2.621393662
H	-2.956097621	-7.844327955	3.735183123

H	-1.745570982	-8.109009768	2.448312167
C	-4.569557048	-6.020472052	2.237849781
H	-4.634552598	-4.987499064	1.824542474
H	-4.738404506	-5.907375655	3.341116552
H	7.908917531	1.698555171	-1.037277384
H	-4.114838663	7.240250641	2.053803532

9.19. $ClSiMe_2CH_2CH_2CH_2B\{C_8H_{14}\}$ (**2**)

Charge = 0 Multiplicity = 1

Cl	-5.321159978	-0.934625740	-0.000252827
Si	-3.582219629	0.271623348	0.000074883
C	-2.102953272	-0.918388301	0.000168006
C	-3.637214338	1.335847475	1.558563013
C	-3.636876977	1.336091219	-1.558259974
H	-2.205957554	-1.585199636	0.886254330
C	-0.709447347	-0.251946900	-0.000067836
H	-2.206105856	-1.585533849	-0.885651973
H	-3.641000839	0.705646786	-2.471421094
H	-2.755132835	2.010373400	-1.608235198
H	-4.550014125	1.966433278	-1.574140483
H	-3.641064658	0.705321876	2.471665479
H	-4.550511991	1.965949165	1.574572657
H	-2.755649402	2.010378273	1.608519583
H	-0.616992816	0.415518368	0.887039550
H	-0.617272338	0.415476771	-0.887229577
C	0.466241757	-1.271796019	-0.000238717
H	0.360068208	-1.923103742	0.897213591
H	0.360135904	-1.922849859	-0.897856801
H	4.502813070	-0.913310279	-2.173361786
C	3.843253696	-1.107618746	-1.296018637
H	2.067082769	-0.293119241	-2.241213195
C	2.642567584	-0.098686820	-1.308434257
H	3.432647653	-2.131618533	-1.456048489
C	3.053619247	1.400110215	-1.297223473
H	2.130291902	2.001872617	-1.463514947
B	1.840500951	-0.487411766	-0.000111359
C	2.642022658	-0.098023845	1.308308445
H	2.066186084	-0.291998484	2.240974451
H	2.129591689	2.002610200	1.462004389
C	3.053011794	1.400786747	1.296451181

H	3.711447878	1.626548240	2.167297886
H	3.432112565	-2.130819058	1.457334241
C	3.842757275	-1.106904293	1.296896119
C	3.734421854	1.891395875	-0.000357364
H	3.754551825	3.003762908	-0.000649642
H	4.801248104	1.587506849	-0.000013669
H	4.501986606	-0.912103178	2.174381124
H	5.374301598	-0.239837321	0.000483890
C	4.685742639	-1.108821280	0.000597926
H	5.348194585	-2.002610480	0.000992759
H	3.712452203	1.625402513	-2.167897200

9.20. $[ClSiMe_2CH_2CH_2CH_2FB\{C_8H_{14}\}]^-$

Charge = –1 Multiplicity = 1

Cl	5.477759983	-0.829036410	-0.549166941
Si	3.726752155	0.169249263	0.177275569
C	2.205708428	-0.556478085	-0.662799278
C	3.975535752	2.001839205	-0.223616447
C	3.725763652	-0.099207410	2.051139342
H	2.319763302	-0.426825114	-1.764212944
C	0.867039243	0.071641328	-0.188570211
H	2.199485854	-1.658637961	-0.494991707
H	3.640797783	-1.178862853	2.294008436
H	2.858366368	0.421772770	2.509636026
H	4.655484717	0.288929445	2.516998702
H	4.060822271	2.155937382	-1.319387771
H	4.892731960	2.402825847	0.255941888
H	3.103645791	2.589659755	0.134504417
H	0.897240653	1.175922987	-0.332304750
H	0.765537479	-0.078681182	0.911971286
C	-0.392667467	-0.460642397	-0.890887420
H	-0.250890870	-0.344050183	-1.995475827
H	-0.459325413	-1.562366490	-0.719153739
H	-2.899960959	-1.453207902	2.486776373
C	-2.558294567	-1.313529758	1.428020044
H	-1.396101094	0.461318672	1.810764482
C	-2.229764508	0.168190479	1.124064432
H	-1.611316402	-1.896925349	1.346200161
C	-3.393167003	1.150601676	1.407910933
H	-2.983192137	2.178139167	1.286963869

B	-1.776183976	0.343199891	-0.461462348
C	-3.105722298	-0.145419924	-1.318691363
H	-2.894475418	-0.080512335	-2.415670239
H	-3.953627105	1.830366175	-1.423947343
C	-4.265102411	0.838251752	-1.027277765
H	-5.198725545	0.558910994	-1.582045518
H	-2.604808369	-2.251078509	-1.414329086
C	-3.434643493	-1.625768275	-1.010090792
C	-4.618876802	1.010265060	0.471033966
H	-5.275774948	1.905317671	0.592026895
H	-5.245668716	0.154381861	0.805434364
H	-4.353688418	-1.968071468	-1.553777165
H	-4.625998003	-1.659178685	0.819736501
C	-3.608386111	-1.963001288	0.492096517
H	-3.586533556	-3.071336874	0.626483695
H	-3.748922485	1.078965772	2.468891087
F	-1.524992378	1.774995788	-0.733749130

9.21. $[ClSiMe_2CH_2CH_2CH_2HB\{C_8H_{14}\}]^-$

Charge = –1 Multiplicity = 1

Cl	5.379197017	-0.997639029	-0.043681348
Si	3.653035822	0.269944386	0.021353147
C	2.117107392	-0.753008308	-0.350636211
C	3.963122440	1.621811489	-1.266970283
C	3.632480106	1.015535163	1.761303252
H	2.256218762	-1.251079153	-1.338314568
C	0.794216378	0.062339114	-0.345675177
H	2.060744777	-1.582718721	0.391840008
H	3.516882109	0.221242560	2.527836220
H	2.776179135	1.715430882	1.865835693
H	4.569139310	1.571095142	1.976000882
H	4.047791096	1.184112746	-2.283376816
H	4.896925478	2.181838846	-1.051398400
H	3.115464520	2.339722223	-1.275921321
H	0.865250930	0.892189037	-1.087337113
H	0.681606322	0.566786056	0.643047112
C	-0.478261540	-0.748499993	-0.644576276
H	-0.336925207	-1.258018435	-1.632234133
H	-0.532696817	-1.583141814	0.100569875
H	-3.003546829	0.095100075	2.759220618

C	-2.657767714	-0.315573477	1.774223739
H	-1.503327735	1.413703461	1.219619783
C	-2.327684588	0.810028639	0.762444412
H	-1.709262533	-0.857510972	1.999439751
C	-3.496400002	1.795528167	0.517626832
H	-3.091625773	2.635402408	-0.093081559
B	-1.862582343	0.161636198	-0.695882439
C	-3.195677345	-0.694517871	-1.189664414
H	-2.994401243	-1.197876599	-2.168651712
H	-4.070856858	0.942310177	-2.286879445
C	-4.364522814	0.290470971	-1.431900427
H	-5.301088814	-0.237852354	-1.753951054
H	-2.691689806	-2.558703478	-0.208484366
C	-3.525230616	-1.818788777	-0.175982677
C	-4.716840242	1.201142379	-0.229132858
H	-5.374669852	2.033720779	-0.578125282
H	-5.345049420	0.631509694	0.490448924
H	-4.442359067	-2.393214856	-0.472125060
H	-4.722709580	-0.931055545	1.420554422
C	-3.702261213	-1.353378424	1.291439899
H	-3.677243400	-2.243022827	1.966378725
H	-3.864522002	2.257167439	1.472160756
H	-1.653847843	1.106789795	-1.515019737

9.22. ($\{C_8H_{14}\}BCH_2CH_2CH_2SiMe_2)_3P_7$ (**3**)

Charge = 0 Multiplicity = 1

P	0.914607695	-1.725778838	0.704889687
P	0.479746038	-1.220549055	-1.434409378
Si	-0.459226392	-3.562695560	1.101301428
C	-0.932153217	-3.495059633	2.938095154
H	-0.035231125	-3.388678321	3.582750674
H	-1.453079296	-4.431653208	3.232517263
H	-1.610140617	-2.642189635	3.146935839
C	0.686628468	-5.044376220	0.780558610
H	1.014850392	-5.077950802	-0.279461387
H	0.162240886	-5.998327254	1.004913364
H	1.594057915	-4.994581234	1.417359208
C	-1.991412217	-3.650615180	-0.025947153
H	-2.574735252	-2.714683478	0.127430858
H	-1.640747113	-3.611887671	-1.083144484

C	-2.893921602	-4.886006567	0.185744447
H	-3.241936448	-4.918199748	1.242767647
H	-2.297745714	-5.816233341	0.043859613
C	-4.118156064	-4.926521589	-0.748660736
H	-3.797672913	-4.839674019	-1.815251820
H	-4.727899523	-3.995453820	-0.605446682
B	-5.177014588	-6.090253492	-0.614023740
C	-5.196942996	-7.215139963	0.506133024
H	-4.493971995	-7.020305208	1.343561969
C	-4.642957975	-8.458811381	-0.278349520
H	-3.595105513	-8.254173512	-0.599935534
H	-4.580185129	-9.332324478	0.410677278
C	-5.488768140	-8.819221570	-1.513334545
H	-5.011235114	-9.659202239	-2.063702555
H	-6.477596103	-9.207381496	-1.182301314
C	-5.698466214	-7.624616472	-2.462388192
H	-6.376731057	-7.914496911	-3.297624982
H	-4.726241595	-7.363026377	-2.941546935
C	-6.272942835	-6.358308915	-1.734104420
H	-6.327959541	-5.542860004	-2.487773094
C	-7.689136091	-6.614715295	-1.121264582
H	-8.407933106	-5.866361720	-1.518915471
H	-8.083425847	-7.604290562	-1.448564832
C	-7.697379614	-6.548928879	0.418069477
H	-8.708710998	-6.794667998	0.810138649
H	-7.501458034	-5.494413509	0.729823783
C	-6.636173962	-7.457027836	1.069140654
H	-6.932590269	-8.523065776	0.936649779
H	-6.638539913	-7.285585468	2.167050596
P	-1.934302027	0.066377384	0.718359068
P	-1.295123200	0.193047467	-1.425535970
Si	-2.834025558	2.173957359	1.126236424
C	-2.521786539	2.550443529	2.959958906
H	-2.871818355	1.720837555	3.608467995
H	-3.069389078	3.470186096	3.259129211
H	-1.441910556	2.711040866	3.157721562
C	-4.692945858	1.920863624	0.823235881
H	-4.896265626	1.654812523	-0.235269211
H	-5.256149272	2.850621308	1.054603732
H	-5.095910274	1.108367008	1.462732934

C	-2.158172536	3.546276598	-0.007979804
H	-1.055563670	3.592338900	0.139293265
H	-2.303544692	3.218466559	-1.063272968
C	-2.786566344	4.941591578	0.202527308
H	-2.642772725	5.262000930	1.258935700
H	-3.890070087	4.883137722	0.062055930
C	-2.217480087	6.024151315	-0.734124782
H	-2.299215223	5.698522836	-1.799637546
H	-1.107204046	6.096898952	-0.590629518
B	-2.713346560	7.518757262	-0.606783633
C	-3.695143567	8.089886731	0.502451326
H	-3.875236931	7.384503512	1.340954459
C	-5.043965600	8.208463340	-0.295672489
H	-5.373425333	7.192149964	-0.614736188
H	-5.843407069	8.583310384	0.384090185
C	-4.935303328	9.115749481	-1.534870943
H	-5.896253778	9.105180859	-2.094318909
H	-4.795260448	10.170339941	-1.208882570
C	-3.781621459	8.711103461	-2.470682876
H	-3.696479665	9.439190659	-3.309859629
H	-4.022348047	7.731392376	-2.945334135
C	-2.402812428	8.600796199	-1.728742982
H	-1.657214559	8.248002829	-2.474113061
C	-1.942498898	9.965568349	-1.119137331
H	-0.935665552	10.227203953	-1.509779242
H	-2.612212538	10.789893997	-1.456113419
C	-1.893802409	9.948083856	0.421092317
H	-1.618485288	10.952652408	0.810864617
H	-1.071517502	9.264118505	0.743071381
C	-3.209795101	9.467008766	1.063343496
H	-3.994660827	10.245068924	0.920116864
H	-3.068532435	9.391038281	2.162939926
P	0.009787366	-0.011152225	1.802206807
P	1.039807820	1.636558659	0.713696572
P	0.816808563	1.023035584	-1.429524183
Si	3.316502661	1.364961188	1.111712202
C	3.491999200	0.901576742	2.943883549
H	2.947319121	1.615536574	3.595731329
H	4.562857378	0.918888448	3.241093824
H	3.095623961	-0.115918945	3.139268379

C	4.024634119	3.102712671	0.812295518
H	3.891233234	3.416158083	-0.244366643
H	5.112428679	3.125051730	1.039141728
H	3.524955454	3.854915953	1.457114024
C	4.166145048	0.096003940	-0.025819481
H	3.653914537	-0.881892121	0.119446912
H	3.956451838	0.387926643	-1.080828682
C	5.688029622	-0.058748351	0.187630654
H	5.889455438	-0.347435462	1.243781148
H	6.190108762	0.926344158	0.052288699
C	6.344290057	-1.090110361	-0.749940864
H	6.108155505	-0.854516242	-1.816187479
H	5.850844443	-2.087813574	-0.609856216
B	7.885148212	-1.411537014	-0.614895486
C	8.864218993	-0.855165484	0.503838616
H	8.338822239	-0.347776043	1.340260845
C	9.653305987	0.252552349	-0.283326991
H	8.944565510	1.050564898	-0.605929017
H	10.374256225	0.751825253	0.404173910
C	10.392673921	-0.294172597	-1.518693195
H	10.873530514	0.542886210	-2.070618504
H	11.228973792	-0.949027008	-1.187791031
C	9.469896169	-1.082666033	-2.465523133
H	10.063410894	-1.520540532	-3.300796649
H	8.749847544	-0.379031184	-2.944788797
C	8.672310800	-2.220112591	-1.734624977
H	7.997664207	-2.682993995	-2.487339566
C	9.613642406	-3.308067391	-1.120971539
H	9.335031167	-4.308121018	-1.517295596
H	10.666078745	-3.143984840	-1.448863315
C	9.561962268	-3.346855384	0.418752769
H	10.288215232	-4.092089396	0.811237973
H	8.554525258	-3.714121191	0.731485029
C	9.804223476	-1.970436583	1.068311592
H	10.873051873	-1.684045972	0.935888944
H	9.657552529	-2.058330829	2.166307869

9.23. $[(\{C_8H_{14}\}BCH_2CH_2CH_2SiMe_2)_2(\{C_8H_{14}\}BFCH_2CH_2CH_2SiMe_2)P_7]^-$

Charge = –1 Multiplicity = 1

P	2.072802297	-0.497318541	0.718740420

P	1.440443004	-0.481018841	-1.432105203
Si	2.441403763	-2.751574282	1.122602627
C	2.022189017	-3.070841924	2.948138859
H	2.537718431	-2.346007610	3.611833579
H	2.340801607	-4.093468866	3.245414709
H	0.930618333	-2.981323152	3.125143251
C	4.316509568	-2.960395842	0.859718176
H	4.600532862	-2.739578823	-0.190816134
H	4.634658169	-4.000833484	1.087986868
H	4.889493083	-2.274039769	1.517424858
C	1.491092009	-3.934320248	-0.031069945
H	0.407385663	-3.706061256	0.084184800
H	1.742241304	-3.658374834	-1.081225322
C	1.750294641	-5.438652832	0.197367887
H	1.493605278	-5.707461063	1.246928885
H	2.838832033	-5.655483580	0.097290257
C	0.965663197	-6.354255682	-0.762214606
H	1.159499871	-6.064984008	-1.823765943
H	-0.132513751	-6.147067760	-0.657908791
B	1.059421669	-7.921719175	-0.617575732
C	1.793708723	-8.715674389	0.546997552
H	2.094615391	-8.069897431	1.398699661
C	3.113202171	-9.182754539	-0.165115123
H	3.708973921	-8.287195495	-0.458349132
H	3.748634375	-9.742257032	0.559925839
C	2.852862226	-10.045846549	-1.413948094
H	3.817385538	-10.287200111	-1.912575385
H	2.427585178	-11.025926153	-1.102071201
C	1.899233371	-9.371704050	-2.417735522
H	1.683195690	-10.065027530	-3.263333582
H	2.410523080	-8.491698742	-2.872776737
C	0.553395215	-8.903555702	-1.762710658
H	-0.030091085	-8.379254821	-2.550346447
C	-0.278566820	-10.097378623	-1.189839031
H	-1.293529752	-10.094812642	-1.642283013
H	0.175892139	-11.070233953	-1.489689154
C	-0.414440272	-10.052278645	0.344792654
H	-0.961266221	-10.948941976	0.711374108
H	-1.052061760	-9.177673221	0.619117246
C	0.939077904	-9.916827843	1.069397718

H	1.505878089	-10.871747293	0.971216881
H	0.754526999	-9.794964885	2.158516803
P	-1.196074872	-1.351978951	0.689233353
P	-0.806809347	-0.804802421	-1.444484707
Si	-3.383751712	-0.575649405	1.073456592
C	-3.443272294	-0.257254244	2.945195792
H	-3.112371246	-1.140378734	3.530162251
H	-4.488620827	-0.016274162	3.234700876
H	-2.807288751	0.607917988	3.224592331
C	-4.409687844	-2.121450255	0.654552013
H	-4.332452324	-2.374863730	-0.423632357
H	-5.480062116	-1.922232764	0.875950111
H	-4.086809589	-3.004488732	1.244156298
C	-3.942075177	0.921026742	0.062563029
H	-3.290806147	1.785166088	0.324454153
H	-3.757274802	0.722012561	-1.018615456
C	-5.435306315	1.277055856	0.303878381
H	-5.621469760	1.413785907	1.393455943
H	-6.070880312	0.413169666	-0.000419183
C	-5.923381044	2.545362322	-0.414886115
H	-5.789324202	2.408112804	-1.514216181
H	-5.241032102	3.387729840	-0.136821253
B	-7.467171741	2.977424089	0.020024781
C	-8.649547817	1.911227491	-0.457383838
H	-8.449506011	0.885707505	-0.062787812
C	-8.645213538	1.815520504	-2.004523049
H	-7.667793261	1.401026702	-2.344560644
H	-9.422213436	1.096551554	-2.372911262
C	-8.875144069	3.176125791	-2.694939701
H	-8.779113798	3.066153006	-3.801260042
H	-9.932438678	3.487903802	-2.524827874
C	-7.941981002	4.295931695	-2.189200966
H	-8.245840135	5.257315801	-2.679077036
H	-6.906422931	4.091718745	-2.546841065
C	-7.934064722	4.424758733	-0.645023399
H	-7.198271274	5.221892645	-0.380404058
C	-9.342856217	4.858030019	-0.116713369
H	-9.248900796	5.762396435	0.527832697
H	-9.992474366	5.183435360	-0.968390439
C	-10.082910017	3.773941525	0.701169528

H	-11.139413083	4.087902996	0.881084501
H	-9.591320069	3.707090833	1.693031720
C	-10.054357306	2.363091621	0.068328924
H	-10.808998248	2.315090069	-0.757111639
H	-10.427052398	1.642663862	0.832738989
P	0.179552409	0.005912621	1.785545169
P	-0.297608698	1.886037146	0.688610459
P	0.028871070	1.301035348	-1.448862431
Si	1.457548389	3.348742489	1.095738066
C	1.916252283	3.171785987	2.931425706
H	1.018742242	3.264089427	3.577568563
H	2.633873408	3.966970172	3.228845545
H	2.384935508	2.186453023	3.132613821
C	0.694282736	5.062170467	0.788564601
H	0.380396252	5.178221202	-0.270028148
H	1.423462257	5.868537114	1.020516600
H	-0.202961807	5.216036399	1.423135400
C	2.978451254	3.097709681	-0.028539800
H	3.333760412	2.052710342	0.118700139
H	2.627486657	3.145659421	-1.085232035
C	4.138514138	4.092556522	0.187356619
H	4.485868476	4.039964861	1.244011018
H	3.771824795	5.135760438	0.051363859
C	5.339242335	3.856521096	-0.749334035
H	5.005587417	3.846861314	-1.815626303
H	5.718345816	2.808996533	-0.611193494
B	6.637918394	4.741116926	-0.614662516
C	6.928474487	5.818454880	0.516416802
H	6.207804079	5.777169423	1.360018233
C	6.663858504	7.164220412	-0.249313911
H	5.593864647	7.206109818	-0.558896695
H	6.808531966	8.022027845	0.447385218
C	7.555917253	7.337747369	-1.492781894
H	7.275167343	8.270371945	-2.029708944
H	8.611197019	7.487371283	-1.172022475
C	7.479359328	6.137785727	-2.454785418
H	8.197144715	6.276645012	-3.296188081
H	6.468161190	6.109031664	-2.922957669
C	7.757460176	4.766837195	-1.745303737
H	7.616959436	3.968916391	-2.506603369

C	9.200488169	4.685469620	-1.148838669
H	9.725432423	3.797413616	-1.562518184
H	9.807260001	5.563276826	-1.471353978
C	9.209910646	4.600929025	0.390159568
H	10.255011582	4.604062537	0.771579207
H	8.780781817	3.615749226	0.694139779
C	8.390373893	5.719364800	1.063257680
H	8.921860366	6.690963069	0.936523480
H	8.365363177	5.538617060	2.159552991
F	-7.433798119	3.077894872	1.496826755

9.24. $[(\{C_8H_{14}\}BCH_2CH_2CH_2SiMe_2)(\{C_8H_{14}\}BFCH_2CH_2CH_2SiMe_2)_2P_7]^{2-}$

Charge = –2 Multiplicity = 1

P	-1.710940581	1.260669761	0.492194377
P	-0.988877240	0.951087079	-1.606628346
Si	-1.114466612	3.456741877	0.909907124
C	-0.743001646	3.600773044	2.770194823
H	-1.566677216	3.165854958	3.373699133
H	-0.626499512	4.666208447	3.066118392
H	0.191511622	3.061105629	3.028182152
C	-2.690885557	4.447867363	0.508211772
H	-2.956934462	4.355169241	-0.565889286
H	-2.554975414	5.527547096	0.736917166
H	-3.553131232	4.075152983	1.099310774
C	0.340673660	4.125550253	-0.130635875
H	1.210705221	3.456058322	0.056012764
H	0.081461764	3.983070818	-1.205071314
C	0.727627930	5.595594739	0.130743662
H	0.980866608	5.729925590	1.206843338
H	-0.151628858	6.257040900	-0.047410207
C	1.908919093	6.090810133	-0.726994569
H	1.707976177	5.910637504	-1.811269407
H	2.800991663	5.433835308	-0.543376207
B	2.479779893	7.544836334	-0.528515352
C	2.057518457	8.572846380	0.609458031
H	1.438409900	8.111908918	1.407309142
C	1.129860574	9.563700351	-0.179022842
H	0.237083112	9.010706845	-0.552381428
H	0.733739271	10.338108550	0.518382981
C	1.838696118	10.239144414	-1.367986415

H	1.115083723	10.872415643	-1.927422215
H	2.612645532	10.941671398	-0.984759934
C	2.496831016	9.227359313	-2.324668691
H	3.058437877	9.767184032	-3.122422508
H	1.700465856	8.652950110	-2.852219990
C	3.452608288	8.224897098	-1.590728999
H	3.822683137	7.505333147	-2.352904303
C	4.661082085	8.944438084	-0.907595218
H	5.613344561	8.510633074	-1.282203433
H	4.691728903	10.020460064	-1.198992052
C	4.633081469	8.836219749	0.629734140
H	5.476790266	9.409992775	1.073737940
H	4.810654023	7.771189415	0.913450760
C	3.294177133	9.288804094	1.244863760
H	3.199673676	10.395488721	1.145277709
H	3.315610421	9.092271104	2.338606331
P	1.596518262	0.668952699	0.711575349
P	1.181655020	0.289812278	-1.454136667
Si	3.253649856	-0.890478408	1.275340025
C	2.897321079	-1.383111713	3.076703255
H	2.811507982	-0.498489168	3.741451180
H	3.727115631	-2.022666462	3.447572235
H	1.956488381	-1.966709709	3.148212945
C	4.816146513	0.199417912	1.225841807
H	5.029360816	0.544616509	0.192050717
H	5.694448595	-0.385608300	1.572856260
H	4.707912826	1.094688840	1.873322784
C	3.455764698	-2.402860307	0.150917602
H	2.579693659	-3.071914550	0.310437316
H	3.398097501	-2.084572654	-0.915369927
C	4.785453650	-3.161272696	0.420640031
H	4.885216114	-3.385102051	1.507718234
H	5.638102436	-2.483725380	0.182889380
C	4.964153802	-4.482550556	-0.346683775
H	4.873448260	-4.273463957	-1.439421015
H	4.097684255	-5.147921635	-0.104078351
B	6.361298766	-5.284954703	0.055857964
C	7.763336107	-4.524353500	-0.420326078
H	7.818601686	-3.489448909	-0.004564750
C	7.768266663	-4.397672959	-1.965033281

H	6.917135066	-3.752164077	-2.282461212
H	8.693153557	-3.880039812	-2.331500380
C	7.655448085	-5.757849321	-2.684819202
H	7.576890188	-5.602933898	-3.787382638
H	8.607933636	-6.319973675	-2.537635281
C	6.484505994	-6.629959410	-2.185969413
H	6.540931847	-7.624717704	-2.700658651
H	5.525473409	-6.172494568	-2.520945586
C	6.463583543	-6.788106412	-0.644947118
H	5.561499902	-7.391688205	-0.383671747
C	7.733391819	-7.558153027	-0.148992657
H	7.433299105	-8.429519727	0.478252868
H	8.277992256	-8.008751264	-1.017698805
C	8.719776253	-6.702200942	0.679640142
H	9.672438126	-7.264200568	0.838093734
H	8.266415128	-6.542100058	1.678967040
C	9.023633004	-5.310617391	0.076746141
H	9.762062866	-5.424752457	-0.757558804
H	9.563869793	-4.718446539	0.851641095
P	-0.294490503	0.011976625	1.681766023
P	-0.580091197	-1.904321717	0.590224745
P	-0.471877017	-1.259214428	-1.547158989
Si	-2.812789728	-2.552560921	0.896116247
C	-3.034411954	-2.503468650	2.784666217
H	-2.238543723	-3.073323823	3.307817026
H	-4.017751741	-2.947863645	3.049475101
H	-3.021179052	-1.459517955	3.160964963
C	-2.774868008	-4.362483397	0.309397548
H	-2.574267412	-4.425760608	-0.780735312
H	-3.762702298	-4.834873238	0.498762329
H	-1.995537656	-4.949564348	0.837925149
C	-4.164276619	-1.572431134	-0.002192011
H	-4.083527737	-0.503874746	0.299087804
H	-3.947478600	-1.588784315	-1.095680544
C	-5.599010578	-2.101732110	0.274376805
H	-5.794293176	-2.101002571	1.370637323
H	-5.664294170	-3.170585834	-0.037308868
C	-6.726627763	-1.304399831	-0.405770321
H	-6.568965964	-1.338956444	-1.510356352
H	-6.604366652	-0.226961259	-0.128180856

B	-8.249279469	-1.773000361	0.058734569
C	-8.679702454	-3.313008017	-0.400368711
H	-7.950042171	-4.063629620	-0.012487504
C	-8.650865575	-3.402492170	-1.947395116
H	-7.610959095	-3.223187445	-2.305569895
H	-8.920761424	-4.430427819	-2.304748014
C	-9.593230823	-2.388508830	-2.629190819
H	-9.473339314	-2.438007112	-3.737803175
H	-10.648207106	-2.696506404	-2.436694779
C	-9.404818904	-0.937845995	-2.138481063
H	-10.189214636	-0.297209840	-2.620709986
H	-8.429424846	-0.553559625	-2.515773397
C	-9.439813660	-0.813296107	-0.594696827
H	-9.247379676	0.257268546	-0.341942872
C	-10.849838561	-1.205574408	-0.038454239
H	-11.248237855	-0.389236680	0.608013874
H	-11.588081464	-1.288861075	-0.876661003
C	-10.872572809	-2.511923839	0.789380301
H	-11.928424934	-2.817178280	0.990728477
H	-10.404011650	-2.294613365	1.770741954
C	-10.096708790	-3.688298659	0.151789335
H	-10.720442711	-4.142595909	-0.659852840
H	-10.008373761	-4.490115689	0.921188035
F	6.329155429	-5.413278466	1.530798458
F	-8.260408437	-1.660174591	1.534755413

9.25. $[(\{C_8H_{14}\}BFCH_2CH_2CH_2SiMe_2)_3P_7]^{3-}$

Charge = –3 Multiplicity = 1

P	1.546731700	1.183190517	-0.471602621
P	0.894461266	0.907124271	1.648714062
Si	1.139235928	3.437006492	-0.940937486
C	0.915373329	3.491431006	-2.831050243
H	1.748505640	2.980542663	-3.357308630
H	0.879841707	4.549378726	-3.169341855
H	-0.035260724	3.002980170	-3.129727323
C	2.817402355	4.222990221	-0.492873432
H	3.003195635	4.166300399	0.600559672
H	2.825050347	5.295788787	-0.782213617
H	3.655390661	3.709610696	-1.008623579
C	-0.271682448	4.344140357	-0.047196719

H	-1.235193946	3.868361151	-0.339119435
H	-0.173906323	4.165893197	1.048727510
C	-0.298291964	5.869455343	-0.347029848
H	-0.356495668	6.036313290	-1.446792941
H	0.674156908	6.317258149	-0.034850676
C	-1.446838032	6.657379893	0.311317596
H	-1.386361105	6.510004770	1.416537554
H	-2.413528844	6.180848319	0.009794702
B	-1.509154082	8.254901342	-0.132974100
C	-0.216366406	9.176625185	0.370584510
H	0.745447514	8.746164894	0.002674291
C	-0.161560446	9.163771893	1.919026394
H	0.011561982	8.120666961	2.269831889
H	0.705830892	9.761205705	2.305254199
C	-1.450728686	9.701121967	2.574994279
H	-1.391310796	9.591702135	3.684464410
H	-1.512337793	10.800815527	2.394181398
C	-2.739505405	9.039349040	2.044289311
H	-3.619546147	9.555334746	2.511746300
H	-2.779906538	7.987067591	2.407168461
C	-2.830155765	9.048028817	0.498048825
H	-3.766470919	8.509557310	0.215597671
C	-2.922160500	10.514280441	-0.043123963
H	-3.808734750	10.622480254	-0.710909395
H	-3.113388552	11.228346294	0.798979926
C	-1.679833309	10.985408480	-0.835417896
H	-1.743052490	12.085168611	-1.026390086
H	-1.702857747	10.480638103	-1.822501993
C	-0.326279717	10.642699059	-0.169465282
H	-0.125931996	11.375571660	0.654014533
H	0.475888455	10.836130806	-0.919471066
P	-1.786971979	0.702569996	-0.549125146
P	-1.297697251	0.313415471	1.598161854
Si	-3.504589708	-0.802578649	-1.057582508
C	-3.309869546	-1.150323398	-2.919257778
H	-3.244730182	-0.211403108	-3.507676441
H	-4.185245728	-1.732721506	-3.279347872
H	-2.395148533	-1.747113592	-3.115176944
C	-5.048737731	0.283330718	-0.790765296
H	-5.171030890	0.543374900	0.281983780

H	-5.960684230	-0.264019226	-1.112343923
H	-4.981202554	1.230452174	-1.365471929
C	-3.653152643	-2.413104777	-0.059141154
H	-2.749707175	-3.032562406	-0.259468382
H	-3.608149420	-2.166191196	1.026874246
C	-4.949105927	-3.210327765	-0.378360314
H	-5.003893103	-3.418975597	-1.471202749
H	-5.832820084	-2.566562861	-0.159209905
C	-5.107525672	-4.549308208	0.367076805
H	-5.070733931	-4.346900942	1.464610751
H	-4.202233243	-5.173433079	0.158892848
B	-6.441773079	-5.422170443	-0.092419472
C	-7.905088299	-4.711502950	0.267182485
H	-7.975744362	-3.695451770	-0.189156277
C	-8.013518093	-4.532915921	1.802437867
H	-7.209067866	-3.843901372	2.147639205
H	-8.979012514	-4.041013319	2.092722741
C	-7.895184006	-5.862557094	2.576119765
H	-7.895150456	-5.668278293	3.675523659
H	-8.813427291	-6.468687404	2.388252565
C	-6.660149395	-6.700882761	2.185084057
H	-6.711764497	-7.680522408	2.730000662
H	-5.744849606	-6.192775921	2.565483424
C	-6.528864277	-6.906846272	0.655515438
H	-5.586736577	-7.478265942	0.475739821
C	-7.728488515	-7.748651121	0.104803527
H	-7.350210022	-8.623469275	-0.474191664
H	-8.308355494	-8.198930688	0.951342518
C	-8.696310946	-6.964412661	-0.812390251
H	-9.612269562	-7.573955529	-1.010937571
H	-8.186279436	-6.815093711	-1.785709452
C	-9.096848275	-5.568953575	-0.278406975
H	-9.881723442	-5.689499782	0.512021370
H	-9.611952002	-5.027379561	-1.105903423
P	0.044120424	-0.014992443	-1.594050861
P	0.288908757	-1.941406729	-0.507255628
P	0.312791350	-1.289273495	1.631312962
Si	2.446688418	-2.730653937	-0.930860030
C	2.618206454	-2.601581356	-2.823095069
H	1.759381413	-3.073288151	-3.344677550

H	3.551222553	-3.111108730	-3.146755845
H	2.677885307	-1.540580697	-3.143012773
C	2.278786065	-4.567369329	-0.447561985
H	2.124140306	-4.677730149	0.646593891
H	3.206752781	-5.116876033	-0.715307049
H	1.420841332	-5.045824700	-0.964024071
C	3.934719364	-1.950475472	-0.043140323
H	3.998148967	-0.878489649	-0.337354650
H	3.732112874	-1.944156196	1.052920456
C	5.273253387	-2.683106673	-0.341406968
H	5.449782277	-2.711479972	-1.440740497
H	5.179638307	-3.750652302	-0.032738760
C	6.525201153	-2.077907281	0.321868349
H	6.366621125	-2.064117519	1.427046575
H	6.588695226	-1.000250190	0.026872008
B	7.944978103	-2.809757098	-0.126386497
C	8.105706855	-4.394557645	0.361052292
H	7.257670362	-5.014176609	-0.016636929
C	8.061977288	-4.452719977	1.908793741
H	7.068180456	-4.092215473	2.260357997
H	8.152079557	-5.506126477	2.283857346
C	9.163748133	-3.604157587	2.577578188
H	9.034998938	-3.613016120	3.686535081
H	10.150877808	-4.092404966	2.395763485
C	9.227855523	-2.151266890	2.062020708
H	10.110069722	-1.646685413	2.537790102
H	8.332171857	-1.599453105	2.427843321
C	9.285396246	-2.060347655	0.516793140
H	9.281078742	-0.977273875	0.246004214
C	10.607420611	-2.699233990	-0.026731085
H	11.142201371	-1.975014432	-0.684883934
H	11.319511627	-2.895227098	0.815945862
C	10.405237542	-4.003360300	-0.833616503
H	11.393051866	-4.489793426	-1.027330005
H	9.980047221	-3.723194316	-1.818678475
C	9.436639549	-5.017630216	-0.181136952
H	9.972055903	-5.562097237	0.638764274
H	9.211187603	-5.803093076	-0.939818757
F	-6.316633719	-5.603898588	-1.556360106
F	7.986164498	-2.731570260	-1.604480612

F	-1.582535171	8.256278791	-1.611883992

9.26. $[(\{C_8H_{14}\}BCH_2CH_2CH_2SiMe_2)_2(\{C_8H_{14}\}BHCH_2CH_2CH_2SiMe_2)P_7]^-$

Charge = –1 Multiplicity = 1

P	-1.729186972	1.040646746	0.710702238
P	-1.109139871	0.805358829	-1.430891529
Si	-1.390155608	3.303499802	1.091674329
C	-0.909233990	3.501328208	2.919185831
H	-1.628505433	2.978547405	3.583253128
H	-0.900489643	4.576093530	3.202853825
H	0.100348789	3.082929491	3.110167789
C	-3.108113978	4.074962077	0.805004905
H	-3.437969631	3.938410412	-0.246463745
H	-3.093294174	5.165631848	1.019472162
H	-3.869294440	3.606069055	1.462851375
C	-0.112176026	4.123547825	-0.060651806
H	0.845728079	3.569305021	0.062018340
H	-0.432391515	3.934898443	-1.111305513
C	0.109303571	5.634580709	0.163235862
H	0.435824612	5.812617208	1.212659398
H	-0.857897827	6.178687583	0.061095345
C	1.140161798	6.259983125	-0.796559532
H	0.864678244	6.048090553	-1.858144917
H	2.118719108	5.719667013	-0.693180227
B	1.544207720	7.776745618	-0.644943096
C	1.114367725	8.748165872	0.537262718
H	0.639946282	8.219920504	1.390976774
C	-0.003777948	9.612918227	-0.146927876
H	-0.855829967	8.952593287	-0.431346295
H	-0.418320310	10.336521918	0.592707004
C	0.491748955	10.362884894	-1.397496635
H	-0.357546370	10.898024965	-1.876594375
H	1.207333901	11.158329899	-1.091004655
C	1.169013695	9.433594451	-2.421818580
H	1.575624407	10.032248916	-3.269553447
H	0.400452581	8.761162941	-2.869062654
C	2.312974953	8.563150900	-1.794502362
H	2.690951932	7.891107859	-2.595247390
C	3.484331173	9.433065009	-1.232237734
H	4.440208946	9.120982462	-1.705284237

H	3.349192215	10.502359030	-1.517194454
C	3.626231121	9.330213907	0.299027465
H	4.431657484	10.007579784	0.659409379
H	3.964191097	8.297419147	0.555673868
C	2.310846131	9.615194060	1.050099709
H	2.069454854	10.700585388	0.969957444
H	2.466781808	9.427847639	2.134387379
P	1.643213471	0.848217980	0.719754561
P	1.128579817	0.420683847	-1.414777918
Si	3.485125891	-0.552052163	1.145750952
C	3.420456071	-0.837435692	3.023411301
H	3.346282651	0.115046177	3.588191029
H	4.348265817	-1.360332083	3.340314193
H	2.558567046	-1.478488393	3.301519886
C	4.938140025	0.601292577	0.725742350
H	4.950078798	0.854338126	-0.355188124
H	5.894750439	0.086572823	0.959064262
H	4.896804095	1.547072749	1.304889685
C	3.572430284	-2.162854972	0.161629951
H	2.661338157	-2.764158667	0.380124380
H	3.520355279	-1.921796378	-0.925523870
C	4.853341175	-2.989551884	0.469076479
H	4.907233290	-3.201261832	1.562404215
H	5.753092176	-2.369610572	0.246498750
C	4.961341743	-4.324434897	-0.287482069
H	4.898929012	-4.102370951	-1.382709639
H	4.041246078	-4.922131646	-0.063876958
B	6.298988622	-5.213172158	0.129982436
C	7.752043782	-4.556192190	-0.359752459
H	7.881580446	-3.514039199	0.021391283
C	7.792046407	-4.472683845	-1.906830684
H	6.986622865	-3.784348061	-2.255485216
H	8.752256835	-4.022182907	-2.271751490
C	7.604210974	-5.840589782	-2.594095461
H	7.554964522	-5.711226477	-3.701767342
H	8.515414191	-6.459283978	-2.414993040
C	6.370611968	-6.619113605	-2.093354303
H	6.367589837	-7.628587841	-2.582701328
H	5.449704540	-6.106323282	-2.457160032
C	6.309605346	-6.733560091	-0.549080701

H	5.368778681	-7.281966107	-0.301488385
C	7.516774858	-7.570827774	-0.005701033
H	7.148584600	-8.405394378	0.636282602
H	8.053951997	-8.077113927	-0.847789806
C	8.534339686	-6.755011773	0.823728900
H	9.440639282	-7.373343482	1.033931920
H	8.069974056	-6.533868336	1.808128697
C	8.948282927	-5.410170766	0.183814758
H	9.691518829	-5.606152143	-0.630545416
H	9.516341263	-4.832035869	0.950193415
P	-0.094591685	-0.008509048	1.807858558
P	-0.207682339	-1.956569133	0.732074972
P	-0.315614878	-1.324917049	-1.414018226
Si	-2.329702294	-2.807704113	1.127020490
C	-2.719758013	-2.497791235	2.961157344
H	-1.897233663	-2.862090013	3.611022345
H	-3.648814803	-3.033040507	3.254615594
H	-2.862876535	-1.415987136	3.161865443
C	-2.127182927	-4.672846256	0.821629151
H	-1.863846410	-4.880297702	-0.236786309
H	-3.068116947	-5.217212940	1.053990072
H	-1.320060990	-5.093356396	1.456531146
C	-3.698347502	-2.105046432	-0.001800599
H	-3.727011219	-1.002706308	0.152970180
H	-3.371273120	-2.247958120	-1.057576385
C	-5.102714091	-2.711981780	0.201645527
H	-5.424419181	-2.567728744	1.257937492
H	-5.060978575	-3.815818623	0.056963765
C	-6.175586710	-2.123766268	-0.735275674
H	-5.847276836	-2.200711587	-1.800582561
H	-6.232607093	-1.012882731	-0.585504479
B	-7.675919201	-2.595342007	-0.615456451
C	-8.269823294	-3.563014485	0.495948407
H	-7.571635775	-3.746031156	1.339657403
C	-8.399416051	-4.913745604	-0.295423358
H	-7.385134027	-5.255693159	-0.606472720
H	-8.787788071	-5.706445890	0.384975607
C	-9.297605000	-4.801143184	-1.541709680
H	-9.293144882	-5.765036606	-2.096470729
H	-10.353262334	-4.647851701	-1.223833209

C	-8.875237310	-3.656089191	-2.480795040
H	-9.599098710	-3.566961364	-3.323706584
H	-7.896463980	-3.910465213	-2.949647284
C	-8.750389422	-2.276521515	-1.745043795
H	-8.382796604	-1.538960795	-2.491293197
C	-10.111791030	-1.794285336	-1.145880605
H	-10.356938423	-0.785250316	-1.542242752
H	-10.943980357	-2.454137689	-1.484717375
C	-10.103851254	-1.738886262	0.394389001
H	-11.107456757	-1.448125525	0.776432292
H	-9.410762570	-0.924720055	0.716748972
C	-9.644045809	-3.058043973	1.045335658
H	-10.431875002	-3.833140828	0.900082203
H	-9.573748580	-2.912631738	2.144921435
H	6.269057245	-5.302409429	1.390937689

9.27. $[(\{C_8H_{14}\}BCH_2CH_2CH_2SiMe_2)(\{C_8H_{14}\}BHCH_2CH_2CH_2SiMe_2)_2P_7]^{2-}$

Charge = –2 Multiplicity = 1

P	-1.957587382	0.834826890	0.596031005
P	-1.667329459	0.752540981	-1.625813302
Si	-0.722249770	2.700340420	1.193507526
C	-0.079115679	2.405314589	2.958685901
H	-0.901931656	2.095370040	3.635957970
H	0.372227504	3.334254907	3.370226625
H	0.692473564	1.608069857	2.969675437
C	-2.003645334	4.109444659	1.219038270
H	-2.425977146	4.283407261	0.206927764
H	-1.544306343	5.060085450	1.567750974
H	-2.847839482	3.864513879	1.896644589
C	0.704284758	3.148747658	0.008166820
H	1.376678379	2.262781719	-0.044527226
H	0.274225687	3.255470326	-1.014427722
C	1.509280471	4.411814369	0.378464216
H	1.930270956	4.300300493	1.403338826
H	0.828487680	5.292532918	0.435560562
C	2.656466043	4.725727663	-0.601962579
H	2.272441532	4.777572095	-1.649975811
H	3.352810983	3.846499436	-0.651659774
B	3.620623218	5.941361799	-0.332320272
C	3.641996264	6.853638241	0.970631849

H	3.026343202	6.446661671	1.800049579
C	2.930371844	8.157990255	0.465207564
H	1.880401117	7.915649989	0.179788479
H	2.852991768	8.889271991	1.303331898
C	3.642233621	8.808105355	-0.736099883
H	3.056854922	9.684985183	-1.091444475
H	4.621734639	9.220088153	-0.404344619
C	3.870532106	7.824846295	-1.899340286
H	4.454223301	8.322050549	-2.708897391
H	2.886863780	7.562882495	-2.353813112
C	4.597890029	6.509945026	-1.453910285
H	4.656121588	5.847778496	-2.344774262
C	6.034327870	6.781238432	-0.898449664
H	6.773922715	6.180817098	-1.470527101
H	6.323818782	7.845239781	-1.065482854
C	6.171832356	6.446252829	0.599760122
H	7.189771471	6.709564813	0.963337617
H	6.084831118	5.340564537	0.725046424
C	5.098102237	7.123459105	1.473970124
H	5.298756465	8.219503724	1.520781175
H	5.202778720	6.759749338	2.518959573
P	1.053366521	-0.604126771	0.088400706
P	0.225593363	-0.454295882	-1.984169849
Si	2.265190682	-2.607608934	0.080515987
C	1.574353540	-3.895368109	-1.126673493
H	0.522707682	-4.151010108	-0.883164718
H	2.194207856	-4.816403158	-1.071197979
H	1.611462243	-3.522378461	-2.171379107
C	2.155978710	-3.251733361	1.865758359
H	2.509410708	-2.491020311	2.592479825
H	2.802835661	-4.149069652	1.971987453
H	1.116630899	-3.534150835	2.132507292
C	4.037961634	-2.097230204	-0.370434285
H	4.025455032	-1.646791365	-1.390789805
H	4.342958497	-1.269290074	0.311146151
C	5.082564985	-3.245453837	-0.313628913
H	4.766624608	-4.072745523	-0.990729434
H	5.077090926	-3.689965864	0.709342025
C	6.521702022	-2.836935451	-0.678909809
H	6.811132449	-1.984352040	-0.012986093

H	6.501320601	-2.395837210	-1.708564448
B	7.612187845	-4.086509122	-0.648286158
C	7.963996310	-4.668044385	0.876661061
H	7.039126773	-4.982795303	1.417536942
C	8.616733854	-3.547730470	1.725555223
H	7.886370559	-2.714589195	1.851402130
H	8.854425563	-3.900206837	2.763996829
C	9.906922598	-2.982516928	1.097265086
H	10.286305431	-2.124362952	1.702734624
H	10.706682832	-3.758392271	1.161908130
C	9.742803407	-2.562388409	-0.377645238
H	10.746849185	-2.247652398	-0.768720290
H	9.103056371	-1.650325449	-0.422400864
C	9.107159876	-3.668585023	-1.256877005
H	9.007780389	-3.246943677	-2.286294212
C	10.042609995	-4.922705575	-1.333897208
H	10.238321596	-5.192782345	-2.398608141
H	11.053105067	-4.686768555	-0.911484903
C	9.481593685	-6.178322768	-0.628069375
H	10.264554818	-6.974447872	-0.581305457
H	8.660267890	-6.581016653	-1.257745432
C	8.907167813	-5.916119357	0.782999969
H	9.753524168	-5.824353354	1.511119843
H	8.361047525	-6.835540908	1.100705590
P	-0.784414057	-0.925969411	1.303568732
P	-1.785174470	-2.460370735	0.039520613
P	-1.797159451	-1.485825621	-1.973242170
Si	-4.043564621	-2.500356675	0.674261511
C	-4.008670599	-2.437051577	2.574859778
H	-3.319983499	-3.195787551	3.001348716
H	-5.030648352	-2.630580148	2.965907948
H	-3.689632720	-1.436414748	2.932698626
C	-4.563973911	-4.245742176	0.116217143
H	-4.517135564	-4.341446379	-0.989064484
H	-5.612570428	-4.442015864	0.427013106
H	-3.911978115	-5.027781423	0.558054293
C	-5.190404418	-1.188500710	-0.070642943
H	-4.794306585	-0.180442380	0.186625751
H	-5.130192614	-1.260126460	-1.181618750
C	-6.666459781	-1.328528916	0.398088113

H	-6.711382643	-1.246722067	1.509061201
H	-7.029359749	-2.357802786	0.167715906
C	-7.648392935	-0.305758284	-0.202825422
H	-7.592178700	-0.394799779	-1.317409135
H	-7.255832053	0.719104515	0.019373498
B	-9.193966641	-0.430068520	0.388168415
C	-10.005714540	-1.818325927	-0.060652969
H	-9.436424226	-2.735375341	0.225888550
C	-10.163029146	-1.855392507	-1.601842346
H	-9.151485138	-1.897180793	-2.069042064
H	-10.691827240	-2.784805131	-1.942116567
C	-10.917057724	-0.633590635	-2.165271021
H	-10.926029607	-0.667758055	-3.281282941
H	-11.989712496	-0.704601931	-1.864702682
C	-10.354229670	0.717396939	-1.678541603
H	-11.015849507	1.536213736	-2.068097033
H	-9.359016549	0.883506191	-2.152465423
C	-10.198426606	0.791827069	-0.138691480
H	-9.756943711	1.790850833	0.094150748
C	-11.594852816	0.702076988	0.565642054
H	-11.733277012	1.565209199	1.259041218
H	-12.421128535	0.805769614	-0.183773199
C	-11.813468161	-0.592916130	1.380863356
H	-12.878029591	-0.661801503	1.714270397
H	-11.198676966	-0.519453879	2.302805913
C	-11.403538571	-1.888744374	0.644302659
H	-12.202485037	-2.158307487	-0.093233543
H	-11.417166579	-2.718815406	1.389679819
H	7.105854490	-5.006341510	-1.352237687
H	-9.087064952	-0.384469688	1.647541151

9.28. $[(\{C_8H_{14}\}BHCH_2CH_2CH_2SiMe_2)_3P_7]^{3-}$

Charge = –3 Multiplicity = 1

P	1.758905143	0.822649151	-0.533184883
P	1.108896905	0.696708913	1.602190110
Si	1.862405233	3.108885677	-1.010672093
C	1.610978220	3.201040567	-2.896394860
H	2.276351749	2.492841501	-3.432930525
H	1.832669541	4.230689835	-3.250967366
H	0.561290016	2.962396297	-3.166186371

C	3.686562064	3.493688482	-0.610705434
H	3.884410155	3.388900401	0.477013431
H	3.928479735	4.539173876	-0.899137981
H	4.372976088	2.808150495	-1.149866649
C	0.719522454	4.316530592	-0.091549029
H	-0.337325556	4.036047033	-0.301663341
H	0.855301493	4.163238294	1.004152820
C	0.978103324	5.805142580	-0.462782451
H	0.846343978	5.940975024	-1.561691054
H	2.049074561	6.048964860	-0.268866791
C	0.093958291	6.840510359	0.260263112
H	0.224911535	6.687767420	1.361798010
H	-0.975841482	6.573490376	0.065787787
B	0.350420774	8.408932139	-0.217924579
C	1.822206701	9.054971781	0.239418883
H	2.671269496	8.425996836	-0.120533769
C	1.918380930	9.091235239	1.785411806
H	1.875932131	8.047765598	2.174749167
H	2.903578658	9.503977830	2.130880352
C	0.790567923	9.910488882	2.445703805
H	0.857284585	9.830891681	3.557713844
H	0.954711710	10.992193326	2.222333876
C	-0.621241242	9.513816293	1.967464718
H	-1.362214541	10.216858114	2.434425942
H	-0.866604428	8.504670993	2.371558907
C	-0.754078273	9.482949755	0.424326966
H	-1.796657114	9.155188638	0.195613083
C	-0.552360457	10.914371832	-0.178858191
H	-1.417822042	11.184656362	-0.829762389
H	-0.555411402	11.688366691	0.631862253
C	0.733657027	11.072890052	-1.021845824
H	0.893897671	12.148991144	-1.280844220
H	0.575174537	10.536743592	-1.981417588
C	2.005339236	10.489771755	-0.363810518
H	2.370596768	11.202831113	0.419980112
H	2.811166265	10.483371568	-1.135882267
P	-1.595958778	1.102486505	-0.533562959
P	-1.161308239	0.603846593	1.602078787
Si	-3.626692839	0.046076875	-1.010381342
C	-3.576725706	-0.229774355	-2.894317067

H	-3.294136323	0.696901439	-3.436169540
H	-4.578800187	-0.553806926	-3.249045934
H	-2.845498259	-1.021952945	-3.157559755
C	-4.872938313	1.436228372	-0.622914246
H	-4.883287120	1.667836940	0.463150352
H	-5.898742605	1.120915933	-0.911225300
H	-4.621310393	2.369532359	-1.168302930
C	-4.105047677	-1.541030164	-0.081851385
H	-3.329521713	-2.315807972	-0.277290454
H	-4.051461448	-1.336248480	1.012573480
C	-5.518441067	-2.068671344	-0.461884368
H	-5.555793625	-2.270898012	-1.557775608
H	-6.267829924	-1.259923208	-0.292567204
C	-5.981571650	-3.338777594	0.278961013
H	-5.929471663	-3.127938434	1.377406955
H	-5.212215739	-4.134447104	0.109867316
B	-7.461521715	-3.911116815	-0.207812455
C	-8.762102707	-2.946860801	0.206043625
H	-8.634294971	-1.906182336	-0.177105976
C	-8.865990097	-2.844396872	1.748547467
H	-7.947273674	-2.350235429	2.140994914
H	-9.721028980	-2.189087431	2.064516625
C	-9.023721165	-4.214104880	2.439817158
H	-9.005902497	-4.089568539	3.549567567
H	-10.039280941	-4.617476958	2.209824094
C	-7.968099445	-5.250485054	2.003436632
H	-8.214726417	-6.231715800	2.491018390
H	-6.977443650	-4.949286905	2.415560704
C	-7.851569587	-5.388079151	0.464820552
H	-7.044298369	-6.133493305	0.266473559
C	-9.183967520	-5.941557701	-0.145196914
H	-8.977130654	-6.842227433	-0.771094925
H	-9.865706781	-6.310344924	0.664505334
C	-9.949325042	-4.926995060	-1.025293864
H	-10.957978366	-5.331118353	-1.289861390
H	-9.390989809	-4.820741290	-1.979380462
C	-10.087796757	-3.518180869	-0.403892647
H	-10.900105637	-3.537773826	0.368129902
H	-10.471796504	-2.835715661	-1.199055415
P	0.000839069	-0.005926536	-1.618441255

P	-0.160809707	-1.942474069	-0.532914928
P	0.054184196	-1.315928068	1.602532647
Si	1.769432346	-3.173165420	-1.009576103
C	1.975372452	-3.002997728	-2.895514785
H	1.029594551	-3.226505021	-3.431941350
H	2.756818973	-3.709452266	-3.249501360
H	2.292796843	-1.974684045	-3.166374139
C	1.193058789	-4.946191510	-0.609381739
H	1.002404180	-5.065248220	0.478165253
H	1.979049080	-5.677208423	-0.896860196
H	0.257176246	-5.199627083	-1.149461701
C	3.385495098	-2.784670620	-0.089338643
H	3.665552293	-1.726479299	-0.293454255
H	3.185071352	-2.832865999	1.006160730
C	4.550134751	-3.744934272	-0.465695297
H	4.738092863	-3.686940362	-1.563236166
H	4.228480546	-4.797382004	-0.283761168
C	5.884583515	-3.498565766	0.265679621
H	5.681523070	-3.545058617	1.365945050
H	6.185581968	-2.436047632	0.081263255
B	7.121020815	-4.496150697	-0.213987624
C	6.947509734	-6.098272220	0.228169321
H	5.981928263	-6.519316808	-0.141381660
C	6.921524168	-6.213230845	1.773022174
H	6.033810335	-5.662539842	2.161702376
H	6.789078087	-7.276487482	2.108146903
C	8.188142722	-5.646655528	2.446697451
H	8.078443908	-5.674462099	3.557803973
H	9.047241930	-6.324154916	2.223360225
C	8.547896149	-4.220253475	1.982994234
H	9.522782726	-3.930178648	2.459217372
H	7.790985830	-3.509561344	2.387897154
C	8.597653204	-4.076824681	0.441349660
H	8.832816345	-3.007369076	0.223066441
C	9.743651414	-4.958429300	-0.161244265
H	10.413071270	-4.336355265	-0.802052281
H	10.410948052	-5.347441778	0.651011685
C	9.248060992	-6.146125243	-1.017891611
H	10.104036519	-6.818009425	-1.275890200
H	8.869114094	-5.734321360	-1.977141158

C	8.105254356	-6.965087679	-0.375143636
H	8.536576042	-7.644070774	0.405338631
H	7.704044284	-7.653709579	-1.156365157
H	-7.397891538	-4.021452859	-1.466114197
H	7.175347051	-4.406545907	-1.474297140
H	0.259332356	8.410319536	-1.479274978

9.29. $ClSiMe_2CH_2CH_2BCy_2$ (**4**)

Charge = 0 Multiplicity = 1

Si	3.484465499	-0.123827040	0.312657177
C	3.991551387	1.640891044	0.750188926
C	3.868349879	-1.318611595	1.722818498
C	1.666940424	-0.230479767	-0.225121623
H	3.294833673	-1.052038387	2.636057365
H	4.948080387	-1.291130203	1.977369234
H	3.609451429	-2.360915126	1.443810450
H	1.483908195	-1.271052356	-0.575921761
H	1.547430669	0.412351243	-1.126006640
C	0.641606776	0.170309219	0.883523283
H	3.422558873	2.009336974	1.630274425
H	3.802887193	2.331710003	-0.097419160
H	5.072412915	1.689798986	0.996655108
H	0.848773002	1.206714610	1.229202278
H	0.783904036	-0.498481134	1.764505198
B	-0.827909697	-0.021938625	0.303077344
C	-1.384453707	-1.505652125	0.126419334
C	-1.931827726	-1.831196846	-1.288307350
C	-2.520591027	-3.251739912	-1.374226944
C	-3.606110775	-3.482313341	-0.308841478
C	-3.082735013	-3.170354129	1.103651282
C	-2.497375068	-1.747754315	1.191447085
H	-0.571247491	-2.240087047	0.344865480
H	-1.128532196	-1.709661776	-2.048011639
H	-2.723320672	-1.094105930	-1.557609515
H	-2.930802811	-3.436518204	-2.391291752
H	-1.702561514	-3.995136091	-1.229834100
H	-3.986201987	-4.525953785	-0.359553356
H	-4.478419068	-2.823326483	-0.527859974
H	-3.893499917	-3.295878809	1.854703344
H	-2.290642340	-3.906634843	1.372987828

H	-2.102939428	-1.565367174	2.215961438
H	-3.319366314	-1.009900981	1.039903828
C	-1.723519965	1.242304987	-0.075361409
C	-1.045369679	2.155119634	-1.135971799
C	-1.899041359	3.392513316	-1.471125849
C	-2.248193138	4.200403687	-0.209530534
C	-2.922237644	3.317097964	0.854144442
C	-2.068304042	2.080114754	1.191144369
H	-2.694024314	0.904153713	-0.510970958
H	-0.836204190	1.579970820	-2.064764058
H	-0.054842908	2.493498231	-0.752043841
H	-1.369337224	4.034683042	-2.208677499
H	-2.840853977	3.061292301	-1.966683986
H	-2.900105715	5.063126627	-0.468264049
H	-1.313494012	4.634623005	0.215973559
H	-3.122868048	3.905412147	1.776508388
H	-3.915829170	2.981890657	0.476435687
H	-2.597077036	1.450431357	1.940234554
H	-1.124677161	2.414263283	1.681535518
Cl	4.647500158	-0.718688968	-1.353750589

9.30. $[ClSiMe_2CH_2CH_2FBCy_2]^-$

Charge = –1 Multiplicity = 1

Si	3.528266987	0.370479940	0.220273926
C	3.866950306	2.151322030	-0.336571486
C	3.687432629	0.250575002	2.106848709
C	1.902865639	-0.305797625	-0.424375565
H	2.890186822	0.850080869	2.595398333
H	4.673398934	0.622654119	2.455850117
H	3.575594468	-0.801515447	2.443038693
H	1.849257824	-1.392471557	-0.184426737
H	1.896860974	-0.250915461	-1.536578540
C	0.665459335	0.448434702	0.131790647
H	3.078853762	2.830832778	0.051553533
H	3.858768792	2.220963322	-1.444333589
H	4.852773087	2.509179960	0.027275110
H	0.810458124	1.546405361	-0.024052783
H	0.612159962	0.314678099	1.239066967
B	-0.766781131	0.004511686	-0.598153408
C	-1.159402809	-1.587494101	-0.274906108

C	-2.299434043	-2.112532009	-1.175998141
C	-2.566122993	-3.621093408	-1.004822700
C	-2.835854093	-3.984707527	0.467065284
C	-1.708593632	-3.478283010	1.385680763
C	-1.458031300	-1.967921012	1.190976747
H	-0.249302857	-2.174476112	-0.569852919
H	-2.064769921	-1.875133089	-2.235529069
H	-3.241140518	-1.558729936	-0.939879523
H	-3.418166411	-3.955859656	-1.642843480
H	-1.672945522	-4.190577183	-1.356848678
H	-2.977057083	-5.084396615	0.585198498
H	-3.796493308	-3.512694311	0.784273677
H	-1.947161389	-3.712975994	2.450036861
H	-0.771560194	-4.035414772	1.146524678
H	-0.631791761	-1.635382039	1.859369475
H	-2.366579603	-1.419581546	1.538591535
C	-1.983793604	1.105013554	-0.251588954
C	-1.886733231	2.366560775	-1.140751634
C	-3.039156573	3.365171209	-0.918575058
C	-3.157413168	3.774221286	0.560857239
C	-3.262229142	2.539116639	1.473148243
C	-2.103999915	1.549665850	1.224742377
H	-2.959451605	0.617567064	-0.515666421
H	-1.829752536	2.062203856	-2.206682120
H	-0.921276439	2.888498546	-0.928202343
H	-2.915703220	4.271752105	-1.557343841
H	-3.998302884	2.890526771	-1.236807751
H	-4.025205562	4.456768013	0.717465806
H	-2.248964027	4.356352820	0.847235938
H	-3.297277625	2.854324935	2.542891620
H	-4.230803888	2.022853373	1.269765111
H	-2.218724712	0.671218948	1.896567636
H	-1.151615627	2.041973976	1.536921896
Cl	5.182160318	-0.772365887	-0.545777333
F	-0.532720451	0.059857466	-2.055994797

9.31. $[ClSiMe_2CH_2CH_2HBCy_2]^-$

Charge = –1 Multiplicity = 1

Si	-3.511478103	0.357646674	-0.160883516
C	-3.851598218	2.190437325	0.189378148

C	-3.638990664	0.031641345	-2.025059471
C	-1.900095591	-0.247332268	0.587797515
H	-2.830729139	0.571812525	-2.562153910
H	-4.617484674	0.365073453	-2.429808786
H	-3.525057231	-1.051233402	-2.241077286
H	-1.834268275	-1.350315451	0.441844619
H	-1.934894324	-0.105022976	1.692563033
C	-0.648434878	0.463080953	0.003838428
H	-3.053063738	2.818979345	-0.258689814
H	-3.861878767	2.383572289	1.282464143
H	-4.829290794	2.509148818	-0.228812520
H	-0.776197935	1.566948001	0.131865586
H	-0.628864310	0.304711586	-1.104695486
B	0.792442428	0.002708193	0.705762956
C	1.164331778	-1.591991113	0.371380722
C	2.239259139	-2.171663566	1.318173304
C	2.526518204	-3.670016263	1.090276586
C	2.892901936	-3.962344930	-0.376196676
C	1.822300027	-3.416016628	-1.338010148
C	1.557662104	-1.916241319	-1.088662834
H	0.231767061	-2.182298288	0.578250665
H	1.934821758	-1.994766476	2.373541967
H	3.192532011	-1.603818590	1.182144429
H	3.336393672	-4.033423018	1.766601740
H	1.615299455	-4.258409859	1.354650100
H	3.048941780	-5.054863794	-0.535938616
H	3.868387398	-3.472017435	-0.609095640
H	2.125978530	-3.602593467	-2.395496182
H	0.874397675	-3.984530300	-1.181863378
H	0.774971916	-1.551331409	-1.791986299
H	2.485983925	-1.353407989	-1.351039916
C	2.022592240	1.088023810	0.353505309
C	2.020991315	2.297880495	1.317581620
C	3.160359547	3.306889580	1.066306402
C	3.176407535	3.795368600	-0.392935254
C	3.214091226	2.609259263	-1.372282916
C	2.063552067	1.616818935	-1.101841464
H	2.999439355	0.561233283	0.523455991
H	2.057099274	1.932685274	2.367191316
H	1.044232461	2.834221814	1.225125761

H	3.088884058	4.176804123	1.761677767
H	4.137111749	2.813243524	1.287772701
H	4.033568404	4.485942989	-0.571898840
H	2.252068451	4.391201730	-0.584901090
H	3.185867637	2.979462051	-2.424671687
H	4.189402701	2.078401463	-1.256393797
H	2.124762989	0.772994116	-1.823325710
H	1.099787822	2.132939561	-1.329187038
Cl	-5.185338668	-0.688094590	0.697541717
H	0.608181169	0.052904863	1.957735784

9.32. ($\{Cy_2B\}CH_2CH_2CH_2SiMe_2)_3P_7$ (**5**)

Charge = 0 Multiplicity = 1

P	0.545949093	-0.838415162	0.442105552
P	1.413443137	0.891787661	-0.662792816
P	0.106682915	-2.139586853	-1.310341904
P	-1.501644581	-0.016778816	0.747358373
P	-1.112413134	-0.797278944	-2.625960801
P	-2.011218580	0.792732740	-1.279474852
P	-0.042916846	1.183446533	-2.339667675
Si	3.307921253	0.093581147	-1.753844033
Si	-1.451940332	-3.718382751	-0.606865015
Si	-1.245519067	1.913461799	2.026709001
C	0.086361121	1.548180989	3.327014386
C	-2.936312430	2.111570616	2.868590850
C	-0.824944415	3.472686183	1.012573358
C	-0.867575013	-4.340448488	1.087286703
C	-1.289100322	-5.099694119	-1.900231029
C	-3.249816723	-3.086530278	-0.543547809
C	4.238497358	-0.977493267	-0.478405572
C	4.283643829	1.672827446	-2.156977432
C	2.972713189	-0.858788089	-3.356499894
H	4.358977063	-0.372903889	0.448708900
C	5.618316393	-1.497946124	-0.964263042
H	3.573646862	-1.823782353	-0.196655738
H	2.389948002	-1.781883443	-3.161541883
H	3.936257190	-1.138133451	-3.835654129
H	2.403839751	-0.236617359	-4.078310563
H	4.524102029	2.247334199	-1.238650807
H	3.706757108	2.336652926	-2.834327285

H	5.237719071	1.419467249	-2.667591455
H	-1.940188441	-5.960459912	-1.636014194
H	-0.243620518	-5.466772888	-1.959691459
H	-1.584832118	-4.745813231	-2.910074309
H	-3.470801068	-2.588431386	-1.514284937
H	-3.289940456	-2.280865526	0.222717955
C	-4.302117777	-4.203122489	-0.248767852
H	-1.493025755	-5.198959748	1.414046421
H	-0.943999713	-3.544710064	1.856507833
H	0.186900886	-4.683611854	1.048365918
H	-2.927129422	2.978042353	3.564133298
H	-3.197038251	1.205547452	3.453727006
H	-3.742224229	2.280655797	2.124054839
H	0.144851052	2.384514351	4.056562045
H	1.084237568	1.427693288	2.857668796
H	-0.145286294	0.621229409	3.891280992
H	0.140488044	3.283951741	0.493234588
C	-0.757772486	4.778212325	1.868595400
H	-1.588262340	3.567969870	0.207716032
H	6.255902481	-0.649961602	-1.298806576
H	5.466764844	-2.120061403	-1.885749856
H	-4.044096518	-4.727703807	0.697342741
H	-4.260936135	-4.963315103	-1.063199322
H	0.027557096	4.656039581	2.650689370
H	-1.722301296	4.932360086	2.399514082
B	-0.367600274	5.983826316	0.905248153
B	6.402234225	-2.492790954	0.001531806
B	-5.742210599	-3.526833197	-0.223888791
C	-1.457828985	7.067428866	0.478094131
C	-1.986363797	7.863241808	1.706523813
C	-3.050062049	8.904367089	1.309963177
C	-4.217278663	8.260236137	0.542260881
C	-3.717681242	7.466232134	-0.676868413
C	-2.654321277	6.424954821	-0.280644933
H	-0.998768632	7.812718664	-0.214610750
H	-1.145497820	8.366023802	2.233378096
H	-2.430454620	7.154367661	2.443270658
H	-3.425076661	9.431869968	2.214603601
H	-2.576001388	9.683702616	0.669416459
H	-4.950682944	9.034775149	0.228272132

H	-4.766819451	7.571411457	1.225501731
H	-4.568921808	6.967410397	-1.190220087
H	-3.277098589	8.171812471	-1.418719378
H	-2.296475603	5.888666805	-1.186798342
H	-3.129963802	5.652812982	0.367851167
C	1.139693619	6.090068491	0.397817594
C	1.816116109	7.277867576	1.149438571
C	3.291727212	7.454923881	0.742537277
C	3.444200799	7.617997523	-0.779430001
C	2.786019450	6.452688524	-1.537983130
C	1.311505570	6.269455133	-1.132775769
H	1.699177785	5.168578418	0.690103786
H	1.743704145	7.134830860	2.250765945
H	1.264962785	8.220239068	0.922362226
H	3.731418519	8.328299973	1.272907027
H	3.870035484	6.562588478	1.076250907
H	4.518178022	7.705165293	-1.054167753
H	2.965096333	8.574825026	-1.092802175
H	2.865956854	6.611419103	-2.635881837
H	3.343593917	5.512774954	-1.318769459
H	0.878670088	5.397680384	-1.670542454
H	0.730807979	7.159124488	-1.469917325
C	-6.375990579	-3.028147887	-1.600068115
C	-6.713192165	-1.511506376	-1.607585628
C	-7.367468450	-1.066328702	-2.928785683
C	-8.618482785	-1.900551626	-3.253644257
C	-8.304291413	-3.406481976	-3.256294440
C	-7.654083442	-3.853381244	-1.933063191
H	-5.652669892	-3.207890825	-2.432393462
H	-5.794857755	-0.912344499	-1.422420037
H	-7.406041820	-1.283224406	-0.764654246
H	-7.623375498	0.015042810	-2.884036052
H	-6.628742408	-1.176174357	-3.756076031
H	-9.049049652	-1.592387292	-4.231420173
H	-9.403858764	-1.693902407	-2.489559025
H	-9.228550227	-3.995673699	-3.445896167
H	-7.610347419	-3.634947517	-4.097993035
H	-7.415061541	-4.939277809	-1.979016039
H	-8.395540833	-3.734025432	-1.109005714
C	-6.533320426	-3.357250426	1.150373855

C	-5.720035267	-2.596072833	2.233512846
C	-6.483833410	-2.487654199	3.566447152
C	-6.913467761	-3.869270897	4.088961985
C	-7.722123095	-4.642184593	3.033104278
C	-6.958826560	-4.751889340	1.699457593
H	-7.473459795	-2.779837697	0.980285476
H	-5.447614608	-1.581027762	1.870440932
H	-4.755878653	-3.126300488	2.411828047
H	-5.860610886	-1.963787947	4.324278863
H	-7.389286957	-1.854836760	3.417326321
H	-7.499769112	-3.765643423	5.028092807
H	-6.004137462	-4.457633599	4.354066081
H	-7.981774178	-5.656355929	3.409071373
H	-8.689841014	-4.118185053	2.856491038
H	-7.582920691	-5.289641303	0.952121827
H	-6.051238523	-5.380967908	1.852512635
C	7.982682882	-2.342789249	0.168326919
C	8.387134905	-0.967199695	0.769660421
C	9.914589000	-0.815629863	0.897645241
C	10.622744208	-1.045694991	-0.448620110
C	10.238779123	-2.403577343	-1.061063505
C	8.711812770	-2.557540548	-1.190526676
H	8.366628599	-3.125303862	0.865693429
H	7.909737967	-0.825180645	1.764378783
H	7.995488389	-0.149364265	0.121552580
H	10.167137096	0.189382621	1.301606553
H	10.293348093	-1.554113638	1.641702770
H	11.725368881	-0.973048628	-0.325406370
H	10.335396146	-0.232591446	-1.155399959
H	10.721765548	-2.531291351	-2.054811699
H	10.633528311	-3.221859728	-0.415431608
H	8.468053039	-3.560310532	-1.605931362
H	8.335556475	-1.816112998	-1.933198396
C	5.616449191	-3.656785032	0.755585719
C	5.591459483	-3.359526407	2.285309112
C	4.851207966	-4.454601980	3.076903713
C	5.436754091	-5.850168367	2.801820948
C	5.462491844	-6.161805347	1.295549087
C	6.206460498	-5.070633096	0.503119262
H	4.552494807	-3.680236596	0.418354793

H	5.119508171	-2.371410076	2.480981353
H	6.637812453	-3.284186061	2.663099312
H	4.885071881	-4.227029870	4.165105104
H	3.774526113	-4.444587766	2.789774825
H	4.861155022	-6.627355011	3.350450170
H	6.476840756	-5.894684874	3.201134448
H	5.929752603	-7.154472861	1.113117438
H	4.415851854	-6.236909886	0.919345191
H	6.180361106	-5.310451865	-0.582971318
H	7.281903029	-5.082755245	0.795426056

9.33. [({Cy_2B}$CH_2CH_2CH_2SiMe_2$)$_2$({Cy_2B}$FCH_2CH_2CH_2SiMe_2$)P_7]$^-$

Charge = –1 Multiplicity = 1

P	0.405068315	-0.743411473	0.480391279
P	1.170766092	1.072333731	-0.547831234
P	0.200260255	-2.048301644	-1.315013798
P	-1.739512765	-0.152122394	0.664694314
P	-1.072748606	-0.816000367	-2.683938513
P	-2.211481514	0.651539740	-1.375406342
P	-0.218349575	1.248909188	-2.289939375
Si	3.274453110	0.487946281	-1.435253807
Si	-1.199843831	-3.796056562	-0.725982993
Si	-1.733229659	1.768288329	1.968995930
C	-0.450015621	1.528523451	3.345070173
C	-3.479780354	1.813773166	2.725871550
C	-1.415218040	3.380833067	0.996822699
C	-0.579813987	-4.454441375	0.941712138
C	-0.911815962	-5.095943035	-2.083527640
C	-3.057694970	-3.353988890	-0.646365265
C	4.217937536	-0.474052727	-0.115625164
C	4.025611870	2.205699834	-1.760464369
C	3.119332837	-0.433097744	-3.086931754
H	4.243821918	0.116081088	0.827959550
C	5.666465278	-0.800433941	-0.556420574
H	3.654178988	-1.403406095	0.126202000
H	2.613936153	-1.411149117	-2.952730620
H	4.139757408	-0.619099931	-3.485990546
H	2.552112427	0.156311016	-3.837173201
H	4.100600297	2.791263861	-0.820799052
H	3.427439734	2.788064440	-2.492331507

H	5.051927977	2.086154344	-2.168867875
H	-1.482680784	-6.024651070	-1.867308843
H	0.163648089	-5.361273466	-2.149759121
H	-1.232024042	-4.719052759	-3.077571820
H	-3.320499987	-2.825911599	-1.590126954
H	-3.171359782	-2.595002482	0.159502844
C	-4.003145983	-4.580285842	-0.421590244
H	-1.116029817	-5.389014630	1.214449689
H	-0.740532717	-3.711918851	1.750292318
H	0.505324034	-4.682722074	0.900383378
H	-3.587800957	2.676132757	3.418740484
H	-3.686275392	0.885759904	3.298641061
H	-4.257241324	1.907587916	1.938655155
H	-0.519167072	2.355117587	4.084884997
H	0.580094530	1.515181845	2.933623749
H	-0.615086582	0.573130717	3.884892410
H	-0.433875778	3.263354130	0.486833334
C	-1.439230294	4.672779272	1.877541412
H	-2.175188167	3.437341111	0.185050675
H	6.167003825	0.138413299	-0.898552923
H	5.648423933	-1.472211589	-1.446904952
H	-3.675907016	-5.154586061	0.472822357
H	-3.921518318	-5.263672241	-1.297688292
H	-0.640444772	4.592595843	2.650494527
H	-2.408901628	4.749249655	2.416967088
B	-1.145435128	5.899260405	0.912671753
B	6.589673463	-1.454543540	0.676571872
B	-5.469910327	-3.990009285	-0.300056473
C	-2.319450855	6.897716324	0.485598407
C	-2.853931286	7.682995888	1.718138894
C	-3.992961210	8.650401539	1.343967139
C	-5.143618079	7.922588084	0.627699760
C	-4.636878676	7.139997694	-0.596101894
C	-3.498028190	6.173025492	-0.221547543
H	-1.926953655	7.656608676	-0.233207406
H	-2.028309185	8.245754143	2.207192178
H	-3.227648160	6.961442495	2.481185647
H	-4.369297602	9.170394123	2.252978403
H	-3.590331541	9.445675303	0.674377342
H	-5.935186354	8.644843908	0.328799965

H	-5.624215714	7.212243141	1.340379709
H	-5.473111585	6.582107605	-1.072550186
H	-4.266315396	7.859847189	-1.362539350
H	-3.137688409	5.645444453	-1.131416371
H	-3.902838827	5.382672229	0.452186179
C	0.344780102	6.122620512	0.391444293
C	0.927881519	7.392599183	1.082155753
C	2.378838624	7.673995483	0.646306019
C	2.499107986	7.785225048	-0.883326657
C	1.931569827	6.538312790	-1.583076831
C	0.482375992	6.250299923	-1.148597222
H	0.982725565	5.263913472	0.710192567
H	0.881099556	7.289661508	2.189457294
H	0.297353429	8.276354611	0.825190414
H	2.751400881	8.602141368	1.134245850
H	3.032768303	6.847007544	1.006094159
H	3.559270863	7.947924456	-1.177145133
H	1.938275428	8.685972357	-1.227997532
H	1.984585481	6.656798380	-2.687777504
H	2.567365110	5.657835773	-1.335612293
H	0.121060390	5.321713603	-1.641617853
H	-0.176160302	7.072870819	-1.513948079
C	-6.194424044	-3.448171311	-1.617154513
C	-6.562036388	-1.941083873	-1.528530225
C	-7.292686444	-1.443471237	-2.789522297
C	-8.541557293	-2.288264032	-3.095203953
C	-8.196820541	-3.784544459	-3.192496201
C	-7.469656704	-4.283182381	-1.929076158
H	-5.509432247	-3.567349473	-2.491436419
H	-5.647645037	-1.331900463	-1.359850278
H	-7.214757897	-1.772060235	-0.640517354
H	-7.567647419	-0.371771717	-2.675550154
H	-6.595234693	-1.493394962	-3.657272822
H	-9.028515988	-1.940275089	-4.032847485
H	-9.290820101	-2.140088650	-2.282380750
H	-9.117456237	-4.384707423	-3.367738306
H	-7.541589408	-3.951941147	-4.078513920
H	-7.211017038	-5.359499716	-2.044404503
H	-8.170775336	-4.224241572	-1.063810792
C	-6.207673160	-3.929717988	1.115710274

C	-5.377330238	-3.227492656	2.224021406
C	-6.093175661	-3.242005855	3.587359787
C	-6.463525915	-4.671142464	4.019818162
C	-7.288700628	-5.388525671	2.937718539
C	-6.573203245	-5.373613615	1.573267801
H	-7.170172545	-3.370849974	1.021969382
H	-5.145553106	-2.181092844	1.929831067
H	-4.392820123	-3.738940430	2.327669433
H	-5.456914686	-2.756914490	4.359955083
H	-7.021868768	-2.628667701	3.518932957
H	-7.016873811	-4.656560741	4.984698912
H	-5.528413359	-5.248789692	4.206877176
H	-7.504801482	-6.435954616	3.245113486
H	-8.277190024	-4.883071300	2.835746448
H	-7.209074798	-5.874965454	0.810351077
H	-5.642194035	-5.982346260	1.645972341
C	8.213852905	-1.438011916	0.265688743
C	8.847869639	-0.042936880	0.473568604
C	10.363890013	-0.011765854	0.199262657
C	10.696686198	-0.536509751	-1.209308108
C	10.083213500	-1.928141959	-1.444708824
C	8.567584823	-1.936047455	-1.155093624
H	8.739310672	-2.123928519	0.981246774
H	8.626939831	0.313164982	1.501408621
H	8.350511394	0.689456788	-0.209068113
H	10.775438039	1.016711095	0.331552837
H	10.882832392	-0.651985884	0.952290560
H	11.798812121	-0.556556822	-1.376273225
H	10.281148758	0.172009807	-1.965218885
H	10.293136369	-2.268954270	-2.485926393
H	10.585737996	-2.662046154	-0.770457695
H	8.163253770	-2.956620565	-1.330756112
H	8.065700738	-1.282029440	-1.908326674
C	6.005017377	-2.939861119	1.164614639
C	6.658280235	-3.432348753	2.475881481
C	6.016759609	-4.720197226	3.028549797
C	6.010503781	-5.845527853	1.977509889
C	5.357637700	-5.381421149	0.662471844
C	6.012225459	-4.088356109	0.132903476
H	4.927270391	-2.756124354	1.418362295

H	6.615986643	-2.619916729	3.232384122
H	7.743983619	-3.627424649	2.296746939
H	6.536408069	-5.064723483	3.953643272
H	4.963598705	-4.502527965	3.327500975
H	5.499715812	-6.754493655	2.372028758
H	7.064178659	-6.148347946	1.767605039
H	5.404833749	-6.196343105	-0.097753130
H	4.273591928	-5.189953945	0.846754021
H	5.510559448	-3.773511124	-0.810043736
H	7.065665277	-4.325586217	-0.150777670
F	6.422143143	-0.563691205	1.840253191

9.34. $[(\{Cy_2B\}CH_2CH_2CH_2SiMe_2)(\{Cy_2B\}FCH_2CH_2CH_2SiMe_2)_2P_7]^{2-}$

Charge = –2 Multiplicity = 1

P	1.450402861	0.202690970	0.065748703
P	2.238346255	1.047348341	-1.836055476
P	1.763745173	-1.952099782	-0.426477605
P	-0.737859742	0.303651824	-0.356855732
P	0.670063955	-2.176862939	-2.371819839
P	-0.853916171	-0.491789728	-2.442008602
P	1.197370123	-0.203831956	-3.368898961
Si	4.493966671	0.428864440	-2.071817128
Si	0.417477914	-3.139574592	1.032168453
Si	-1.365436077	2.555733217	-0.595119206
C	-0.782142760	3.306657916	-2.235778005
C	-0.511797403	3.424750498	0.864369808
C	-3.258335958	2.599994581	-0.435199286
C	0.554866296	-2.330940214	2.744916481
C	1.215884913	-4.869962569	1.089735804
C	-1.411262239	-3.318957297	0.501120055
C	5.462498776	0.822113364	-0.494942098
C	5.007327214	1.565388759	-3.514394553
C	4.714067163	-1.374299542	-2.631360875
H	5.287259328	1.883025492	-0.207569686
C	6.984983738	0.577460911	-0.666276120
H	5.050800861	0.213936284	0.342050600
H	4.368069270	-2.079216248	-1.847936649
H	5.793471469	-1.559484183	-2.821386244
H	4.149846246	-1.586512552	-3.563459407
H	4.886192515	2.634112168	-3.240690432

H	4.404318552	1.368646018	-4.425861485
H	6.077397130	1.394197024	-3.760177773
H	0.700100357	-5.525681270	1.824396590
H	2.283803375	-4.796059791	1.383180358
H	1.171497780	-5.364298560	0.096194792
H	-1.420011386	-3.633250276	-0.566696286
H	-1.851984950	-2.297402039	0.515316173
C	-2.239387189	-4.315733810	1.377324144
H	0.045614553	-2.953232539	3.512546718
H	0.087240064	-1.324963177	2.743436425
H	1.616733991	-2.213780773	3.045494879
H	-0.850702885	4.482153678	0.906035801
H	0.592547299	3.413877953	0.757777090
H	-0.774363891	2.945730203	1.830562098
H	-1.085829450	4.374868230	-2.272731272
H	-1.251495298	2.788887877	-3.098343424
H	0.319728612	3.235956851	-2.341877710
H	-3.689309070	1.959370624	-1.238290114
C	-3.825213858	4.041724502	-0.522009749
H	-3.532194641	2.102523934	0.522325703
H	7.336202065	1.110603353	-1.584184717
H	7.169023595	-0.504934432	-0.868241867
H	-2.180286420	-4.017638423	2.447268979
H	-1.785237680	-5.330604099	1.295296014
H	-3.535568384	4.473903662	-1.510039766
H	-3.306339622	4.686838850	0.230329156
B	-5.473605677	4.331190320	-0.417777138
B	7.909289701	1.107942576	0.618164738
B	-3.715742765	-4.322391383	0.798779256
C	-6.037513253	4.468984499	1.156934388
C	-5.742179906	5.866143540	1.751320763
C	-6.332584286	6.073593933	3.159522722
C	-5.865569753	4.980545818	4.137664178
C	-6.148986874	3.575602765	3.577300075
C	-5.556395670	3.398408843	2.162829519
H	-7.156895382	4.384016757	1.114431829
H	-6.106991315	6.647321186	1.052679940
H	-4.635133053	6.009654875	1.807027035
H	-6.072453556	7.082112688	3.560742018
H	-7.447111372	6.044063038	3.094684306

H	-6.340365255	5.111214466	5.138403450
H	-4.766496828	5.087621400	4.299145436
H	-5.757520278	2.798621801	4.275569849
H	-7.253853354	3.422612246	3.529525339
H	-5.783041651	2.374329240	1.794611743
H	-4.444100468	3.451682863	2.243415800
C	-6.378022038	3.286296398	-1.368902188
C	-7.694918834	3.946409466	-1.842230583
C	-8.503067057	3.075855301	-2.823758673
C	-8.797102649	1.683278126	-2.237295637
C	-7.504356338	0.996934361	-1.761689949
C	-6.710481772	1.893630728	-0.787569393
H	-5.771425787	3.107738961	-2.297448692
H	-7.467008225	4.935983358	-2.289805748
H	-8.334027417	4.160199192	-0.949968763
H	-9.456708437	3.577191599	-3.116112752
H	-7.917383732	2.950999554	-3.765970518
H	-9.335705286	1.044736682	-2.976309716
H	-9.485269562	1.797739837	-1.365551183
H	-7.742741481	0.012161801	-1.294960547
H	-6.867087016	0.773247788	-2.650010875
H	-5.786693185	1.363296100	-0.469791026
H	-7.320229326	2.022994145	0.139056628
C	-3.989144075	-5.052777063	-0.597666909
C	-4.564242553	-4.099668188	-1.680894240
C	-4.862999597	-4.831322829	-3.002663923
C	-5.797792996	-6.034462123	-2.789334783
C	-5.241610985	-6.995104041	-1.723600136
C	-4.945362241	-6.263543483	-0.400155837
H	-3.028743672	-5.461954412	-0.995703849
H	-3.860325012	-3.260714091	-1.867850186
H	-5.503780476	-3.633642116	-1.303967154
H	-5.303025709	-4.124650921	-3.739996863
H	-3.904588059	-5.187067696	-3.447021430
H	-5.967169721	-6.573849264	-3.747968850
H	-6.797153120	-5.665524825	-2.459159332
H	-5.951286942	-7.835191975	-1.548833951
H	-4.299562322	-7.455344682	-2.102896925
H	-4.519224047	-6.979963222	0.337887852
H	-5.907668872	-5.906214217	0.035596784

C	-4.907881802	-3.619621073	1.593644427
C	-4.625833244	-2.129990678	1.934590381
C	-5.767254547	-1.493021849	2.747538459
C	-6.064715715	-2.288883164	4.029703177
C	-6.349563361	-3.768589935	3.718756414
C	-5.209161243	-4.407785647	2.903017175
H	-5.842988648	-3.644334174	0.983441129
H	-4.454988173	-1.544353916	1.006377941
H	-3.679507718	-2.057539529	2.518521901
H	-5.519767502	-0.437888445	2.991905432
H	-6.685989967	-1.454688424	2.118182892
H	-6.918480393	-1.836279636	4.580876149
H	-5.185144608	-2.224419252	4.712395360
H	-6.514437529	-4.340872661	4.659733249
H	-7.297979010	-3.843559836	3.137177758
H	-5.460088638	-5.467475900	2.671725605
H	-4.289000257	-4.439698174	3.531795684
C	9.535443291	1.158646499	0.197936681
C	9.884759755	2.439269835	-0.595104982
C	11.386224580	2.572463235	-0.915961499
C	11.919479721	1.333694755	-1.658622800
C	11.591211018	0.040222858	-0.891495212
C	10.084522420	-0.064209037	-0.572996803
H	10.121795231	1.219281967	1.153126217
H	9.520821224	3.327856598	-0.038390976
H	9.316431698	2.438298451	-1.557583844
H	11.591245732	3.493217625	-1.512486026
H	11.953439248	2.689354600	0.038799315
H	13.016788227	1.419159400	-1.840074181
H	11.441083221	1.284792036	-2.666013793
H	11.941446451	-0.847683860	-1.469828815
H	12.166114209	0.034093069	0.065535814
H	9.889755876	-1.006070981	-0.015285598
H	9.530614284	-0.167947271	-1.536749284
C	7.590092154	0.249665961	2.016040470
C	8.188984061	0.921065684	3.272259644
C	7.780229668	0.228930157	4.587570924
C	8.122937925	-1.272282868	4.567600013
C	7.527915574	-1.966365522	3.328560213
C	7.946929867	-1.252051189	2.026289305

H	6.477148525	0.307889905	2.144283703
H	7.895447536	1.992275935	3.287380047
H	9.304667823	0.910565632	3.199861751
H	8.260432197	0.718450695	5.468165682
H	6.679532152	0.343176239	4.731584189
H	7.777466251	-1.770983846	5.503292892
H	9.233390928	-1.388105157	4.545679824
H	7.825552217	-3.041665503	3.311578757
H	6.415108472	-1.952746193	3.407729808
H	7.491310320	-1.769461197	1.152104794
H	9.051146464	-1.370887269	1.907209210
F	7.493466982	2.494257548	0.902749658
F	-5.631811312	5.667037372	-1.033154548

9.35. $[(\{Cy_2B\}FCH_2CH_2CH_2SiMe_2)_3P_7]^{3-}$

Charge = –3 Multiplicity = 1

P	0.040603690	0.593569151	0.263084281
P	0.551945095	1.856118496	-1.499715100
P	0.902299222	-1.345742444	-0.442040455
P	-2.106578718	0.210137341	-0.239558183
P	-0.074331594	-1.594193062	-2.435090382
P	-1.983028610	-0.362373764	-2.398412620
P	-0.061500624	0.542879294	-3.206532596
Si	2.862343548	2.081857377	-1.783263807
Si	-0.010973986	-3.094136872	0.823246783
Si	-3.249561628	2.257676121	-0.295797056
C	-2.916162985	3.266232532	-1.869434903
C	-2.614066230	3.205526900	1.227155024
C	-5.105562885	1.852201860	-0.130943777
C	-1.809530377	-3.503426073	0.375458349
C	0.082419920	-2.478004620	2.621853613
C	1.123264718	-4.602182630	0.550534012
C	3.535582974	3.327388192	-0.517577897
C	2.936836358	2.830506810	-3.538965300
C	3.835624865	0.451794165	-1.782631445
H	2.921890990	4.254589966	-0.579038854
C	5.030212977	3.682113165	-0.748277813
H	3.373624382	2.930860878	0.510521026
H	3.775854829	-0.057793783	-0.799936990
H	4.903251284	0.670106480	-2.004122059

H	3.448797185	-0.253560851	-2.546401404
H	2.346889087	3.769070348	-3.597556858
H	2.545452234	2.124731323	-4.301321551
H	3.991478180	3.074829094	-3.790708432
H	-0.206446526	-3.302072717	3.309290007
H	-0.601253704	-1.620228482	2.789639166
H	1.112573708	-2.158413815	2.884275441
H	2.165501409	-4.282998582	0.780152201
H	1.121142924	-4.836870734	-0.538323025
C	0.722654574	-5.837040696	1.402892888
H	-2.175231327	-4.320034243	1.034848339
H	-1.886069627	-3.852238099	-0.675687950
H	-2.463894066	-2.615797639	0.495449773
H	-3.200430590	4.141476112	1.350987130
H	-1.542578939	3.472138246	1.118039675
H	-2.730386067	2.603873710	2.152764377
H	-3.475840857	4.224863810	-1.815045552
H	-3.261430719	2.717899045	-2.770969073
H	-1.833321048	3.478514298	-1.982216032
H	-5.382900569	1.180922876	-0.976055773
C	-6.008101952	3.115936758	-0.117871741
H	-5.237385663	1.237112637	0.787963482
H	5.187820869	3.892864746	-1.835602252
H	5.658372359	2.783630564	-0.537064811
H	-0.350773232	-6.081925988	1.205637740
H	0.745209516	-5.540744141	2.480114121
H	-5.836099819	3.676444935	-1.068546478
H	-5.648903208	3.811539214	0.681797307
B	-7.675219417	3.005942022	-0.010103321
B	5.573041998	5.009005458	0.100474128
B	1.603502962	-7.260260579	1.352627442
C	-8.252477181	2.902751284	1.564042209
C	-8.288337777	4.286767589	2.253401411
C	-8.905472911	4.255664778	3.665345268
C	-8.192766355	3.237133414	4.573637259
C	-8.142414188	1.845902930	3.917260748
C	-7.530953633	1.907851991	2.501058782
H	-9.322312550	2.563746823	1.503006202
H	-8.827478693	5.007271836	1.603629726
H	-7.244852208	4.679101231	2.331962764

H	-8.886060573	5.267740180	4.137054709
H	-9.983052418	3.971730328	3.585353168
H	-8.680744192	3.186043594	5.575859709
H	-7.148333241	3.586139277	4.754370064
H	-7.577236481	1.136429505	4.566460906
H	-9.181732857	1.443262021	3.846864923
H	-7.513668270	0.886887321	2.063205054
H	-6.461335651	2.211745198	2.596623135
C	-8.324150430	1.846899509	-1.036090418
C	-9.764926456	2.209455405	-1.467441707
C	-10.358247607	1.242830624	-2.510908168
C	-10.307948887	-0.217156380	-2.024812900
C	-8.883798499	-0.610477750	-1.593445001
C	-8.311131717	0.378500870	-0.555613447
H	-7.703666769	1.881821672	-1.971627957
H	-9.781616495	3.253750579	-1.843333076
H	-10.425715378	2.203901542	-0.565021901
H	-11.407525340	1.523738646	-2.771895106
H	-9.771306375	1.324978142	-3.457122191
H	-10.691218792	-0.912089277	-2.809070036
H	-10.992766055	-0.328174565	-1.149604341
H	-8.876892947	-1.653576576	-1.198479062
H	-8.223121973	-0.615551766	-2.492493128
H	-7.284377938	0.061495318	-0.273448671
H	-8.922215179	0.294538906	0.375718728
C	3.260798458	-7.012459785	1.449286890
C	4.004532089	-6.642011528	0.146520812
C	5.513329426	-6.389145132	0.352929526
C	6.203151288	-7.591593219	1.021517110
C	5.487483192	-7.988046093	2.325782413
C	3.980461779	-8.217771356	2.099350008
H	3.403484176	-6.150330191	2.154812753
H	3.545284105	-5.751836227	-0.334301639
H	3.890723880	-7.476849334	-0.586980862
H	6.014630306	-6.148236977	-0.613994826
H	5.645324700	-5.491145046	1.001628327
H	7.281909485	-7.376719156	1.208062716
H	6.175821135	-8.459096502	0.318546819
H	5.972111601	-8.891582749	2.769267672
H	5.622019787	-7.167520520	3.070768770

H	3.473503592	-8.474848310	3.052791688
H	3.854790109	-9.113267904	1.441032545
C	1.156981876	-8.295010015	0.106704771
C	0.973777495	-7.658571977	-1.289700033
C	0.604307395	-8.681194522	-2.385428738
C	-0.654441124	-9.483401937	-2.009694333
C	-0.508342625	-10.130327950	-0.620215485
C	-0.122173975	-9.092858596	0.452147796
H	1.974748552	-9.059048086	0.002812114
H	1.884517492	-7.102270002	-1.598693206
H	0.160107695	-6.896757773	-1.236357405
H	0.457345619	-8.177235797	-3.369649821
H	1.455927096	-9.390733528	-2.522368655
H	-0.881919024	-10.252257850	-2.785841546
H	-1.529063778	-8.790665525	-1.993390207
H	-1.450673620	-10.663167159	-0.345883530
H	0.286757645	-10.913561397	-0.671535422
H	0.003280662	-9.578209756	1.442467605
H	-0.972184502	-8.378016546	0.575761728
C	7.067736212	5.523983669	-0.479942609
C	6.921374040	6.411620295	-1.737395427
C	8.259400302	6.985226789	-2.243610680
C	9.290657802	5.871977772	-2.504861545
C	9.462403178	4.969977196	-1.269069762
C	8.107369848	4.418671982	-0.776055607
H	7.522173842	6.181895508	0.308581176
H	6.196093651	7.226067474	-1.530582041
H	6.462648275	5.807130063	-2.557796262
H	8.114651379	7.595819652	-3.167287578
H	8.671875340	7.681301833	-1.473474968
H	10.270629296	6.301787758	-2.821915379
H	8.935166740	5.246525443	-3.358323079
H	10.174921399	4.141323434	-1.496255794
H	9.931311689	5.567777479	-0.450569898
H	8.272613963	3.778539039	0.117679202
H	7.701629277	3.736220422	-1.560413329
C	5.542119156	4.763946765	1.753939238
C	5.713419123	6.076549547	2.550817864
C	5.545660516	5.894479394	4.072421074
C	6.490857179	4.808666756	4.619655253

C	6.329983909	3.485741875	3.848153364
C	6.496105824	3.695260748	2.328136358
H	4.501830023	4.408239454	1.974599732
H	4.995192402	6.831535142	2.165820084
H	6.731875085	6.496240552	2.356667478
H	5.712133963	6.855508671	4.616345440
H	4.492901442	5.594312172	4.288588779
H	6.325670903	4.651681113	5.711578697
H	7.545192470	5.161798821	4.512257651
H	7.053654276	2.728006468	4.231756905
H	5.313239571	3.073253755	4.048448117
H	6.360211783	2.725201347	1.799751377
H	7.553480868	4.000987300	2.135636830
F	4.617864041	6.107273419	-0.151384761
F	-8.152414897	4.308324990	-0.535788895
F	1.234185916	-7.951789143	2.611546642

9.36. $[(\{Cy_2B\}CH_2CH_2CH_2SiMe_2)_2(\{Cy_2B\}HCH_2CH_2CH_2SiMe_2)P_7]^-$

Charge = –1 Multiplicity = 1

P	0.417208156	-0.871410342	0.545944172
P	1.290617341	0.858927458	-0.538467157
P	0.066054110	-2.202457528	-1.208124354
P	-1.672293459	-0.116242295	0.759852052
P	-1.131953121	-0.903768520	-2.583967406
P	-2.127317010	0.676135068	-1.289214942
P	-0.115457870	1.097999404	-2.258582729
Si	3.339459175	0.134551746	-1.445753980
Si	-1.473549714	-3.799343208	-0.537088253
Si	-1.489085670	1.824958270	2.020505223
C	-0.188267182	1.519097032	3.366815278
C	-3.204175313	2.014153022	2.825323773
C	-1.079879269	3.390514413	1.006428748
C	-0.940504370	-4.402560355	1.180940233
C	-1.262555616	-5.199263226	-1.806580595
C	-3.293856367	-3.213839232	-0.530340407
C	4.301068789	-0.733466211	-0.071852795
C	4.122508076	1.796682043	-1.941926058
C	3.105749111	-0.918565702	-3.006452149
H	4.311454078	-0.089194853	0.836730171
C	5.754174873	-1.066972102	-0.496839123

H	3.751387441	-1.655349140	0.226519826
H	2.573353946	-1.863843314	-2.776117158
H	4.107561962	-1.171902193	-3.415410990
H	2.534710201	-0.375900416	-3.788446623
H	4.253942031	2.455577464	-1.058638714
H	3.511112130	2.335415445	-2.695899401
H	5.127000993	1.610243214	-2.378513481
H	-1.904502749	-6.068621096	-1.547215129
H	-0.208764934	-5.546069252	-1.834054689
H	-1.536520579	-4.862244757	-2.828419158
H	-3.491938805	-2.715525378	-1.505679012
H	-3.370909163	-2.410805444	0.236086291
C	-4.335228678	-4.353269940	-0.274429812
H	-1.555270504	-5.273943630	1.494301088
H	-1.057921816	-3.601911087	1.939887466
H	0.123498561	-4.717682513	1.179381854
H	-3.227817888	2.895126861	3.502543956
H	-3.459252320	1.114985142	3.423860242
H	-3.996986513	2.149296239	2.059765248
H	-0.172591835	2.365596027	4.086974650
H	0.825237098	1.417305332	2.927518272
H	-0.409753421	0.592120687	3.935250499
H	-0.109291444	3.201195208	0.497035456
C	-1.024502426	4.701981744	1.856331760
H	-1.837359946	3.473556605	0.194717610
H	6.255757910	-0.124047982	-0.826230802
H	5.728469347	-1.718052289	-1.406585736
H	-4.083490684	-4.894872366	0.663727116
H	-4.274124005	-5.091626626	-1.106379418
H	-0.236825684	4.589105628	2.636806918
H	-1.990109208	4.852383333	2.387290743
B	-0.646052744	5.886779706	0.868819718
B	6.664494470	-1.760052611	0.722194328
B	-5.755346154	-3.648492894	-0.246573724
C	-1.749636127	6.949268137	0.410654712
C	-2.255456995	7.787203324	1.620124525
C	-3.326623425	8.815267875	1.209227107
C	-4.507793586	8.145997882	0.485323651
C	-4.029937730	7.311203044	-0.715392368
C	-2.958779492	6.283920299	-0.303879567

H	-1.301148931	7.670324157	-0.314436596
H	-1.405128048	8.307212366	2.114173004
H	-2.685412690	7.103624484	2.388453039
H	-3.685484717	9.373942681	2.102211010
H	-2.864847731	9.571973032	0.533159419
H	-5.248342998	8.909671350	0.159830238
H	-5.042924883	7.479738907	1.201717923
H	-4.890832521	6.796395286	-1.196372497
H	-3.603464434	7.992549286	-1.487892428
H	-2.616877097	5.717928030	-1.197623419
H	-3.421913589	5.532784945	0.377120089
C	0.859963418	6.002551094	0.359325282
C	1.520344726	7.231726495	1.055093877
C	2.992301489	7.411615909	0.636301812
C	3.138111571	7.510512681	-0.891918733
C	2.495285130	6.303483453	-1.596452845
C	1.024863150	6.115812806	-1.178766098
H	1.433176046	5.103213677	0.688797163
H	1.453203637	7.135227902	2.161941136
H	0.954962535	8.155884924	0.788768096
H	3.421910679	8.313176828	1.127181312
H	3.583392508	6.542494666	1.005388147
H	4.210332227	7.599794302	-1.173111130
H	2.644222962	8.446591567	-1.244751548
H	2.569409771	6.415413808	-2.700588864
H	3.066959461	5.382482731	-1.340361801
H	0.606907434	5.212342768	-1.673739446
H	0.428166085	6.979998994	-1.554112394
C	-6.394009541	-3.150816942	-1.624132381
C	-6.682311762	-1.624792541	-1.651254504
C	-7.336141792	-1.177651074	-2.971988936
C	-8.616389571	-1.976032177	-3.272748487
C	-8.349911360	-3.491227860	-3.256218376
C	-7.700048202	-3.938827446	-1.932900279
H	-5.684041372	-3.365733645	-2.459307629
H	-5.743330708	-1.053511616	-1.485012730
H	-7.358469685	-1.361705631	-0.804650261
H	-7.556908402	-0.087946691	-2.941157217
H	-6.609899782	-1.322844002	-3.804733058
H	-9.047762005	-1.668381352	-4.250835041

H	-9.386858628	-1.734242689	-2.503379076
H	-9.294354423	-4.053721707	-3.429650483
H	-7.671096650	-3.752477715	-4.100780529
H	-7.496151921	-5.032469722	-1.965063418
H	-8.429808923	-3.783986342	-1.103895339
C	-6.531694952	-3.434498811	1.133032558
C	-5.687756672	-2.714373820	2.219454262
C	-6.447700393	-2.576521898	3.551700511
C	-6.938185260	-3.939395259	4.070177860
C	-7.778124706	-4.673438262	3.010876082
C	-7.018061867	-4.810869319	1.677773190
H	-7.446607437	-2.816449269	0.964521434
H	-5.368094719	-1.712096208	1.860846631
H	-4.748407750	-3.286859480	2.396907291
H	-5.803070057	-2.082879536	4.311823364
H	-7.325053200	-1.904442820	3.403889553
H	-7.520605625	-3.813663189	5.009525962
H	-6.055545020	-4.567585408	4.333677454
H	-8.082054325	-5.676954842	3.383532019
H	-8.722082408	-4.106941417	2.834800422
H	-7.663254508	-5.320677951	0.928428635
H	-6.137749478	-5.477524076	1.830017744
C	8.302531687	-1.677188486	0.374841078
C	8.916088471	-0.318283346	0.787624322
C	10.434200471	-0.210803290	0.537253912
C	10.794456669	-0.531573660	-0.924002385
C	10.223386151	-1.896156004	-1.347648138
C	8.703956499	-1.976276160	-1.090945932
H	8.816905361	-2.450779795	1.004862944
H	8.689417301	-0.121131530	1.858118273
H	8.403454361	0.498603691	0.221700805
H	10.816915957	0.799952252	0.814376105
H	10.963051853	-0.934875146	1.202493850
H	11.898257438	-0.497963179	-1.077738314
H	10.364638217	0.259155499	-1.584434668
H	10.459759415	-2.095239328	-2.419691954
H	10.735365880	-2.697586397	-0.763040191
H	8.326599833	-2.973950395	-1.404600638
H	8.200186214	-1.242096793	-1.764956655
C	6.115083282	-3.286597505	1.116485344

C	6.643093801	-3.776842168	2.483870576
C	6.076823767	-5.145154165	2.915160051
C	6.305676205	-6.220190690	1.837377218
C	5.761808413	-5.764558124	0.471414023
C	6.345951621	-4.395687036	0.063583681
H	5.001794052	-3.202446697	1.237341215
H	6.426548173	-3.009354385	3.259545171
H	7.757383551	-3.852790883	2.440438004
H	6.517266458	-5.475787899	3.885457397
H	4.979101709	-5.044403801	3.091904633
H	5.849053549	-7.190656784	2.141890779
H	7.401750404	-6.408866774	1.742591333
H	5.971851723	-6.539526563	-0.303190372
H	4.650370838	-5.682495539	0.535531271
H	5.928951640	-4.088975056	-0.922574675
H	7.443457721	-4.522849467	-0.098785399
H	6.469554144	-1.048966375	1.750546899

9.37. $[(\{Cy_2B\}CH_2CH_2CH_2SiMe_2)(\{Cy_2B\}HCH_2CH_2CH_2SiMe_2)_2P_7]^{2-}$

Charge = –2 Multiplicity = 1

P	1.465209780	0.257833473	0.082069830
P	2.288196154	1.088514072	-1.810628404
P	1.741916643	-1.902732307	-0.407995846
P	-0.716296859	0.403119140	-0.363034608
P	0.664653202	-2.104803370	-2.364884701
P	-0.825092061	-0.390039186	-2.449494418
P	1.240986055	-0.142218512	-3.355440857
Si	4.537164802	0.441794335	-2.023624215
Si	0.353951428	-3.061961589	1.034272774
Si	-1.296386039	2.668971370	-0.605490731
C	-0.688285459	3.399648118	-2.247267411
C	-0.419363098	3.520577332	0.851119998
C	-3.186181926	2.764814508	-0.446208747
C	0.489849210	-2.257657298	2.749271959
C	1.114599764	-4.809064782	1.099311510
C	-1.472403204	-3.201728355	0.483298950
C	5.496963244	0.852296327	-0.443198782
C	5.070778842	1.552609446	-3.479341497
C	4.743034447	-1.370652161	-2.557850905
H	5.262110258	1.894212627	-0.127600823

C	7.030598288	0.687284081	-0.620320151
H	5.118565828	0.204642367	0.380502848
H	4.373202401	-2.061368772	-1.772727671
H	5.823025648	-1.572907468	-2.725914810
H	4.192632418	-1.586055872	-3.497500378
H	4.963676902	2.626718514	-3.220950279
H	4.468533195	1.352242931	-4.390551778
H	6.139414164	1.362674899	-3.717384765
H	0.577041897	-5.454100522	1.827786484
H	2.180604307	-4.757922796	1.404360059
H	1.070636009	-5.301686361	0.104893421
H	-1.476555938	-3.516257944	-0.584472006
H	-1.890712612	-2.170719239	0.492533163
C	-2.331875297	-4.179685424	1.350406866
H	-0.040583870	-2.869533389	3.510909486
H	0.043514098	-1.242071302	2.743536921
H	1.550708451	-2.163117428	3.061226953
H	-0.732830129	4.585916764	0.890680321
H	0.684314349	3.482602901	0.743658810
H	-0.692889524	3.050583976	1.818699404
H	-0.961952781	4.475899718	-2.286967876
H	-1.170452200	2.893580791	-3.109749173
H	0.411440417	3.298229880	-2.350995942
H	-3.637118329	2.125589289	-1.239611030
C	-3.719517910	4.224052031	-0.555381466
H	-3.473915298	2.289136688	0.518008220
H	7.357430804	1.332883087	-1.472621743
H	7.246090165	-0.359122526	-0.955360725
H	-2.275849650	-3.884365545	2.421286392
H	-1.900998574	-5.204931339	1.271301006
H	-3.404892280	4.620458851	-1.550812587
H	-3.173435745	4.864718862	0.182459431
B	-5.363609624	4.531033384	-0.415218833
B	7.932931912	1.082419283	0.727001485
B	-3.802700457	-4.151460120	0.758551403
C	-5.913062988	4.655863333	1.165263484
C	-5.633631206	6.055245347	1.764449275
C	-6.183756836	6.253560236	3.191765206
C	-5.680584362	5.161948684	4.152652113
C	-5.974381243	3.758256294	3.595613312

C	-5.411822911	3.587706335	2.168025261
H	-7.031949648	4.555996712	1.134154625
H	-6.042304953	6.834003807	1.084061855
H	-4.529218740	6.227749404	1.781392324
H	-5.921972004	7.263847872	3.587685427
H	-7.299413187	6.213346152	3.158836459
H	-6.126090962	5.288267437	5.167366062
H	-4.577674034	5.274126847	4.281475139
H	-5.566629799	2.980010570	4.283293572
H	-7.080036514	3.604222121	3.572399031
H	-5.643838823	2.564682387	1.799192010
H	-4.298123257	3.642343367	2.227029689
C	-6.302161304	3.541652457	-1.389269225
C	-7.589140905	4.257073358	-1.865998468
C	-8.461700425	3.419170107	-2.822961796
C	-8.815958346	2.049413023	-2.217695267
C	-7.549071922	1.301237341	-1.767838248
C	-6.695086788	2.160242673	-0.810835115
H	-5.701766216	3.337223792	-2.316339573
H	-7.316494869	5.223800953	-2.343215438
H	-8.203527545	4.530108964	-0.972390516
H	-9.392773454	3.967544509	-3.103745175
H	-7.900998182	3.254047188	-3.774287613
H	-9.405088519	1.435713408	-2.939095828
H	-9.475909898	2.206071788	-1.330816862
H	-7.825286941	0.328763431	-1.295773824
H	-6.942187587	1.046839982	-2.669358092
H	-5.788862124	1.589567719	-0.512530528
H	-7.278327484	2.314301495	0.129270817
C	-4.079886950	-4.872627500	-0.641962616
C	-4.629939331	-3.907449225	-1.727313642
C	-4.934199829	-4.631689495	-3.051932971
C	-5.892861911	-5.817295823	-2.846141008
C	-5.361554035	-6.789719397	-1.778424983
C	-5.059909875	-6.065547317	-0.452152656
H	-3.124768504	-5.299268550	-1.034297486
H	-3.908982098	-3.081787669	-1.908505409
H	-5.562804959	-3.424126275	-1.355624173
H	-5.356166799	-3.915992564	-3.791138707
H	-3.979920515	-5.005020229	-3.490725211

H	-6.066436917	-6.352081081	-3.806622570
H	-6.887124154	-5.430041901	-2.521572174
H	-6.088037244	-7.616515350	-1.609339914
H	-4.425976607	-7.267095432	-2.152518368
H	-4.651991809	-6.790773148	0.287588866
H	-6.018059162	-5.690683869	-0.022009501
C	-4.986150123	-3.424683763	1.544745173
C	-4.676576172	-1.940217869	1.884952444
C	-5.810166059	-1.279302303	2.689750938
C	-6.131329976	-2.066828600	3.971287057
C	-6.444027745	-3.541039109	3.660963495
C	-5.311700459	-4.204335940	2.853259929
H	-5.917439760	-3.431599343	0.928252578
H	-4.488416268	-1.359502222	0.956998258
H	-3.732624239	-1.885598664	2.474627543
H	-5.543228946	-0.228837265	2.933618659
H	-6.723994260	-1.223818947	2.054560383
H	-6.979127281	-1.596171576	4.516530879
H	-5.254824636	-2.018911225	4.659269520
H	-6.626129966	-4.108377578	4.601785620
H	-7.390204127	-3.597907157	3.073676255
H	-5.582468286	-5.259161625	2.621933021
H	-4.396377237	-4.253835502	3.487916957
C	9.548543339	1.320943669	0.332537200
C	9.823067689	2.762928129	-0.155054068
C	11.304298522	3.047123760	-0.479585113
C	11.871443498	2.032105344	-1.488012266
C	11.639148973	0.588119921	-1.009379863
C	10.150277826	0.330563021	-0.694997967
H	10.146392078	1.196366914	1.275099652
H	9.451694006	3.485787003	0.603714177
H	9.213642086	2.958216527	-1.071686076
H	11.441078564	4.086355988	-0.863184896
H	11.900214442	2.984091061	0.462838624
H	12.955058219	2.219858194	-1.675182645
H	11.357729972	2.172342220	-2.469114405
H	12.020381758	-0.134069258	-1.770355179
H	12.242991545	0.414024830	-0.086348578
H	10.020015502	-0.718332704	-0.350176389
H	9.576661648	0.402865360	-1.650155667

C	7.692228497	0.002914737	1.980309496
C	8.145833837	0.561839212	3.347907384
C	7.854013804	-0.379950836	4.534242410
C	8.448390294	-1.781726864	4.307019594
C	7.985950181	-2.371155272	2.962125157
C	8.292095571	-1.410189586	1.793937418
H	6.582285567	-0.139803248	2.064810625
H	7.668312119	1.552082317	3.517417917
H	9.245771030	0.760759570	3.316126633
H	8.235602301	0.046920968	5.492394751
H	6.748873846	-0.476618003	4.656930917
H	8.188099594	-2.464530201	5.149603646
H	9.562421912	-1.705159880	4.301647106
H	8.457708576	-3.369367220	2.798458524
H	6.885531542	-2.551162644	3.007340146
H	7.938622843	-1.858734378	0.837921254
H	9.402040776	-1.326608405	1.698171232
H	7.492261675	2.193146390	1.141907109
H	-5.492455500	5.691992789	-0.903101413

9.38. $[(\{Cy_2B\}HCH_2CH_2CH_2SiMe_2)_3P_7]^{3-}$

Charge = –3 Multiplicity = 1

P	0.050658180	0.584522225	0.295043231
P	0.644602973	1.828840510	-1.454280153
P	0.786753037	-1.401045012	-0.426270202
P	-2.116031459	0.345410568	-0.213028457
P	-0.199829977	-1.564869028	-2.423237992
P	-2.024953540	-0.212063226	-2.377669264
P	-0.046731486	0.573537437	-3.174810293
Si	2.965198200	1.924796585	-1.728189743
Si	-0.256586668	-3.092618441	0.820808609
Si	-3.127731119	2.462107955	-0.239459344
C	-2.717847286	3.472960635	-1.794600863
C	-2.432070987	3.339922199	1.299902115
C	-5.007649959	2.185999280	-0.086794827
C	-2.064262794	-3.388677892	0.321189852
C	-0.180201843	-2.470693740	2.619026483
C	0.787289929	-4.670761721	0.591665314
C	3.688600772	3.166990366	-0.483673362
C	3.089573232	2.627001586	-3.500717797

C	3.854162770	0.248000421	-1.682577214
H	3.067064492	4.091571279	-0.511544477
C	5.175383632	3.526544774	-0.759888914
H	3.562124795	2.763429900	0.547180203
H	3.753776327	-0.238553962	-0.691558055
H	4.934917817	0.408121474	-1.889971330
H	3.443704087	-0.451782689	-2.439034012
H	2.553591372	3.595620482	-3.585260922
H	2.661568439	1.926857122	-4.248437640
H	4.157526603	2.805377823	-3.751454837
H	-0.543341054	-3.269398012	3.301157839
H	-0.811932375	-1.569484633	2.760506561
H	0.859792245	-2.216676849	2.912658128
H	1.841069764	-4.416489364	0.849301057
H	0.803073271	-4.919354672	-0.493721540
C	0.285741599	-5.872133191	1.448080900
H	-2.497764225	-4.184107668	0.965253455
H	-2.132920891	-3.727331244	-0.733876852
H	-2.665583672	-2.462686875	0.428897280
H	-2.951424484	4.312993428	1.436178957
H	-1.343805216	3.531209313	1.198767553
H	-2.595301742	2.734400856	2.215880728
H	-3.212026551	4.466127455	-1.725935146
H	-3.095045406	2.964363857	-2.706624178
H	-1.622636339	3.613528761	-1.900100428
H	-5.331629538	1.565861499	-0.954386631
C	-5.819702944	3.515119636	-0.034229361
H	-5.190164045	1.552320896	0.810133670
H	5.272500193	3.815650625	-1.836188744
H	5.790594439	2.596013088	-0.663086621
H	-0.794675021	-6.049366982	1.216140099
H	0.282893714	-5.541649828	2.515626042
H	-5.576922349	4.089558400	-0.961017973
H	-5.411934250	4.150197202	0.792788981
B	-7.492786671	3.499102785	0.083959682
B	5.795855201	4.766012303	0.167070945
B	1.085520188	-7.346548327	1.410225523
C	-8.084158758	3.312497271	1.644781708
C	-8.079835274	4.647074207	2.428280030
C	-8.682185565	4.550395958	3.845347910

C	-8.001814862	3.450018277	4.679056548
C	-8.018708157	2.103068823	3.935418233
C	-7.409815013	2.229930400	2.522331369
H	-9.165331379	3.016866543	1.556379474
H	-8.614003352	5.422495622	1.836219696
H	-7.027102702	5.013103639	2.513380246
H	-8.619807897	5.529841503	4.378470180
H	-9.770586112	4.312436665	3.761013990
H	-8.480582368	3.357263385	5.682785528
H	-6.941507223	3.743215854	4.866612459
H	-7.484551615	1.328034363	4.534150095
H	-9.076147973	1.754293284	3.847252450
H	-7.439390200	1.240125396	2.017980174
H	-6.327177259	2.477863621	2.632801745
C	-8.227879330	2.497522577	-1.042508756
C	-9.622309483	3.023274186	-1.459418618
C	-10.319463889	2.178358463	-2.545917207
C	-10.415274764	0.696886892	-2.139810769
C	-9.033457445	0.139866178	-1.755218825
C	-8.359430889	1.001796224	-0.666206216
H	-7.593438830	2.536736712	-1.968244096
H	-9.531292742	4.079886696	-1.795003085
H	-10.282307656	3.054212555	-0.556774234
H	-11.335523049	2.579512564	-2.780205316
H	-9.732257063	2.250273344	-3.492955540
H	-10.876897616	0.089854811	-2.954398259
H	-11.097674492	0.607459239	-1.260236635
H	-9.125591014	-0.922244257	-1.426515006
H	-8.385082121	0.127364933	-2.663412580
H	-7.364495585	0.572812168	-0.419776854
H	-8.966876998	0.914876945	0.267441350
C	2.742877137	-7.210668733	1.626869815
C	3.588720593	-6.917078814	0.364024040
C	5.102924825	-6.795945115	0.638867300
C	5.649820908	-8.049131722	1.344503217
C	4.848673445	-8.356363301	2.622180522
C	3.335471581	-8.451370938	2.337000484
H	2.906135209	-6.346875247	2.325552893
H	3.230202356	-5.993330339	-0.138871005
H	3.436515383	-7.742385027	-0.373557861

H	5.668427891	-6.609041668	-0.304541521
H	5.285775224	-5.908124653	1.289596410
H	6.735879898	-7.935213089	1.573407805
H	5.567593907	-8.919430172	0.649302231
H	5.229616452	-9.291947037	3.099338674
H	5.027832721	-7.537631268	3.359896831
H	2.777919033	-8.635093605	3.281813875
H	3.150508521	-9.354061915	1.702721516
C	0.651108493	-8.344495125	0.130762392
C	0.536797560	-7.682018498	-1.263786647
C	0.155768791	-8.664569572	-2.392022482
C	-1.150269951	-9.412141923	-2.070806706
C	-1.069561636	-10.101055823	-0.696841980
C	-0.662734231	-9.111385833	0.414630389
H	1.448073772	-9.132374219	0.039792900
H	1.482053751	-7.162379457	-1.531162548
H	-0.242561129	-6.884298347	-1.221724178
H	0.063834299	-8.135808045	-3.370057415
H	0.976354509	-9.412024075	-2.517614781
H	-1.395099679	-10.149232278	-2.872179656
H	-1.990193892	-8.677606330	-2.057337891
H	-2.040916934	-10.598437192	-0.459234308
H	-0.308572074	-10.917369251	-0.751240370
H	-0.575399114	-9.642304075	1.388100865
H	-1.489789354	-8.372508764	0.553761813
C	7.223516839	5.371242265	-0.488134907
C	6.956714878	6.440544065	-1.573419368
C	8.234150239	7.065431751	-2.172279576
C	9.192262108	5.990211951	-2.715489410
C	9.500428797	4.930766268	-1.642374767
C	8.206286411	4.321303448	-1.062256143
H	7.775554810	5.900640499	0.334839089
H	6.301553279	7.235222769	-1.154297249
H	6.365374490	5.977119047	-2.401021623
H	7.986849041	7.800103237	-2.976137484
H	8.762889435	7.643713685	-1.375997857
H	10.132397774	6.451966356	-3.101191583
H	8.711959670	5.487372012	-3.588701738
H	10.164384528	4.139132698	-2.065491115
H	10.079020617	5.413464948	-0.817814381

H	8.466577904	3.565734064	-0.289419512
H	7.695885111	3.750975577	-1.874638044
C	5.918764978	4.354866467	1.783069937
C	6.077150957	5.584078625	2.706106044
C	6.106479759	5.237239288	4.209182589
C	7.185753587	4.186784242	4.528921326
C	7.027712367	2.938914871	3.641382535
C	7.004321674	3.314773045	2.144675908
H	4.936610925	3.889937469	2.060933433
H	5.260483599	6.309660804	2.495408969
H	7.026287398	6.117056637	2.448264250
H	6.264157487	6.150421209	4.832538889
H	5.111728668	4.827696420	4.506060276
H	7.162897733	3.910586146	5.609546497
H	8.191992539	4.635094809	4.343436122
H	7.840439296	2.206347246	3.861849069
H	6.070925293	2.429036177	3.904248248
H	6.878642183	2.395538241	1.529570249
H	8.008845140	3.725152092	1.877030115
H	4.954470144	5.711131913	0.120794577
H	-7.831620293	4.673555269	-0.255205032
H	0.668815303	-7.944987787	2.447984193

9.39. ($\{(C_6F_5)_2B\}CH_2CH_2SiMe_2)_3P_7$ (**6**)

Charge = 0 Multiplicity = 1

P	-0.645368454	-1.197412357	-0.164940596
P	-1.306800713	0.462433996	1.165121115
P	0.088137963	-2.586977054	1.413028114
P	1.314172359	-0.325343935	-0.770016399
P	1.520142633	-1.307774241	2.568816362
P	2.164230642	0.370624240	1.185361022
P	0.408257863	0.669703236	2.591911250
Si	-3.003869373	-0.394999930	2.500861574
Si	1.490608521	-4.108356802	0.351399155
Si	0.814747439	1.666314328	-1.872826114
C	-0.780874639	1.390719356	-2.859084228
C	2.271987392	1.917508857	-3.061459701
C	0.665301921	3.151212925	-0.684034780
C	0.669075309	-4.576556481	-1.291148047
C	1.534377596	-5.601077650	1.520215792

C	3.253198632	-3.425608760	0.080431962
C	-4.145751335	-1.350585135	1.298712123
C	-3.882172047	1.149255518	3.166799127
C	-2.446475133	-1.486650746	3.941714145
H	-4.353456734	-0.692966159	0.425556895
C	-5.482894326	-1.827071301	1.959976686
H	-3.568992077	-2.216122467	0.906810686
H	-1.938612784	-2.402861964	3.578417242
H	-3.327955740	-1.783503360	4.550452194
H	-1.744585898	-0.940553361	4.605645942
H	-4.245193673	1.799642298	2.344470742
H	-3.197072452	1.750758401	3.800365663
H	-4.752927723	0.862507215	3.794921033
H	2.139308950	-6.422025454	1.078630326
H	0.512450041	-5.991194421	1.706619351
H	1.984836893	-5.335419029	2.499116204
H	3.658160200	-3.141166881	1.076082989
H	3.147891983	-2.481205190	-0.497728761
C	4.218575406	-4.421938567	-0.647226708
H	1.226294825	-5.406993110	-1.775721946
H	0.648314289	-3.717385665	-1.992533448
H	-0.374773516	-4.917752021	-1.132207221
H	2.131710404	2.846014207	-3.655997272
H	2.349255494	1.071842141	-3.775878155
H	3.240631407	2.001193577	-2.527147353
H	-0.970771552	2.261809683	-3.522754394
H	-1.654822132	1.271869459	-2.186636806
H	-0.709121655	0.487566913	-3.499653103
H	-0.181527178	2.941610114	0.005268700
C	0.458036402	4.518055878	-1.399811092
H	1.579695320	3.173650035	-0.051548284
H	-6.038828523	-0.941782195	2.333441391
H	-5.262730602	-2.479288033	2.828539239
H	3.781581480	-4.707990663	-1.626754349
H	4.346102011	-5.343632919	-0.045965528
H	-0.433101563	4.478004627	-2.058639704
H	1.328469358	4.720650461	-2.068030415
B	0.373163318	5.756493410	-0.414408885
B	-6.324694253	-2.555959038	0.836815374
B	5.556476528	-3.609064685	-0.863505307

C	-7.070388289	-1.649815957	-0.233413040
C	-6.861378252	-1.820499290	-1.617430934
C	-7.940981280	-0.604621891	0.137658485
C	-7.460928825	-1.000721211	-2.587869125
C	-8.576628833	0.227879964	-0.800187048
C	-8.327636438	0.027827857	-2.171091214
C	-6.388236693	-4.128008070	0.709139218
C	-5.373543022	-4.980321018	1.212169359
C	-7.494944127	-4.790474532	0.118684911
C	-5.430733324	-6.381408349	1.127731980
C	-7.600298555	-6.188985848	0.035493293
C	-6.556298043	-6.987165695	0.538991915
C	5.562681025	-2.548614338	-2.047482155
C	5.267263302	-2.906999630	-3.376856390
C	5.778425955	-1.178197190	-1.804178172
C	5.184833901	-1.967286296	-4.419066241
C	5.684364223	-0.203445850	-2.811139817
C	5.384910596	-0.605105638	-4.126178063
C	6.808452166	-3.738744163	0.086333991
C	8.116639234	-3.397753356	-0.345113215
C	6.721909831	-4.237088995	1.411007610
C	9.256551905	-3.544009063	0.463014297
C	7.834203895	-4.378041367	2.257158776
C	9.110192485	-4.031882583	1.774828155
C	-0.856097487	6.748061982	-0.514006709
C	-0.695627746	8.151347811	-0.506273628
C	-2.180359092	6.278628739	-0.658599595
C	-1.770448197	9.043878215	-0.653057315
C	-3.285981241	7.136679752	-0.784787588
C	-3.074090109	8.528445763	-0.786015348
C	1.510577723	6.030809794	0.649984457
C	2.873860148	5.730061059	0.415106474
C	1.220959342	6.581036126	1.922293730
C	3.889329223	5.979746730	1.353464405
C	2.202623256	6.824002780	2.897068360
C	3.546009988	6.521559355	2.606167614
F	0.533300164	8.687726845	-0.388040650
F	-2.432137785	4.955745556	-0.638836999
F	-1.572937494	10.366768949	-0.663757268
F	-4.527402857	6.650742423	-0.897979559

F	-4.109360694	9.359992458	-0.914928672
F	4.493037722	6.750276800	3.515746177
F	5.166785660	5.700266369	1.075803964
F	3.266856485	5.212001877	-0.763382061
F	-0.046216199	6.875268308	2.262493724
F	1.877435486	7.330729949	4.090961413
F	-8.532458567	-4.088976400	-0.366357046
F	-6.036337103	-2.792898903	-2.053349958
F	-7.221598889	-1.183961936	-3.891361623
F	-8.914081407	0.813256398	-3.076958895
F	-9.411521410	1.196751880	-0.407847174
F	-8.228104812	-0.394118733	1.438333561
F	-4.267057102	-4.466343613	1.777520117
F	-4.435618460	-7.140325572	1.599230401
F	-6.634212090	-8.315082290	0.460298960
F	-8.673464036	-6.766308596	-0.514063120
F	5.532489241	-4.576951316	1.938326065
F	7.695511618	-4.836177433	3.505512984
F	10.178939601	-4.169118464	2.558562950
F	10.470767042	-3.225279012	0.003379553
F	8.329570792	-2.941218284	-1.589675788
F	6.057372481	-0.757772756	-0.555092943
F	5.078133056	-4.204146828	-3.696221480
F	4.914531486	-2.348282744	-5.673510162
F	5.285015659	0.307713764	-5.096634172
F	5.856451711	1.096268487	-2.533146249

9.40. $[(\{(C_6F_5)_2B\}CH_2CH_2CH_2SiMe_2)_2(\{(C_6F_5)_2B\}FCH_2CH_2CH_2SiMe_2)P_7]^-$

Charge = –1 Multiplicity = 1

P	0.475587445	-1.379135888	0.053773257
P	1.286866511	0.087684561	-1.415274916
P	-0.311397918	-2.857957859	-1.416435222
P	-1.412189654	-0.299440127	0.528151436
P	-1.638476625	-1.604650213	-2.716628014
P	-2.172465640	0.240366876	-1.506371028
P	-0.384491713	0.285130268	-2.895110637
Si	2.910980440	-1.011420498	-2.644471146
Si	-1.823191918	-4.174334521	-0.263608354
Si	-0.778159098	1.785131644	1.432551130
C	0.617763801	1.358612082	2.649841242

C	-2.334275618	2.281421119	2.406014447
C	-0.289670984	3.104601384	0.176217019
C	-1.023571487	-4.634303202	1.395263877
C	-2.031544691	-5.731097929	-1.334404498
C	-3.525695551	-3.347757134	0.011565084
C	3.971620180	-1.958082205	-1.354486299
C	3.925946854	0.390081450	-3.421160580
C	2.304827693	-2.187046522	-4.001729799
H	4.231402848	-1.245863154	-0.540516877
C	5.267185354	-2.596443923	-1.966303169
H	3.321082166	-2.734138400	-0.896809813
H	1.738871323	-3.037308120	-3.570160352
H	3.169121712	-2.583249270	-4.578017640
H	1.638473371	-1.653820246	-4.711368962
H	4.313960791	1.085606195	-2.648747438
H	3.298703070	0.984593222	-4.118162658
H	4.785380407	-0.012153736	-3.999912826
H	-2.697231721	-6.467492628	-0.834580119
H	-1.051962651	-6.220612179	-1.514741199
H	-2.476660811	-5.482374780	-2.320347816
H	-3.935895656	-3.087141583	-0.988145658
H	-3.330788547	-2.383451684	0.531119128
C	-4.533485422	-4.235151492	0.821228003
H	-1.642702031	-5.383754132	1.933922067
H	-0.911727880	-3.742388999	2.045532714
H	-0.016201874	-5.073554842	1.240524131
H	-2.157941699	3.267034129	2.887900613
H	-2.584319044	1.547817338	3.200260274
H	-3.216349406	2.391593949	1.741872801
H	0.866958316	2.262340776	3.246305505
H	1.536033016	1.044661137	2.112181128
H	0.326061932	0.550095652	3.351809298
H	0.576977973	2.736227644	-0.414610621
C	0.070806529	4.454841316	0.844157936
H	-1.129477958	3.234010500	-0.541648009
H	5.898814520	-1.791343274	-2.395105781
H	4.995968938	-3.290825214	-2.786388271
H	-4.088815678	-4.485260351	1.806484470
H	-4.732807235	-5.183320148	0.283272475
H	0.835277691	4.278849145	1.638101073

H	-0.812575267	4.895231830	1.353041507
B	0.777150824	5.521812032	-0.205003147
B	6.002264915	-3.300963022	-0.763251984
B	-5.807045713	-3.322878342	1.004339037
C	6.820005534	-2.389717137	0.249480772
C	6.567348322	-2.413821701	1.636159273
C	7.811097794	-1.486113610	-0.184433384
C	7.238135943	-1.584173922	2.549810803
C	8.519488591	-0.650409100	0.696698351
C	8.223830753	-0.699821846	2.071739900
C	5.899346502	-4.858036210	-0.494346447
C	4.810581558	-5.646788616	-0.941801563
C	6.925952587	-5.572434768	0.173140789
C	4.724956086	-7.034046036	-0.731377760
C	6.888020898	-6.961220081	0.382995181
C	5.775302698	-7.693737763	-0.068326794
C	-5.731619698	-2.174092125	2.097346559
C	-5.358219637	-2.426387083	3.432480428
C	-5.956151774	-0.823784649	1.759878093
C	-5.209229057	-1.406618227	4.387405211
C	-5.799886421	0.228697422	2.677154164
C	-5.421170351	-0.070470867	3.999267669
C	-7.093949801	-3.456300273	0.093303417
C	-8.377016983	-3.059788570	0.547817544
C	-7.063554600	-4.017906319	-1.207075483
C	-9.545916926	-3.208555392	-0.217639218
C	-8.206320384	-4.163711754	-2.012047447
C	-9.455834660	-3.759027125	-1.508862527
C	1.368111682	6.805387489	0.691391799
C	0.490839897	7.690183809	1.344438985
C	2.734619718	7.074217046	0.898319434
C	0.905738192	8.774480110	2.139286539
C	3.201202305	8.148182484	1.685251121
C	2.280855058	9.005143869	2.308853266
C	-0.216037371	6.157192773	-1.398470819
C	-1.609460884	6.021638994	-1.506597421
C	0.377315556	6.924108087	-2.421328085
C	-2.373947338	6.575867017	-2.553039027
C	-0.338464977	7.495886168	-3.489997867
C	-1.730700223	7.319906211	-3.553739056

F	-0.851701104	7.523201822	1.231081683
F	3.696313610	6.302822511	0.351455920
F	0.011171015	9.588658784	2.738322541
F	4.523830599	8.363111422	1.850172391
F	2.711191946	10.034110455	3.066713076
F	-2.439579557	7.858755126	-4.565450966
F	-3.711254490	6.403604318	-2.605636933
F	-2.322981002	5.325513678	-0.585304709
F	1.706553061	7.162439042	-2.412496519
F	0.285806110	8.219110228	-4.443145954
F	8.026435839	-4.939387004	0.615033569
F	5.632449289	-3.249443336	2.130331410
F	6.958482965	-1.627130880	3.858049333
F	8.882247586	0.089071251	2.924637785
F	9.469952579	0.177999473	0.247579083
F	8.147583508	-1.427303417	-1.489000658
F	3.771530361	-5.081757571	-1.577702179
F	3.664468326	-7.730744980	-1.156961376
F	5.718402914	-9.012958811	0.128976993
F	7.893148896	-7.591778642	1.003674682
F	-5.903269981	-4.421496545	-1.752450334
F	-8.120591024	-4.685338136	-3.241697911
F	-10.554901251	-3.902258605	-2.253038856
F	-10.737581968	-2.835342046	0.265260158
F	-8.539194146	-2.543437344	1.777715473
F	-6.311843977	-0.501146346	0.502206217
F	-5.162403993	-3.695006447	3.852012474
F	-4.868535648	-1.689197539	5.652226918
F	-5.262432903	0.913491104	4.888000459
F	-5.994561205	1.499371124	2.309798530
F	1.840504746	4.832165216	-0.874106378

9.41. $[(\{(C_6F_5)_2B\}CH_2CH_2CH_2SiMe_2)(\{(C_6F_5)_2B\}FCH_2CH_2CH_2SiMe_2)_2P_7]^{2-}$

Charge = –2 Multiplicity = 1

P	1.790843763	1.080912493	-0.722697438
P	1.982321825	2.336169342	-2.558165062
P	2.847883038	-0.674228114	-1.587095297
P	-0.307972533	0.359334060	-0.934595407
P	1.627044740	-1.178307090	-3.393825189
P	-0.398714405	-0.199722662	-3.094505862

P	1.254681633	0.934800141	-4.152865816
Si	4.303172417	2.459857031	-2.950018375
Si	2.565977633	-2.433206189	-0.134845497
Si	-1.740455569	2.213463050	-0.817576331
C	-1.596824951	3.350071718	-2.328684818
C	-1.186862886	3.131074185	0.751906392
C	-3.499633349	1.523189059	-0.641990864
C	2.971615559	-1.776212488	1.597454799
C	3.855372331	-3.717977941	-0.679598972
C	0.816727160	-3.219440663	-0.159637186
C	5.311522602	2.234299383	-1.356368381
C	4.482554588	4.227797944	-3.637886176
C	4.834837736	1.232747205	-4.299516765
H	4.905058938	2.909875116	-0.570145427
C	6.822826525	2.489206000	-1.573107137
H	5.128134766	1.199832570	-0.997799602
H	4.743032119	0.189277884	-3.936257021
H	5.900259703	1.415241144	-4.557982486
H	4.226425566	1.343268911	-5.221126253
H	4.152579018	4.981037096	-2.892094268
H	3.878344053	4.369808226	-4.558749709
H	5.546734322	4.432073338	-3.884239368
H	3.903216839	-4.563479363	0.040262793
H	4.864148409	-3.259699817	-0.740291232
H	3.608204857	-4.131634442	-1.679933932
H	0.557085237	-3.434770834	-1.217682237
H	0.106047021	-2.432861768	0.178059237
C	0.717904235	-4.517036878	0.721034975
H	3.066420558	-2.611093762	2.324996876
H	2.177142694	-1.089546380	1.956183543
H	3.926949687	-1.211734529	1.596540302
H	-1.883234586	3.975082380	0.946128021
H	-0.160552519	3.540721158	0.650873850
H	-1.212298298	2.463589631	1.638001789
H	-2.244549832	4.240554582	-2.178711271
H	-1.939736989	2.831019504	-3.248104845
H	-0.551513715	3.686004898	-2.486639724
H	-3.711867658	0.829786731	-1.485843401
C	-4.574112640	2.640560372	-0.611891124
H	-3.545667282	0.903026571	0.279996966

H	6.978192754	3.560729164	-1.840625933
H	7.189096240	1.909060516	-2.450811662
H	1.053629448	-4.289159163	1.753514515
H	1.379080759	-5.304092583	0.307996009
H	-4.477905586	3.249998247	-1.541321973
H	-4.391206963	3.340930213	0.231527280
B	-6.130475143	2.086190852	-0.612088534
B	7.888504783	2.229617916	-0.333439785
B	-0.815813493	-4.863629265	0.688560991
C	7.299037206	2.937215836	1.062487118
C	6.416397125	2.276589980	1.935385115
C	7.599698891	4.262952226	1.428808926
C	5.882993761	2.849028469	3.105528767
C	7.092830538	4.882824873	2.588720671
C	6.229425044	4.169265771	3.435240696
C	8.286797713	0.630089138	-0.007267290
C	7.785446985	-0.518566312	-0.639604412
C	9.279486130	0.375830543	0.959722516
C	8.224379986	-1.826818920	-0.351619837
C	9.758886797	-0.908513266	1.278065643
C	9.218004729	-2.024409440	0.618366868
C	-1.763905778	-4.062074473	1.678621816
C	-1.534694812	-4.036541470	3.067901714
C	-2.848079924	-3.292273090	1.211588481
C	-2.329105900	-3.292947918	3.956102004
C	-3.654516275	-2.515462156	2.061157424
C	-3.392069606	-2.524582018	3.444909569
C	-1.443124615	-5.912163281	-0.323837406
C	-2.669906696	-6.571057643	-0.054831650
C	-0.804944880	-6.306691431	-1.526371420
C	-3.228514206	-7.541798063	-0.904017535
C	-1.341797925	-7.257920822	-2.411549530
C	-2.560725517	-7.881360414	-2.093307773
C	-7.135929167	3.396347131	-0.915221196
C	-7.268723234	4.435792703	0.023315400
C	-7.868335827	3.582842360	-2.103266957
C	-8.066891069	5.578991803	-0.167277221
C	-8.682428299	4.710119035	-2.342079178
C	-8.783757540	5.714604056	-1.367209445
C	-6.680209764	1.377992802	0.809067558

C	-6.012291025	1.322681095	2.042902296
C	-7.955807821	0.779269883	0.808276649
C	-6.544121833	0.712866219	3.197147151
C	-8.530039221	0.151700871	1.929506861
C	-7.815511926	0.123153900	3.138309631
F	-6.602572900	4.376075674	1.204648776
F	-7.832173971	2.680425184	-3.104749310
F	-8.154585952	6.541463246	0.776967983
F	-9.368373831	4.839236638	-3.499610981
F	-9.557042454	6.800560493	-1.582028536
F	-8.346617497	-0.467194872	4.229347836
F	-5.855498822	0.696650048	4.357373127
F	-4.781159488	1.867632393	2.201391252
F	-8.718521203	0.794658818	-0.307464762
F	-9.759166044	-0.407017815	1.868573148
F	9.834574172	1.394473953	1.653879546
F	6.029231205	0.998768447	1.680907412
F	5.050918333	2.155398288	3.908668206
F	5.734691837	4.747575450	4.548499794
F	7.422157278	6.156556385	2.896935960
F	8.404214101	5.034823301	0.665627619
F	6.817106331	-0.438702871	-1.584708380
F	7.689765441	-2.895316831	-0.985778098
F	9.654614537	-3.267397653	0.908758651
F	10.715793148	-1.088131926	2.214875228
F	0.364863833	-5.760681107	-1.895969173
F	-0.703271473	-7.583564436	-3.543546372
F	-3.079208803	-8.800571406	-2.915052718
F	-4.383065381	-8.147871010	-0.592536287
F	-3.355867617	-6.317823257	1.072372499
F	-3.128094044	-3.272220315	-0.104091958
F	-0.539531247	-4.779818108	3.602458939
F	-2.093882147	-3.314249446	5.277141686
F	-4.153405979	-1.810256037	4.275468279
F	-4.657501079	-1.779433611	1.578117969
F	-6.259059235	1.101188533	-1.642552099
F	9.131930399	2.842403276	-0.716457549

9.42. $[(\{(C_6F_5)_2B\}FCH_2CH_2CH_2SiMe_2)_3P_7]^{3-}$

Charge = –3 Multiplicity = 1

P	0.260403868	-0.173408964	0.846774876
P	-0.399313775	1.700238585	-0.170439296
P	2.239641776	-0.421544783	-0.155586812
P	-0.908467159	-1.641026159	-0.359967640
P	1.672421181	-0.356692645	-2.320634307
P	-0.520279331	-0.937313844	-2.449551569
P	0.069151994	1.252585287	-2.313391352
Si	1.141319002	3.370755850	0.396247028
Si	2.904089087	-2.643143202	0.175126852
Si	-3.179276909	-1.174413779	-0.028078710
C	-3.811119725	0.249689274	-1.110979593
C	-3.346121431	-0.719376459	1.810876281
C	-4.100870474	-2.795501783	-0.408215428
C	2.120110759	-3.847544757	-1.064154450
C	2.360481671	-3.079921663	1.944126615
C	4.805143399	-2.660505005	0.035553720
C	0.228792878	5.032105113	0.194827746
C	2.714857657	3.324232740	-0.663042741
C	1.573426321	3.063921619	2.222441092
H	-0.125398414	5.116396588	-0.858352920
C	1.100505329	6.253813818	0.577498874
H	-0.691513451	4.986280740	0.817104278
H	0.662457371	3.065992490	2.855947668
H	2.241382251	3.874689222	2.585129818
H	2.094717051	2.093771745	2.355537955
H	2.479146558	3.473811351	-1.737455234
H	3.237997063	2.352365521	-0.551956991
H	3.399152522	4.140242780	-0.344651947
H	2.717253454	-4.101075673	2.199209645
H	1.255684149	-3.059249534	2.042227854
H	2.794624100	-2.374541043	2.682482926
H	5.201297712	-1.919569330	0.763838122
H	5.089989696	-2.274063317	-0.970111922
C	5.418423444	-4.062173964	0.276280117
H	2.475154672	-4.879318222	-0.852169780
H	2.406911741	-3.593129237	-2.105934439
H	1.013514389	-3.825490009	-0.990423168
H	-4.421352424	-0.590728268	2.061682944
H	-2.815201692	0.226375277	2.044117969
H	-2.939742647	-1.521480324	2.461260415

H	-4.886570823	0.431509808	-0.896375956
H	-3.713608350	0.004561707	-2.189174671
H	-3.243214184	1.181266635	-0.910277104
H	-3.884897702	-3.097123977	-1.457561396
C	-5.634937004	-2.714815593	-0.197896104
H	-3.657840566	-3.591203852	0.230967850
H	1.989457134	6.290528347	-0.095462448
H	1.515243886	6.117679161	1.602309307
H	5.027294326	-4.766997519	-0.494925697
H	5.063458394	-4.468887379	1.250562539
H	-6.038351099	-1.882727290	-0.822966059
H	-5.870865071	-2.442792441	0.853845235
B	-6.460420968	-4.060996926	-0.675409545
B	0.475874957	7.779952061	0.498852382
B	7.054170012	-4.277354928	0.220720877
C	-0.142795978	8.033329537	-1.034759940
C	-1.471227229	7.741958405	-1.391013478
C	0.656147562	8.503876063	-2.094501893
C	-2.003174255	7.929563699	-2.681162910
C	0.172470983	8.709632084	-3.402179135
C	-1.169084589	8.421162960	-3.697604934
C	-0.671251148	8.189696016	1.660367688
C	-1.032432264	7.429267674	2.784716018
C	-1.255550267	9.470707715	1.615490552
C	-1.907662101	7.884719843	3.793551548
C	-2.132112737	9.974149752	2.593511514
C	-2.462023488	9.169102507	3.695881018
C	7.626878123	-3.697732180	-1.240528560
C	7.660724299	-4.497260958	-2.399146298
C	8.053713338	-2.372296347	-1.434391587
C	8.118285557	-4.036574888	-3.650013368
C	8.519323805	-1.863058333	-2.661816375
C	8.552425906	-2.708294415	-3.782076520
C	7.947345430	-3.628780395	1.491329228
C	9.349753989	-3.760185071	1.472547865
C	7.435610604	-3.067685884	2.672934240
C	10.194330774	-3.368924764	2.527012106
C	8.237069815	-2.657157080	3.759394004
C	9.629046174	-2.809675150	3.684542003
C	-8.103662061	-3.705607147	-0.642539764

C	-8.773153621	-3.487378546	0.575468881
C	-8.911811660	-3.563745998	-1.787101363
C	-10.138786743	-3.164737616	0.683404006
C	-10.284501942	-3.242186670	-1.731809172
C	-10.903061938	-3.042655403	-0.488152783
C	-6.258185457	-5.447335308	0.259067624
C	-5.598459969	-5.547851151	1.495332179
C	-6.808138523	-6.657285488	-0.209120708
C	-5.464387569	-6.752093995	2.217078473
C	-6.699026725	-7.885639712	0.469454186
C	-6.020293084	-7.930878704	1.697904490
F	-8.101157413	-3.586221262	1.750284279
F	-8.413494124	-3.727520716	-3.029590996
F	-10.724660836	-2.973028415	1.886884724
F	-11.017734278	-3.122916997	-2.862354269
F	-12.216875377	-2.732723459	-0.418663997
F	-5.905659586	-9.096141347	2.370980262
F	-4.815363950	-6.788589815	3.400546634
F	-5.039729134	-4.465072678	2.084651507
F	-7.506578989	-6.691626075	-1.366259344
F	-7.249118487	-9.016222565	-0.030074212
F	-0.987300784	10.311496332	0.588537770
F	-2.339138905	7.261042491	-0.466013149
F	-3.294619192	7.647312196	-2.955754275
F	-1.649781634	8.611376093	-4.945275234
F	0.982403047	9.176616983	-4.379599984
F	1.964871983	8.788307829	-1.912743923
F	-0.548325814	6.178850093	2.978129858
F	-2.213983744	7.107258344	4.854325508
F	-3.298412398	9.629686849	4.651461393
F	-2.662717347	11.214720191	2.492007052
F	6.105274770	-2.882858755	2.849902305
F	7.685212294	-2.127184721	4.872089936
F	10.417104024	-2.429026027	4.713591210
F	11.536654167	-3.519554597	2.447077162
F	9.974446938	-4.292890508	0.395731821
F	8.049922048	-1.492915299	-0.401543787
F	7.242771112	-5.782415369	-2.376190208
F	8.141145326	-4.853340328	-4.727912689
F	8.993349355	-2.249149091	-4.973157885

F	8.933239476	-0.583276949	-2.778958612
F	-6.049588178	-4.403391561	-2.002984625
F	1.554840161	8.705813329	0.742240684
F	7.310813358	-5.694064253	0.310441299

9.43. [({$(C_6F_5)_2$B}$CH_2CH_2CH_2SiMe_2$)$_2$({$(C_6F_5)_2$B}H$CH_2CH_2CH_2SiMe_2$)P_7]$^-$

Charge = –1 Multiplicity = 1

P	0.935557118	-1.142378807	0.069037015
P	1.332188934	0.451166825	-1.438948253
P	0.539662711	-2.806037919	-1.360609499
P	-1.160856502	-0.574654736	0.549662478
P	-1.073752201	-1.959021410	-2.666958184
P	-2.051630651	-0.286636774	-1.483368815
P	-0.347659038	0.183910253	-2.898515548
Si	3.170455858	-0.234760084	-2.667538236
Si	-0.587420148	-4.424329967	-0.148965179
Si	-1.073863381	1.616181255	1.423426839
C	0.473618346	1.627092160	2.526879272
C	-2.626179573	1.658696906	2.519483131
C	-1.084411593	3.000654436	0.139315319
C	0.276806521	-4.584412413	1.533077006
C	-0.361468750	-6.026219816	-1.146919677
C	-2.451207560	-4.060285706	0.075899288
C	4.445604448	-0.855734101	-1.374300865
C	3.801314462	1.356098241	-3.487382589
C	2.865973412	-1.552437199	-3.995077369
H	4.523549150	-0.083327976	-0.577559613
C	5.855212431	-1.159603600	-1.991057663
H	4.016783182	-1.760613316	-0.892448318
H	2.528694927	-2.506399678	-3.541390255
H	3.798874791	-1.735970104	-4.571414264
H	2.085758186	-1.215107208	-4.708799516
H	4.024194929	2.139845969	-2.734338593
H	3.037292308	1.766793715	-4.180432765
H	4.722545228	1.162018003	-4.078179496
H	-0.813122421	-6.890327846	-0.613552773
H	0.715144353	-6.244758537	-1.304661954
H	-0.844705222	-5.949346132	-2.143251611
H	-2.893038167	-3.947468593	-0.937885165
H	-2.521859142	-3.061862468	0.561580021

C	-3.214114489	-5.150043768	0.905942169
H	-0.133334828	-5.446101108	2.102681364
H	0.138665348	-3.666738994	2.141002666
H	1.366702901	-4.749389972	1.404448717
H	-2.695963706	2.653128438	3.010009517
H	-2.595476318	0.881809114	3.311460385
H	-3.552669858	1.518589440	1.925178868
H	0.493079363	2.572964398	3.109637882
H	1.400434437	1.584453020	1.918559671
H	0.482112326	0.778889659	3.242427842
H	-0.227558741	2.862079821	-0.556453521
C	-1.026593862	4.405172401	0.791926993
H	-2.005743130	2.891348679	-0.475203738
H	6.255194759	-0.232953701	-2.452147760
H	5.762064324	-1.923947647	-2.788177954
H	-2.741949500	-5.245142359	1.905543260
H	-3.152566830	-6.134314536	0.400641374
H	-0.111073324	4.469325935	1.426328027
H	-1.887610856	4.539661121	1.483958641
B	-0.918415607	5.640632221	-0.304970008
B	6.764500767	-1.615676124	-0.786787010
B	-4.681587174	-4.586529244	1.037427549
C	7.328130154	-0.495801038	0.189522920
C	7.112244834	-0.551947323	1.581578222
C	8.037897289	0.626879086	-0.282783687
C	7.553426839	0.447549525	2.464490661
C	8.513060622	1.641374177	0.566708198
C	8.262223356	1.549133736	1.948446813
C	7.077870274	-3.137256986	-0.482513088
C	6.225277141	-4.194391853	-0.886385899
C	8.267471789	-3.540355703	0.174790456
C	6.509635616	-5.549732429	-0.645135653
C	8.598311659	-4.884706347	0.414285623
C	7.707990305	-5.894143314	0.005672032
C	-4.926148450	-3.433411515	2.100826318
C	-4.560708686	-3.568938556	3.454555189
C	-5.453536630	-2.182559188	1.721273724
C	-4.703986850	-2.530045874	4.389535880
C	-5.594737472	-1.110181430	2.617840380
C	-5.215625583	-1.290645546	3.961382288

C	-5.869130216	-5.060090282	0.105200568
C	-7.224144338	-4.968424093	0.513430406
C	-5.663979945	-5.645724758	-1.168774851
C	-8.298132160	-5.422365074	-0.270813271
C	-6.711996224	-6.092198377	-1.991723151
C	-8.037539300	-5.982060345	-1.534564105
C	-0.613639426	7.053269194	0.495712069
C	-1.588686295	7.717619268	1.262790052
C	0.653485626	7.667370819	0.496224020
C	-1.353670011	8.908339041	1.975916087
C	0.940585371	8.858491126	1.192718883
C	-0.072875799	9.484419015	1.936826160
C	-2.212911282	5.846529479	-1.314665885
C	-3.505301673	5.312748018	-1.161891922
C	-2.049711002	6.635858519	-2.472883375
C	-4.556149078	5.514218708	-2.079275992
C	-3.064622768	6.871568724	-3.418538301
C	-4.334378063	6.304908686	-3.217070521
F	-2.842471545	7.209931272	1.354435074
F	1.686949584	7.126557703	-0.186067499
F	-2.330489106	9.500701270	2.695909878
F	2.174042642	9.407906600	1.157566765
F	0.182624644	10.623431556	2.613077668
F	-5.325624911	6.516895769	-4.106950689
F	-5.773401893	4.967430409	-1.874361294
F	-3.821269218	4.542460897	-0.089112684
F	-0.858577291	7.221848925	-2.731647811
F	-2.840985811	7.634780432	-4.509913136
F	9.171268853	-2.629822138	0.576312363
F	6.439439460	-1.592364252	2.111751462
F	7.315134090	0.364216993	3.778857571
F	8.700953260	2.504807442	2.771516935
F	9.202864386	2.680670283	0.081397437
F	8.326828014	0.741294637	-1.595082720
F	5.063074524	-3.936819944	-1.507746783
F	5.660940456	-6.510291690	-1.029726332
F	8.001975837	-7.176577441	0.231775817
F	9.744415440	-5.214139838	1.023232094
F	-4.424269164	-5.779201940	-1.670694190
F	-6.464249459	-6.623243198	-3.195106781

F	-9.045679428	-6.413200101	-2.295934843
F	-9.559185887	-5.329736618	0.168860725
F	-7.545815577	-4.462186968	1.715519006
F	-5.821378320	-1.975427058	0.442998769
F	-4.082441035	-4.747121574	3.910320705
F	-4.361599983	-2.704306683	5.673510215
F	-5.341922882	-0.286718897	4.832392810
F	-6.071138710	0.071059571	2.211790940
H	0.048605362	5.405073572	-1.040216149

9.44. $[(\{(C_6F_5)_2B\}CH_2CH_2CH_2SiMe_2)(\{(C_6F_5)_2B\}HCH_2CH_2CH_2SiMe_2)_2P_7]^{2-}$

Charge = –2 Multiplicity = 1

P	-1.631634485	-1.466473508	-0.723018114
P	-1.794108453	-2.633747146	-2.618030741
P	-2.792102396	0.270248269	-1.480069966
P	0.420275838	-0.622251537	-0.926039241
P	-1.627336655	0.935529612	-3.270299068
P	0.447750344	0.041454559	-3.057979863
P	-1.170484781	-1.114813149	-4.148404831
Si	-4.119365377	-2.855319238	-2.940874494
Si	-2.610012780	1.942625646	0.087279512
Si	1.933356752	-2.414090978	-0.904845946
C	1.835411393	-3.482523138	-2.468971162
C	1.428943469	-3.430795983	0.619334722
C	3.662311474	-1.654474516	-0.700519762
C	-2.788721593	1.134340107	1.792520932
C	-4.089250948	3.080049714	-0.274876651
C	-0.985598839	2.961422034	-0.009461585
C	-5.080545607	-2.729125752	-1.308194196
C	-4.238494643	-4.606036967	-3.686004882
C	-4.751514370	-1.611129131	-4.230610971
H	-4.595710602	-3.385237886	-0.550062922
C	-6.581683839	-3.091358200	-1.459180112
H	-4.957922263	-1.690962335	-0.935688375
H	-4.696435893	-0.577657436	-3.832948466
H	-5.815548374	-1.834863684	-4.461510246
H	-4.170037920	-1.660716611	-5.174663623
H	-3.846193366	-5.367076423	-2.979260530
H	-3.662076260	-4.687312930	-4.631776296
H	-5.300072794	-4.854080748	-3.902052935

H	-4.243063401	3.815197406	0.544330187
H	-5.020324781	2.486557278	-0.383951043
H	-3.934750464	3.645138809	-1.218062134
H	-0.880408710	3.312064711	-1.058730759
H	-0.133928355	2.271610940	0.177847661
C	-0.983273391	4.165629810	1.000229161
H	-2.913133641	1.909069564	2.579915337
H	-1.891976229	0.528775621	2.038492683
H	-3.670537783	0.461986493	1.831032245
H	2.155521755	-4.259405574	0.761910440
H	0.415625252	-3.868757880	0.506676731
H	1.442501996	-2.809571352	1.538704673
H	2.533719047	-4.341205360	-2.367739189
H	2.137868720	-2.901610082	-3.365288131
H	0.808726434	-3.868700766	-2.634243584
H	3.847903936	-0.942044710	-1.536743823
C	4.787211873	-2.721508955	-0.655483286
H	3.664249670	-1.041496377	0.227840859
H	-6.652969995	-4.169549859	-1.733078626
H	-7.020366540	-2.544402130	-2.326645914
H	-1.016081719	3.765463185	2.033774216
H	-1.885871730	4.791410736	0.847757685
H	4.721616056	-3.344479934	-1.578708463
H	4.604965573	-3.422544395	0.190023131
B	6.322958711	-2.107813907	-0.628803116
B	-7.596172454	-2.873717689	-0.159593193
B	0.360326523	4.937922353	0.730545427
C	-6.890418123	-3.374842177	1.245171038
C	-6.043407282	-2.562775190	2.022057893
C	-7.074620808	-4.676388515	1.750713622
C	-5.431010772	-2.976272001	3.219957135
C	-6.483633275	-5.141915166	2.941431723
C	-5.656203579	-4.282824748	3.683754789
C	-8.249104523	-1.363672800	0.042413477
C	-7.801145610	-0.155203613	-0.520757676
C	-9.406913281	-1.229072312	0.838519626
C	-8.439785925	1.087102280	-0.336039634
C	-10.085268613	-0.014632803	1.052012393
C	-9.590979491	1.160702680	0.462448080
C	1.719378423	4.357409508	1.303740667

C	1.861862578	3.951703038	2.647700122
C	2.862222281	4.204268311	0.490015276
C	3.060736041	3.432347255	3.164869826
C	4.075237578	3.675323175	0.960631055
C	4.171686677	3.291102788	2.312187522
C	0.398964681	6.269201446	-0.138794936
C	1.329351724	7.302974814	0.127826449
C	-0.519732759	6.542135936	-1.180341745
C	1.355848427	8.518506332	-0.577038802
C	-0.511372198	7.735692417	-1.924229376
C	0.431358059	8.731287816	-1.614468171
C	7.404503343	-3.338552329	-0.862922450
C	7.691292594	-4.296632913	0.127488727
C	8.083824318	-3.532958418	-2.081061341
C	8.587697316	-5.368010315	-0.047766282
C	8.990325477	-4.588273142	-2.307302972
C	9.245216929	-5.511853074	-1.280685774
C	6.773598912	-1.250990040	0.714523247
C	6.114567652	-1.207011119	1.956726638
C	7.944968737	-0.466292551	0.657395886
C	6.552308628	-0.439186906	3.055033806
C	8.428090949	0.313510419	1.724102346
C	7.723890124	0.323830917	2.939278770
F	7.086160947	-4.225104385	1.338375360
F	7.893382686	-2.695598024	-3.124023650
F	8.824295004	-6.257458421	0.942210517
F	9.620571665	-4.726938527	-3.495637844
F	10.108906802	-6.531598430	-1.478648988
F	8.167717486	1.061077099	3.979358661
F	5.869884823	-0.438979639	4.220308726
F	4.983127833	-1.922683686	2.173015360
F	8.684360272	-0.431061742	-0.475619929
F	9.558437736	1.045388872	1.602221406
F	-9.940709771	-2.311025628	1.451779849
F	-5.774001768	-1.288778614	1.632731009
F	-4.633724975	-2.144987207	3.922328853
F	-5.081832127	-4.710330738	4.826768894
F	-6.702172101	-6.400044701	3.384252484
F	-7.853270257	-5.567576285	1.095421306
F	-6.685271133	-0.114661943	-1.290255939

F	-7.942314066	2.213552333	-0.894953247
F	-10.218582385	2.340353547	0.656554542
F	-11.196568300	0.041720407	1.820299737
F	-1.447898461	5.636600790	-1.529753450
F	-1.391617428	7.937522313	-2.914009488
F	0.445201453	9.880545546	-2.300447253
F	2.246475503	9.474275534	-0.271703539
F	2.230119248	7.176922095	1.119505813
F	2.811188639	4.566092964	-0.807085223
F	0.840864146	4.103634060	3.517265821
F	3.160825229	3.088044661	4.455162209
F	5.318052702	2.805905079	2.788866948
F	5.130860664	3.544765421	0.152156112
H	6.436796696	-1.322341093	-1.578760110
H	-8.579751878	-3.600956044	-0.355580572

9.45. $[(\{(C_6F_5)_2B\}HCH_2CH_2CH_2SiMe_2)_3P_7]^{3-}$

Charge = –3 Multiplicity = 1

P	0.250294148	-0.150330720	0.882688189
P	-0.442821773	1.709332982	-0.138289006
P	2.226074529	-0.377556077	-0.132431945
P	-0.905093024	-1.640964076	-0.308486600
P	1.642235890	-0.329701537	-2.293449064
P	-0.543114386	-0.942000306	-2.403958483
P	0.016132445	1.256673335	-2.282139372
Si	1.078860698	3.405405333	0.407594627
Si	2.924685586	-2.588646068	0.204077435
Si	-3.182002045	-1.218023567	0.033071933
C	-3.842045579	0.217507719	-1.017615682
C	-3.352216019	-0.805020108	1.881602392
C	-4.075392881	-2.848297284	-0.382993877
C	2.145399388	-3.806412566	-1.026182950
C	2.391168438	-3.025204027	1.976760384
C	4.824976904	-2.587180824	0.056797066
C	0.147700297	5.056179625	0.207835362
C	2.645208729	3.368496914	-0.663816885
C	1.531294112	3.108981157	2.231118858
H	-0.223162075	5.131575090	-0.840428168
C	1.014211050	6.292410366	0.569103551
H	-0.762730072	5.008921842	0.844231952

H	0.626102465	3.103440809	2.872873937
H	2.193387002	3.928383107	2.585202685
H	2.064574342	2.145165159	2.362920565
H	2.400130801	3.514646012	-1.736633337
H	3.176811530	2.400960667	-0.555004311
H	3.325771058	4.190282431	-0.352200816
H	2.759838850	-4.041592435	2.234157505
H	1.286577131	-3.016120080	2.078928661
H	2.820469836	-2.312744391	2.711187977
H	5.219281049	-1.840664282	0.780224503
H	5.103314596	-2.202966565	-0.951741480
C	5.455998416	-3.984242110	0.300905193
H	2.509802113	-4.834206311	-0.810570977
H	2.426143535	-3.554897239	-2.070341327
H	1.038949701	-3.793432034	-0.948044976
H	-4.428380305	-0.692326691	2.135682826
H	-2.829836612	0.140180126	2.135663808
H	-2.938221924	-1.617901266	2.513505709
H	-4.920583236	0.373180713	-0.797981098
H	-3.740954224	-0.001522289	-2.101133563
H	-3.291532671	1.155205767	-0.797747064
H	-3.830978382	-3.133730237	-1.432065922
C	-5.614163357	-2.797920959	-0.194544880
H	-3.627513159	-3.644890937	0.252612374
H	1.898269110	6.312448139	-0.110453950
H	1.440929810	6.161896259	1.589902604
H	5.044359985	-4.692862941	-0.455591662
H	5.110020489	-4.381386418	1.282574884
H	-6.021562739	-1.975415448	-0.829132673
H	-5.854072478	-2.507697496	0.853346480
B	-6.412044695	-4.172320653	-0.651506667
B	0.346562332	7.806560893	0.480845077
B	7.102259524	-4.158885080	0.226331546
C	-0.256421452	8.079107708	-1.030513906
C	-1.583120970	7.808156785	-1.413099881
C	0.557342147	8.584458645	-2.063016519
C	-2.091859795	8.036317218	-2.706544701
C	0.099755541	8.826316249	-3.372356568
C	-1.238954173	8.551007764	-3.696031775
C	-0.737890937	8.243521968	1.655147608

C	-1.107972665	7.491041974	2.786315858
C	-1.297951753	9.539198598	1.629830657
C	-1.951847237	7.966617484	3.812055654
C	-2.144689719	10.061790765	2.622926684
C	-2.480211797	9.262917273	3.728367950
C	7.668385886	-3.634777228	-1.232104926
C	7.709217203	-4.489563419	-2.350782075
C	8.114047324	-2.323994630	-1.483283152
C	8.172271320	-4.094988796	-3.620475509
C	8.590888466	-1.880091062	-2.732081657
C	8.619249668	-2.777270843	-3.811723130
C	8.003220462	-3.556266814	1.479684217
C	9.404439854	-3.728019794	1.466707616
C	7.518647034	-2.961275224	2.660256445
C	10.263516466	-3.355426601	2.515258095
C	8.335073389	-2.570336302	3.742427914
C	9.721972345	-2.764230164	3.668528407
C	-8.045615656	-3.896425479	-0.649515405
C	-8.802909734	-3.798197756	0.533170463
C	-8.785117578	-3.711114269	-1.834060753
C	-10.188300588	-3.548918796	0.565060552
C	-10.171761999	-3.459151226	-1.856337581
C	-10.879004853	-3.379563894	-0.645997963
C	-6.110013235	-5.549575263	0.220527511
C	-5.505941741	-5.635661712	1.488889583
C	-6.485642984	-6.796249286	-0.323087088
C	-5.267786600	-6.848730969	2.167316333
C	-6.273749195	-8.033647731	0.312328485
C	-5.658823853	-8.058923194	1.574742459
F	-8.203501774	-3.943445470	1.740072249
F	-8.186102373	-3.767756221	-3.043553909
F	-10.863713376	-3.468737954	1.734558560
F	-10.835964062	-3.295650829	-3.024005573
F	-12.209452291	-3.137255060	-0.646020808
F	-5.448163682	-9.233686050	2.209118563
F	-4.683080814	-6.863950900	3.385107756
F	-5.109827784	-4.522838529	2.150285372
F	-7.096330516	-6.857159598	-1.530098992
F	-6.658022367	-9.195812729	-0.265773299
F	-1.010395772	10.383480155	0.608300382

F	-2.464486390	7.308951236	-0.512972659
F	-3.380037539	7.768192843	-3.011644987
F	-1.699091323	8.777120757	-4.946318914
F	0.924615378	9.324885410	-4.322194043
F	1.858985885	8.878895089	-1.834340513
F	-0.654851855	6.226184151	2.967887103
F	-2.262440711	7.189681246	4.872817507
F	-3.291047844	9.741063155	4.699128643
F	-2.640282470	11.318560219	2.533195304
F	6.195053818	-2.727461521	2.837146789
F	7.800887266	-2.007371790	4.848343451
F	10.524980352	-2.397887015	4.692984858
F	11.599940061	-3.556712677	2.433595547
F	10.007487125	-4.308428936	0.399734749
F	8.115157749	-1.401501316	-0.490734311
F	7.299061760	-5.776123452	-2.251351368
F	8.200106943	-4.963826876	-4.657559812
F	9.069039693	-2.378083842	-5.021843800
F	9.018122153	-0.611060448	-2.909560128
H	-6.083926490	-4.440284407	-1.814281478
H	1.279524394	8.614862524	0.644337728
H	7.330798266	-5.381854121	0.278503042

9.46. ($\{C_8H_{14}\}BCH_2CH_2SiMe_2)_3As_7$ (**7**)

Charge = 0 Multiplicity = 1

As	0.838565474	-0.609104223	0.906929978
As	1.450993100	1.316823002	-0.493439029
As	0.580638705	-2.267193676	-0.887534336
As	-1.537663342	-0.068235209	1.218554766
As	-1.021727902	-1.162169761	-2.409548690
As	-2.249593870	0.541326968	-1.067005366
As	-0.199627195	1.189098707	-2.325960238
Si	3.480443059	0.571395407	-1.617769729
Si	-0.816067472	-3.989749819	0.121021262
Si	-1.484196967	2.109325227	2.312987897
C	-0.055038121	2.086738725	3.566319133
C	-3.141143037	2.173208674	3.247665935
C	-1.344050452	3.594920735	1.126329115
C	-0.216393313	-4.250986444	1.904225561
C	-0.434291983	-5.537910821	-0.915792438

C	-2.691520152	-3.629034538	0.076582855
C	4.530194156	-0.361157454	-0.323241529
C	4.332256634	2.197098095	-2.122446050
C	3.201049971	-0.485090867	-3.168578771
H	4.575240158	0.262266720	0.598079884
C	5.957230608	-0.740242749	-0.782289421
H	3.965450883	-1.274479033	-0.029190979
H	2.696797404	-1.441329334	-2.919225408
H	4.177217532	-0.716553997	-3.648331050
H	2.576369135	0.050242962	-3.913454292
H	4.537601076	2.840478007	-1.241645590
H	3.700940799	2.777665123	-2.827502925
H	5.298538021	1.989842444	-2.631674142
H	-0.968829789	-6.423097673	-0.508526851
H	0.652393748	-5.763434734	-0.909875538
H	-0.748830099	-5.406748101	-1.972438413
H	-2.968638641	-3.396439647	-0.976886660
H	-2.864010879	-2.690600336	0.650388097
C	-3.570959997	-4.797993193	0.635098240
H	-0.717114612	-5.137182776	2.350666497
H	-0.444021860	-3.370538098	2.540027776
H	0.878740548	-4.427301969	1.938725266
H	-3.215123080	3.103697814	3.851498881
H	-3.244219727	1.311231774	3.939165845
H	-4.003432490	2.152199147	2.548544873
H	-0.095856533	2.991922117	4.210447053
H	0.930146466	2.072418887	3.055822931
H	-0.112477808	1.198448518	4.229068213
H	-0.403132073	3.474613939	0.544464910
C	-1.380787297	4.978446599	1.815734377
H	-2.163675837	3.512527414	0.377181637
H	6.535321351	0.167823210	-1.088542002
H	5.930280934	-1.334599416	-1.729892699
H	-3.282478506	-5.011420744	1.688031788
H	-3.393348358	-5.718314806	0.036074300
H	-0.577814115	5.065078402	2.589404580
H	-2.312302173	5.101056247	2.425328337
B	-1.294187319	6.288365494	0.931664617
C	-1.243581516	7.734893095	1.582874199
H	-1.301224614	7.705702090	2.694707223

C	0.141320408	8.348931483	1.205048289
H	0.212687824	9.391468820	1.593550154
H	0.926940546	7.776505794	1.751167074
C	0.478411589	8.333868347	-0.302758427
H	1.559322710	8.562184940	-0.434807998
H	-0.054016247	9.161877015	-0.813310120
C	0.157221457	6.995558605	-1.005308167
H	0.237363113	7.124693888	-2.109613883
H	0.944774486	6.253655435	-0.737656617
C	-1.228984220	6.368429642	-0.647936594
H	-1.274196828	5.369846666	-1.134745044
C	-2.460495573	7.188836044	-1.142286195
H	-2.406539957	7.322841451	-2.247596390
H	-3.371668611	6.571724671	-0.965096417
C	-2.655901877	8.563590064	-0.464539765
H	-3.673268665	8.944683146	-0.704580827
H	-1.960526840	9.303310377	-0.910951567
C	-2.476248889	8.539927639	1.069743408
H	-3.388117965	8.090865762	1.527879379
H	-2.436757598	9.584923745	1.456239231
B	6.901184287	-1.538107285	0.206341306
C	6.551346108	-2.013254336	1.675969706
H	5.535566777	-1.700005503	2.002516731
C	6.571015520	-3.575591393	1.656596564
H	6.380229342	-3.966831496	2.682868945
H	5.704810097	-3.918976981	1.044684035
C	7.859596201	-4.213330455	1.090634734
H	7.675808920	-5.294064568	0.900738912
H	8.658019839	-4.189103350	1.859828468
C	8.368698529	-3.553087527	-0.210457141
H	9.391928834	-3.928744297	-0.444215319
H	7.728870687	-3.894809636	-1.057114969
C	8.363443798	-1.991629449	-0.210528549
H	8.638261049	-1.658946132	-1.237154092
C	9.372819344	-1.336559822	0.781249408
H	10.407541262	-1.685815943	0.557319906
H	9.382194195	-0.240016175	0.580000956
C	9.065245704	-1.560872221	2.278643256
H	9.687439772	-0.865535332	2.884635308
H	9.394552812	-2.576051829	2.580092230

C	7.579376209	-1.361956350	2.652193218
H	7.365079056	-0.268680600	2.689483095
H	7.403199503	-1.731864265	3.688937789
B	-5.046538301	-4.254070632	0.500792399
C	-5.821977242	-3.484097271	1.646982964
H	-5.177688490	-3.256644983	2.525561382
C	-6.410151299	-2.144737938	1.120986700
H	-7.047319601	-1.670350056	1.903500381
H	-5.557345490	-1.444173889	0.968429540
C	-7.209931325	-2.243127769	-0.197525727
H	-7.358387133	-1.219587429	-0.607381344
H	-8.233178987	-2.616715968	0.012757111
C	-6.545654489	-3.124634770	-1.278956248
H	-7.272880557	-3.305099198	-2.104639331
H	-5.710164841	-2.550327592	-1.740785982
C	-5.961142286	-4.473648238	-0.773663038
H	-5.416635884	-4.951967130	-1.618642654
C	-7.030759105	-5.505729012	-0.276838417
H	-7.756197400	-5.716901215	-1.096469426
H	-6.510923104	-6.473247960	-0.085197786
C	-7.795869607	-5.093546603	1.001862059
H	-8.347759937	-5.976468900	1.394168655
H	-8.580956392	-4.353639194	0.745836512
C	-6.891101291	-4.527628160	2.120545563
H	-6.352824257	-5.374337741	2.606809357
H	-7.522300051	-4.078991667	2.922433106

9.47. $[(\{C_8H_{14}\}BCH_2CH_2SiMe_2)_2(\{C_8H_{14}\}BFCH_2CH_2SiMe_2)As_7]^-$

Charge = –1 Multiplicity = 1

As	0.593066247	-0.772085000	0.944913607
As	2.119774515	0.571511556	-0.444107900
As	-0.486980943	-2.088382413	-0.818103137
As	-1.189052944	0.898454342	1.227708829
As	-1.244316919	-0.332532254	-2.375873707
As	-1.470121915	1.768495692	-1.067671097
As	0.650917465	1.287489612	-2.296225588
Si	3.451668025	-1.118327082	-1.566379326
Si	-2.637094341	-2.779318389	0.198743976
Si	-0.064702066	2.771956041	2.287495984
C	1.095676539	2.078255257	3.629760215

C	-1.478592075	3.734086705	3.126653253
C	0.894801014	3.938189968	1.115820485
C	-2.203779688	-3.243118133	1.994497542
C	-2.983230862	-4.381261887	-0.778128405
C	-4.073953619	-1.563578396	0.077961524
C	3.856315960	-2.494714852	-0.299944628
C	5.055019319	-0.203761682	-2.054060728
C	2.685286492	-1.861665671	-3.136602447
H	4.225994434	-2.009156260	0.631329662
C	4.862379507	-3.563691456	-0.782812820
H	2.892179709	-2.973098261	-0.015973095
H	1.750013526	-2.408337620	-2.895877330
H	3.398197077	-2.567500018	-3.617064030
H	2.438175613	-1.070041018	-3.874214904
H	5.564168278	0.223989595	-1.164987550
H	4.835788408	0.632010216	-2.751725664
H	5.766089404	-0.889723521	-2.564622585
H	-3.900952961	-4.863970086	-0.378800599
H	-2.144349268	-5.104382624	-0.702055689
H	-3.159352105	-4.165857790	-1.853216149
H	-4.208451097	-1.267464079	-0.988293028
H	-3.816469659	-0.626339526	0.620030643
C	-5.386442300	-2.146899982	0.658638135
H	-3.086565161	-3.729029988	2.463075254
H	-1.960710321	-2.340137987	2.592143247
H	-1.346246385	-3.945547278	2.051595429
H	-1.085135322	4.587227531	3.721641959
H	-2.054232473	3.076157112	3.810465630
H	-2.191066660	4.137875696	2.376871810
H	1.505706879	2.900395847	4.256464974
H	1.947532442	1.528818198	3.177747888
H	0.555016979	1.378515761	4.300634700
H	1.674686298	3.330473380	0.604778490
C	1.528971008	5.175007726	1.790987217
H	0.197502503	4.253991885	0.307335271
H	5.831010669	-3.095820550	-1.093537101
H	4.516495614	-4.036138988	-1.737330205
H	-5.208521415	-2.494025286	1.706459381
H	-5.686801401	-3.054518356	0.084977358
H	2.201655867	4.874833825	2.634278684

H	0.755355209	5.789080939	2.319262621
B	2.357542695	6.197490944	0.917687654
C	3.075470494	7.464003715	1.556840350
H	2.896505754	7.546089108	2.653193416
C	4.609310621	7.266830844	1.349373139
H	5.163794856	8.155110790	1.733981371
H	4.931612851	6.415878214	1.993657775
C	5.044771902	6.975738271	-0.104786022
H	6.098200358	6.616400195	-0.102669251
H	5.065109099	7.921489301	-0.684293563
C	4.159220261	5.937842982	-0.831010321
H	4.408341553	5.931111001	-1.917804376
H	4.424862144	4.920406417	-0.462312403
C	2.620053288	6.123672834	-0.643094209
H	2.121489911	5.252839390	-1.120670818
C	2.036405650	7.415394869	-1.293135494
H	2.263339694	7.426524366	-2.384726536
H	0.925717581	7.356171786	-1.224749114
C	2.500350140	8.745163267	-0.657032214
H	1.849368793	9.569953003	-1.023958596
H	3.517362253	8.999419494	-1.020270807
C	2.485869686	8.742353336	0.888329959
H	1.431108270	8.848074483	1.233561741
H	3.012515065	9.649993179	1.266799739
B	5.244440464	-4.764491029	0.169945740
C	4.688010232	-5.039669131	1.627510596
H	3.980267761	-4.255008408	1.972259181
C	3.894012484	-6.383059276	1.557990083
H	3.519072961	-6.652314503	2.572728565
H	2.980598205	-6.205715099	0.945087627
C	4.667992208	-7.579010760	0.958686927
H	3.950217140	-8.399336198	0.734973678
H	5.356442977	-7.999699264	1.720167400
C	5.457558377	-7.237429967	-0.325232419
H	6.138528480	-8.083062038	-0.580810396
H	4.740009998	-7.168959194	-1.175582986
C	6.262274145	-5.901228679	-0.275624603
H	6.677316699	-5.725183633	-1.294197590
C	7.457383655	-5.896946471	0.724815761
H	8.163984405	-6.724687800	0.480355362

H	8.034782287	-4.957602481	0.558617909
C	7.066820471	-5.977358076	2.217737611
H	7.955525981	-5.727187362	2.839402629
H	6.817681388	-7.025504163	2.482009894
C	5.896782638	-5.048432295	2.612851535
H	6.280800800	-4.004602385	2.687046789
H	5.547375103	-5.307121329	3.639584359
B	-6.608010725	-1.014154439	0.719045332
C	-7.951698548	-1.572498128	1.505048482
H	-7.670700243	-1.955501693	2.518122558
C	-8.909124498	-0.375719108	1.728517960
H	-9.847380103	-0.688795311	2.255809142
H	-8.389271352	0.330159201	2.413891700
C	-9.302009025	0.400044276	0.446350569
H	-9.772588413	1.370066367	0.736234316
H	-10.104319100	-0.152042489	-0.090600959
C	-8.126502198	0.677460583	-0.523856946
H	-8.546679412	1.064083958	-1.488069327
H	-7.515411063	1.504401721	-0.098670818
C	-7.166102484	-0.516376752	-0.755722586
H	-6.329707837	-0.129346372	-1.389542117
C	-7.798931691	-1.703363847	-1.522698448
H	-8.218521624	-1.379051204	-2.509754165
H	-6.984024181	-2.422161085	-1.774401802
C	-8.903573852	-2.472854101	-0.756257467
H	-9.111488916	-3.437322548	-1.278717314
H	-9.856227259	-1.904794516	-0.822423272
C	-8.582701724	-2.755848674	0.732595164
H	-7.872871670	-3.614669937	0.780722924
H	-9.518588890	-3.120588258	1.229896987
F	-6.056879447	0.137164992	1.451814415

9.48. $[(\{C_8H_{14}\}BCH_2CH_2SiMe_2)(\{C_8H_{14}\}BFCH_2CH_2SiMe_2)_2As_7]^{2-}$

Charge = –2 Multiplicity = 1

As	0.006256812	-1.046634063	1.177259615
As	1.629002595	0.034938406	-0.311542590
As	-1.315404627	-2.331741457	-0.449919314
As	-1.580795913	0.831045670	1.376488086
As	-1.889623576	-0.614580350	-2.119931209
As	-1.819692511	1.588641998	-0.966898360

As	0.194926734	0.750679161	-2.185103012
Si	3.028736766	-1.701814242	-1.328537240
Si	-3.492558752	-2.671881114	0.669476923
Si	-0.206121821	2.614739936	2.275576648
C	0.966451010	1.880131951	3.585007790
C	-1.445115745	3.772325222	3.155796616
C	0.783317782	3.625748485	0.989047863
C	-3.143926302	-2.803624325	2.538326709
C	-3.927756640	-4.417982242	0.025586721
C	-4.882643762	-1.445937568	0.277300850
C	4.326156157	-2.252004378	-0.059473793
C	3.845531640	-0.732301947	-2.757733947
C	2.064714082	-3.163164631	-2.064277082
H	4.744424781	-1.345458487	0.435821563
C	5.462910264	-3.104384586	-0.682891519
H	3.829753527	-2.830245846	0.752240578
H	1.514778326	-3.722077059	-1.280197306
H	2.783213202	-3.859996155	-2.548538290
H	1.326594414	-2.828914616	-2.821904621
H	4.388545726	0.160470016	-2.382338554
H	3.098480423	-0.395753616	-3.507215618
H	4.584644653	-1.387135545	-3.267937821
H	-4.867054385	-4.771400242	0.502824182
H	-3.121797872	-5.148630235	0.247992269
H	-4.090389870	-4.409720812	-1.073165831
H	-4.940171843	-1.315413301	-0.828405467
H	-4.612223620	-0.444375850	0.680176559
C	-6.254738873	-1.888307671	0.849288749
H	-4.071217609	-3.131125812	3.056617062
H	-2.850146515	-1.818927374	2.957250037
H	-2.339285810	-3.534809205	2.761826980
H	-0.920829554	4.609171733	3.668161094
H	-2.030269363	3.216975883	3.918480597
H	-2.168345482	4.208959599	2.434910965
H	1.490554137	2.686072955	4.144480722
H	1.732731703	1.231023764	3.112684255
H	0.406377877	1.265085353	4.320054632
H	1.440782963	2.905677333	0.452181372
C	1.614895947	4.798226671	1.554744470
H	0.064809245	3.991960075	0.221435933

H	5.976049739	-2.512598619	-1.477248943
H	5.018280236	-3.984758746	-1.210219806
H	-6.159076009	-2.056720309	1.950942656
H	-6.535101530	-2.879376996	0.420964480
H	2.297980504	4.454282394	2.371153203
H	0.961389562	5.534633467	2.092562977
B	2.479225993	5.678537452	0.570625830
C	3.505424337	6.775641906	1.095135444
H	3.456646566	6.909025054	2.200174070
C	4.921765608	6.204980918	0.761476107
H	5.707530333	6.938810642	1.060420163
H	5.088610565	5.312966092	1.408252454
C	5.133767731	5.788632302	-0.712510977
H	6.060593901	5.177331300	-0.785156006
H	5.331985833	6.688679616	-1.330893984
C	3.956099252	4.988653708	-1.315065192
H	4.092585161	4.895732866	-2.417602498
H	3.994572269	3.945542727	-0.926784698
C	2.532389203	5.560887326	-1.008005341
H	1.795981952	4.831105920	-1.407352593
C	2.233111671	6.944716472	-1.652988300
H	2.361175423	6.887299788	-2.759452419
H	1.152079139	7.164075576	-1.493133611
C	3.061947724	8.127575899	-1.103001262
H	2.603869227	9.083689754	-1.443755590
H	4.074842129	8.116648441	-1.555920688
C	3.185026351	8.148013306	0.437176806
H	2.221415219	8.511168972	0.865276887
H	3.946368042	8.907136168	0.737073014
B	6.546346388	-3.697581873	0.429107336
C	7.424423374	-2.549453714	1.238071987
H	6.745874734	-1.817451583	1.742466315
C	8.219626493	-3.256331588	2.364369299
H	8.842668726	-2.531890422	2.951366570
H	7.470270232	-3.668381063	3.076250332
C	9.131209289	-4.420862776	1.901048095
H	9.451215683	-5.011482531	2.793515320
H	10.076550418	-4.009762413	1.482478141
C	8.480325823	-5.374163165	0.867957265
H	9.274886786	-6.055666096	0.464695955

H	7.756583329	-6.025084202	1.407228347
C	7.694118400	-4.672397169	-0.265843472
H	7.197769720	-5.478576769	-0.862287152
C	8.577841178	-3.864524277	-1.245981324
H	9.388465157	-4.499116576	-1.691467011
H	7.940364260	-3.557694618	-2.107586631
C	9.232574725	-2.592693962	-0.650270239
H	9.624843236	-1.956651029	-1.480235069
H	10.133361300	-2.884247374	-0.067999851
C	8.299946074	-1.743270120	0.249326057
H	7.618929457	-1.156228164	-0.410184207
H	8.927035349	-0.979522323	0.779171641
B	-7.463199948	-0.766765980	0.630623152
C	-8.889856943	-1.220176573	1.344205549
H	-8.716414972	-1.460487482	2.423329661
C	-9.852807927	-0.008212043	1.305012655
H	-10.842755576	-0.247012213	1.775795909
H	-9.393920657	0.787866685	1.932731709
C	-10.110559476	0.581164516	-0.104629573
H	-10.600680004	1.579458667	0.000421401
H	-10.860048665	-0.045223518	-0.637267671
C	-8.841633073	0.728409149	-0.981699744
H	-9.161666826	0.976176854	-2.027448711
H	-8.269796994	1.609776962	-0.615458689
C	-7.872070866	-0.479022216	-0.948219726
H	-6.972488593	-0.177661466	-1.539559946
C	-8.431689301	-1.763851283	-1.605011974
H	-8.745518133	-1.582988580	-2.666071838
H	-7.600974896	-2.504847464	-1.668291318
C	-9.615953323	-2.427172531	-0.858047997
H	-9.776544331	-3.456432530	-1.260800341
H	-10.552693893	-1.879970949	-1.100364626
C	-9.452511321	-2.499811824	0.681025597
H	-8.760559309	-3.341040436	0.919154640
H	-10.439492032	-2.796406062	1.124208834
F	-6.994352134	0.483488122	1.248389996
F	5.767951420	-4.484753002	1.398864299

9.49. $[(\{C_8H_{14}\}BFCH_2CH_2SiMe_2)_3As_7]^{3-}$

Charge = –3 Multiplicity = 1

As	0.058874705	-0.102112771	1.292398948
As	-0.342917851	2.096441486	0.254382077
As	2.005425566	-0.806816453	-0.045277851
As	-1.686823327	-1.398966224	0.132860340
As	1.130325996	-0.568557530	-2.339832646
As	-1.359741489	-0.675083418	-2.202671475
As	-0.200398991	1.533999198	-2.143499266
Si	1.727953100	3.377760130	0.602332368
Si	2.125498482	-3.249952161	0.218254659
Si	-3.814214412	-0.310746151	0.714956495
C	-4.170640039	1.229483774	-0.341435406
C	-3.645293715	0.207756208	2.541673094
C	-5.180577041	-1.619719615	0.507807289
C	0.961093736	-4.175996561	-0.965597119
C	1.601144673	-3.603853332	2.017161468
C	3.939630902	-3.748306454	-0.071078484
C	1.249493818	5.219364421	0.552352090
C	3.065340200	2.978612028	-0.689269459
C	2.361321349	2.889711753	2.332803971
H	0.733574528	5.427377229	-0.413986774
C	2.442239371	6.192648760	0.753490818
H	0.481858877	5.405182848	1.338051376
H	1.574054624	3.027069509	3.103345356
H	3.221894299	3.538238988	2.606445035
H	2.694852328	1.831494132	2.357316615
H	2.717936199	3.226228859	-1.714318959
H	3.339684842	1.903667459	-0.664511415
H	3.972061556	3.586711894	-0.478193849
H	1.750446895	-4.682749475	2.239763365
H	0.532151262	-3.355618824	2.182293960
H	2.210563259	-3.019582720	2.738109891
H	4.582061559	-3.118389489	0.587435981
H	4.230139088	-3.471547632	-1.110247164
C	4.232661411	-5.255395909	0.159010064
H	1.063543219	-5.270777944	-0.800309066
H	1.219047187	-3.964977423	-2.024373138
H	-0.096247553	-3.884502353	-0.797825637
H	-4.624918360	0.581772980	2.911090520
H	-2.890958860	1.012286412	2.665310717
H	-3.348136036	-0.651907762	3.178084440

H	-5.138437265	1.675555207	-0.024293670
H	-4.250746806	0.966622379	-1.417023688
H	-3.369849903	1.989237558	-0.227258603
H	-5.142347096	-2.013722176	-0.534599903
C	-6.607001214	-1.108251658	0.845413726
H	-4.935839141	-2.490686697	1.157753957
H	3.168929245	6.058304680	-0.082611506
H	3.004965013	5.900374270	1.675628255
H	3.541215012	-5.863106856	-0.476249760
H	3.970770712	-5.524846440	1.210045009
H	-6.879199407	-0.289455017	0.137995579
H	-6.595104833	-0.626226346	1.855326519
B	-7.767805644	-2.293683855	0.895282030
C	-9.255146516	-1.715057474	1.354850240
H	-9.162367298	-1.154168246	2.318859759
C	-9.803465800	-0.720109306	0.304893689
H	-10.826129250	-0.347597823	0.579445235
H	-9.149630002	0.182339484	0.317132684
C	-9.858455571	-1.257601877	-1.147245280
H	-10.016886900	-0.404200799	-1.850204755
H	-10.762061867	-1.894307557	-1.267536439
C	-8.607874792	-2.059043953	-1.588382876
H	-8.841943594	-2.562039163	-2.563217540
H	-7.793881911	-1.334115282	-1.820592854
C	-8.063513436	-3.061600859	-0.543386600
H	-7.117133321	-3.484716244	-0.961373544
C	-8.997795714	-4.265613056	-0.265855154
H	-9.239533830	-4.831850525	-1.203807094
H	-8.430269942	-4.968700556	0.383688503
C	-10.327827007	-3.920053852	0.450668847
H	-10.799861580	-4.862336749	0.822950734
H	-11.054047955	-3.514999381	-0.288990327
C	-10.182114082	-2.924401381	1.629054301
H	-9.746241525	-3.474852518	2.492667386
H	-11.208880032	-2.601511039	1.948312196
B	2.005665008	7.782812846	0.943619409
C	1.259147588	8.476684608	-0.363541291
H	0.364033040	7.878706590	-0.664239343
C	0.733100020	9.869660952	0.062921677
H	0.214818041	10.397068738	-0.780897505

H	-0.041637438	9.694746122	0.842272858
C	1.798818665	10.826778815	0.654106864
H	1.284502957	11.684508566	1.153211774
H	2.381163463	11.291019560	-0.172831043
C	2.771798209	10.162514257	1.660741763
H	3.604588073	10.883832484	1.876605498
H	2.228092869	10.018588205	2.621143822
C	3.306463751	8.773208656	1.236039433
H	3.898220919	8.376768746	2.099254852
C	4.255832930	8.804815725	0.015083410
H	5.120215189	9.501884987	0.178998522
H	4.710944747	7.792784662	-0.088843971
C	3.586423572	9.174815399	-1.332527004
H	4.279858587	8.920245846	-2.170360510
H	3.466805748	10.278906634	-1.390458530
C	2.214041462	8.499645873	-1.580533775
H	2.396122542	7.444416167	-1.889816995
H	1.739869388	8.990343363	-2.470753860
B	5.779517330	-5.721168553	-0.224566048
C	6.010841555	-7.358418355	-0.069770515
H	5.232778053	-7.907776043	-0.657281419
C	7.380376776	-7.716272988	-0.696560590
H	7.598513322	-8.815400275	-0.627165879
H	7.308875987	-7.481065607	-1.782046122
C	8.590011990	-6.940766693	-0.116594823
H	9.472237990	-7.081126883	-0.788381373
H	8.896337565	-7.400251192	0.849381921
C	8.342402335	-5.424894753	0.086851902
H	9.199227510	-5.003604304	0.676063587
H	8.380196598	-4.933664757	-0.910899797
C	6.974575139	-5.057336438	0.713605495
H	6.909351133	-3.941574887	0.697868997
C	6.809799398	-5.496012577	2.188181517
H	7.625910892	-5.083052341	2.837487939
H	5.869076399	-5.040750732	2.575176173
C	6.738144142	-7.026458005	2.420072179
H	6.369609019	-7.226054297	3.455486277
H	7.767166557	-7.447276899	2.400924381
C	5.853991249	-7.795517969	1.405893064
H	4.787084506	-7.648615348	1.692704741

H	6.046300188	-8.893679243	1.536659573
F	6.006043981	-5.342689607	-1.630964393
F	1.095662256	7.841346041	2.102059486
F	-7.320158575	-3.288824945	1.887198949

9.50. [({C_8H_{14}}$BCH_2CH_2SiMe_2$)$_2$({C_8H_{14}}$BHCH_2CH_2SiMe_2$)As_7]$^-$

Charge = –1 Multiplicity = 1

As	0.420628537	-0.815168140	0.940008070
As	2.085707359	0.330592918	-0.467641501
As	-0.871067710	-1.935185668	-0.814600270
As	-1.095979307	1.088632094	1.296910676
As	-1.420358595	-0.061952117	-2.319638872
As	-1.309978862	2.027987498	-0.978745385
As	0.687694993	1.271277579	-2.273877798
Si	3.150990946	-1.515399549	-1.627425282
Si	-3.066585259	-2.375355309	0.245080629
Si	0.327069099	2.754552730	2.346913027
C	1.437914696	1.868437303	3.615583186
C	-0.896012533	3.880622378	3.278444948
C	1.384014977	3.809409048	1.154636388
C	-2.648099153	-2.900920511	2.027621503
C	-3.610736959	-3.919271844	-0.734247784
C	-4.360393078	-1.002271806	0.169586066
C	3.396227913	-2.930966761	-0.363659642
C	4.850736877	-0.823885796	-2.153905119
C	2.252818644	-2.144119303	-3.177323345
H	3.858865774	-2.495947290	0.551007319
C	4.225591386	-4.135781747	-0.862409693
H	2.383865649	-3.267033505	-0.045390294
H	1.256391680	-2.555263816	-2.913299835
H	2.849507591	-2.941797141	-3.672174385
H	2.101064183	-1.325093360	-3.910828731
H	5.434892393	-0.472469049	-1.277594299
H	4.730110134	0.036241567	-2.845956463
H	5.449470009	-1.597985141	-2.682185629
H	-4.565304167	-4.302049667	-0.314067496
H	-2.853852957	-4.730039722	-0.686429259
H	-3.790598914	-3.674064850	-1.802269509
H	-4.483918436	-0.675482312	-0.888928792
H	-3.984659848	-0.107374680	0.715732476

C	-5.715738665	-1.462115839	0.765710129
H	-3.572691110	-3.276854865	2.516257351
H	-2.279567205	-2.039192419	2.622143734
H	-1.883062096	-3.704450461	2.060307533
H	-0.358122839	4.657518854	3.864788580
H	-1.519551960	3.291539815	3.982828207
H	-1.583991149	4.396412379	2.575906512
H	1.980581091	2.607696084	4.244583844
H	2.188289929	1.225225807	3.110603508
H	0.836347259	1.225637560	4.291544176
H	2.028896249	3.110877781	0.576105423
C	2.241319930	4.906300869	1.825207369
H	0.697177225	4.260220502	0.403163314
H	5.237820970	-3.811873755	-1.215857925
H	3.781938553	-4.569116328	-1.794364363
H	-5.553799738	-1.824958063	1.811122121
H	-6.079483041	-2.353237155	0.196898747
H	2.914800010	4.470547556	2.606639179
H	1.607693474	5.605242328	2.428832036
B	3.161670880	5.826772647	0.930354549
C	4.090566266	6.954743005	1.557164651
H	3.994060936	7.017955767	2.665089309
C	5.566605139	6.562064805	1.239837248
H	6.260474466	7.352109398	1.612350821
H	5.809783887	5.651135172	1.834948041
C	5.865627645	6.271883210	-0.248450215
H	6.857965217	5.774189626	-0.328340262
H	5.979013078	7.228066546	-0.799305110
C	4.802520611	5.391580082	-0.943773537
H	4.978852430	5.394648728	-2.044717807
H	4.950230857	4.334000940	-0.625411810
C	3.318420505	5.773336593	-0.645264016
H	2.676241238	4.996712347	-1.113730913
C	2.874709081	7.156288309	-1.214532839
H	3.029719216	7.178976361	-2.318436846
H	1.773053305	7.243722467	-1.070959706
C	3.555613518	8.386372043	-0.573006413
H	3.001399639	9.304576011	-0.870355141
H	4.572632012	8.515199609	-0.997113249
C	3.639166107	8.326055045	0.968940577

H	2.632789637	8.558885626	1.388383472
H	4.307850746	9.139259080	1.337615980
B	4.481284123	-5.359168399	0.104089445
C	3.980432203	-5.513801823	1.599224860
H	3.402291003	-4.632445361	1.951961143
C	3.020261770	-6.744586505	1.634716329
H	2.681012904	-6.928570216	2.680583503
H	2.098773242	-6.471726205	1.070987425
C	3.595391295	-8.050278184	1.040837530
H	2.766101076	-8.777683061	0.894848206
H	4.272342387	-8.530783297	1.776817311
C	4.337437708	-7.857535690	-0.300931581
H	4.886443413	-8.792856425	-0.561396095
H	3.581070785	-7.724576943	-1.108926909
C	5.307652094	-6.637060704	-0.355027552
H	5.676765502	-6.551111735	-1.402441870
C	6.555215187	-6.754311354	0.572502377
H	7.132552276	-7.675286516	0.322159305
H	7.236644721	-5.904864550	0.332762905
C	6.253685916	-6.731256794	2.087945355
H	7.204944358	-6.577609069	2.645034799
H	5.889694415	-7.728062104	2.410969002
C	5.239818773	-5.644473889	2.511018845
H	5.757948700	-4.657486346	2.517139220
H	4.926647671	-5.818954741	3.566811782
B	-6.860484101	-0.252693198	0.819117300
C	-8.244999060	-0.725912010	1.599170540
H	-8.005746543	-1.123923814	2.617238494
C	-9.140269174	0.519195868	1.811128619
H	-10.103678372	0.259413432	2.324109022
H	-8.596346030	1.199220455	2.506417035
C	-9.478097676	1.312050993	0.523975315
H	-9.894674790	2.309075425	0.805542226
H	-10.308614274	0.803516716	-0.013075512
C	-8.288244907	1.514141377	-0.447572797
H	-8.692732914	1.909311769	-1.416199537
H	-7.636846234	2.319237898	-0.036422979
C	-7.389998383	0.270214621	-0.661802429
H	-6.538020895	0.609267935	-1.302662669
C	-8.086290654	-0.881884268	-1.428867299

H	-8.482141801	-0.541882080	-2.421038345
H	-7.311319728	-1.646951192	-1.671323990
C	-9.236481006	-1.584816083	-0.665115693
H	-9.492898287	-2.540669546	-1.182502465
H	-10.157049919	-0.966778835	-0.743716131
C	-8.943793757	-1.874109115	0.828628632
H	-8.286396438	-2.773194379	0.887637689
H	-9.904224535	-2.180212515	1.319911381
H	-6.333609831	0.712454450	1.442242942

9.51. $[(\{C_8H_{14}\}BCH_2CH_2SiMe_2)(\{C_8H_{14}\}BHCH_2CH_2SiMe_2)_2As_7]^{2-}$

Charge = –2 Multiplicity = 1

As	-0.012202701	-1.061183040	1.136266312
As	1.644062472	-0.000663043	-0.329481766
As	-1.351717744	-2.297157946	-0.513734857
As	-1.552180019	0.854149713	1.348197805
As	-1.875957589	-0.547779332	-2.165751439
As	-1.759883565	1.641206904	-0.988450580
As	0.241230113	0.767908495	-2.204943688
Si	3.007391936	-1.771586092	-1.335313726
Si	-3.538337579	-2.610525388	0.596368779
Si	-0.137116037	2.592466786	2.272753757
C	1.013858661	1.816395129	3.577359036
C	-1.351704419	3.768716355	3.163057251
C	0.879956883	3.596627057	1.002415060
C	-3.171403572	-2.817494839	2.455223777
C	-4.025641377	-4.320864675	-0.103446068
C	-4.898233668	-1.332741003	0.262753943
C	4.157927688	-2.474087475	-0.000717949
C	3.992266586	-0.783067619	-2.640305457
C	2.009986867	-3.116845054	-2.231303075
H	4.631607076	-1.619640746	0.536214624
C	5.244747201	-3.430107671	-0.562439256
H	3.551629999	-3.001396205	0.771150960
H	1.356564679	-3.673640661	-1.529258060
H	2.716892000	-3.837478695	-2.697395205
H	1.364901110	-2.687083488	-3.025171075
H	4.564887223	0.044595784	-2.171578988
H	3.328339144	-0.351337690	-3.418800439
H	4.721664365	-1.459227034	-3.136494421

H	-4.980148888	-4.655287662	0.357041703
H	-3.246812092	-5.085277018	0.101483033
H	-4.180612611	-4.273473487	-1.202269294
H	-5.020095409	-1.228557788	-0.840783193
H	-4.559004037	-0.332635051	0.615443385
C	-6.248204328	-1.706597374	0.931642355
H	-4.097688727	-3.150289151	2.971619263
H	-2.856989787	-1.854292596	2.907988525
H	-2.375451069	-3.568474605	2.641173366
H	-0.810078545	4.587319295	3.686735602
H	-1.951908643	3.218268827	3.917595903
H	-2.062153647	4.230469813	2.445060182
H	1.555901098	2.603722148	4.146197410
H	1.765371493	1.153882840	3.099973592
H	0.437421547	1.207411844	4.304754176
H	1.523411303	2.868633290	0.459317922
C	1.734945219	4.743297216	1.585713875
H	0.173007236	3.988256756	0.236587229
H	5.815103495	-2.895260436	-1.362796197
H	4.742440610	-4.281104779	-1.086469828
H	-6.089981124	-1.798348232	2.035188489
H	-6.546799523	-2.731558264	0.596577671
H	2.409720672	4.373017310	2.397791758
H	1.097130411	5.485493115	2.133969234
B	2.623073726	5.615791530	0.615991417
C	3.655639374	6.698877603	1.157285524
H	3.599008693	6.824233607	2.262889184
C	5.070905236	6.122956698	0.829795843
H	5.858736646	6.849572360	1.140745316
H	5.227509565	5.224658929	1.470452281
C	5.291882030	5.717570783	-0.645873601
H	6.215894829	5.101731303	-0.716603194
H	5.499883614	6.621619047	-1.255137451
C	4.114403383	4.929438319	-1.264176645
H	4.258908291	4.845332898	-2.366409197
H	4.144126443	3.882789645	-0.884755605
C	2.691879543	5.506525000	-0.962721575
H	1.954131309	4.784531950	-1.373518169
C	2.404688197	6.897421573	-1.598708960
H	2.540297487	6.847913866	-2.704647547

H	1.323784382	7.121617992	-1.444840853
C	3.236248878	8.071097869	-1.033341083
H	2.786143034	9.032508894	-1.369752417
H	4.252374661	8.057920615	-1.478930515
C	3.348018405	8.078565195	0.507839223
H	2.383388246	8.443967325	0.931700824
H	4.111548763	8.830667462	0.819605435
B	6.256725299	-4.054748978	0.599760884
C	7.256640319	-2.943884295	1.322576662
H	6.670089990	-2.097356180	1.759192354
C	7.974709239	-3.628699664	2.511212328
H	8.679195290	-2.930098408	3.036274843
H	7.190171687	-3.891496759	3.257492265
C	8.755400836	-4.919496560	2.156982581
H	9.008649721	-5.465540593	3.098170706
H	9.740608433	-4.644107401	1.719902832
C	8.019437470	-5.882356525	1.191346049
H	8.753647513	-6.659919362	0.848990848
H	7.241509395	-6.429365725	1.772124917
C	7.301265148	-5.199238323	0.002547501
H	6.735423060	-6.002632270	-0.532588871
C	8.266357765	-4.573155988	-1.034709276
H	9.004669303	-5.323055544	-1.424724424
H	7.661221303	-4.269687944	-1.920799609
C	9.054652687	-3.334476468	-0.539432164
H	9.511097063	-2.813019158	-1.415671730
H	9.921533446	-3.672818478	0.069424831
C	8.220559644	-2.319787761	0.282633303
H	7.608199700	-1.717364242	-0.428594770
H	8.927934897	-1.588067740	0.754513394
B	-7.465224369	-0.601058051	0.676562723
C	-8.826642011	-0.923683428	1.571623218
H	-8.567090572	-1.018888364	2.656002468
C	-9.792597769	0.279931560	1.453094055
H	-10.743178176	0.115295567	2.027993743
H	-9.289907535	1.150504054	1.933874912
C	-10.170725606	0.679850093	0.004627050
H	-10.644589650	1.691605740	0.014570371
H	-10.969759968	0.000476399	-0.366285016
C	-8.991773412	0.679005013	-1.000918864

H	-9.417477458	0.777878708	-2.034816330
H	-8.387573699	1.599109981	-0.828592118
C	-8.025127977	-0.525163474	-0.884120216
H	-7.191209721	-0.326506874	-1.602155195
C	-8.654502292	-1.876843570	-1.304418855
H	-9.068864694	-1.838965290	-2.346171004
H	-7.836710237	-2.634037797	-1.342930110
C	-9.763601078	-2.412884539	-0.364318160
H	-9.963339719	-3.485779510	-0.603308457
H	-10.719116540	-1.891434711	-0.591806736
C	-9.455827727	-2.274179763	1.147983958
H	-8.744564238	-3.083887095	1.434457650
H	-10.397821176	-2.493950757	1.717313071
H	-7.011903885	0.530321298	1.010254265
H	5.522206798	-4.585495867	1.481419717

9.52. $[(\{C_8H_{14}\}BHCH_2CH_2SiMe_2)_3As_7]^{3-}$

Charge = –3 Multiplicity = 1

As	-0.060298467	-0.117831905	-1.368142982
As	0.342106571	2.086710732	-0.342799124
As	-1.986855011	-0.824366553	-0.002878329
As	1.706538445	-1.399857702	-0.224837674
As	-1.083828472	-0.575394881	2.279162771
As	1.404869434	-0.669748550	2.111391156
As	0.233322069	1.533434158	2.058520846
Si	-1.735632922	3.361353650	-0.674006971
Si	-2.118465700	-3.268183211	-0.246392719
Si	3.826689305	-0.316893134	-0.838122193
C	4.185902696	1.249124489	0.178574703
C	3.645323651	0.158274937	-2.676256548
C	5.192783932	-1.623269383	-0.607005749
C	-0.880689232	-4.193052399	0.861263374
C	-1.709195793	-3.622994318	-2.075164936
C	-3.911018489	-3.766075390	0.161350500
C	-1.261763094	5.204829398	-0.613773561
C	-3.072058333	2.946513793	0.613340948
C	-2.365595212	2.877736054	-2.407764225
H	-0.796380226	5.416844909	0.377309364
C	-2.439238560	6.182673716	-0.880076318
H	-0.448021775	5.384498367	-1.354552419

H	-1.581073557	3.029111879	-3.178589693
H	-3.234198390	3.517922514	-2.675496718
H	-2.686467684	1.815794983	-2.439253790
H	-2.725205676	3.186983259	1.640167801
H	-3.342987792	1.870844372	0.579755190
H	-3.979900655	3.554449809	0.407409382
H	-1.874685415	-4.701963138	-2.286066354
H	-0.652917940	-3.375509793	-2.309224367
H	-2.363763824	-3.039140359	-2.755851590
H	-4.593981118	-3.155969043	-0.474897869
H	-4.141878710	-3.454709783	1.206864590
C	-4.211995706	-5.280477974	-0.010331551
H	-0.993485718	-5.287638515	0.702582691
H	-1.071853762	-3.981802740	1.934263344
H	0.164120008	-3.900213102	0.628915651
H	4.621244812	0.529562114	-3.058101701
H	2.885211016	0.954875205	-2.815393074
H	3.350342498	-0.717480041	-3.291582609
H	5.153850124	1.685715971	-0.151110150
H	4.268769482	1.011909188	1.259922753
H	3.385362295	2.006593846	0.048518293
H	5.183329937	-1.957764000	0.456829365
C	6.615465827	-1.149235166	-1.012779007
H	4.916237093	-2.530560642	-1.193130382
H	-3.227263305	6.007094292	-0.105248542
H	-2.928016153	5.904297114	-1.847743758
H	-3.492627328	-5.863222852	0.617968619
H	-3.968893742	-5.573593930	-1.062987249
H	6.881872863	-0.257613186	-0.391280286
H	6.581541173	-0.761533502	-2.062247703
B	7.783196923	-2.329900317	-0.942953751
C	9.259567898	-1.825753300	-1.519671057
H	9.151812117	-1.405083878	-2.551219806
C	9.832774388	-0.689357594	-0.637387599
H	10.845814485	-0.355191708	-0.988830598
H	9.171012784	0.199241104	-0.760318331
C	9.930824688	-1.013358223	0.874920137
H	10.100385408	-0.067028944	1.444101725
H	10.844253624	-1.620900950	1.060233405
C	8.700281998	-1.750172602	1.462184627

H	8.968194544	-2.109198299	2.491093951
H	7.888697774	-1.001665528	1.615201060
C	8.128047131	-2.889094664	0.582686240
H	7.201746593	-3.250159762	1.093872600
C	9.062335917	-4.117390980	0.461365162
H	9.343152344	-4.535617815	1.465189497
H	8.482549638	-4.917853221	-0.053287278
C	10.367921338	-3.877890993	-0.338361675
H	10.834420876	-4.863073745	-0.586340404
H	11.112090233	-3.373007229	0.316735971
C	10.193130547	-3.054440969	-1.639557755
H	9.755014278	-3.722752030	-2.416537227
H	11.215487657	-2.774073242	-2.012823189
B	-2.002255466	7.783437223	-0.973220018
C	-1.458434660	8.450263528	0.447726218
H	-0.609157749	7.856884749	0.867672742
C	-0.895112810	9.861022927	0.149087130
H	-0.521027714	10.371677385	1.076845353
H	-0.004253438	9.723972952	-0.506150625
C	-1.874862224	10.826313986	-0.565038252
H	-1.305775231	11.703841792	-0.959731776
H	-2.573625827	11.259878855	0.184366576
C	-2.693845050	10.189235168	-1.716210101
H	-3.500532268	10.914241866	-2.010837218
H	-2.027317480	10.093705921	-2.604399781
C	-3.257947510	8.779092895	-1.418759575
H	-3.724593805	8.415198498	-2.368734762
C	-4.374544172	8.766575578	-0.345765904
H	-5.218758608	9.458484091	-0.610261618
H	-4.822626664	7.745927446	-0.337263026
C	-3.910459947	9.102527406	1.094123821
H	-4.712952224	8.814807068	1.816288277
H	-3.818478861	10.206125077	1.200681932
C	-2.577322363	8.435762061	1.518439534
H	-2.787869184	7.370987426	1.771935778
H	-2.243164986	8.907324661	2.480387680
B	-5.751421617	-5.735939425	0.419511163
C	-5.975682595	-7.383915967	0.390041115
H	-5.197051265	-7.893151171	1.011965175
C	-7.342238602	-7.712300190	1.037774594

H	-7.557919977	-8.815307458	1.034599901
H	-7.274941649	-7.415570397	2.109975798
C	-8.558990032	-6.984945690	0.411497269
H	-9.437205239	-7.079894747	1.096618501
H	-8.867812901	-7.519077256	-0.514466893
C	-8.326094468	-5.488116353	0.084180588
H	-9.187687953	-5.132953226	-0.542654954
H	-8.381810959	-4.912346026	1.036590300
C	-6.959455752	-5.158530543	-0.564039383
H	-6.911609291	-4.044442727	-0.643470205
C	-6.799271734	-5.709282809	-2.002693381
H	-7.620241552	-5.355338214	-2.681064213
H	-5.862682509	-5.278323868	-2.426514224
C	-6.713774260	-7.252485748	-2.116216210
H	-6.346846936	-7.527753805	-3.135104008
H	-7.738884573	-7.681310935	-2.060971376
C	-5.818961091	-7.932821737	-1.049545861
H	-4.754206755	-7.794368860	-1.349138328
H	-5.998830449	-9.040367124	-1.098631459
H	-5.948090976	-5.319759616	1.598528458
H	-1.086855567	7.869459430	-1.843494629
H	7.377982673	-3.296932078	-1.652462007

9.53. $ClSiMe_2CH_2CH_2B\{C_8H_{14}\}$ (**8**)

Charge = 0 Multiplicity = 1

Si -0.8165770000 -2.1082890000 1.2911930000

C -0.7799610000 -1.8515180000 3.1619890000

C -0.9418070000 -3.9426590000 0.8589410000

C -2.1763620000 -1.0858020000 0.4544220000

H -1.8862030000 -4.3784840000 1.2497210000

H -0.0963380000 -4.5105870000 1.2994430000

H -0.9197460000 -4.0954610000 -0.2399900000

H -1.7180390000 -2.2172890000 3.6319700000

H -0.6631560000 -0.7772960000 3.4145610000

H 0.0675760000 -2.4034590000 3.6185540000

H -1.9687530000 -0.0138540000 0.6714230000

C -3.6269020000 -1.4471360000 0.8501980000

H -2.0373620000 -1.1878370000 -0.6455860000

H -3.7739300000 -1.3607310000 1.9571400000

H -3.8390180000 -2.5298510000 0.6664830000

B -4.8257900000 -0.6364840000 0.2086860000
C -6.3434440000 -0.9966480000 0.5028160000
H -6.4513290000 -1.8520970000 1.2076740000
C -7.0133900000 0.2451050000 1.1663890000
H -8.0999110000 0.0519700000 1.3240340000
H -6.5827950000 0.3560350000 2.1887560000
C -6.8310450000 1.5781000000 0.4070190000
H -7.1234900000 2.4188490000 1.0743110000
H -7.5428980000 1.6250550000 -0.4420190000
C -5.3933530000 1.8190770000 -0.1056890000
H -5.3880570000 2.6877570000 -0.8041730000
H -4.7551630000 2.1290410000 0.7541290000
C -4.7109000000 0.5909030000 -0.7849000000
H -3.6584310000 0.8742440000 -1.0059250000
C -5.3645960000 0.1473970000 -2.1329450000
H -5.3621390000 1.0006220000 -2.8502600000
H -4.7057610000 -0.6252630000 -2.5930610000
C -6.7944300000 -0.4268960000 -2.0166420000
H -7.0646510000 -0.9248060000 -2.9740770000
H -7.5222190000 0.4025300000 -1.9079250000
C -6.9748030000 -1.4316140000 -0.8570910000
H -6.5133290000 -2.4029980000 -1.1514740000
H -8.0588370000 -1.6508860000 -0.7180010000
Cl 1.0313420000 -1.4097380000 0.5279080000

9.54. $[ClSiMe_2CH_2CH_2FB\{C_8H_{14}\}]^-$

Charge = –1 Multiplicity = 1
Cl -0.9163420000 -1.0911260000 1.0942890000
Si 1.0553650000 -0.7889770000 1.9034200000
C 2.3221540000 -0.6500040000 0.5293090000
C 1.3511630000 -2.2850370000 3.0304190000
C 0.9091130000 0.7827650000 2.9572330000
H 2.3157150000 -1.5947590000 -0.0608730000
C 3.7633930000 -0.3892210000 1.0458370000
H 2.0033070000 0.1461690000 -0.1815970000
H 0.6401790000 1.6548440000 2.3251810000
H 1.8811700000 1.0043080000 3.4469340000
H 0.1338380000 0.6728030000 3.7442500000
H 1.3711640000 -3.2208600000 2.4339340000
H 0.5562880000 -2.3772280000 3.7997840000

H 2.3316320000 -2.1899350000 3.5430740000
H 3.9984720000 -1.1066240000 1.8720940000
H 3.8155890000 0.6241110000 1.5096850000
C 6.4595910000 -0.5475610000 0.4578550000
H 6.5728800000 -1.2527040000 1.3194900000
C 6.5238530000 2.0220640000 -0.0151690000
H 7.3802030000 2.0783840000 -0.7211470000
H 6.5371080000 2.9978240000 0.5274990000
C 7.4255780000 -1.0358280000 -0.6493130000
H 8.4944510000 -1.0036030000 -0.3122350000
H 7.1915350000 -2.1082580000 -0.8328980000
C 7.3147500000 -0.2807800000 -1.9977600000
H 7.8485110000 -0.8620820000 -2.7876560000
H 7.8726030000 0.6793350000 -1.9309800000
C 6.7706700000 0.8712760000 0.9923010000
H 6.1313430000 1.0477000000 1.8889290000
H 7.8256560000 0.9510610000 1.3633720000
B 4.9151590000 -0.6189830000 -0.1306970000
F 4.6660230000 -1.9646440000 -0.6816180000
C 4.8931550000 0.4857070000 -1.3626370000
H 3.8794460000 0.5350130000 -1.8328250000
C 5.8635720000 -0.0056370000 -2.4657340000
H 5.9002650000 0.7048530000 -3.3320130000
H 5.4436490000 -0.9564880000 -2.8627450000
C 5.2073370000 1.9016570000 -0.8222810000
H 4.3638020000 2.2087800000 -0.1607570000
H 5.2296600000 2.6626830000 -1.6449530000

9.55. [$ClSiMe_2CH_2CH_2HB\{C_8H_{14}\}]^-$

Charge = –1 Multiplicity = 1

Si	-3.019718422	-0.212085275	-0.077213810
C	-3.547477095	-1.207984535	-1.603242612
C	-3.165244002	-1.298365321	1.471286169
C	-1.331471978	0.584060989	-0.266652412
H	-2.435360093	-2.133912644	1.419739119
H	-4.185210431	-1.724293601	1.575123853
H	-2.940917077	-0.708373282	2.384470959
H	-2.830610248	-2.037071789	-1.782733714
H	-3.553303522	-0.563184809	-2.506846172
H	-4.563023630	-1.638205125	-1.476818320

H	-1.351074767	1.281720860	-1.135712877
C	-0.183663340	-0.447774578	-0.444780332
H	-1.142152536	1.232709099	0.619835281
H	-0.417476654	-1.104361332	-1.320319922
H	-0.179897921	-1.135702802	0.438052190
B	1.302740114	0.248914274	-0.699929449
C	2.492592248	-0.854503589	-1.042661683
H	2.187648381	-1.505351187	-1.900536248
C	3.760998257	-0.092186713	-1.496845367
H	4.609775302	-0.788902322	-1.728642041
H	3.510761946	0.416084040	-2.456511366
C	4.275966341	0.978990669	-0.502686371
H	5.012967794	1.638133083	-1.022053526
H	4.863182724	0.481928782	0.300484728
C	3.172095746	1.856677328	0.139249760
H	3.635838429	2.439820714	0.978304787
H	2.846118470	2.610266298	-0.614179938
C	1.903109928	1.095053549	0.595095197
H	1.179118535	1.875222155	0.940182778
C	2.137172428	0.149266929	1.799615849
H	2.572659216	0.692664898	2.678684873
H	1.140525956	-0.213580631	2.145248213
C	3.025681974	-1.085912795	1.507870870
H	2.924181904	-1.819350684	2.344102508
H	4.094230500	-0.779948585	1.530253263
C	2.728357874	-1.797216724	0.163954007
H	1.811311529	-2.418642490	0.293729022
H	3.554796664	-2.529551564	-0.033594509
Cl	-4.550487828	1.284747728	0.157180014
H	1.167849669	1.035162808	-1.682901002

9.56. ($\{C_8H_{14}\}$BO(Mes)CH)$_3$P$_7$ (**10**)

Charge = 0 Multiplicity = 1

P	0.700899844	1.086900679	-3.028074549
P	0.695377011	-1.170871530	-3.001857023
P	-1.257433905	-0.037652430	-3.031964763
P	1.265156628	1.483359739	-0.870552323
P	0.705235949	-1.834764681	-0.836957326
P	-1.887320226	0.310922884	-0.884948506
P	0.017790892	-0.001595678	0.236247534

O	0.568037423	3.400811662	0.892317611
O	2.671086539	-2.209110276	0.968821888
O	-3.246516197	-1.163876299	0.909299100
C	0.340357234	3.148623364	-0.499075823
H	-0.739210038	2.983854222	-0.681410976
C	0.861793440	4.259566899	-1.394219901
C	0.021197818	4.760459032	-2.434779427
C	-1.381631123	4.236185240	-2.669238625
H	-1.392331246	3.157117808	-2.934139126
H	-1.867585296	4.781623444	-3.501804865
H	-2.028866049	4.352233318	-1.773609248
C	0.502120975	5.783359210	-3.273874592
H	-0.158056136	6.163215128	-4.072031282
C	1.789109310	6.334442227	-3.125068944
C	2.287561237	7.410727473	-4.062828964
H	2.576732166	6.980810964	-5.047190920
H	3.177537175	7.930428533	-3.654901664
H	1.505249553	8.172264759	-4.262726712
C	2.596449623	5.831227102	-2.089705203
H	3.607705811	6.248854951	-1.946893752
C	2.167168730	4.807544932	-1.219781510
C	3.111263985	4.363406557	-0.121678433
H	2.818846977	4.793850624	0.858402371
H	4.146622668	4.689680670	-0.345664189
H	3.113697936	3.263176829	0.013205123
C	-0.018508779	4.028071052	3.298982322
H	1.067698726	3.868164562	3.475617185
C	-0.807010291	3.009817341	4.176501343
H	-0.386534433	1.997274850	3.978220988
H	-0.616106376	3.206339113	5.257144284
C	-2.734415286	2.964926007	2.432076046
H	-3.830699273	3.146326603	2.346055448
H	-2.570037661	1.943556411	2.018631237
C	-1.961006724	3.970243546	1.523826262
H	-2.277322980	3.774822457	0.475374552
C	-2.248237621	5.476338719	1.823811871
H	-1.752921619	6.082042672	1.030184467
H	-3.338996113	5.679678199	1.717488530
C	-1.767621375	5.978952223	3.204904371
H	-1.811100726	7.090601273	3.217541070

H	-2.484173123	5.655408966	3.986987492
C	-0.339269497	5.526742588	3.587991201
H	-0.155094235	5.757517580	4.663040972
H	0.394424539	6.145127902	3.021168495
C	2.599739359	-1.866375369	-0.420064437
H	2.998989205	-0.845943496	-0.578682082
C	3.325358501	-2.859308504	-1.311781695
C	4.204275317	-2.365767062	-2.322825630
C	4.457462945	-0.885360539	-2.528528500
H	3.535664091	-0.331665371	-2.808456396
H	5.193825628	-0.725046434	-3.340244241
H	4.858792129	-0.396686670	-1.614895529
C	4.871314442	-3.281182435	-3.159994293
H	5.549902400	-2.887369451	-3.935527190
C	4.702610746	-4.672778876	-3.036632837
C	5.400578318	-5.631633668	-3.974497454
H	6.383906965	-5.238911128	-4.304781000
H	4.794820389	-5.803736767	-4.891871333
H	5.564473349	-6.621333715	-3.502001512
C	3.838976473	-5.136747221	-2.027713586
H	3.693525343	-6.223489422	-1.903524099
C	3.145234815	-4.267078401	-1.162018977
C	2.263776820	-4.878851381	-0.092664661
H	2.758250197	-4.850003670	0.900306203
H	2.038636582	-5.936807383	-0.334803298
H	1.304468777	-4.336011951	0.024959513
C	3.465225779	-2.066747991	3.392593582
H	2.772824580	-2.924189387	3.540776446
C	2.989829734	-0.895677987	4.303309940
H	1.904478925	-0.735872284	4.109056004
H	3.060476437	-1.191026526	5.376000892
C	3.732976851	0.443528290	4.091813319
H	3.167303903	1.255228432	4.600131534
H	4.713784905	0.411003629	4.608938879
C	3.939121240	0.833584212	2.609612256
H	4.652179619	1.688075980	2.550350320
H	2.977407769	1.221737111	2.202727938
C	4.416823825	-0.317434955	1.671486067
H	4.416243800	0.083332326	0.633691692
C	5.857898852	-0.845374546	1.965759794

H	6.132683982	-1.555619581	1.152060025
H	6.588581718	-0.007061547	1.889894668
C	6.036708321	-1.557019128	3.327320960
H	7.014746519	-2.087318470	3.329477267
H	6.119696437	-0.799648578	4.133148693
C	4.916458776	-2.565517048	3.673878473
H	5.015114137	-2.874302398	4.740584616
H	5.076564797	-3.494418349	3.079318200
C	-2.880774421	-1.302383734	-0.468995751
H	-2.198468987	-2.164967675	-0.595092361
C	-4.080391849	-1.442431070	-1.390647494
C	-4.068727644	-2.463268367	-2.388890811
C	-2.909980734	-3.426517844	-2.553294498
H	-1.960650879	-2.909592795	-2.810297282
H	-3.117505663	-4.151378712	-3.364647281
H	-2.714939743	-4.010280483	-1.628011187
C	-5.173950530	-2.593182699	-3.251940296
H	-5.154054815	-3.388015812	-4.016728084
C	-6.296484449	-1.748555283	-3.167408051
C	-7.452873160	-1.886569726	-4.131687344
H	-7.261662317	-1.316313849	-5.067813867
H	-8.396489118	-1.496944841	-3.699029252
H	-7.617518935	-2.943605107	-4.425057734
C	-6.289381991	-0.754711296	-2.172066101
H	-7.159247098	-0.082243815	-2.078860398
C	-5.210708595	-0.578254094	-1.281602647
C	-5.324464955	0.507425467	-0.231279651
H	-5.576329300	0.081600692	0.762021695
H	-6.118138804	1.230549902	-0.506519895
H	-4.376140943	1.065049172	-0.095949427
C	-2.632582147	-3.656605943	1.645188720
H	-2.338754781	-3.908064717	0.603950275
C	-3.748893291	-4.653735374	2.121128885
H	-4.062186664	-5.294892413	1.269627467
H	-3.331889384	-5.352080228	2.882237871
C	-4.997066150	-3.950884819	2.695973893
H	-5.525958294	-3.433156780	1.860010637
H	-5.716812313	-4.706156027	3.081292370
C	-4.674449070	-2.910918659	3.789383516
H	-4.344238016	-3.436833927	4.714316077

H	-5.610761382	-2.381533226	4.067935027
C	-3.590121516	-1.869987666	3.341979270
H	-3.981018907	-0.837286670	3.461087501
C	-2.271357941	-1.992205078	4.169796699
H	-1.569448734	-1.186163409	3.854982820
H	-2.481300505	-1.809268349	5.249023425
C	-1.581668865	-3.358941073	3.999072567
H	-0.613852705	-3.363155705	4.546237805
H	-2.198957354	-4.145995307	4.487840788
C	-1.342690202	-3.741136489	2.526229754
H	-0.920434449	-4.770716127	2.470358771
H	-0.564038970	-3.072229157	2.093004014
B	-0.414048589	3.754240548	1.791197703
B	3.447694067	-1.550688099	1.896413523
B	-3.171736181	-2.175085295	1.841624173
C	-2.332125640	2.969679374	3.925201413
H	-2.754666058	2.063246462	4.412126531
H	-2.817356888	3.823881739	4.440328069

9.57. [({C_8H_{14}}BO(Mes)CH)$_2$({C_8H_{14}}BFO(Mes)CH)P_7]$^-$

Charge = –1 Multiplicity = 1

P	-0.819232624	0.016395668	-3.166162532
P	1.062377769	1.246468681	-2.956358374
P	1.184674854	-1.011923179	-3.019011600
P	-1.681585378	0.234411774	-1.083327219
P	1.400446560	1.554991024	-0.737915877
P	1.034522090	-1.767702572	-0.891358266
P	0.127196777	-0.033615928	0.187810042
O	-3.110040760	-1.172156891	0.604595379
O	0.407473606	3.321564707	1.044730671
O	2.884600769	-2.174498496	1.050689080
C	-2.731767633	-1.382844034	-0.697731575
H	-2.031512470	-2.243039332	-0.808642348
C	-3.800943218	-1.470190068	-1.789619008
C	-3.641973329	-2.423540655	-2.840449781
C	-2.481908191	-3.399575520	-2.877557102
H	-1.497195216	-2.891295152	-2.964714511
H	-2.576251356	-4.083438184	-3.745345782
H	-2.438569241	-4.020570848	-1.957049849
C	-4.597012399	-2.484346915	-3.874117874

H	-4.461502155	-3.233253724	-4.674318332
C	-5.712846024	-1.627876713	-3.912160173
C	-6.706417161	-1.691407495	-5.052377497
H	-6.307228493	-1.201479687	-5.969103335
H	-7.657116475	-1.181222057	-4.794353411
H	-6.946362742	-2.740246179	-5.328655774
C	-5.857358933	-0.702914379	-2.863378565
H	-6.731349982	-0.027887218	-2.858705771
C	-4.933778228	-0.604482347	-1.801615832
C	-5.217579982	0.380407994	-0.691521966
H	-5.532342341	-0.182629701	0.211821992
H	-6.018429163	1.087741610	-0.992088171
H	-4.318227991	0.958108610	-0.399193841
C	-3.677033121	-1.873029667	3.007389883
H	-4.072871187	-0.842566864	3.181832163
C	-2.183143501	-1.871197857	3.409540515
H	-1.690979713	-1.040883828	2.852616545
H	-2.049254799	-1.635429236	4.495613651
C	-1.701731399	-3.762603194	1.688358201
H	-1.262357572	-4.790513013	1.634102250
H	-1.125847098	-3.160685669	0.946494169
C	-3.189321773	-3.761326845	1.258454858
H	-3.232548283	-4.107398441	0.195831685
C	-4.091309278	-4.729942803	2.067107456
H	-5.087150469	-4.737926628	1.571008214
H	-3.708966947	-5.780581029	2.014710071
C	-4.292036438	-4.353879134	3.556735548
H	-5.134330347	-4.955973362	3.972540757
H	-3.401821025	-4.671874125	4.141072673
C	-4.568729500	-2.850910086	3.813338872
H	-4.498765425	-2.658738368	4.913777884
H	-5.623948903	-2.638373288	3.530162950
C	0.324528493	3.129627685	-0.374718841
H	-0.717887624	2.888021815	-0.660110142
C	0.821134042	4.335991133	-1.153826160
C	0.032333901	4.835010295	-2.233777588
C	-1.293870881	4.213692270	-2.622342678
H	-1.188172483	3.153842523	-2.937843229
H	-1.752030898	4.766121117	-3.466440537
H	-2.021508374	4.220657264	-1.782977691

C	0.492000834	5.948206443	-2.965453798
H	-0.129187292	6.323564830	-3.796796294
C	1.707064288	6.592087358	-2.669158249
C	2.196248835	7.760173585	-3.497098008
H	1.353301101	8.368357262	-3.885634713
H	2.778204497	7.411700842	-4.379653976
H	2.861383440	8.427823026	-2.911727231
C	2.464198841	6.090220630	-1.595172484
H	3.419684996	6.578628105	-1.335979081
C	2.053295506	4.979009971	-0.830863935
C	2.936100103	4.534181464	0.316724883
H	2.512306720	4.842708649	1.294525283
H	3.948982797	4.975465653	0.221151804
H	3.034960526	3.430600409	0.359715415
C	-0.431453061	3.696273059	3.425793003
H	0.645980151	3.597584666	3.684812979
C	-1.212880192	2.559805829	4.151599675
H	-0.709217441	1.595965515	3.912670342
H	-1.122054178	2.679193762	5.257073687
C	-2.705772472	2.437769173	3.769070676
H	-3.092434705	1.462961608	4.137582384
H	-3.293698498	3.208318310	4.310873073
C	-2.986590630	2.528702256	2.250917607
H	-4.081206787	2.657453880	2.088108738
H	-2.738369733	1.550377191	1.781734326
C	-2.207596829	3.642357094	1.484609291
H	-2.418294450	3.503720206	0.401291283
C	-2.620606813	5.102137309	1.858479420
H	-2.098701809	5.798117275	1.160741617
H	-3.709476735	5.245189414	1.665525160
C	-2.303487509	5.525508335	3.311987012
H	-2.432251881	6.627765388	3.403847679
H	-3.059083108	5.087619850	3.995361807
C	-0.884879893	5.140794448	3.794349251
H	-0.816518680	5.296770463	4.897130174
H	-0.151973955	5.851970004	3.346576688
C	2.886234916	-1.702036263	-0.303812438
H	3.218788156	-0.646527929	-0.333002898
C	3.770609820	-2.558270659	-1.196148428
C	4.710331526	-1.919644595	-2.059720602

C	4.869956939	-0.413339005	-2.109160330
H	3.938972176	0.099350647	-2.432552739
H	5.668951406	-0.128615312	-2.822256974
H	5.138212846	0.012194599	-1.118436505
C	5.526317605	-2.708141911	-2.894555673
H	6.248601264	-2.200342343	-3.556774919
C	5.449255745	-4.112876172	-2.908245160
C	6.306672963	-4.934402012	-3.845775707
H	5.821749908	-5.043475301	-4.841677743
H	6.478239900	-5.957795716	-3.453564982
H	7.295580144	-4.460966617	-4.017890697
C	4.524462637	-4.722161559	-2.041510914
H	4.446011332	-5.823063220	-2.026843364
C	3.682595965	-3.982672861	-1.185010056
C	2.742545534	-4.745350868	-0.274492204
H	3.124619900	-4.769759528	0.766961363
H	2.625507502	-5.790388747	-0.625655691
H	1.737555667	-4.280706599	-0.220448690
C	4.182482586	-0.104485075	2.129795867
H	4.262051011	0.384754930	1.135564819
C	5.619976687	-0.294989004	2.731691468
H	6.382787226	-0.007231383	1.975928088
H	5.768001322	0.405444775	3.585828946
C	5.906830338	-1.738868106	3.197013232
H	5.972559074	-2.392569170	2.294394281
H	6.908606633	-1.793456337	3.679185005
C	4.825858413	-2.313210580	4.137165937
H	4.893675827	-1.808474382	5.128607993
H	5.054853269	-3.383234003	4.332936023
C	3.374900715	-2.177112568	3.558720751
H	2.879887715	-3.170532276	3.531369622
C	2.485371714	-1.212955478	4.404549660
H	1.452729521	-1.216051165	3.988866860
H	2.393494188	-1.596116150	5.447521208
C	3.021808773	0.230692636	4.426868710
H	2.308654792	0.887138299	4.971698292
H	3.965582103	0.265766429	5.018208548
C	3.278777398	0.807620618	3.021974484
H	3.740143705	1.818408087	3.110316663
H	2.305812428	0.965459676	2.504128074

B	-3.824811160	-2.257689335	1.424502127
B	-0.677066140	3.514460107	1.870742754
B	3.455944713	-1.520482322	2.113725759
C	-1.418718458	-3.179189478	3.093631720
H	-0.323117794	-2.997772992	3.195411772
H	-1.653556028	-3.942741707	3.865840320
F	-5.219139732	-2.247568127	1.012030528

9.58. [({C_8H_{14}}BO(Mes)CH)({C_8H_{14}}BFO(Mes)CH)$_2$$P_7$]$^{2-}$

Charge = –2 Multiplicity = 1

P	-1.142442192	0.911950204	-2.923502293
P	0.980200093	0.165922683	-3.029723777
P	-0.728744251	-1.311257725	-2.938427716
P	-1.256623931	1.596847420	-0.769610207
P	1.757568489	-0.037770063	-0.915140005
P	-1.099993217	-1.751954220	-0.743517049
P	-0.123900752	-0.017568477	0.283978827
O	-3.195922867	1.657445851	1.072735525
O	3.271318407	1.402094963	0.722588110
O	0.102774313	-3.455941946	0.991620712
C	-3.143855493	1.295842109	-0.251339266
H	-3.335501569	0.208303291	-0.408126717
C	-3.982367881	2.100244127	-1.251898632
C	-4.699163367	1.420142119	-2.278960731
C	-4.724419135	-0.092291418	-2.382725955
H	-3.722136369	-0.522009050	-2.596480225
H	-5.402585011	-0.414785809	-3.199600252
H	-5.076935761	-0.560816900	-1.438811318
C	-5.432508926	2.164630579	-3.227371977
H	-5.984080850	1.619818116	-4.014499804
C	-5.482466539	3.568272295	-3.198726353
C	-6.246800950	4.355120689	-4.242689654
H	-5.560618928	4.901133421	-4.929103926
H	-6.907912723	5.119622574	-3.779122964
H	-6.881484183	3.693375004	-4.868272133
C	-4.785151278	4.221862079	-2.165132419
H	-4.819878646	5.324604657	-2.103949808
C	-4.047264099	3.525532040	-1.187767451
C	-3.403072265	4.317698412	-0.073365568
H	-3.999188758	4.174562628	0.851669242

H	-3.366970941	5.397202749	-0.331499230
H	-2.379023645	3.967640163	0.162081699
C	-4.076093850	1.676902466	3.477298322
H	-3.627602658	2.691651838	3.604312414
C	-3.012333970	0.644390942	3.917862846
H	-2.083992057	0.866176874	3.346206036
H	-2.744081058	0.764011062	4.998458166
C	-4.019336918	-1.111222808	2.279384724
H	-4.432718209	-2.152372616	2.275853423
H	-3.196871185	-1.119390066	1.527645414
C	-5.077791007	-0.081865680	1.813713613
H	-5.361415338	-0.346247025	0.764384556
C	-6.393659701	-0.093903479	2.633063841
H	-7.106997936	0.584521305	2.113429732
H	-6.869345015	-1.108643149	2.630428993
C	-6.257389786	0.378697053	4.102329553
H	-7.276221941	0.560940775	4.522615396
H	-5.840281695	-0.447686273	4.717669320
C	-5.392610549	1.650178724	4.293262007
H	-5.201670803	1.788087739	5.388627061
H	-5.994496487	2.530441037	3.972252896
C	2.679442607	1.648734075	-0.497132089
H	1.882604071	2.424888866	-0.435382539
C	3.579605414	1.957304685	-1.699441077
C	3.198261111	2.987058094	-2.609751599
C	1.958278593	3.832642988	-2.398330317
H	1.023482442	3.232168932	-2.399715857
H	1.864636482	4.594709435	-3.198956789
H	1.987897871	4.363382813	-1.422344386
C	3.999234183	3.247538446	-3.741608695
H	3.689442158	4.051437026	-4.433016607
C	5.170256650	2.519541864	-4.012498058
C	5.995007778	2.784614808	-5.254576743
H	5.773508840	3.783023212	-5.686452409
H	5.792213139	2.031522265	-6.050135030
H	7.084832447	2.739843248	-5.041701775
C	5.536132732	1.514478553	-3.097921162
H	6.458430203	0.932542782	-3.276525929
C	4.775626231	1.219049721	-1.948798695
C	5.290578454	0.162231905	-0.998446823

H	5.699977862	0.667453889	-0.098777369
H	6.082187349	-0.447506202	-1.483735355
H	4.485284592	-0.508097473	-0.638744208
C	4.135670979	2.015011665	3.059109699
H	4.611396415	1.004930631	3.110758092
C	2.722723813	1.895937761	3.677061437
H	2.201172929	1.064834178	3.151121388
H	2.767932918	1.597131292	4.755649805
C	1.842058839	3.162968598	3.556442649
H	0.788395883	2.900501037	3.804041233
H	2.143569324	3.900646096	4.331789026
C	1.877938750	3.835406664	2.162428571
H	1.377075690	4.833437834	2.236444842
H	1.232534587	3.236971956	1.478982803
C	3.282047052	3.954147689	1.520004788
H	3.146496236	4.356810760	0.485719338
C	4.238730307	4.936696339	2.244266886
H	5.147324252	5.034952727	1.608964611
H	3.793908812	5.962324997	2.314660901
C	4.682962632	4.498110748	3.662577830
H	5.541172987	5.134258749	3.988835882
H	3.871404703	4.721729332	4.388100709
C	5.082456180	3.005485755	3.781792463
H	5.192157699	2.753241719	4.867998422
H	6.093322508	2.879261127	3.332076438
C	0.187526756	-3.172460132	-0.414760829
H	1.192363233	-2.774396818	-0.655725035
C	-0.116867379	-4.403370177	-1.253860763
C	0.757250098	-4.748432696	-2.327606508
C	1.987180032	-3.929994319	-2.661303501
H	1.730101644	-2.888697691	-2.951117873
H	2.543202432	-4.384701539	-3.505639934
H	2.684687437	-3.851346160	-1.800350242
C	0.475638472	-5.887314175	-3.109674173
H	1.162072704	-6.140059813	-3.936658534
C	-0.643822272	-6.703639404	-2.870201523
C	-0.950390089	-7.895898077	-3.751205012
H	-1.650974130	-7.623139009	-4.572641739
H	-1.429805547	-8.717313780	-3.178207632
H	-0.032889489	-8.299894850	-4.227679620

C	-1.488983105	-6.351247521	-1.802229937
H	-2.375465849	-6.974054588	-1.588100332
C	-1.255731722	-5.223361170	-0.988702358
C	-2.221880577	-4.945706307	0.144008208
H	-1.783774484	-5.222108946	1.125042775
H	-3.159541323	-5.521587660	0.004762971
H	-2.477993280	-3.869265039	0.217817154
C	2.729478094	-3.472899034	1.491436201
H	2.951614617	-3.305545671	0.416314484
C	3.434432081	-4.793079862	1.967256609
H	3.886957270	-5.312493854	1.094100538
H	4.286446987	-4.541713921	2.640676659
C	2.488034365	-5.777690081	2.687867850
H	1.771455677	-6.189848830	1.937356724
H	3.062497481	-6.653819112	3.067226691
C	1.676365901	-5.130248543	3.830205429
H	2.359235380	-4.900418074	4.680987981
H	0.952417404	-5.878055108	4.223105047
C	0.916739745	-3.835970884	3.380156683
H	-0.167341739	-3.932564978	3.601104700
C	1.449509271	-2.551116442	4.090760892
H	0.833274093	-1.680999150	3.771894472
H	1.305858503	-2.638467847	5.193700488
C	2.929890896	-2.261906597	3.778643689
H	3.226211693	-1.294666145	4.238160182
H	3.567971511	-3.034793814	4.268107524
C	3.239237121	-2.216394956	2.270753821
H	4.337176569	-2.104635055	2.118882422
H	2.792743870	-1.299713564	1.825780140
B	-4.456704332	1.438514703	1.902331117
B	4.018117714	2.486494507	1.494132333
B	1.179911428	-3.584813217	1.831544240
C	-3.392101405	-0.835388860	3.668054280
H	-2.483356565	-1.470453519	3.786015122
H	-4.087499657	-1.182045706	4.463970476
F	-5.449439423	2.410637181	1.457981446
F	5.347846016	2.607308403	0.897412016

9.59. $[(\{C_8H_{14}\}BFO(Mes)CH)_3P_7]^{3-}$

Charge = –3 Multiplicity = 1

P	-0.572275270	-1.114686707	-2.834290708
P	-0.725838629	1.135164258	-2.799408693
P	1.299309865	0.143593564	-2.813685440
P	-1.128704449	-1.578230716	-0.683824701
P	-0.824904547	1.797283331	-0.631199273
P	1.948885437	-0.154310752	-0.660134267
P	-0.001314728	0.004546172	0.434583945
O	-0.839827795	-3.750804711	0.871621775
O	-2.836776518	2.585289401	0.967917551
O	3.678999158	1.141915634	0.939384382
C	-0.254488024	-3.315456996	-0.300540919
H	0.829582024	-3.086955202	-0.179470555
C	-0.444874870	-4.192018456	-1.545016790
C	0.662204308	-4.445170734	-2.408041450
C	2.051180019	-3.920362902	-2.109050394
H	2.091651596	-2.810835884	-2.068326749
H	2.770921304	-4.254450491	-2.885012956
H	2.417400214	-4.279204418	-1.122703182
C	0.476322231	-5.220192890	-3.572529050
H	1.347340596	-5.403747931	-4.227652040
C	-0.774071971	-5.757658129	-3.921764840
C	-0.965860173	-6.542222034	-5.203287339
H	-1.395375497	-5.909684244	-6.014508965
H	-1.661886305	-7.397957209	-5.062713419
H	-0.003128213	-6.946489903	-5.582636882
C	-1.850802551	-5.514560822	-3.048809978
H	-2.844756003	-5.936129295	-3.286859893
C	-1.715923197	-4.756249411	-1.868649678
C	-2.920149457	-4.617367281	-0.964355695
H	-2.790506461	-5.301699009	-0.100091175
H	-3.852060200	-4.875564423	-1.512248160
H	-3.017021798	-3.599090493	-0.540255834
C	-1.028286858	-4.994571624	3.096241797
H	-2.138169980	-4.891039212	3.022509006
C	-0.501031978	-3.769685591	3.879157044
H	-0.898809431	-2.863824884	3.371355624
H	-0.905964405	-3.740766716	4.923824441
C	1.765904290	-3.913951565	2.604911955
H	2.864717664	-3.992870636	2.804259926
H	1.649215028	-3.008674953	1.967523595

C	1.245736908	-5.131363842	1.802189058
H	1.782302345	-5.139916918	0.821167105
C	1.524915505	-6.506992958	2.460014932
H	1.227873768	-7.287302436	1.723192695
H	2.619544230	-6.657500493	2.651490047
C	0.758908275	-6.772611573	3.780889131
H	0.841790656	-7.857098854	4.041122404
H	1.267582292	-6.239999593	4.613437764
C	-0.736579180	-6.367209994	3.750605280
H	-1.139325338	-6.423142177	4.796093965
H	-1.291802931	-7.134656284	3.164105260
C	-2.762368439	1.892345907	-0.224038598
H	-3.102303829	0.835399904	-0.128050672
C	-3.439644091	2.527315160	-1.444982948
C	-4.218644385	1.716960099	-2.323023516
C	-4.453889294	0.243875232	-2.060414750
H	-3.511762795	-0.344818091	-2.043620199
H	-5.109114326	-0.193044601	-2.842423077
H	-4.938767174	0.079309675	-1.073788434
C	-4.806686311	2.294942630	-3.468155320
H	-5.405790819	1.649032658	-4.135567525
C	-4.650833742	3.655483838	-3.783353980
C	-5.244383672	4.246185197	-5.045614606
H	-6.082480460	3.627320131	-5.431393473
H	-4.489547618	4.317318262	-5.862834965
H	-5.631120895	5.275013806	-4.877136165
C	-3.895983611	4.444294533	-2.895466577
H	-3.767118478	5.521878972	-3.106521557
C	-3.296812971	3.918260666	-1.733329128
C	-2.569223305	4.868262222	-0.808604988
H	-3.221653741	5.073745050	0.065618429
H	-2.331835058	5.819090792	-1.332954061
H	-1.635911745	4.432961313	-0.401754647
C	-3.796956005	3.309290256	3.222881236
H	-3.157715312	4.223674945	3.165017000
C	-2.986141760	2.224078356	3.968481428
H	-2.008395811	2.133149649	3.446848585
H	-2.747869475	2.534312651	5.018774371
C	-3.643561951	0.822989475	4.008267921
H	-2.886463854	0.074073741	4.335453293

H	-4.427699169	0.800462104	4.797925601
C	-4.252853884	0.360700116	2.661501938
H	-4.865448243	-0.558131812	2.845535444
H	-3.417359336	0.027779609	2.005297643
C	-5.060173220	1.436989694	1.894963885
H	-5.345106042	1.000364895	0.905682517
C	-6.385288435	1.860369478	2.578546064
H	-6.923060898	2.524644921	1.864675869
H	-7.057496050	0.980237813	2.754676023
C	-6.220099036	2.622297555	3.917949049
H	-7.199366777	3.082016269	4.201508413
H	-6.001306310	1.894618993	4.729232076
C	-5.125791606	3.719585253	3.903733782
H	-4.962101747	4.069462603	4.956889330
H	-5.522968548	4.597703600	3.344695690
C	3.011263863	1.462582865	-0.225197163
H	2.268638335	2.281134221	-0.083127787
C	3.868497901	1.772542649	-1.458749854
C	3.534429843	2.880899180	-2.291492712
C	2.388523504	3.815238195	-1.963064292
H	1.405010073	3.298455166	-1.941638302
H	2.322630147	4.631995905	-2.711663379
H	2.516197131	4.276964840	-0.960005589
C	4.297185126	3.137224643	-3.450742647
H	4.020470585	4.000424266	-4.083115487
C	5.386980314	2.331700329	-3.822530469
C	6.159745789	2.589528545	-5.099564036
H	5.822296336	1.924217083	-5.927908685
H	7.248663765	2.407416756	-4.966720533
H	6.030860172	3.635557066	-5.450881219
C	5.716682823	1.256448122	-2.976471115
H	6.579413034	0.613651311	-3.231263679
C	4.993661720	0.964396559	-1.802731322
C	5.479266287	-0.167015958	-0.924235498
H	6.013889352	0.267531624	-0.053876108
H	6.165345119	-0.833942217	-1.489780903
H	4.648296665	-0.767637477	-0.506439020
C	3.719882471	3.569224100	2.028269492
H	3.422892048	4.103884170	1.094643595
C	4.644766084	4.526909198	2.853858275

H	4.907774988	5.425938238	2.248791934
H	4.085587734	4.928911205	3.735515193
C	5.966897353	3.887509176	3.341597823
H	6.637467906	3.786940622	2.462910982
H	6.477416765	4.573357123	4.062654780
C	5.801973072	2.484386831	3.973191217
H	5.403773913	2.597132478	5.012819963
H	6.821059126	2.049478402	4.104169418
C	4.892956363	1.507516792	3.155327590
H	5.438601237	0.546362190	3.006688559
C	3.575230415	1.193675942	3.904960420
H	3.007363281	0.442666614	3.314063757
H	3.774058997	0.726488696	4.903948946
C	2.690648190	2.440050948	4.108816388
H	1.721892084	2.146862320	4.573551654
H	3.179215863	3.118695200	4.849786898
C	2.424784745	3.225055128	2.808108489
H	1.843228559	4.147947140	3.057606296
H	1.756912251	2.615383309	2.159560325
B	-0.380170010	-5.003144751	1.588229314
B	-4.143712870	2.789765565	1.704969069
B	4.511701295	2.161638584	1.692335799
C	1.040695689	-3.645973846	3.946893282
H	1.309468137	-2.625119345	4.302865373
H	1.437006781	-4.334185993	4.726691184
F	-0.870736429	-6.163359064	0.837066749
F	-4.914996664	3.811094414	0.989129150
F	5.714685074	2.415682039	0.894448978

9.60. [({C_8H_{14}}BO(Mes)CH)$_2$({C_8H_{14}}BHO(Mes)CH)P_7]$^-$

Charge = –1 Multiplicity = 1

P	-0.828355439	0.019048743	-3.147518019
P	0.996007755	1.324043864	-2.891547559
P	1.215335225	-0.925466422	-2.989486399
P	-1.731748253	0.141509503	-1.075152388
P	1.285128243	1.612555190	-0.664724985
P	1.075363070	-1.721832355	-0.874714906
P	0.073199818	-0.048123982	0.217919687
O	-3.047643148	-1.454066821	0.553976998
O	0.187084189	3.343372567	1.106498234

O	2.952327530	-2.117156569	1.054774931
C	-2.655382654	-1.565727205	-0.750703853
H	-1.890417204	-2.363318421	-0.911069547
C	-3.731713229	-1.684071759	-1.835708071
C	-3.529815127	-2.575315352	-2.931309223
C	-2.314588552	-3.477887159	-3.023640906
H	-1.364262981	-2.908543510	-3.115866007
H	-2.387705211	-4.139535598	-3.910663654
H	-2.214174481	-4.121567707	-2.123822228
C	-4.496058932	-2.648903037	-3.953903262
H	-4.326326483	-3.349122501	-4.790945642
C	-5.667127173	-1.868770288	-3.934473900
C	-6.672908229	-1.942617359	-5.063342822
H	-6.327524399	-1.372845606	-5.955472417
H	-7.654393540	-1.520737010	-4.764100687
H	-6.838903777	-2.989196960	-5.396575515
C	-5.857214256	-1.010240369	-2.837241104
H	-6.776065301	-0.399734420	-2.784638583
C	-4.922146992	-0.901678206	-1.786888263
C	-5.247571770	0.004189914	-0.621826343
H	-5.467598087	-0.609361033	0.277009454
H	-6.125019114	0.642329347	-0.856053740
H	-4.394385493	0.654500307	-0.342989050
C	-4.097756075	-2.281086531	2.761993713
H	-4.720472457	-1.354670384	2.812768656
C	-2.782991636	-1.980510530	3.522251078
H	-2.346966389	-1.059522371	3.072020730
H	-2.974473418	-1.736890761	4.598630025
C	-1.516973184	-3.707793613	2.045831810
H	-0.867670107	-4.615105604	2.137710967
H	-0.928552484	-2.982951853	1.435836791
C	-2.823420587	-4.013858150	1.273818328
H	-2.521755174	-4.364911432	0.255249359
C	-3.681068262	-5.152939611	1.880487108
H	-4.501629115	-5.367832776	1.157526876
H	-3.096221069	-6.103439107	1.971951681
C	-4.319123984	-4.838126414	3.256107191
H	-5.098392062	-5.604476768	3.481563782
H	-3.556209340	-4.967502282	4.053836771
C	-4.945611732	-3.426179573	3.369085105

H	-5.188694345	-3.231887097	4.444808385
H	-5.922280567	-3.435157075	2.832097649
C	0.135828782	3.136819695	-0.312513417
H	-0.889978388	2.847637214	-0.612610678
C	0.586965310	4.365026078	-1.085958447
C	-0.214466699	4.833866521	-2.170033970
C	-1.510504273	4.158295138	-2.570052034
H	-1.357802330	3.104840313	-2.887710971
H	-1.985181472	4.693480432	-3.416164205
H	-2.243994233	4.132536594	-1.736220441
C	0.202492388	5.968310151	-2.894579401
H	-0.427783677	6.319513925	-3.729637584
C	1.386328434	6.662874129	-2.586515129
C	1.831331331	7.853633484	-3.407060489
H	0.966116221	8.428551280	-3.797370536
H	2.431317327	7.533148184	-4.288139232
H	2.465674123	8.545352500	-2.815498502
C	2.155317493	6.190997304	-1.507411046
H	3.086080710	6.720062685	-1.238260443
C	1.786495312	5.060677182	-0.749640942
C	2.675827147	4.653864149	0.407031901
H	2.228244045	4.943616649	1.379965130
H	3.669628164	5.138619556	0.322137871
H	2.822100353	3.555604072	0.451349132
C	-0.714429842	3.693014660	3.469528450
H	0.364012542	3.722955144	3.741083069
C	-1.373072221	2.498275072	4.221960754
H	-0.761555082	1.590995254	4.014194452
H	-1.308257549	2.657134093	5.324347689
C	-2.839139797	2.200745053	3.831346181
H	-3.118276235	1.199245903	4.224032007
H	-3.513658626	2.915421057	4.348145445
C	-3.114007441	2.220997163	2.309440689
H	-4.214572821	2.228043846	2.136771555
H	-2.760708717	1.262946583	1.866366543
C	-2.451323143	3.389425297	1.516018342
H	-2.633753174	3.193651909	0.436410297
C	-3.026522525	4.804843091	1.840128860
H	-2.574473585	5.533899339	1.127434391
H	-4.121804567	4.822109562	1.631956045

C	-2.776630036	5.301204175	3.283250348
H	-3.026668895	6.384682580	3.342374423
H	-3.488044796	4.801640997	3.971754278
C	-1.330190290	5.088806143	3.789508031
H	-1.292595093	5.283630410	4.887593940
H	-0.674653975	5.863044466	3.326709099
C	2.919282374	-1.579880781	-0.274753029
H	3.190501240	-0.506766836	-0.253729441
C	3.853677138	-2.340779127	-1.202603771
C	4.763801342	-1.609791805	-2.023521443
C	4.847391101	-0.096935809	-1.990636125
H	3.893733882	0.385635001	-2.293349981
H	5.635064006	0.265422760	-2.680764519
H	5.088421844	0.287148149	-0.976308143
C	5.623632508	-2.309873350	-2.892565352
H	6.321637567	-1.731076230	-3.521744316
C	5.619948323	-3.713957369	-2.980546150
C	6.521903109	-4.439423213	-3.954806017
H	6.042228222	-4.529442645	-4.955158254
H	6.753016986	-5.468596909	-3.611189337
H	7.481449155	-3.901995890	-4.103462828
C	4.725753360	-4.415281470	-2.152094518
H	4.706082460	-5.518097260	-2.194304669
C	3.842646079	-3.766553724	-1.264233034
C	2.943923629	-4.624615464	-0.397868790
H	3.329568963	-4.686561689	0.640773883
H	2.879286163	-5.653489270	-0.805822262
H	1.916890317	-4.215465019	-0.318689502
C	3.972318323	0.034631941	2.264298748
H	3.980686716	0.592044189	1.303308419
C	5.424853742	-0.001729030	2.858417671
H	6.139717964	0.432582339	2.126086927
H	5.483132297	0.655311441	3.756712668
C	5.901176714	-1.422377660	3.230538817
H	6.046490956	-2.002713707	2.287909092
H	6.904282409	-1.376454791	3.710890951
C	4.910185898	-2.192332468	4.129314148
H	4.918680849	-1.748193368	5.151541667
H	5.277867209	-3.233824156	4.255274341
C	3.448834227	-2.209625896	3.560105000

H	3.087446744	-3.255529424	3.469823953
C	2.449807505	-1.424944958	4.466211335
H	1.422059418	-1.539429408	4.053722654
H	2.420303238	-1.879506707	5.483899657
C	2.791515670	0.072841873	4.576013053
H	2.002222017	0.595595799	5.159275254
H	3.726784895	0.196704323	5.169028234
C	2.959383505	0.761329578	3.208501252
H	3.279485014	1.818486553	3.356702018
H	1.970601422	0.814765119	2.699214252
B	-3.769823214	-2.667749827	1.203706709
B	-0.920175539	3.440109412	1.918592527
B	3.436731827	-1.460256342	2.158484211
C	-1.712701963	-3.096177375	3.453740320
H	-0.737532425	-2.690755777	3.813758875
H	-1.967570305	-3.901928875	4.175829733
H	-4.820587749	-2.896578193	0.552409363

9.61. [({C_8H_{14}}BO(Mes)CH)({C_8H_{14}}BHO(Mes)CH)$_2$$P_7$]$^{2-}$

Charge = –2 Multiplicity = 1

P	1.135293811	-1.066385260	-2.862260673
P	-0.950816177	-0.227016310	-2.994934495
P	0.823026010	1.172620318	-2.917507614
P	1.216032998	-1.712293517	-0.695068734
P	-1.733800921	0.026965946	-0.888612169
P	1.200101398	1.635307488	-0.728890071
P	0.141147388	-0.036129111	0.321739010
O	3.162624722	-1.741856761	1.149738641
O	-3.313340668	-1.386757549	0.732880501
O	0.092668364	3.459557258	0.949874776
C	3.109023507	-1.445748985	-0.184596148
H	3.333758883	-0.373453902	-0.402633913
C	3.929815494	-2.330470569	-1.134791781
C	4.686688294	-1.736771765	-2.185842469
C	4.777890310	-0.233684427	-2.362205768
H	3.797923177	0.225932628	-2.613817347
H	5.481999328	0.020151294	-3.181580661
H	5.134546325	0.263122758	-1.434992203
C	5.404171378	-2.559886115	-3.078980770
H	5.987760378	-2.081188370	-3.885946827

C	5.404193021	-3.961144682	-2.968566371
C	6.144579410	-4.831437031	-3.962371114
H	5.454406439	-5.266564571	-4.720985261
H	6.650894085	-5.685834171	-3.463676781
H	6.915577274	-4.254910674	-4.515441271
C	4.673523841	-4.528778057	-1.907929061
H	4.670985319	-5.626459267	-1.780637564
C	3.945242895	-3.749945877	-0.986298528
C	3.249347729	-4.450156171	0.158870798
H	3.782392587	-4.240016091	1.109268578
H	3.222790953	-5.547026110	-0.013015541
H	2.213825706	-4.085202361	0.309443648
C	4.339959999	-2.077929739	3.418484097
H	4.041012600	-3.154731938	3.418813780
C	3.208116551	-1.300236413	4.132669423
H	2.259221340	-1.557310418	3.611798203
H	3.078591365	-1.638140597	5.193185260
C	3.789869353	0.831479722	2.758162973
H	4.051839524	1.911073723	2.903685229
H	2.900092121	0.827752864	2.088085312
C	4.917054888	0.061494420	2.028670962
H	5.046383935	0.551282253	1.031007856
C	6.300957584	0.128013915	2.720828756
H	7.039701036	-0.339360137	2.028309582
H	6.642127470	1.186239017	2.865082622
C	6.391096208	-0.601207904	4.084220880
H	7.464639681	-0.701125506	4.377323511
H	5.939886400	0.038970544	4.872993463
C	5.721270420	-1.997638638	4.112806982
H	5.669322412	-2.341602490	5.178593780
H	6.396698819	-2.716346026	3.592208834
C	-2.697809207	-1.640381065	-0.466200602
H	-1.912519128	-2.430110110	-0.394386308
C	-3.591072569	-1.936073972	-1.679540790
C	-3.233574771	-2.979948169	-2.582301323
C	-2.028096932	-3.868374142	-2.347969958
H	-1.071820000	-3.302655606	-2.346757237
H	-1.954879592	-4.643549876	-3.138286903
H	-2.088000760	-4.383927898	-1.365496980
C	-4.030137806	-3.217126116	-3.721972726

H	-3.738573678	-4.031891939	-4.408763551
C	-5.177309329	-2.455481397	-4.005064594
C	-5.992881332	-2.695709029	-5.258191923
H	-5.860564371	-3.729807196	-5.640336623
H	-5.695469378	-2.004650993	-6.080099321
H	-7.077554567	-2.534480236	-5.079598396
C	-5.525783718	-1.442662146	-3.092350735
H	-6.432487235	-0.838440377	-3.277100531
C	-4.765654118	-1.168484883	-1.937493543
C	-5.258483563	-0.102728537	-0.985125045
H	-5.650906091	-0.587221791	-0.065724154
H	-6.061664902	0.502529676	-1.456727481
H	-4.444755960	0.570740317	-0.650581231
C	-4.755586003	-1.922920409	2.801977415
H	-5.298869074	-0.955657477	2.664542421
C	-3.563664039	-1.638632410	3.747873026
H	-2.986451345	-0.799796912	3.298182942
H	-3.908557772	-1.275067938	4.750759541
C	-2.589355430	-2.822097104	3.961566860
H	-1.656822459	-2.446002911	4.442594392
H	-3.023122116	-3.534016487	4.698225739
C	-2.217178805	-3.580578423	2.664667534
H	-1.667162810	-4.514737217	2.943877575
H	-1.481828592	-2.958684933	2.105330558
C	-3.400386129	-3.874288608	1.710609162
H	-2.966952666	-4.342381684	0.791937053
C	-4.437123298	-4.888148534	2.256157679
H	-5.143253863	-5.117746651	1.424369838
H	-3.957364832	-5.863151413	2.530162404
C	-5.265434978	-4.396927581	3.469442739
H	-6.131878733	-5.084757689	3.625367932
H	-4.655783655	-4.494574341	4.393622700
C	-5.784055395	-2.942079245	3.350143343
H	-6.186145595	-2.628054681	4.348518251
H	-6.655904494	-2.940406003	2.654800268
C	-0.023199486	3.119620017	-0.442157644
H	-1.046894450	2.754540024	-0.654044786
C	0.316844057	4.304277699	-1.333142935
C	-0.562405342	4.646592587	-2.403851594
C	-1.832418446	3.870438272	-2.684191597

H	-1.626839213	2.807899204	-2.935093285
H	-2.383194335	4.315347367	-3.537166425
H	-2.517076374	3.857584626	-1.809518253
C	-0.248350300	5.742781528	-3.232839347
H	-0.939090440	5.993455648	-4.056892955
C	0.908284090	6.519713426	-3.043151200
C	1.246929721	7.663904073	-3.974691112
H	1.879429521	7.320804965	-4.824558907
H	1.812889705	8.464007902	-3.453083478
H	0.334376902	8.119083874	-4.413405557
C	1.757220973	6.172080487	-1.976892180
H	2.672196234	6.764955489	-1.801061059
C	1.492519268	5.085547623	-1.117636998
C	2.466596828	4.810465599	0.008979757
H	2.059485250	5.145882318	0.985010420
H	3.426136508	5.337413075	-0.169700695
H	2.675368339	3.727319073	0.122370198
C	-2.522645251	3.543177974	1.494482728
H	-2.767309733	3.327884375	0.432917174
C	-3.205016688	4.890863022	1.922184577
H	-3.670733203	5.373827138	1.035162178
H	-4.045211060	4.680783041	2.624003348
C	-2.233699365	5.897245033	2.576299368
H	-1.527607805	6.264522990	1.793250106
H	-2.790597834	6.797279435	2.924591609
C	-1.407160105	5.294877385	3.732415188
H	-2.076307154	5.113740287	4.605688964
H	-0.667714593	6.051825592	4.075873675
C	-0.670370613	3.971565103	3.331127919
H	0.418606347	4.065679060	3.527247331
C	-1.204260064	2.730145175	4.113982708
H	-0.600863706	1.839035363	3.830664707
H	-1.043050073	2.872037000	5.208891500
C	-2.692296259	2.441794735	3.839169474
H	-2.991804645	1.500882174	4.348753777
H	-3.314924030	3.243699778	4.300775212
C	-3.027460158	2.328293957	2.340322087
H	-4.128273812	2.216132188	2.211967415
H	-2.593092633	1.389998336	1.929347383
B	4.506713408	-1.530881608	1.879383943

B	-4.206542085	-2.480161403	1.357855798
B	-0.965683602	3.649314330	1.801014258
C	3.359839452	0.240193017	4.122910415
H	2.393278654	0.703611512	4.430957086
H	4.085941950	0.549418940	4.907448221
H	5.409645770	-2.160560264	1.264585699
H	-5.165674002	-2.721103758	0.575240022

9.62. $[(\{C_8H_{14}\}BHO(Mes)CH)_3P_7]^{3-}$

Charge = –3 Multiplicity = 1

P	0.339981993	-0.760785812	-2.888223522
P	-1.706032784	0.123679756	-2.511472001
P	0.096681165	1.470424838	-2.649665169
P	0.763436127	-1.624166958	-0.840072756
P	-2.035659076	0.160279891	-0.265930209
P	0.910711167	1.803730658	-0.564458814
P	0.061711965	0.026038388	0.513717207
O	2.988296665	-2.693846674	0.229211629
O	-3.558283953	-1.288812185	1.398050747
O	0.925356158	4.336382285	0.710165875
C	2.740928606	-1.722975066	-0.715783388
H	3.064162082	-0.705938505	-0.390366409
C	3.288555525	-1.993580902	-2.124636770
C	3.950569731	-0.957473835	-2.846197106
C	4.194104409	0.408141116	-2.240021429
H	3.252297284	0.930577015	-1.962126204
H	4.746340636	1.059320178	-2.949607204
H	4.790635359	0.334094221	-1.305107262
C	4.417997308	-1.207305003	-4.154058513
H	4.925464901	-0.389083738	-4.697145085
C	4.260988227	-2.455808862	-4.779903110
C	4.720813056	-2.687608405	-6.204480211
H	3.886170664	-2.564445792	-6.933292276
H	5.120882540	-3.715344035	-6.346932345
H	5.516059028	-1.969819859	-6.499419691
C	3.631128440	-3.475882302	-4.042354466
H	3.505931024	-4.474863732	-4.499620057
C	3.149779516	-3.277094096	-2.733090761
C	2.539477553	-4.453902926	-2.004258730
H	3.249270718	-4.826665837	-1.235869562

H	2.304047648	-5.274166758	-2.716610768
H	1.620097490	-4.177271243	-1.451503422
C	4.412599292	-4.299030690	1.645932182
H	3.919404228	-5.172691194	1.152105058
C	3.572445334	-3.962458439	2.901140897
H	2.524652298	-3.815565010	2.557331507
H	3.544243494	-4.818817456	3.625751501
C	4.322916179	-1.476494268	2.750056004
H	4.797609894	-0.675233567	3.372009365
H	3.354462651	-1.052407747	2.403188800
C	5.160393210	-1.797700519	1.488298778
H	5.221143848	-0.852598355	0.894347726
C	6.622011432	-2.224260636	1.768263828
H	7.142738408	-2.286950979	0.783615635
H	7.177193933	-1.450434617	2.361169104
C	6.784252996	-3.588618970	2.484260776
H	7.852792198	-3.913714991	2.422671931
H	6.589313252	-3.457154741	3.571013433
C	5.874407119	-4.716528785	1.935753994
H	5.927361992	-5.585981970	2.644330300
H	6.312754691	-5.077548538	0.975318205
C	-2.973369337	-1.527535895	0.173341572
H	-2.178483931	-2.309400059	0.214424594
C	-3.929898374	-1.845952932	-0.984770031
C	-3.629682050	-2.905891226	-1.890487823
C	-2.412288209	-3.788132175	-1.713396206
H	-1.458718050	-3.218380181	-1.757271800
H	-2.376290037	-4.569317818	-2.501179625
H	-2.422051725	-4.295802338	-0.724764050
C	-4.496518660	-3.165493138	-2.972849299
H	-4.242904325	-3.989565686	-3.664531642
C	-5.664206274	-2.415014070	-3.194313624
C	-6.551563811	-2.673667691	-4.394392743
H	-6.462304310	-3.722275495	-4.751202198
H	-6.285766123	-2.015751433	-5.254341571
H	-7.622338205	-2.483070196	-4.163688682
C	-5.961517579	-1.393020462	-2.273860880
H	-6.881999760	-0.795368228	-2.408933167
C	-5.130260587	-1.097742230	-1.174598330
C	-5.572659139	-0.021156870	-0.208207773

H	-5.939988955	-0.488856006	0.729613462
H	-6.383896064	0.594081960	-0.654524289
H	-4.738313117	0.640896594	0.095853902
C	-5.028024622	-1.776645683	3.449535117
H	-5.686370281	-0.912187407	3.185284971
C	-3.923350298	-1.227552711	4.383798286
H	-3.441869451	-0.379811574	3.847730610
H	-4.351568476	-0.796310904	5.327149312
C	-2.818407252	-2.242666109	4.764929955
H	-1.960751232	-1.693496644	5.219334094
H	-3.188682865	-2.912056871	5.574525600
C	-2.298711399	-3.094336457	3.580567490
H	-1.649593570	-3.911291444	3.987318847
H	-1.625447419	-2.451899015	2.970699844
C	-3.390627789	-3.645656398	2.631333125
H	-2.857972258	-4.148163817	1.786570709
C	-4.314828890	-4.718673805	3.256890738
H	-4.954310020	-5.124056370	2.437487698
H	-3.732714966	-5.591748732	3.653532418
C	-5.246218272	-4.207438681	4.384223215
H	-6.024877135	-4.980951433	4.600053327
H	-4.664187006	-4.119034716	5.327224931
C	-5.939177673	-2.854660010	4.083695592
H	-6.421828336	-2.489500710	5.029497614
H	-6.774038817	-3.045643852	3.368457278
C	0.002969402	3.569349276	0.072787917
H	-0.786561369	3.165116443	0.742692358
C	-0.599396376	4.310329640	-1.112788353
C	-2.013531392	4.443189833	-1.234804879
C	-2.962638051	3.838959375	-0.224234092
H	-2.880624473	2.728451183	-0.188103547
H	-4.014167433	4.094905874	-0.469505696
H	-2.751699840	4.200920085	0.804881062
C	-2.551529454	5.172996233	-2.317355181
H	-3.650298499	5.257219687	-2.398751119
C	-1.740272193	5.794652699	-3.281463290
C	-2.340880253	6.526800221	-4.463525549
H	-2.416785030	5.867876648	-5.359134267
H	-1.725694265	7.403658247	-4.761446159
H	-3.367173425	6.889996845	-4.241873038

C	-0.345380591	5.681087187	-3.126726555
H	0.321503953	6.164750324	-3.863784821
C	0.240377703	4.961410017	-2.067305029
C	1.749997968	4.911992678	-1.974209670
H	2.115273264	5.536098918	-1.133856545
H	2.213581400	5.255092500	-2.923448787
H	2.114702398	3.887389201	-1.753062793
C	0.176565411	4.514341223	3.262777930
H	-0.833685338	4.091185011	3.052694801
C	0.063285037	6.076041484	3.285176522
H	-0.949518211	6.396111327	2.945798160
H	0.145519937	6.458179955	4.334926354
C	1.108057619	6.796318396	2.403642410
H	0.832584258	6.619746501	1.342659875
H	1.056092022	7.900544994	2.570507581
C	2.555712346	6.295136158	2.605628022
H	2.954478096	6.707936853	3.568046495
H	3.191643956	6.756827433	1.813951827
C	2.700649671	4.735637026	2.577625957
H	3.511134429	4.467420463	1.861700723
C	3.106058527	4.180115238	3.965794928
H	3.278903300	3.084646322	3.870060006
H	4.075039030	4.621849423	4.318523865
C	2.035079595	4.421661172	5.049801240
H	2.337463355	3.924074731	6.002092615
H	2.002238051	5.512377542	5.289013005
C	0.621855193	3.957420835	4.638966714
H	-0.096163529	4.243728646	5.451091831
H	0.609300595	2.845524670	4.589508147
B	4.434431336	-3.005495243	0.625796405
B	-4.341870419	-2.407716350	2.090695124
B	1.293644086	3.989441843	2.155823841
C	4.009090739	-2.687472682	3.662427279
H	3.206624525	-2.400597583	4.381858116
H	4.893774323	-2.916538886	4.299087476
H	5.113940131	-3.267052418	-0.407717293
H	-5.229743291	-2.854901580	1.309501999
H	1.405347763	2.739298841	2.217629241

10. References

(1) Cicač-Hudi, M.; Bender, J.; Schlindwein, S. H.; Bispinghoff, M.; Nieger, M.; Grützmacher, H.; Gudat, D. Direct Access to Inversely Polarized Phosphaalkenes from Elemental Phosphorus or Polyphosphides. *Eur. J. Inorg. Chem.* **2016**, *2016* (5), 649–658.
(2) Réant, B. L. L.; van Ijzendoorn, B.; Whitehead, G. F. S.; Mehta, M. Mapping Boron Catalysis onto a Phosphorus Cluster Platform. *Dalton Trans.* **2022**, *51* (47), 18329–18336.
(3) Kaden, F.; Metz, P. Enantioselective Total Synthesis of the Guaianolide (−)-Dehydrocostus Lactone by Enediyne Metathesis. *Org. Lett.* **2021**, *23* (4), 1344–1348.
(4) Bispinghoff, M. From Elemental Phosphorus to Functionalized Organophosphorus Compounds. ETH Zurich, 2017.
(5) Kuveke, R. E. H.; Barwise, L.; van Ingen, Y.; Vashisth, K.; Roberts, N.; Chitnis, S. S.; Dutton, J. L.; Martin, C. D.; Melen, R. L. An International Study Evaluating Elemental Analysis. *ACS Cent. Sci.* **2022**, *8* (7), 855–863.
(6) a) Sheldrick, G. SHELXT - Integrated space-group and crystal-structure determination. *Acta Crystallogr. Sect. A* **2015**, *71* (1), 3–8. b) Dolomanov, O. V.; Bourhis, L. J.; Gildea, R. J.; Howard, J. A. K.; Puschmann, H. OLEX2: A Complete Structure Solution, Refinement and Analysis Program. *J. Appl. Crystallogr.* **2009**, *42* (2), 339–341.
(7) a) Kohn, W.; Sham, L. J. Self-Consistent Equations Including Exchange and Correlation Effects. *Phys. Rev.* **1965**, *140* (4A), A1133–A1138. b) Hohenberg, P.; Kohn, W. Inhomogeneous Electron Gas. *Phys. Rev.* **1964**, *136* (3B), B864–B871. c) Peng, C.; Bernhard Schlegel, H. Combining Synchronous Transit and Quasi-Newton Methods to Find Transition States. *Isr. J. Chem.* **1993**, *33* (4), 449–454. d) Peng, C.; Ayala, P. Y.; Schlegel, H. B.; Frisch, M. J. Using Redundant Internal Coordinates to Optimize Equilibrium Geometries and Transition States. *J. Comp. Chem.* **1996**, *17* (1), 49–56. e) Gaussian 09, R. A., M. J. Frisch, G. W. Trucks, H. B. Schlegel, G. E. Scuseria, M. A. Robb, J. R. Cheeseman, G. Scalmani, V. Barone, G. A. Petersson, H. Nakatsuji, X. Li, M. Caricato, A. Marenich, J. Bloino, B. G. Janesko, R. Gomperts, B. Mennucci, H. P. Hratchian, J. V. Ortiz, A. F. Izmaylov, J. L. Sonnenberg, D. Williams-Young, F. Ding, F. Lipparini, F. Egidi, J. Goings, B. Peng, A. Petrone, T. Henderson, D. Ranasinghe, V. G. Zakrzewski, J. Gao, N. Rega, G. Zheng, W. Liang, M. Hada, M. Ehara, K. Toyota, R. Fukuda, J. Hasegawa, M. Ishida, T. Nakajima, Y. Honda, O. Kitao, H. Nakai, T. Vreven, K. Throssell, J. A. Montgomery, Jr., J. E. Peralta, F. Ogliaro, M. Bearpark, J. J. Heyd, E. Brothers, K. N. Kudin, V. N. Staroverov, T. Keith, R. Kobayashi, J. Normand, K. Raghavachari, A. Rendell, J. C. Burant, S. S. Iyengar, J. Tomasi, M. Cossi, J. M. Millam, M. Klene, C. Adamo, R. Cammi, J. W. Ochterski, R. L. Martin, K. Morokuma, O. Farkas, J. B. Foresman, and D. J. Fox, Gaussian, Inc., Wallingford CT, 2016.
(8) Sivaev, I. B.; Bregadze, V. I. Lewis Acidity of Boron Compounds. *Coord. Chem. Rev.* **2014**, *270−271*, 75−88.
(9) Alharbi, M. M.; van Ingen, Y.; Roldan, A.; Kaehler, T.; Melen, R. L. Synthesis and Lewis Acidity of Fluorinated Triaryl Borates. *Dalton Trans.* **2023**, *52* (6), 1820−1825.
(10) Christe, K.; Dixon, D.; McLemore, D.; Wilson, W.; Sheehy, J.; Boatz, J. On a Quantitative Scale for Lewis Acidity and Recent Progress in Polynitrogen Chemistry. *J. Fluor. Chem.* **2000**, *101*, 151–153.

(11) Erdmann, P.; Leitner, J.; Schwarz, J.; Greb, L. An Extensive Set of Accurate Fluoride Ion Affinities for p-Block Element Lewis Acids and Basic Design Principles for Strong Fluoride Ion Acceptors. *ChemPhysChem* **2020**, *21* (10), 987–994.
(12) Böhrer, H.; Trapp, N.; Himmel, D.; Schleep, M.; Krossing, I. From Unsuccessful H_2-Activation with FLPs Containing $B(Ohfip)_3$ to a Systematic Evaluation of the Lewis Acidity of 33 Lewis Acids Based on Fluoride, Chloride, Hydride and Methyl Ion Affinities. *Dalton Trans.* **2015**, *44* (16), 7489−7499.
(13) a) Bage, A. D.; Nicholson, K.; Hunt, T. A.; Langer, T.; Thomas, S. P. The Hidden Role of Boranes and Borohydrides in Hydroboration Catalysis. *ACS Catal.* **2020**, *10* (22), 13479−13486. b) Bage, A. D.; Hunt, T. A.; Thomas, S. P. Hidden Boron Catalysis: Nucleophile-Promoted Decomposition of HBpin. *Org. Lett.* **2020**, *22* (11), 4107−4112.
